# A Review of C_4_ Plants in Southwest Asia: An Ecological, Geographical and Taxonomical Analysis of a Region With High Diversity of C_4_ Eudicots

**DOI:** 10.3389/fpls.2020.546518

**Published:** 2020-11-05

**Authors:** Alexander Rudov, Marjan Mashkour, Morteza Djamali, Hossein Akhani

**Affiliations:** ^1^ Halophytes and C_4_ Plants Research Laboratory, Department of Plant Sciences, School of Biology, College of Sciences, University of Tehran, Tehran, Iran; ^2^ Archéozoologie, Archéobotanique: Sociétés, Pratiques et Environnements (AASPE/ UMR7209)—CNRS (Centre national de Recherche Scientifique) et MNHN (Muséum national d’Histoire naturelle), Paris, France; ^3^ Institut Méditerranéen de Biodiversité et d’Ecologie (IMBE/UMR7263), Aix Marseille Univ, Avignon Univ, CNRS, IRD, IMBE, Aix-en-Provence, France

**Keywords:** C_3_–C_4_ switching plants, C_4_ crops, Chenopodiaceae, conservation, Irano-Turanian region, Poaceae, single-cell C_4_, δ^13^C-isotope ratio

## Abstract

Southwest Asia is climatically and topographically a highly diverse region in the xeric belt of the Old World. Its diversity of arid habitats and climatic conditions acted as an important area for the evolution and diversification of up to 20 (of 38 known) independent Eudicot C_4_ origins. Some of these lineages present unique evolutionary strategies like single-cell functioning C_4_ and C_3_–C_4_ switching mechanisms. The high diversity of C_4_ taxa in Southwest (SW) Asia is also related to the presence of seven phytogeographic zones including the Irano-Turanian region as a center of diversification of many Caryophyllales lineages and the Somali-Masai region (Southern Oman and Yemen) as a center of diversification for C_4_ Monocots. Nevertheless, the C_4_ flora of SW Asia has not received detailed attention. This paper presents a comprehensive review of all known C_4_ species in the area based on a literature survey, own floristic observations, as well as taxonomic, phylogenetic and herbarium data, and δ^13^C-isotope ratio analysis. The resulting checklist includes a total number of 923 (861 native, of which 141 endemic, and 62 introduced) C_4_ species, composed of 350 Eudicots and 509 Monocots, most of which are therophytic and hemicryptophytic xerophytes with pluriregional and Irano-Turanian distribution. Two hundred thirty-nine new δ^13^C-isotope ratios of C_4_ and C_3_ plants, as well as some taxonomic changes are presented. An analysis of the distribution of the three main C_4_ plant families (Chenopodiaceae, Poaceae, and Cyperaceae) in the region in relation to climatic variables indicates that the increase of C_4_ species follows more or less a latitudinal gradient similar to global patterns, while separate taxonomic groups seem to depend on specific factors as continentality (Chenopodiaceae), average annual temperature (Cyperaceae), and the presence of summer precipitation (Poaceae). An increase of C_4_ Eudicots in W-E direction even in similar longitudinal belts is explained by a combination of edaphic and climatic conditions. The provided data should encourage a deeper interest in the evolution of C_4_ lineages in SW Asia and their adaptation to ecological and climatical conditions and awaken interest in the importance of local C_4_ crops, the conservation of threatened C_4_ taxa, and awareness of human impacts on the rapid environmental changes in the region.

## Introduction

Since its discovery, during the seventh decade of the twentieth century, the C_4_ photosynthetic pathway has received attention of extensive studies (Hatch, 1999). In contrast to C_3_ photosynthesis, which evolved under high atmospheric CO_2_ levels and mesic conditions, C_4_ photosynthesis developed under low CO_2_ levels and arid conditions. The climatic changes during the Oligocene (30–25 M.y.a.) and the following Miocene, marked by dropping CO_2_ levels and increasing seasonality with hot and dry periods and the resulting expansion of arid habitats, favored the convergent evolution and diversification of various C_4_ lineages (Sage, 2001; Osborne and Beerling, 2006; Christin et al., 2008; Sage, 2016). C_4_ photosynthesis involves a CO_2_ concentrating mechanism in hot and arid conditions through the activity of the phosphoenolpyruvate carboxylase (PEPC), an enzyme with a high affinity for $HCO_{3}^{-}$. The mechanism avoids photorespiration by concentrating CO_2_ levels around Ribulose-1,5-bisphosphate carboxylase/oxygenase (Rubisco) using a special dual compartmentation named Kranz-anatomy (Sage et al., 2012; Bräutigam and Gowik, 2016). This structure allows fixation of CO_2_ by PEPC in the mesophyll and its decarboxylation and concentration around Rubisco in the bundle sheath cells. Subsequently, it has been shown, however, that Kranz-anatomy is not always required for C_4_ photosynthesis in terrestrial plants (Voznesenskaya et al., 2001).

For the classification as a C_4_ plant, the δ^13^C ratio is a decisive indicator in all known fully functional C_4_ plants. It is related to differences in the fractionation of stable carbon isotopes ^12^C and ^13^C between C_3_ and C_4_ plants (O’Leary, 1988; Von Caemmerer et al., 2014). Based on the type of decarboxylating enzymes, C_4_ plants have been distinguished according to their metabolic type in NAD-dependent malic enzyme (NAD-ME)-type, NADP-dependent malic enzyme (NADP-ME)–type, and phosphoenolpyruvate carboxykinase (PEP-CK)-type (Pyankov et al., 2010). Recent studies, however, question this classification, indicating that C_4_ plants can be classified in relation to the malate-decarboxylating enzymes as NAD-ME or NADP-ME, while PEP-CK may be considered as an additional decarboxylating pathway (Bräutigam et al., 2014; Wang et al., 2014; Rao and Dixon, 2016).

C_4_ photosynthesis has been a metabolic revolution within the plant kingdom and a highly favorable metabolic pathway in plants growing under hot and arid conditions. In fact, while C_4_ plants comprise only 3% of vascular plants, they account for 25% of terrestrial photosynthesis (Sage, 2004; Osborne, 2010). Furthermore, this photosynthetic pathway evolved at least 64 times convergently in different plant families (Sage, 2016). Three most numerous C_4_ taxonomic groups can be distinguished: C_4_ Poaceae (with around 19 independent C_4_ clades including ca. 321 genera and over 5,000 species), C_4_ Cyperaceae (including 6 independent C_4_ clades in around 7 genera and over 1,300 species) and C_4_ Caryophyllales [including 24 independent C_4_ clades within 8 families (namely Amaranthaceae s. str., Aizoaceae, Caryophyllaceae, Chenopodiaceae, Gisekiaceae, Molluginaceae, Polygonaceae, and Portulacaceae] ca. 50 genera and over 1000 species) (Sage, 2016). The most interesting case in Caryophyllales is the particular diversity of independent C_4_ clades within the Chenopodiaceae family, that may include up to 13 independent C_4_ clades and 15 different C_4_ leaf anatomical types (Pyankov et al., 2001; Kadereit et al., 2003; Kadereit et al., 2012; Sage, 2016). [Following Hernandez-Ledesma et al., 2015; Walker et al., 2018, Chenopodiaceae is treated in this article as a separate family and not as a part of Amaranthaceae, although APG III and IV suggest it to be included in Amaranthaceae (APG III (The Angiosperm Phylogeny Group), 2009; APG IV (The Angiosperm Phylogeny Group), 2016)].

Previous studies have shown that different taxonomic groups of C_4_ plants [e.g., Monocots (Poaceae, Cyperaceae) and Eudicots (Chenopodiaceae)] follow different distributions in relation to climatic variables (Stowe and Teeri, 1978; Pyankov et al., 2010). In the case of the European continent, the distribution of C_4_ Monocots seems to be related to high temperatures, while the distribution of C_4_ Chenopodiaceae and several other C_4_ Eudicot lineages shows a relation to aridity (Pyankov et al., 2010). Similar tendencies can be observed also in other regions. E.g., the adaptation to aridity extends the dominion of C_4_ Chenopods to the highly continental Gobi desert and over 4,000 m altitude of the Pamir (Pyankov et al., 2000a). In tropical and subtropical Asia, Africa, Australia, and South America the majority of C_4_ Poaceae confirm the trend observed in Europe, being mainly distributed in hot climates with the obligatory presence of summer rainfall (Hattersley, 1983; Cabido et al., 2008; Ehleringer et al., 1987; Schulze et al., 1996; Wooler et al., 2001). C_4_ Cyperceae, finally, have been reported to be abundant in warm tropical temporary wetlands (Stock et al., 2004). The biodiversity of C_4_ plants has been so far reported for Europe (Pyankov et al., 2010) and China (Wang and Ma, 2016), while further studies on the distribution of C_4_ plants in relation to climatic and ecological parameters are available for various C_4_ lineages or specific geographical areas of the Old and New World (Raghavendra and Das, 1976; Teeri and Stowe, 1976; Tieszen et al., 1979; Waller and Lewis, 1979; Winter, 1981; Hattersley, 1983; Ehleringer et al., 1987; Batanouny et al., 1988; Medina et al., 1989; Schulze et al., 1996; Akhani et al., 1997; Rundel et al., 1999; Pyankov et al., 2000a; Wooler et al., 2001; Stock et al., 2004; Cabido et al., 2008; Mantlana et al., 2008).

The C_4_ flora of Southwest Asia is of great interest because of the remarkable diversity of C_4_ Eudicots and discovery of single-cell functioning C_4_ (Rechinger, 1963-2015; Nikitin and Geldikhanov, 1988; Akhani et al., 1997; Edwards et al., 2004; Akhani and Ghasemkhani, 2007). Southwest (SW) Asia in an extended sense including the Middle East, European parts of Turkey, Transcaucasia, Turkmenistan, Afghanistan, and Pakistan presents a topographically very diverse region.

Southwest and Central Asia have been proposed to be the origin of at least 20 of the 38 accepted C_4_ Eudicot lineages (Sage, 2011; Kadereit and Freitag, 2011; Sage, 2016). In fact, both regions can be considered an exceptional areas for the evolution and diversity of C_4_ Eudicots in a predominantly Monocot dominated “C_4_ world.” This and the high diversity of ecological and morphological features and adaptations of SW Asian C_4_ plants, as well as the quick degradation of the arid regions in the Middle East by improper agriculture, overgrazing, water mismanagement, desertification, and climate change (Motagh et al., 2008; Akhani, 2015b) and finally the need of detailed information on the C_4_ taxa of this region, inspired compilation of this work.

The aims of this work are: 1) to present an “as complete as possible” checklist of C_4_ species of SW Asia, with information on their respective ecological, floristic, and anatomical characteristics, where available; 2) to publish new δ^13^C stable isotope ratios for taxa with previously unpublished isotope data; 3) to analyze the distribution of C_4_ plants in SW Asia in relation to climatic variables and to evaluate different adaptations of the three main taxonomic groups of C_4_ plants (Chenopodiaceae, Cyperaceae, Poaceae) in relation to climate; 4) to discuss shortly the economical and ecological importance of major regionally cultivated or wild growing C_4_ crops in a region highly affected by climate change and desertification.

The biodiversity of C_4_ plants of Southwest Asia is of particular interest to comprehend the evolutionary history of C_4_ plants and the interaction of habitats, biogeographic regions, climate and soil in relation to C_3_^_^C_4_ domination. This allows to understand and predict future scenarios of the arid belts of the world, affected by global warming.

## Material and Methods

### Data Collection and Nomenclature

**Figure 1:**
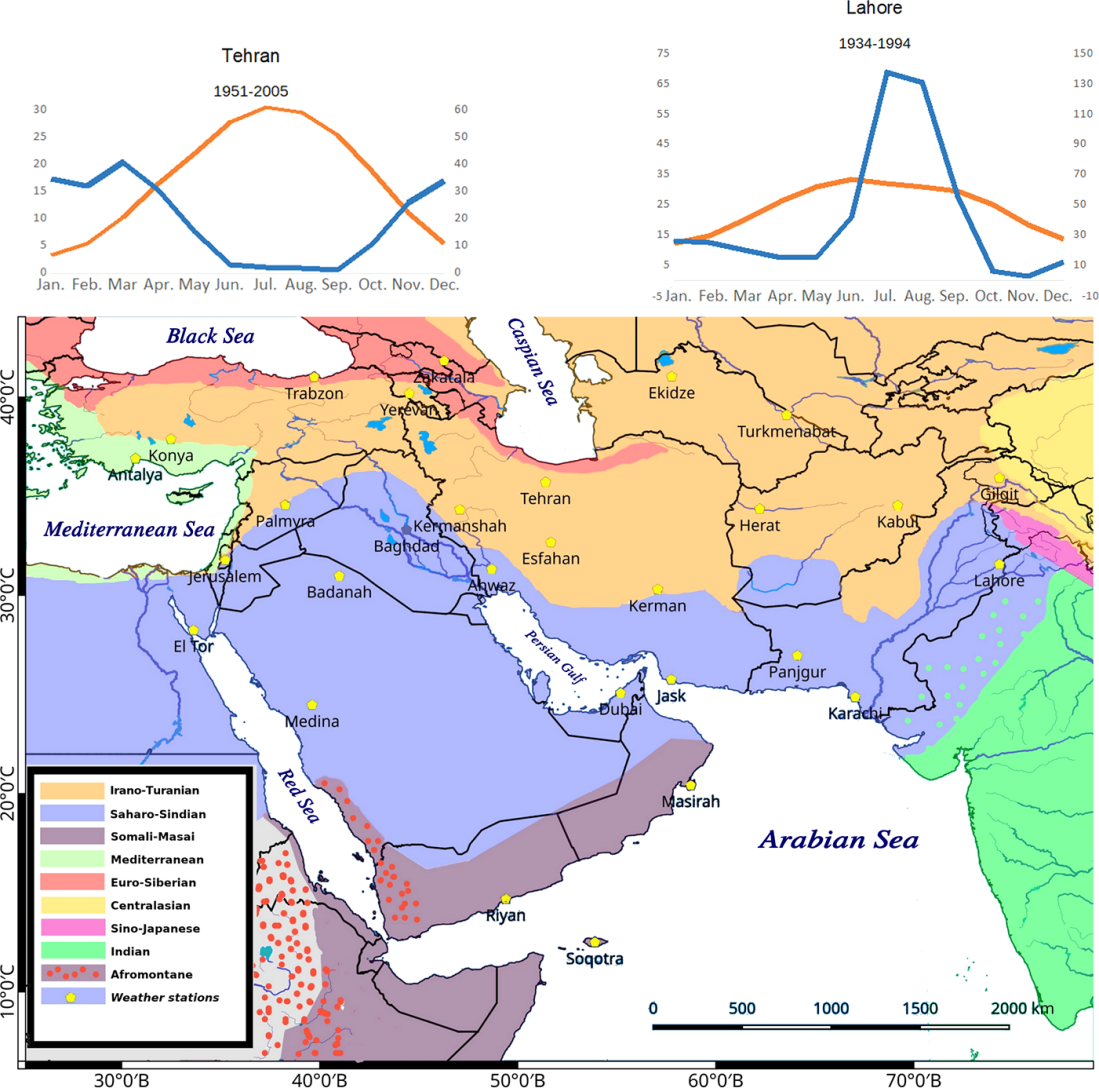
The study area includes Southwest (SW) Asia in an extended sense (**Figure 1**). Phytogeographic boundaries after White and Léonard (1991). We considered 29 weather stations for the evaluation of distribution of main C_4_ taxonomic groups in relation to climate. The two extreme climatic conditions showing winter rainfall regime (Tehran) and summer rainfall regime (Lahore) are shown. All other climatic diagrams are depicted in **Supplementary Figure 1**.

In this article, we treated SW Asia in an extended way (**Figure 1**). The geographical area considered in this article (extended Southwest Asia) includes the territories of the following countries and geographic areas: Afghanistan, Armenia, Azerbaijan, Bahrain, Iran, Iraq, Israel/Palestine, Jordan, Kuwait, Lebanon, Oman, Pakistan, Qatar, Saudi Arabia, Sinai Peninsula, Syria, Turkey, Turkmenistan, United Arab Emirates, and Yemen (**Figure 1**). The C_4_ species distribution data, habitat, and altitude preferences used for compilation of the checklist of C_4_ plants of SW Asia were obtained from standard floras, regional contributions, revisions, monographs, reports, and data bases and electronics sources (**Supplementary Appendix Table 1**). Data from Herbarium collections were obtained from the Herbarium of H. Akhani (Halophytes and C_4_ Plants Research Laboratory, School of Biology, University of Tehran), the Royal Botanic Garden Edinburgh Herbarium (E), and the Herbarium of Russian Academy of Sciences—V. L. Komarov Botanical Institute (LE). Finally, we received some unpublished data such as Cyperaceae of the Arabian Peninsula, kindly provided by Dr. David A. Simpson through personal communications (Royal Botanical Gardens Kew).

The published literature has been screened for all C_4_ species in the area, their respective habitat, distribution, life form, choro-, morpho-, and ecotypes. Taxonomic treatment and nomenclature of the C_4_ species were mainly based on global databases such as IPNI (2019) and POWO (2019). The naming of families followed the Angiosperm Phylogeny Group classification (APG IV, 2016) with the exception of the family Chenopodiaceae, which is treated as a separate family and not as a part of Amaranthaceae following Hernandez-Ledesma et al. (2015). The polymorphic genus *Calligonum* was treated taxonomically in accordance with the taxonomic simplifications proposed by Soskov (2011) and the genus *Tribulus* according to the simplifications proposed by Thomas and colleagues (Al-Hemaid and Thomas, 1996; Varghese et al., 2006) (see *Discussion* for further notes). Plants were classified as C_4_ species based on stable carbon isotope ratios (δ^13^C-values) as far as previous or own data support, leaf anatomy (presence of Kranz-anatomy), and biochemical subtypes (**Supplementary Appendix Table 1**). Species with no specific data available, but taxonomically belonging to pure C_4_ clades, were as well included in the list but marked with AR (analysis required). The C_4_ biochemical subtypes are based on published literature (**Supplementary Appendix Table 1**). In many cases we have extrapolated the “deduced” subtypes based on respective lineage unless there are evidences of multiple subtypes. Furthermore, species with lacking data, belonging to genera with both C_4_ and C_3_ clades and unknown attribution were preliminarily excluded from the list and listed separately (**Supplementary Table 2**). Life form, eco- and morphotype categorization for each species were based on the above-mentioned sources and/or own observations. The consideration of a species as native or introduced was based on distribution data and indications from the standard sources. Chorotypes were proposed based on a species distribution data in relation to the boundaries of the phytochoria. We used the phytogeographical system suggested for SW Asia and Africa by White and Léonard (1991) and considered other references such as Zohary (1973); Takhtajian (1992); Miller and Cope (1996); Djamali et al. (2012); Welk (2015) (**Figure 1**).

### δ^13^C Analysis

A total of 234 plant samples (**Supplementary Appendix Table 1**) with unpublished δ^13^C-values (^13^C/^12^C ratios) have been sampled from herbarium samples. The δ^13^C were analyzed according to the standard procedure relative to PDB (Pee Dee Belemnite) limestone as the carbon isotope standard and calculated according to this formula: δ = 1,000 x (R_sample_/R_standard_ − 1) (Osmond et al., 1975; Akhani et al., 2009). The samples have been fine ground using a Retsch ball grinder and transferred in microtubes for isotopic measurement. Each sample was weighted to a mass between 1.50 to 1.90 mg at the SSMIM Mass Spec Lab of the National Museum of Natural History of Paris and burnt in an automated combustion system (EA Flash 2000 Thermo device), interfaced with a DeltaV Advantage Thermo isotope ratio mass spectrometer (continuous flow). The analytical uncertainty within each run estimated from repeated analyses of our laboratory standard (alanine, normalized to IAEA caffeine-600) was lower than 0.08‰ (k = 1) for δ^13^C values.

### Climate Data and Statistical Analysis

For the correlation of C_4_ taxonomic group distributions within the study area and climatic variables, bioclimatic data were extracted from the Worldwide Bioclimatic Classification System (Rivas-Martinez and Rivas-Saenz, 1996-2019; Djamali et al., 2011). For a few stations we obtained climatic data of the Iranian Meteorological Organization (IRIMO) and Scholte and De Geest (2010) and Raza et al. (2015) (**Supplementary Figure 1**). Variables, representative for the SW Asia, have been extracted and/or calculated:

- Mean annual daily temperature (T)
- Mean annual precipitation (P)
- Continentality index [I_c_=T_max_ (mean temperature of warmest month) − T_min_ (mean temperature of coldest month)]
- De Martonne Annual Aridity Index [P/(T+10)]
- Duration of dry season (number of months with P<2T)
- Mean summer precipitation (P_s_—mean precipitation of warmest 3 months)
- Ombrothermic index of summer (Ios_3_=P_p3_/T_p3_*10), where P_p3_ is the precipitation of the whole summer and T_p3_ the sum of the mean temperatures for each month of the summer).

The distribution and diversity of C_4_ plants in relation to climatic variables (annual mean daily temperature, annual mean precipitation, De Martonne aridity index, continentality index, mean summer precipitation, duration of dry season, and ombrothermic index of summer) has been calculated by linear correlation analysis. The correlation is considering the number of total C_4_ plant species, as well as the numerically and ecologically most important taxonomic groups of C_4_ plants, e.g., number of C_4_ Poaceae, C_4_ Cyperaceae and C_4_ Chenopodiaceae, and the C_4_ Monocot/Eudicot ratio. The data have been imported into “OriginPro,” which has been used to calculate the linear correlation and Pearson correlation coefficient and for graphical design (Origin Pro, 2017).

## Results

A complete list of all SW Asian C_4_ species with life form, chorotype, ecotypes, δ^13^C-values, metabolic and Kranz anatomical subtypes is presented in **Supplementary Appendix Table 1**.

### General Statistics

**Figure 2:**
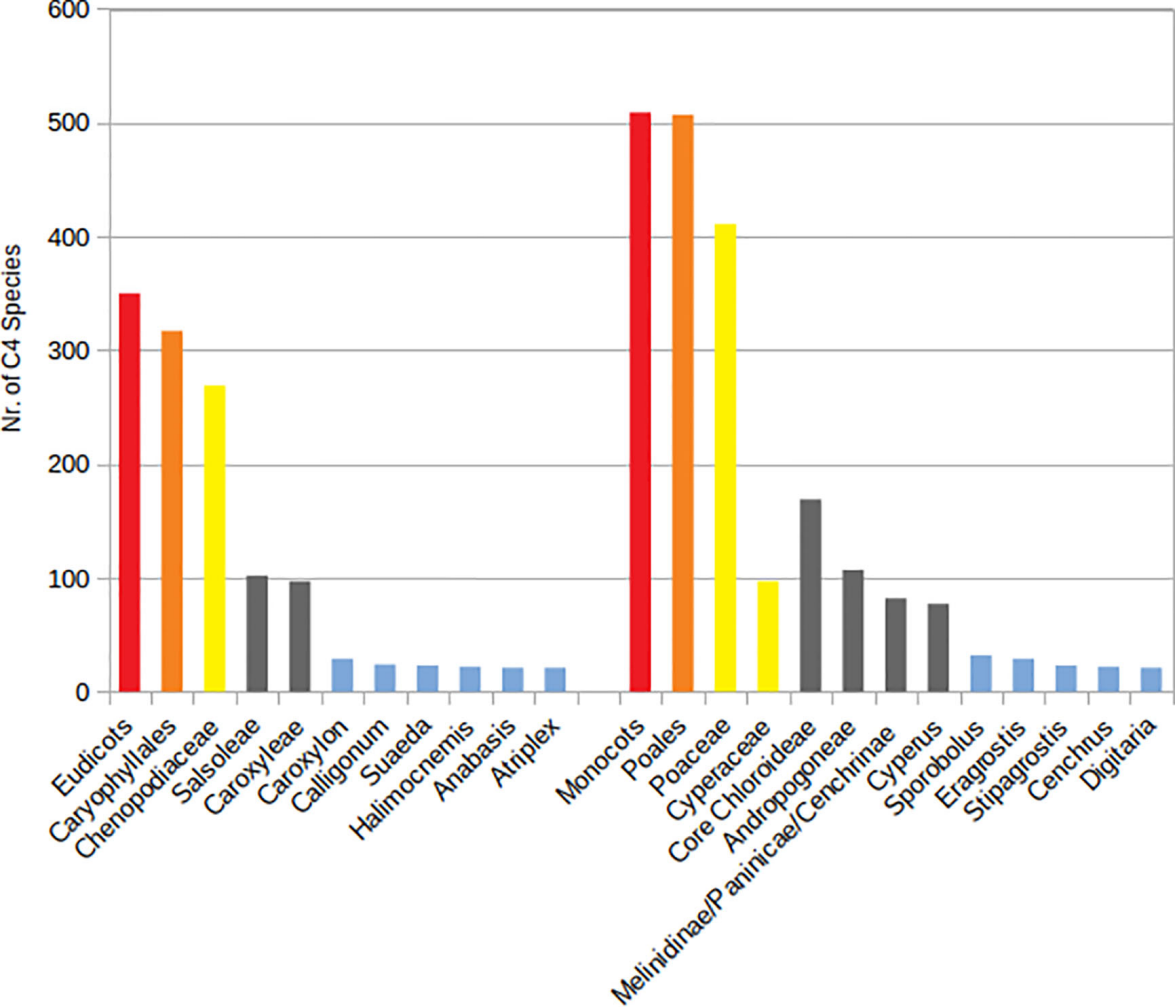
Main taxonomic groups of Southwest (SW) Asian C_4_ plants. Red—clades, orange—species richest orders, yellow—species richest families, gray—species richest independent C_4_ lineages, blue—genera with more than 20 species.

A total number of 923 (861 native and 62 introduced) C_4_ species belonging to 166 genera, 48 independent C_4_ lineages, 19 families, and 9 orders have been known from extended SW Asia (**Supplementary Appendix Table 1**, **Figure 1**). For the taxonomic diversity of SW Asian C_4_ plants view **Figure 2**.

In the case of *Polycarpaea* (a polyphyletic genus with both C_3_ and C_4_ species), we have only included one species in our list, considering the fact that we could not verify the photosynthetic pathway of seven additional species, reported from the area (mostly from Socotra). A list of these species is given in the **Supplementary Table 2**.

### Distribution of C_4_ Species by Country

**Figure 3:**
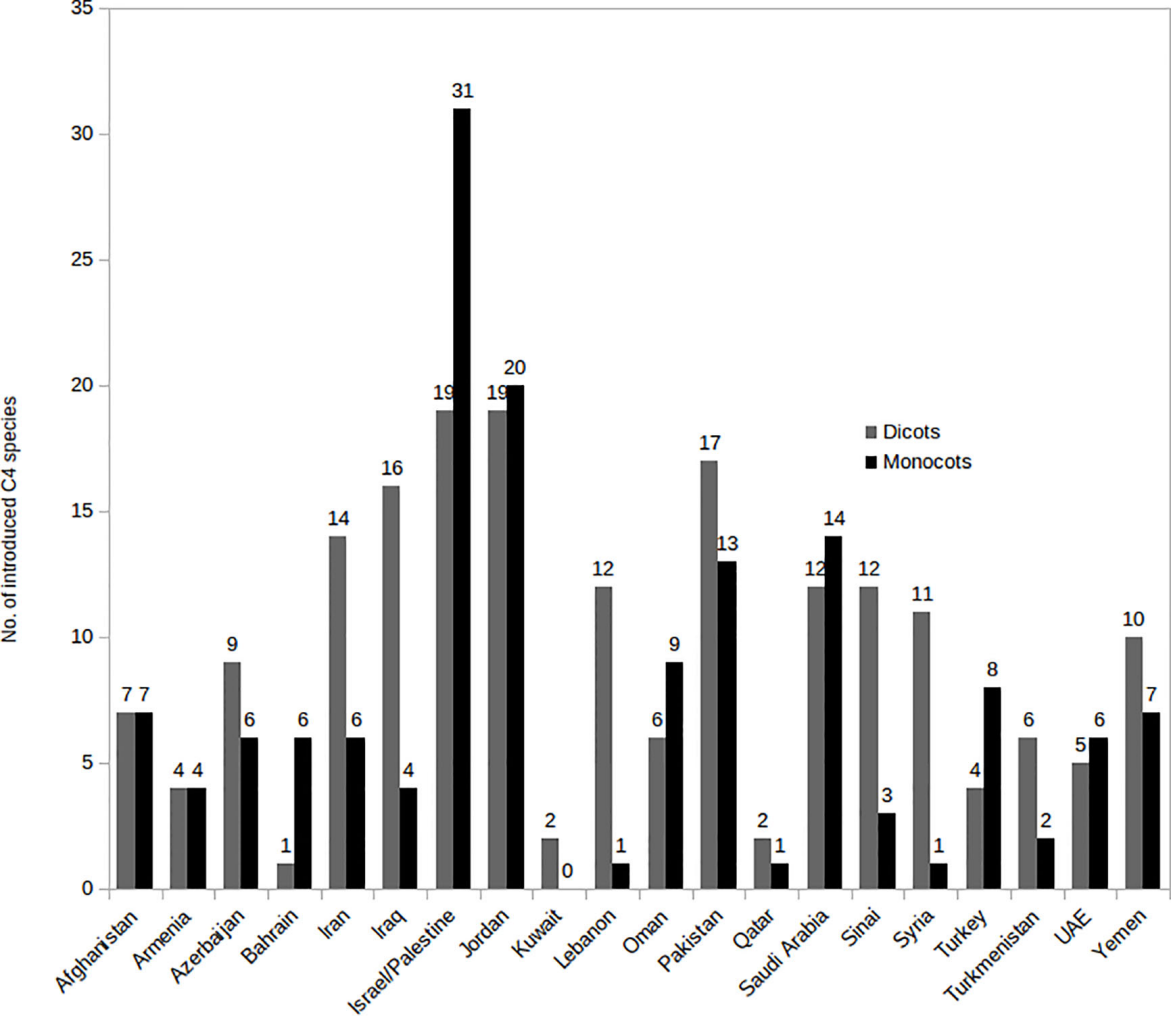
Distribution of introduced C_4_ Monocots and Eudicots per country in various Southwest (SW) Asian countries.

**Figure 4:**
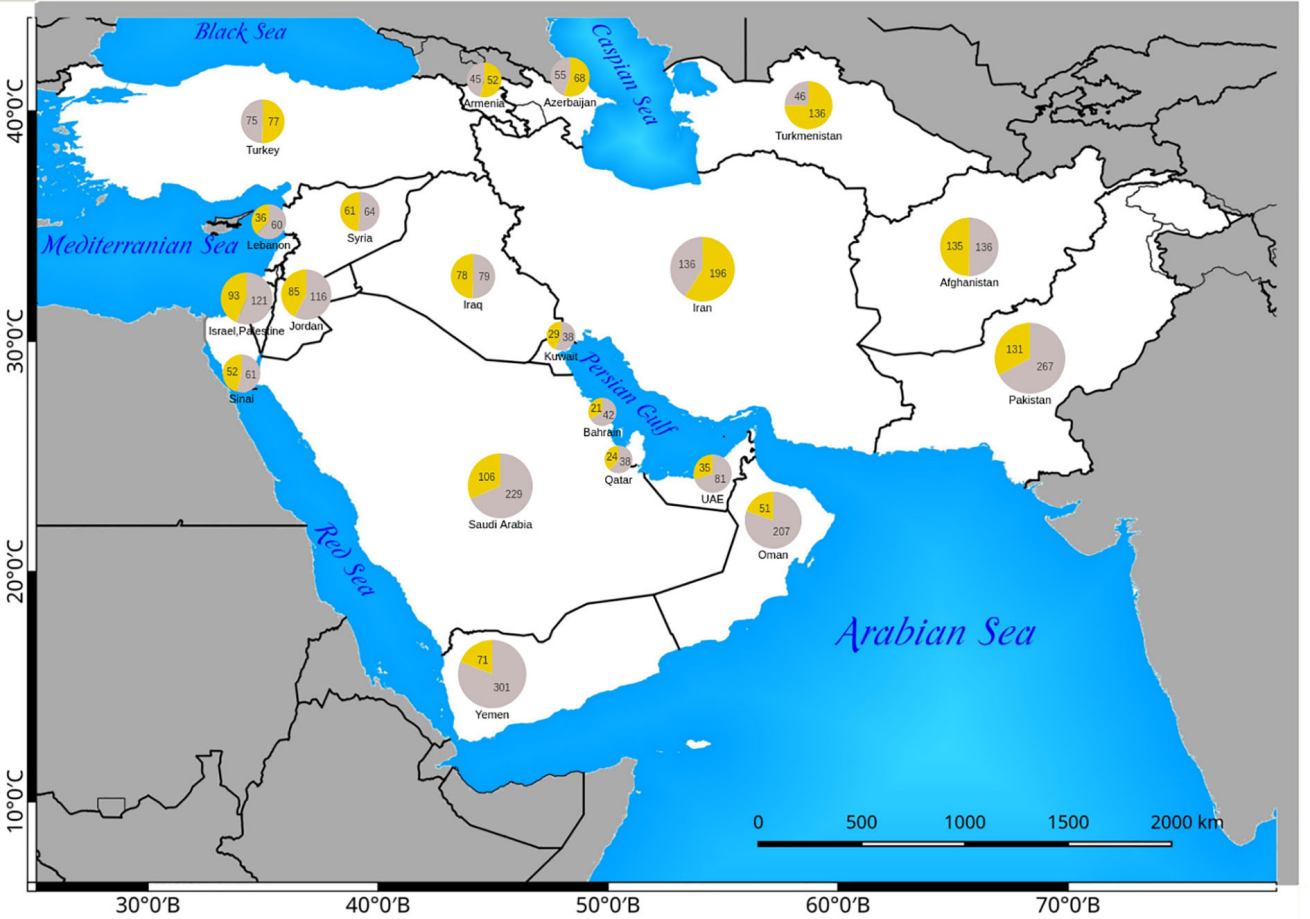
Distribution of native Eudicot (yellow) and Monocot (gray) species in Southwest (SW) Asian countries. The absolute number of C_4_ Monocots and Eudicots are indicated in the respective pie charts.

The number of native and introduced C_4_ species in individual SW Asian countries/regions and respective Monocot/Eudicot proportions are shown in **Figures 3** and **4** respectively. The highest diversity of C_4_ plants has been documented for Pakistan (account for 43% of all known native SW Asian species), Yemen (38%), Iran (36%), Saudi Arabia (36%), Afghanistan (30%), and Oman (28%), respectively. The differences in the proportion of C_4_ Monocot/Eudicots allowed us to categorize countries into three groups: 1) countries with remarkably high percentage of C_4_ Eudicots, such as Turkmenistan and Iran; 2) countries with more or less equal proportion of Monocot/Eudicots such as Armenia, Azerbaijan, Afghanistan, Turkey, Syria, Iraq, and Sinai Peninsula; 3) counties with higher percentage of C_4_ Monocots, that is all other countries located in southern parts of the region.

**Figure 5:**
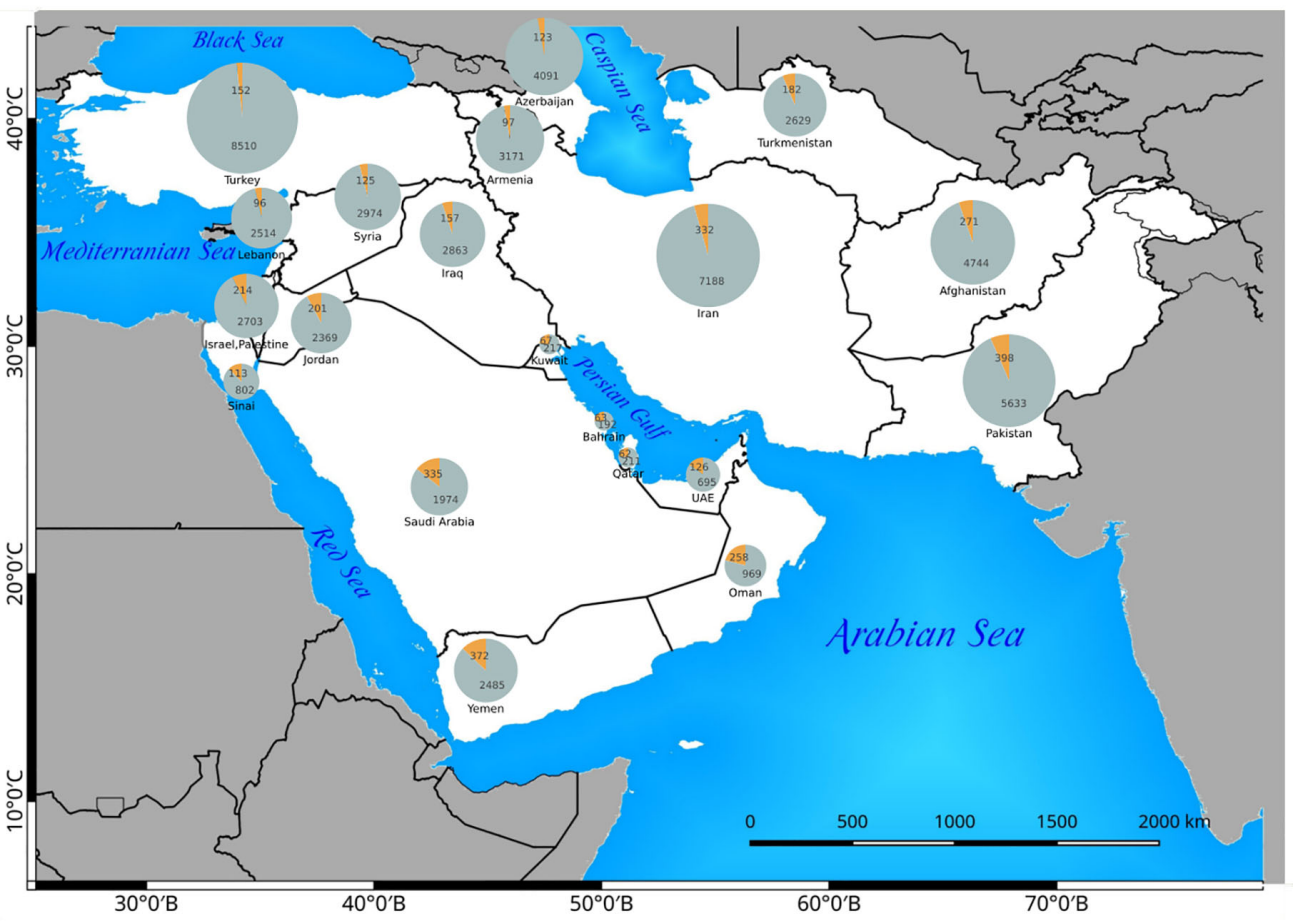
The distribution of C_4_ plants (orange) in relation to the total flora (gray) of any Southwest (SW) Asian country. The absolute number of C_4_ and C_3_ species are indicated in the related pie charts.

The countries with the highest number of introduced C_4_ plants are Israel and Palestine with 50 introduced species, Jordan with 39 introduced species, and Pakistan with 30 introduced species respectively. The percentage of C_4_ plants in relation to total number of recorded plant species per country is highest in Kuwait (23%), Bahrain (22.5%), Qatar (22%), and Oman (20%), respectively (**Figure 5**).

### C_4_ Endemics of Southwest Asia

One hundred forty-one C_4_ species (36 Monocots and 105 Eudicots, 93 of which are Chenopodiaceae) are endemics of SW Asia; 74 of those (22 Monocots and 52 Eudicots) are strict “country endemics.” The highest number of “country endemics” are documented in Iran (27 species), Yemen (14 species, 9 of which are endemic to the island of Socotra), Afghanistan (8 species), and Oman (7 species), respectively. The highest number of endemism occurs in Chenopodiaceae (93 species) and Poaceae (31 species). The three endemic richest genera (with 10 or more endemic species) are *Halothamnus* (12 endemic sp. in SW Asia), *Halimocnemis*, and *Climacoptera* (respectively 10 endemic sp. in SW Asia).

The only generic C_4_ endemic of the area is the monotypic genus *Halarchon* (*Halarchon vesiculosum*) restricted to Afghanistan. Except a few old records outside of SW Asia, the range of three known species of *Bienertia* is limited to this area.

### Climate Correlation

**Figure 6:**
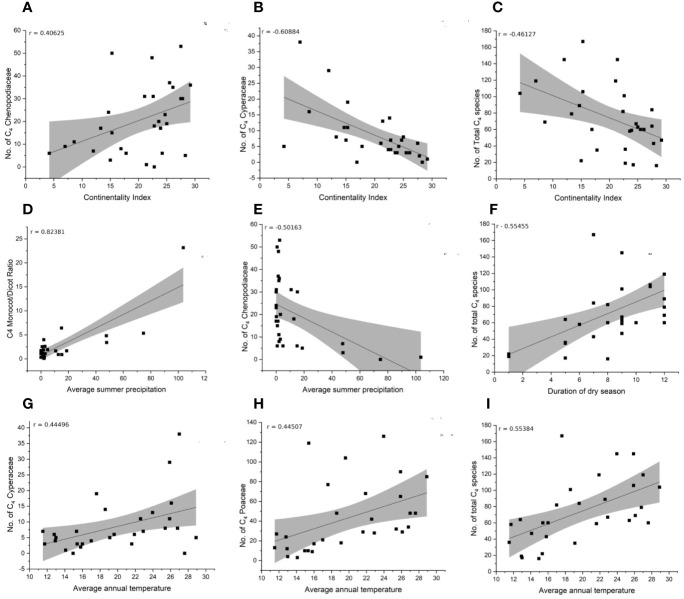
Correlation of climate variables and distribution of C_4_ main taxonomic groups (Chenopodiaceae, Cyperaceae, Poaceae, Monocot/Eudicot ratio). **(A–C)** Continentality index. **(D, E)** Average summer precipitation. **(F)** Duration of dry season. **(G–I)** Average annual temperature.

The species-richness of C_4_ Chenopodiaceae increases with increasing continentality and decreases with increasing mean summer precipitation (**Figures 6A, E**). The C_4_ richness of Cyperaceae increases with increasing mean annual temperature and is negatively affected by increasing continentality (**Figures 6B, G**). The number of C_4_ Poaceae increases with increasing mean annual daily temperature (**Figure 6H**). Totally, the diversity and abundance of C_4_ plants increases with increasing annual daily temperature and duration of the dry season and decreases with increasing continentality (**Figures 6C, F, I**). Finally, the prevalence of Monocots over Eudicots is related to increasing average summer precipitation (**Figure 6D**).

### Life Forms and Ecotypes

**Figure 7:**
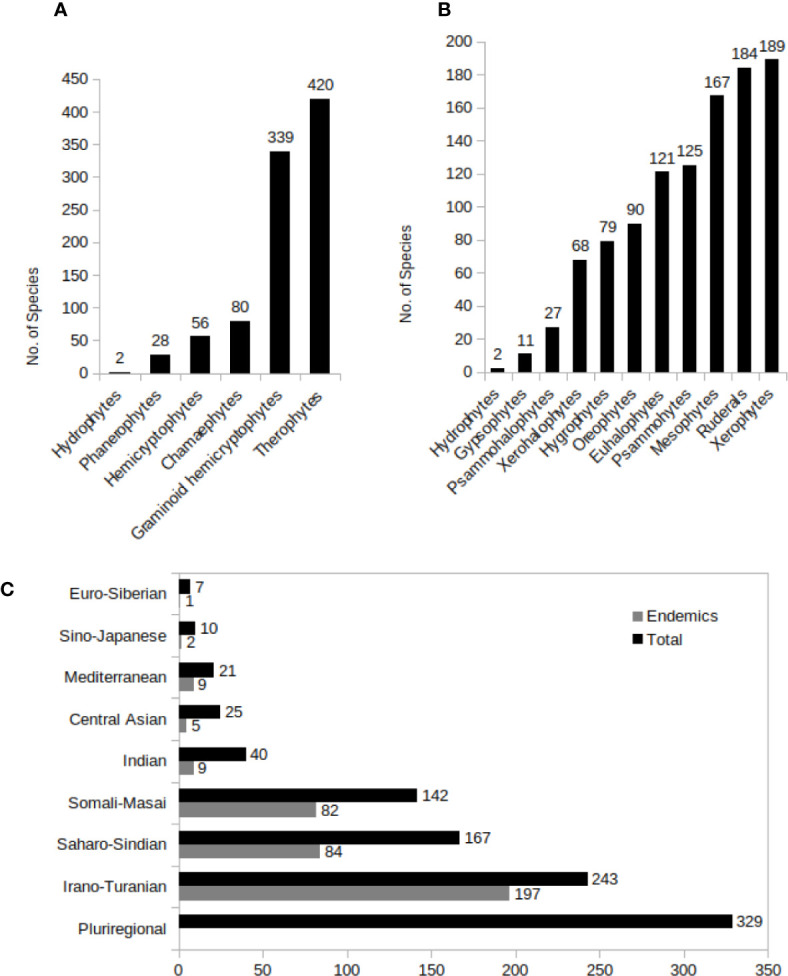
**(A–C)** C_4_ Species distribution in different growth types **(A)**, predominant ecotypes **(B)**, and different phytogeographic regions in Southwest (SW) C_4_ plants. A plant was considered as pluriregional, when occur in more than three phytochorion, and as endemic, when present in just one phytochorion.

Life forms and ecotypes of the C4 plants are shown in **Figures 7A, B**.

### Phytogeographic Distribution of C_4_ Species of Southwest Asia

The phytogeographic distribution of C_4_ species of SW Asia are shown in **Figure 7C**.

### Occurrence of C_4_ Biochemical Subtypes

Based on major taxonomic groups (C_4_ Poaceae, C_4_ Cyperaceae, and C_4_ Eudicots), the division in metabolic subtypes is as follows. Within the C_4_ Eudicots NAD-ME metabolic types occur in the *Cleome gynandra*, *Cleome angustifolia*, *Amaranthus* spp., C_4_ *Atriplex*, Caroxyleae tribe, *Bienertia*, *Suaeda* sect. *Salsina*, *Suaeda* sect. *Schoeberia*, *Calligonum*, *Gisekia*, *Hypertelis*, *Blepharis*, *Anticharis*, and *Tetraena simplex* C_4_ lineages (ca. 213 species). The NADP-ME subtype occurs in *Flaveria* clade A, *Euploca*, C_4_ *Polycarpaea*, C_4_ *Aerva*, *Gomphrena*, C_4_ *Althernanthera*, C_4_ *Camphorosmeae*, C_4_ *Salsoleae*, *Boerhavia*, *Euphorbia* subgen. *Chamaesyce*, and *Tribulus/Kallstroemia* C_4_ lineages (ca. 167 species). C_4_ Sesuvioideae and *Portulaca* include both NADP-ME and NAD-ME metabolic types.

Within Poaceae a NAD-ME subtype (including mixed NAD-ME/PEP-CK subtypes) is present in *Centropodia*, Melinidinae, Panicinae, and core Chloroideae (ca. 213 species), while a NADP-ME subtype (including mixed PEP-CK/NADP-ME subtypes—for further information consult **Supplementary Appendix Table 1**) is present within *Alloteropsis*, tribe Andropogoneae, *Aristida*, tribe Arundinelleae, Tristachyideae, *Digitaria*, tribe Cenchrinae, *Paspalum*, and *Stipagrostis* (228 species). Within the Cyperaceae (C_4_ *Cyperus*, C_4_ *Fimbristylis*, and *Bulbostylis* C_4_ lineages, 100 species) and within the Hydrocharitaceae (*Hydrilla* and *Elodea* C_4_ lineages) all species known to perform NADP-ME metabolic type.

### Isotope Data

Two hundred thirty-four δ^13^C-isotope ratios, mainly for species with previously unpublished data, are presented in the **Supplementary Appendix Table 1** (data marked with * and +). Additionally, the δ^13^C-isotope ratios of the C_3_ plants *Polycarpaea caespitosa* Balf., *Polycarpaea spicata* Wight ex Arn., *Polycarpaea repens* (Forsskal) Aschers. and Schweinf., *Cyperus pulcherrimus* Willd. ex Kunth, and *Fimbristylis turkestanica* (Regel) B. Fedtsch., distributed in Southwest Asia, are published in **Supplementary Table 3**.

## Discussion

### Southwest Asia Center of Origin of Major C_4_ Flora

The checklist appended in this paper has been compiled with great caution to include as much data as available, thanks to intensive Flora compilation in SW Asian countries published during more than half a century (Rechinger, 1963-2015; Davis, 1966-2001; Guest and Ghazanfar, 1966-2013; Nasir and Ali, 1970-2003; Nikitin and Geldikhanov, 1988; Miller and Cope, 1996) and intensive botanical explorations in the region. However, as a first contribution, it requires more data to fill some gaps on local flora of the Levant (Syria, Jordan and Lebanon) and the absence of up-to-date information on specific groups such as Cyperaceae in the Arabian Peninsula.

In spite of our efforts to check photosynthetic types of all putative C_4_ groups, we could not get enough samples for a few *Polycarpaea* species. The preliminary phylogenetic studies show that *Polycarpaea* is polyphyletic including both C_3_ and C_4_ species. It has been suggested to segregate C_4_ species in a separate genus *Polia* (Kool, 2012). Within our area, however, only *Polycarpaea corymbosa* (L.) Lam. is a reliable C_4_ species. Some other SW Asian species such as *P. repens* (Forsskal) Aschers. and Schweinf., *P. spicata* Wight ex Arn., *P. hassalensis* Chamberlain, and *P. haufensis* A.G. Miller) have been reported to be C_3_ according to Kool (2012) and a few ones (**Supplementary Table 3**) require further investigation.

So far 174 species of *Calligonum* have been described worldwide (Soskov, 2011), however phylogenetic studies and attempts to barcode these species revealed little information to support this diversity (Tavakkoli et al., 2010; Li et al., 2014; Doostmohammadi et al., 2020). We followed the recent monography of the genus which accepts a wide species concept including only 28 species and 8 interspecific hybrids (Soskov, 2011) and recent minor changes by Shi et al. (2016).

The polymorphic, mainly Saharo-Sindian genus *Tribulus*, has been partly reviewed for India and Saudi Arabia by synonymizing many species (Al-Hemaid and Thomas, 1996; Varghese et al., 2006). A systematic review of the genus in our area is highly welcome.

Despite recent progress in the taxonomy and phylogeny of Chenopodiaceae (Kadereit et al., 2003; Akhani et al., 2007), still there are ambiguities in monophyly of some genera such as *Hammada*. The phylogenetic tree based on combined nuclear and chloroplast markers showed polyphyly of three species *Hammada articulata*, *H. salicornica*, and *H. griffithii* (Akhani et al., 2007; Schüssler et al., 2017). In a long debate on the nomenclatural status of the *Kali*-clade within Salsoleae which has been separated from *Salsola* s.l. based on strong molecular and morphological data, recent unexpected decision on replacing the type of the genus *Salsola* by *Salsola kali* L. instead of *S. soda* by International Code of Nomenclature (Akhani et al., 2014; Mosyakin et al., 2017; Turland et al., 2018) resulted in instability and chaos of all names used since 2007. Therefore, in order to keep phylogenetic classification of Salsoloideae we are pushed to change the name of many species traditionally classified in *Salsola* into *Soda* (see **Nomenclatural Appendix**). Furthermore, we provide new combinations for some species which have been overlooked in the phylogenetically based system of Salsoleae by Akhani et al. (2007).

The phylogenetic relationships within Poaceae have seen several recent revisions (Hodkinson et al., 2002; Kellogg, 2015; Soreng et al., 2015; Soreng et al., 2017). These revisions regarded also changes within the PACMAD clade (Panicoideae, Arundinoideae, Chloridoideae, Micrairoideae, Aristidoideae, and Danthonioideae subfamiles), which includes all C_4_ Poaceae lineages. While some phylogenetic relationships may need further clarification (e.g., genera *Saccharum*, *Miscanthus*, *Miscanthidium*, etc.), several clades have been synonymized [e.g., *Urochloa* (=*Brachiaria, Snowdenia*)] or separated (e.g., *Narenga* separated from *Saccharum*). In the nomenclatural appendix we provide new combinations for the species that have been affected by the last revision by Soreng et al. (2017).

Based on our data (**Table 1**), the C_4_ flora of SW Asia includes 923 (ca. 11% of world known C_4_ species) and represent 48 of 65 known C_4_ lineages of the world (Sage, 2016; Akhani et al., 2007; Kadereit and Freitag, 2011). The area, as one of the major center of diversity of C_4_ Eudicots, harbors the origin of ca. 19 C_4_ Eudicot lineages and has representatives of all families known to have C_4_ species either as native or introduced (Sage, 2016) (**Table 1**, **Supplementary Appendix Table 1**).

**Table 1:** C4 Lineages and number of species in each genus in 20 Southwest (SW) Asian countries.

Lineage	Genus	Distribution in SW Asia																				
		Afghanistan	Armenia	Azerbaijan	Bahrain	Iran	Iraq	Israel/Palestine	Jordan	Kuwait	Lebanon	Oman	Pakistan	Qatar	Saudi Arabia	Sinai	Syria	Turkey	Turkmenistan	UAE	Yemen	
**Monocots**																						
**ALISMATALES**																						
**Hydrocharitaceae**																						
***1) Egeria***	***Elodea***																	**1**				
***2) Hydrilla***	***Hydrilla***	**1**				**1**							**1**									
**POALES**																						
**Cyperaceae**																						
***3) Bulbostylis***	***Bulbostylis***												**2**					**2**			**4**	
***4) C4 Cyperus***	***Cyperus***	**22**	**6**	**12**	**6**	**24**	**16**	**17**	**14**	**4**	**10**	**22**	**46**	**16**	**23**	**9**	**11**	**15**	**5**	**9**	**39**	
***5) C4 Fimbrystylis***	***Fimbristylis***	**4**	**1**	**3**		**3**	**3**	**3**	**3**		**2**	**3**	**13**		**4**	**2**	**3**	**3**	**1**	**2**	**6**	
**Poaceae**																						
***6) C4 Alloteropsis***	***Alloteropsis***												**1**									
***7) Andropogoneae***	***Andropogon***							**1**	**1**		**1**	**1**			**1**	**1**	**1**	**1**			**6**	
	***Apluda***	**1**					**1**					**1**	**1**								**1**	
	***Arthraxon***	**3**	**1**			**1**						**6**	**3**		**1**			**1**			**3**	
	***Bothriochloa***	**3**	**2**	**2**		**2**	**1**	**1**			**1**	**3**	**3**		**2**		**1**	**1**	**1**		**2**	
	***Capillipedium***											**1**	**2**									
	***Chrysopogon***	**2**	**1**	**1**		**2**	**1**	**1**	**1**		**1**	**3**	**5**	**2**	**2**		**1**	**1**			**3**	
	***Cleistachne***											**1**										
	***Coix***	**1**																				
	***Cymbopogon***	**3**			**3**	**2**	**2**	**2**	**2**		**1**	**4**	**6**	**1**	**3**		**2**			**2**	**5**	
	***Dichanthium***	**1**			**2**	**2**	**1**	**2**	**2**	**2**		**4**	**2**	**2**	**3**	**2**		**1**		**2**	**3**	
	***Diectomis***																				**1**	
	***Dimeria***											**1**										
	***Elionurus***					**1**	**1**					**1**	**1**		**1**						**2**	
	***Euclasta***											**1**										
	***Eulaliopsis***	**1**											**1**									
	***Hackelochloa***											**1**									**1**	
	***Hemarthria***	**1**					**1**	**1**	**1**		**1**		**2**		**1**		**1**	**1**				
	***Heteropogon***	**1**				**1**	**1**				**1**	**2**	**1**		**1**						**1**	
	***Hyparrhenia***	**1**			**1**	**1**	**1**	**1**	**1**		**1**	**1**	**1**		**1**	**1**	**1**	**1**			**7**	
	***Imperata***	**1**	**1**	**1**		**1**	**1**	**1**	**1**	**1**	**1**	**1**	**1**		**1**	**1**	**1**	**1**	**1**		**1**	
	***Ischaemum***												**2**		**1**						**1**	
	***Iseilema***												**1**									
	***Lasiurus***	**1**				**1**	**1**	**1**	**1**	**1**		**1**	**1**	**1**	**1**					**1**	**1**	
	***Microstegium***			**1**		**1**							**2**									
	***Miscanthus***	**1**																				
	***Mnesithea***	**1**											**1**									
	***Narenga***												**1**									
	***Ophiuros***												**1**									
	***Phacelurus***	**1**						**1**	**1**		**1**		**1**				**1**	**1**				
	***Pogonatherum***	**1**											**2**		**1**							
	***Polytoca***												**1**									
	***Pseudodichanthium***											**1**										
	***Pseudopogonatherum***												**1**									
	***Rottboellia***											**1**									**1**	
	***Saccharum***	**4**				**3**		**1**	**1**		**1**	**2**	**5**		**2**	**1**	**1**		**1**	**1**	**2**	
	***Schizachyrium***											**1**	**2**									
	***Sehima***											**1**	**2**		**1**						**2**	
	***Sorghum***	**1**	**1**	**1**		**1**	**1**	**2**	**2**	**1**	**2**	**2**	**3**	**1**	**2**	**2**	**2**	**1**	**1**	**1**	**1**	
	***Spodiopogon***						**1**	**1**	**1**		**1**		**1**				**1**	**1**				
	***Thelepogon***												**1**									
	***Themeda***	**1**					**1**				**1**	**1**	**1**		**2**		**1**	**2**			**1**	
	***Tripidium***	**2**	**1**	**1**		**2**	**2**	**2**	**2**		**3**	**1**	**2**		**1**		**3**	**2**	**1**	**1**	**1**	
***8) Aristida***	***Aristida***	**2**	**1**	**1**	**1**	**2**	**1**	**2**	**2**		**1**	**7**	**7**	**3**	**9**	**2**	**1**	**1**	**1**	**4**	**12**	
***9) Centropodia***	***Centropodia***	**1**			**1**	**1**	**1**	**1**	**1**	**1**		**1**		**1**	**2**	**1**				**2**	**2**	
***10) Core Chloridoideae***	***Acrachne***	**1**										**1**	**1**		**1**						**1**	
	***Aeluropus***	**3**	**2**	**2**	**2**	**3**	**2**	**2**	**2**	**2**	**1**	**1**	**3**	**1**	**2**	**2**	**2**	**2**	**3**	**2**	**1**	
	***Chloris***	**2**		**1**	**3**	**2**	**2**	**4**	**4**	**1**		**5**	**5**	**3**	**4**				**1**	**4**	**5**	
	***Cleistogenes***	**1**	**1**	**1**		**1**							**1**					**1**	**2**			
	***Coelachyrum***					**1**						**3**	**1**		**3**					**2**	**3**	
	***Ctenium***														**1**							
	***Cynodon***	**1**	**1**	**1**	**1**	**2**	**1**	**1**	**1**	**1**	**1**	**1**	**2**	**1**	**2**	**1**	**1**	**1**	**1**	**1**	**1**	
	***Dactyloctenium***	**2**			**1**	**2**	**1**	**1**	**1**	**2**	**1**	**4**	**3**	**1**	**3**	**1**	**1**	**1**		**2**	**5**	
	***Desmostachya***	**1**				**1**	**1**	**1**	**1**			**1**	**1**		**1**	**1**				**1**	**1**	
	***Dignathia***											**1**									**1**	
	***Dinebra***	**1**				**1**	**1**	**1**	**1**	**1**		**1**	**1**		**2**						**1**	
	***Diplachne***					**1**		**1**	**1**	**1**			**1**	**1**	**1**					**1**	**1**	
	***Disakisperma***											**1**			**2**						**2**	
	***Eleusine***	**2**	**2**	**1**	**1**	**1**		**1**	**1**			**3**	**2**		**4**	**1**		**1**	**1**		**4**	
	***Enneapogon***	**1**	**1**	**1**		**2**	**1**	**3**	**3**			**4**	**3**		**5**				**1**	**2**	**6**	
	***Enteropogon***											**1**	**1**		**2**						**2**	
	***Eragrostis***	**1**	**5**	**5**		**8**	**7**	**11**	**10**	**2**	**3**	**11**	**15**	**3**	**17**	**3**	**4**	**6**	**4**	**6**	**19**	
	***Eustachys***											**1**									**1**	
	***Fingerhuthia***	**1**										**1**	**1**		**1**						**1**	
	***Halopyrum***				**1**	**1**				**1**		**1**	**1**		**1**				**1**	**1**	
	***Harpachne***														**1**						**1**	
	***Leptocarydion***																				**1**	
	***Leptochloa***	**1**						**2**	**2**													
	***Leptothrium***											**1**	**1**		**1**						**1**	
	***Lepturus***																				**4**	
	***Melanocenchris***					**1**						**1**	**2**		**1**						**2**	
	***Microchloa***											**1**			**1**						**1**	
	***Muhlenbergia***	**3**											**3**									
	***Neyraudia***	**1**											**1**									
	***Orinus***												**1**									
	***Oropetium***											**1**			**3**						**2**	
	***Schmidtia***																				**1**	
	***Schoenefeldia***														**1**						**1**	
	***Sporobolus***	**4**	**4**	**3**	**2**	**5**	**4**	**9**	**9**	**1**	**5**	**16**	**8**	**2**	**14**	**2**	**5**	**5**	**3**	**2**	**23**	
	***Tetrachaete***											**1**									**1**	
	***Tetrapogon***	**1**			**1**	**1**		**1**	**1**		**1**	**2**	**2**		**3**	**1**	**1**		**1**	**1**	**4**	
	***Tragus***	**1**	**1**	**1**		**2**					**1**	**2**	**3**		**2**		**1**	**1**	**1**	**2**	**2**	
	***Trichoneura***											**1**			**1**						**1**	
	***Trigonochloa***																				**2**	
	***Tripogon***	**1**										**6**	**2**		**4**						**6**	
	***Triraphis***											**1**			**1**						**1**	
	***Urochondra***											**1**	**1**		**1**						**1**	
	***Zaqiqah***														**1**						**1**	
***11) Arundinellae***	***Arundinella***											**2**	**1**									
	***Garnotia***											**1**										
***12) Tristachyideae***	***Danthoniopsis***					**1**							**1**		**1**						**1**	
	***Loudetia***											**1**										
***13) Digitaria***	***Anthephora***					**1**		**1**	**1**						**2**	**1**					**2**	
	***Digitaria***	**5**	**2**	**4**	**1**	**6**	**1**	**2**	**2**	**1**	**1**	**6**	**13**		**6**	**1**	**1**	**2**	**2**	**2**	**8**	
***14) Echinochloa***	***Echinochloa***	**4**	**1**	**2**	**1**	**3**	**2**	**2**	**2**	**1**	**2**	**2**	**5**	**1**	**4**	**2**	**2**	**3**	**2**	**2**	**2**	
***15) Panicum/Cenchrus/Urochloa/Setaria***	***Cenchrus***	**5**	**1**	**1**	**5**	**9**	**3**	**9**	**9**	**4**	**4**	**8**	**10**	**4**	**19**	**6**	**4**	**1**	**1**	**5**	**18**	
	***Eriochloa***		**1**	**1**		**1**	**1**					**1**	**2**		**1**				**1**		**2**	
	***Melinis***									**1**		**1**	**1**		**1**						**2**	
	***Moorochloa***	**1**	**1**	**1**			**1**	**1**	**1**			**1**	**1**		**1**			**1**	**1**	**1**	**1**	
	***Panicum***	**3**	**2**	**1**	**1**	**6**	**3**	**6**	**6**	**2**	**1**	**5**	**10**	**1**	**6**	**2**	**1**	**4**	**2**	**3**	**8**	
	***Setaria***	**3**	**2**	**2**	**2**	**3**	**3**	**5**	**5**	**3**	**4**	**6**	**7**	**2**	**7**	**2**	**4**	**3**	**2**	**2**	**10**	
	***Tricholaena***	**1**			**1**	**1**		**1**	**1**		**1**	**1**	**1**		**1**		**1**			**1**	**2**	
	***Urochloa***	**3**			**1**	**2**		**4**	**4**		**1**	**7**	**7**		**10**		**1**			**3**	**15**	
***16) Paspalum***	***Paspalum***	**1**	**1**	**1**	**1**	**1**	**1**	**1**	**1**		**1**	**1**	**3**		**3**	**2**	**1**	**3**	**1**	**2**	**3**	
***17) Stipagrostis***	***Stipagrostis***	**9**	**1**	**1**	**2**	**13**	**5**	**9**	**9**	**4**	**2**	**11**	**7**	**4**	**12**	**10**	**2**	**1**	**4**	**8**	**13**	
**Eudicots**																					
**ZYGOPHYLLALES**																					
**Zygophyllaceae**																					
***18) Tribulus/Kallstroemia***	***Tribulus***	**3**	**1**	**1**	**1**	**3**	**3**	**3**	**3**	**1**	**1**	**3**	**3**	**3**	**3**	**2**	**1**	**1**	**2**	**2**	**2**	
***19) Tetraena***	***Tetraena***				**1**	**1**		**1**	**1**			**1**	**1**		**1**					**1**	**1**	
**MALPIGHIALES**																					
**Euphorbiaceae**																					
***20) Euphorbia* subgen. *Chamaesyce***	***Euphorbia***	**6**	**5**	**7**	**1**	**9**	**7**	**9**	**9**	**3**	**6**	**6**	**12**	**1**	**11**	**3**	**5**	**8**	**2**	**3**	**10**	
**BRASSICALES**																					
**Cleomaceae**																					
***21) Cleome angustifolia***	***Cleome***																				**1**	
***22) Cleome gynandra***								**1**	**1**			**1**	**1**		**1**					**1**	**1**	
**CARYOPHYLLALES**																					
**Polygonaceae**																					
***23) Calligonum***	***Calligonum***	**5**	**1**	**3**	**1**	**17**	**3**	**2**	**1**	**1**	**1**	**3**	**2**	**1**	**3**	**1**	**1**	**2**	**15**	**2**	**2**	
**Caryophyllaceae**																					
***24) C4 Polycarpea***	***Polycarpaea***												**1**		**1**						**1**	
**Amaranthaceae**																					
***25) C4 Aerva***	***Aerva***	**1**			**1**	**1**	**1**	**1**	**1**	**1**		**1**	**1**	**1**	**1**	**1**				**1**	**1**	
***26) C4 Alternanthera***	***Alternanthera***							**1**	**1**				**2**		**1**						**1**	
***27) Amaranthus***	***Amaranthus***	**7**	**4**	**8**	**2**	**11**	**10**	**16**	**15**	**2**	**11**	**5**	**12**	**3**	**9**	**12**	**11**	**15**	**8**	**4**	**8**	
***28) Gomphreneae***	***Gomphrena***						**1**						**2**		**1**						**2**	
**Chenopodiaceae**																					
***29) C4 Atriplex***	***Atriplex***	**10**	**3**	**3**	**1**	**11**	**2**	**6**	**5**	**3**	**4**	**2**	**9**	**1**	**6**	**6**	**5**	**7**	**6**	**2**	**4**	
***30) Camphorosmeae***	***Bassia***	**9**	**5**	**5**	**1**	**8**	**1**	**5**	**4**	**3**	**1**	**1**	**8**	**1**	**7**	**3**	**3**	**3**	**6**	**1**	**1**	
***31) Bassia***	***Camphorosma***	**1**	**1**	**1**		**1**							**1**					**1**	**1**			
***32) Tecticornia***	***Tecticornia***												**1**									
***33) Caroxyleae***	***Caroxylon***	**13**	**6**	**7**	**2**	**20**	**9**	**6**	**6**	**3**		**4**	**9**	**3**	**12**	**3**	**3**	**10**	**9**	**3**	**4**	
	***Cimacoptera***	**8**	**1**	**1**		**9**	**3**	**1**					**2**				**2**	**2**	**9**			
	***Halarchon***	**1**																				
	***Halimocnemis***	**5**	**4**	**5**		**12**	**2**	**1**	**1**				**2**					**3**	**12**			
	***Halocharis***	**6**				**3**	**2**						**4**		**1**		**1**		**3**			
	***Kaviria***	**4**	**2**	**3**		**7**	**3**					**1**	**2**						**3**	**1**	**1**	
	***Petrosimonia***	**1**	**2**	**3**		**3**												**3**	**2**			
	***Piptoptera***	**1**				**1**													**1**			
	***Pyankovia***	**1**				**1**													**1**			
***34) Nanophyton***	***Nanophyton***																		**1**			
***35) C4 Salsoleae***	***Anabasis***	**5**	**2**	**4**	**2**	**12**	**3**	**5**	**4**	**1**	**1**	**1**	**3**	**1**	**4**	**2**	**1**	**1**	**9**	**1**	**1**	
	***Arthrophytum***	**1**																	**1**			
	***Cornulaca***	**2**			**1**	**2**	**3**	**1**	**1**	**2**	**1**	**2**	**2**	**2**	**4**	**1**	**2**	**1**	**1**	**1**	**2**	
	***Girgensohnia***	**4**	**1**	**1**		**4**	**1**	**1**					**1**					**1**	**2**			
	***Halogeton***	**2**					**1**	**1**	**1**	**1**	**1**		**3**		**1**	**1**	**1**		**1**			
	***Halothamnus***	**7**	**1**	**1**		**9**	**3**	**2**	**2**	**1**	**1**	**1**	**3**		**3**		**3**	**1**	**5**	**1**	**1**	
	***Haloxylon***	**2**				**2**	**1**	**1**	**1**			**1**	**1**		**1**	**1**			**2**	**1**		
	***Hammada***	**4**			**1**	**1**	**3**	**5**	**5**	**1**	**2**	**1**	**4**	**1**	**1**	**4**	**2**		**2**	**1**		
	***Horaninovia***	**2**				**4**													**3**			
	***Iljinia***																		**1**			
	***Lagenantha***																				**1**	
	***Noaea***	**2**	**2**	**1**		**1**	**1**	**1**	**1**				**1**		**1**			**2**	**1**			
	***Soda***	**2**	**2**	**3**	**1**	**8**	**2**	**5**	**4**		**3**	**5**	**1**	**3**	**3**	**3**	**4**	**3**	**1**	**1**	
	***Sevada***											**1**			**1**						**1**	
	***Traganum***						**1**	**1**	**1**	**1**	**1**			**1**	**1**	**1**	**1**					
	***Turania***	**1**				**1**													**5**			
	***Xylosalsola***	**3**				**2**							**2**						**5**			
***36) Salsola***	***Salsola***	**6**	**2**	**5**		**6**	**1**	**1**					**4**		**1**			**3**	**3**		**1**	
***37) Bienertia***	***Bienertia***	**1**	**1**	**1**		**3**	**1**			**1**			**1**	**1**	**1**			**1**	**1**	**1**		
***38) Suaeda sect. Salsina***	***Suaeda***	**6**	**3**	**3**	**2**	**8**	**5**	**6**	**6**	**2**	**3**	**4**	**5**	**2**	**4**	**4**	**4**	**2**	**4**	**2**	**4**	
***39) Suaeda sect. Schoberia***		**1**	**1**	**1**		**5**	**1**	**1**	**1**		**1**		**2**			**1**	**1**	**6**	**2**			
**Gisekiaceae**																					
***40) C4 Gisekia***	***Gisekia***	**1**				**1**		**1**	**1**			**1**	**1**		**1**					**1**	**1**	
**Aizoaceae**																					
***41) Sesuvium/Trianthema/Zaleya***	***Sesuvium***												**1**		**1**						**1**	
	***Trianthema***					**1**		**1**	**1**			**1**	**2**		**4**						**4**	
	***Zaleya***	**1**				**1**		**1**	**1**			**1**	**1**		**1**					**1**	**1**	
**Nyctaginaceae**																					
***42) Boerhavia***	***Boerhavia***					**1**		**1**	**1**			**1**	**4**		**2**	**1**				**1**	**2**	
**Molluginaceae**																					
***43) Hypertelis cerviana/H. fragilis***	***Hypertelis***					**1**							**1**		**1**			**1**			**1**	
**Portulacaceae**																					
***44) Portulacaceae***	***Portulaca***	**2**	**1**	**2**	**2**	**1**	**2**	**1**	**1**	**1**	**1**	**2**	**5**	**1**	**5**	**1**	**1**	**1**	**1**	**1**	**3**	
**BORAGINALES**																					
**Boraginaceae**																					
***45) Euploca***	***Euploca***					**1**						**1**	**2**		**2**					**1**	**4**	
**LAMIALES**																					
**Scrophulariaceae**																					
***46) Anticharis***	***Anticharis***					**1**		**1**	**1**				**2**		**1**	**1**				**1**	**2**	
**Acanthaceae**																					
***47) Blepharis***	***Blepharis***					**2**		**2**	**2**			**2**	**3**		**2**	**1**					**3**	
**ASTERALES**																					
**Asteraceae**																					
***48) Flaveria clade A***	***Flaveria***						**2**					**1**			**1**	**1**				**1**	**1**	

SW and Central Asia which represent largely the Irano-Turanian flora are the center of origin of at least 12 C_4_ Chenopodiaceae lineages (Akhani et al., 2007; Sage, 2011; Sage, 2016). We consider Salsoleae s. str. as one C_4_ origin with understanding that present topologies suggest two additional origins that are not well resolved (Akhani et al., 2007; Kadereit and Freitag, 2011).

The region shows also the highest diversity of species, anatomical and ecological types, and life forms in C_4_ Eudicots (see **Supplementary Appendix Table 1**). Of those lineages, 11 (*Suaeda* sect. *Salsina*, *Suaeda* sect. *Schoberia*, *Bienertia, Camphorosma, Bassia*, C_4_ Salsoleae s. str., Caroxyleae, *Salsola* (=*Kali*), *Nanophyton*, C_4_ *Atriplex*, and C_4_ *Tecticornia*) are distributed throughout SW Asia. The origin of the strictly psammophytic genus *Calligonum* (Polygonaceae) has been proposed to be in northern Iran (Irano-Turanian region), on the former shores of the Tethys sea, from where it spread to Central, South, and Southwest Asia and northern Africa (Soskov, 2011). On the other hand, C_4_ Aizoaceae, C_4_ Zygophyllaceae (*Tetraena simplex* and *Tribulus*/*Kallstroemia* lineages), the *Cleome angustifolia*, and C_4_ *Aerva* lineages and probably C_4_ *Gisekia* and C_4_ *Polycarpaea* have originated on the Arabian Peninsula and adjacent Africa (Sage, 2011).

### C_4_ Dominated Vegetation in Southwest Asia

**Figure 8:**
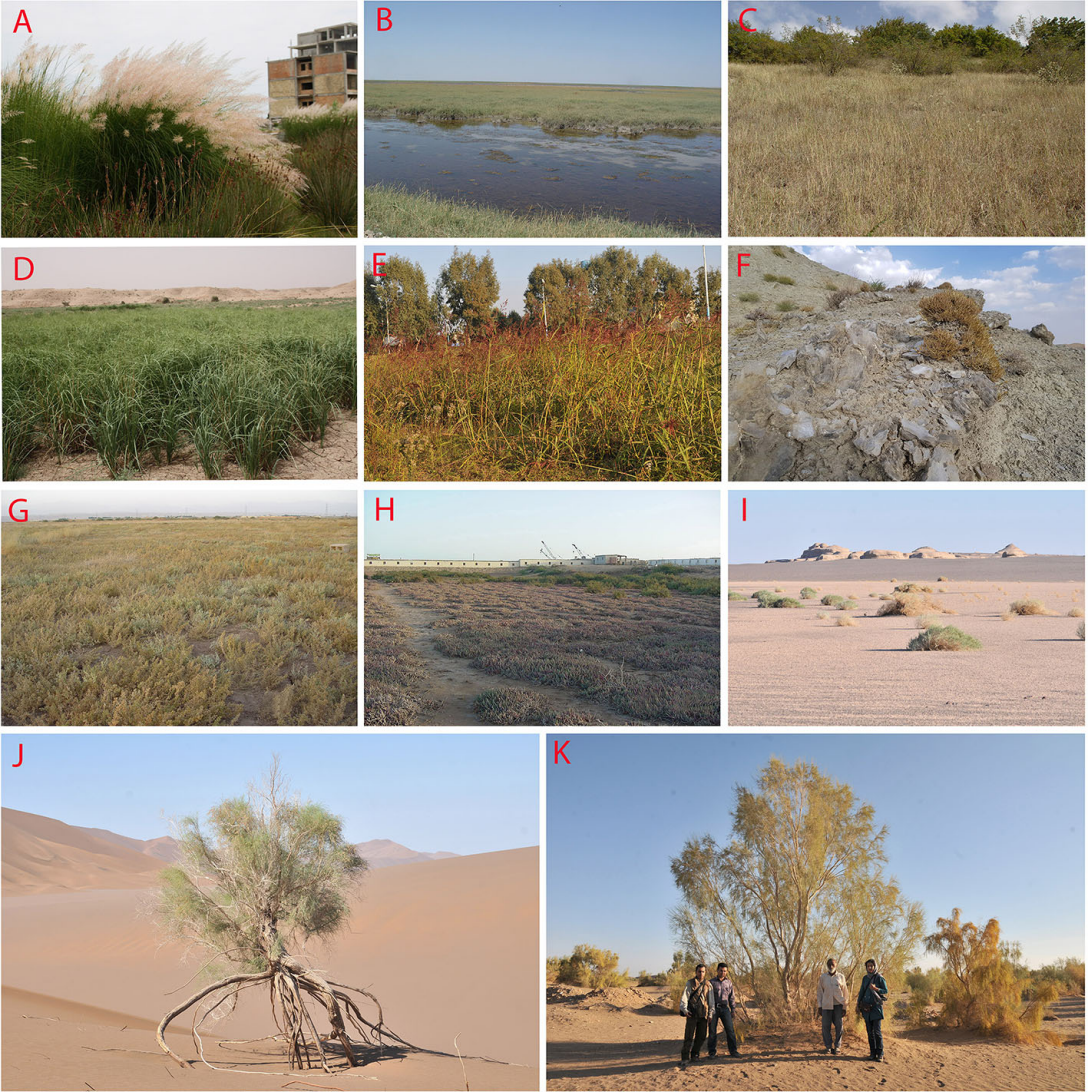
**(A–K)** Habitats dominated by C_4_ vegetation: **(A)** habitat with high water table in Northeast (NE) Iran, near Caspian Sea, dominated by *Saccharum spontaneum L.* **(B)** euhalophytic vegetation of *Aeluropus litoralis* (Gouan) Parl. community, close to the SE Caspian shores, Turkman Sahra salt flats, Iran; **(C)** C_4_ grassland in a temperate forest on rocky S-facing slopes of Golestan National Park, Iran; **(D)** *Desmostachya bipinnata* on temporarily flooded dry soils in Khuzestan, Southwest (SW) Iran; **(E)** ruderal vegetation dominated by *Sorghum halepense* (L.) Pers., SE Caspian coasts, Farahabad, Mazandaran, Iran; **(F)** gyspsiferous outcrop of *Anabasis eugeniae* Iljin and *Anabasis calcarea* (Charif & Aellen) Bokhari & Wendelbo community, NW Iran; **(G)** euhalophytic community in central Iran, 60 km W Tehran mainly dominated by *Bienertia cycloptera* Bunge ex Boiss. and *Climacoptera turcomanica* (Litv.) Botsch. **(H)** shores of the Makran coast (Pakistan) dominated by *Tecticornia* indica (Willd.) K. A. Sheph. & Paul G. Wilson, **(I)** *Hammada salicornica* (Moq.) Iljin community in Desert Lut, S. Iran; **(J)** *Calligonum amoenum* Rech. f. on moving dunes in Lut desert; **(K)** *Haloxylon persicum* Bunge shrubland near Mesr Village, c. 40 km E of Jandagh, Dasht-e-Kavir, Iran. All photos H. Akhani.

**Figure 9:**
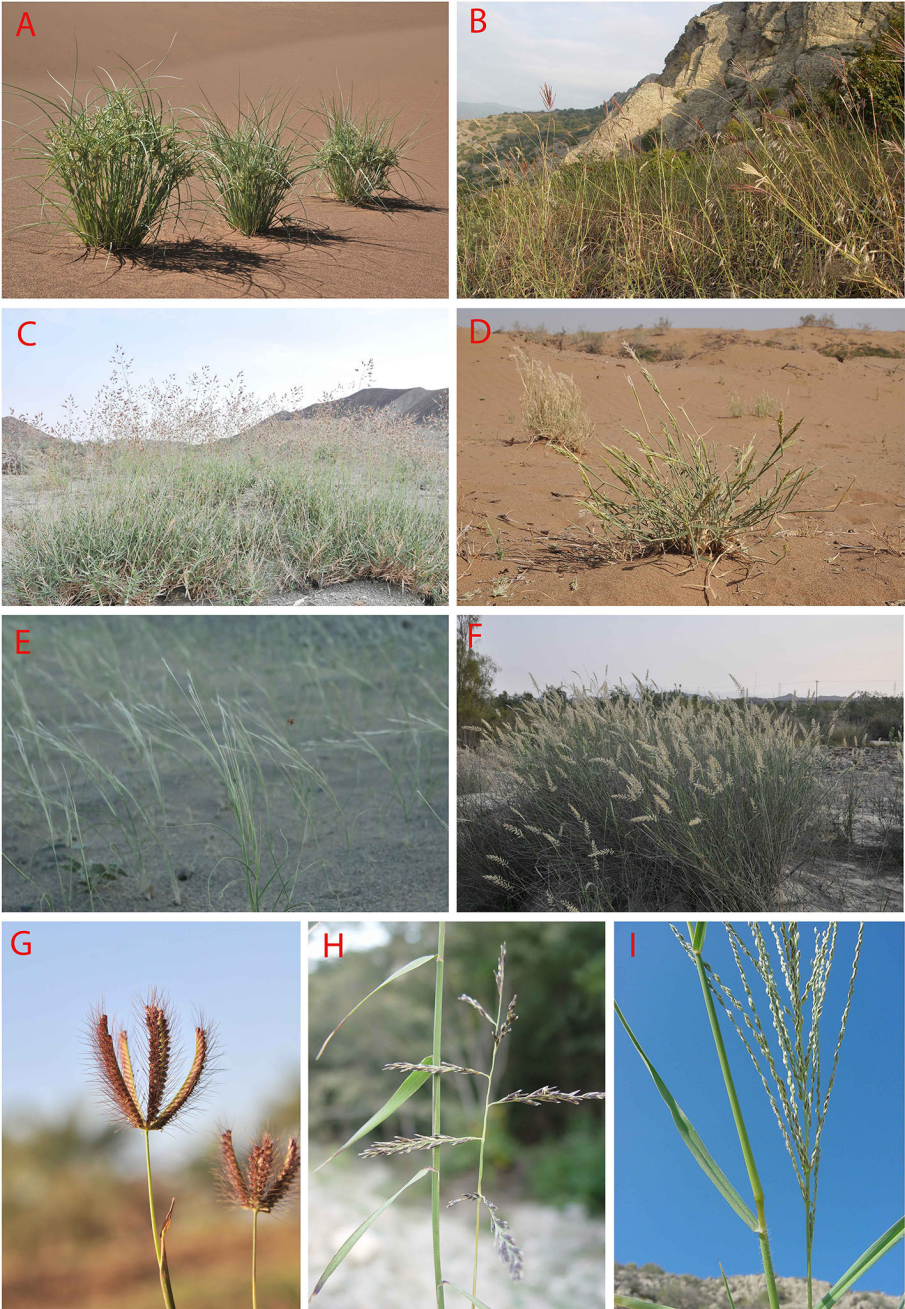
**(A–I)**: Some representatives of important C_4_ Monocot lineages in Southwest (SW) Asia: **(A)** *Cyperus aucheri* Jaub. and Spach, sand dune in Desert Lut, Iran; **(B)** *Bothriochloa ischaemum* (L.) Keng-*B. bladhii* (Retz.) S.T. Blake*-Cleistogenes serotina* (L.) Keng community, S-facing rocky outcrop in Golestan National Park, Iran; **(C)** *Danthoniopsis stocksii* (Boiss.) C.E. Hubb., dry river bed, Baluchistan, Iran; **(D)** *Centropodia forskalii* (Vahl) Cope, Aran-Bidgol dunes in Esfahan Province, Iran, 6.6.2010; **(E)** *Stipagrostis multinervis* H. Scholz, Desert Lut, Iran, 1.4.2011; **(F)** *Cenchrus divisus* (J.F. Gmel.) Verloove, 19.2.2013, river side in Bahukalat, Baluchestan, Iran; **(G)** *Chloris barbata* Sw., 17.2.2013, ruderal places, in Zehkalut, Kerman, Iran; **(H)** *Cleistogenes serorina* (L.) Keng, 13.10.2003, limestone rocky outcrops in Golestan National Park, Iran; **(I)** *Digitaria nodosa* Parl., 18.12.2001, rocky shrubland, Kuhe Geno, Hormozgan, Iran (photos by H. Akhani).

**Figure 10:**
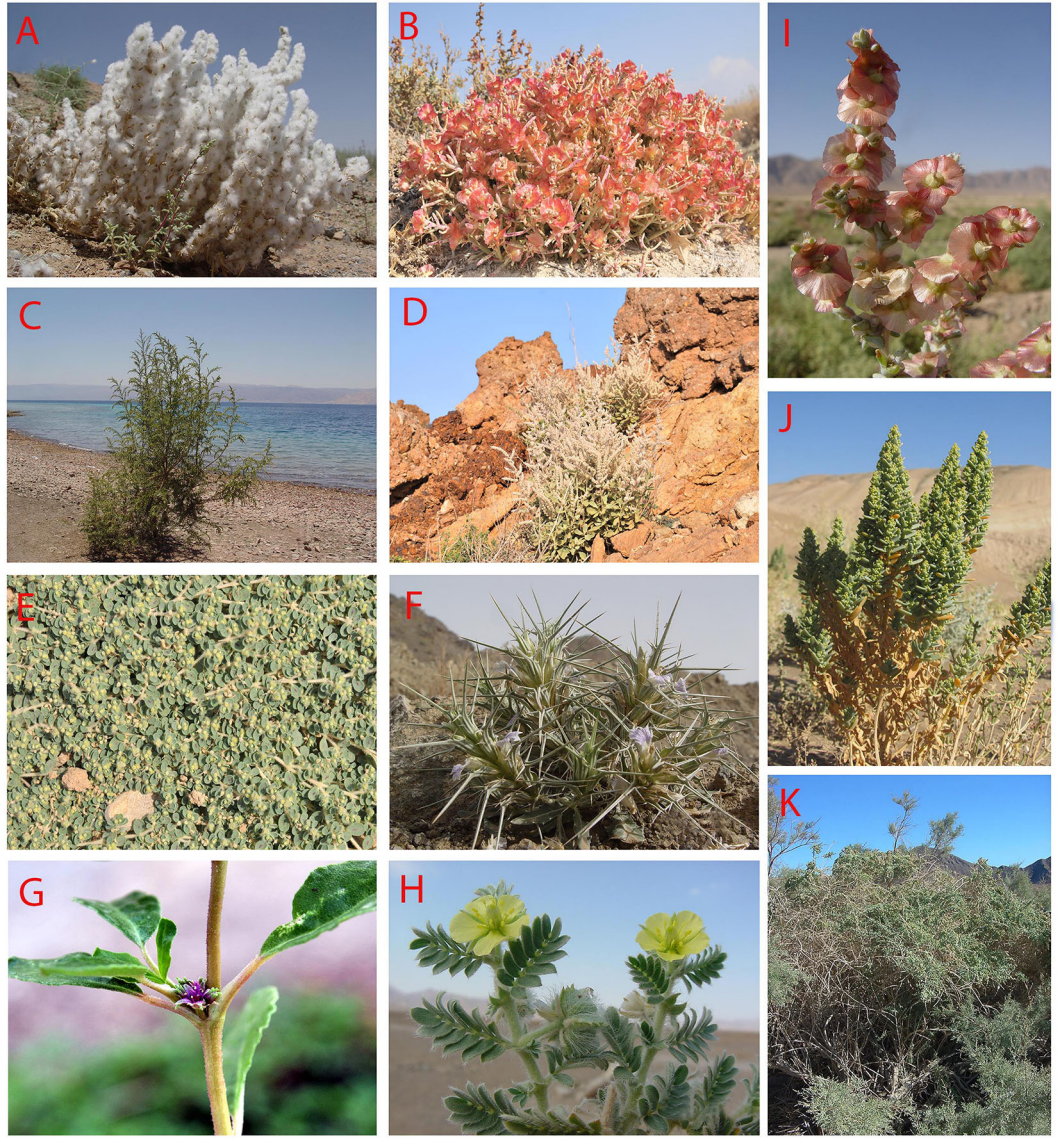
**(A–K)**: Some representatives of important C_4_ Eudicot lineages in Southwest (SW) Asia: **(A)** *Bassia eriantha* (Fisch. and C.A. Mey.) Kuntze, 10.6.2004, *Artemisia*+*Stipagrostis* semi-desert steppe, 50 km SE Esfahan, Iran; **(B)** *Anabasis firouzii* Akhani, 10.10.2012, marl slopes, Qorkhod Protected Area, N. Khorassan, Iran; **(C)** *Suaeda monoica* Forssk. ex J.F. Gmel. 18.6.2007, Red Sea coasts, Aqabeh, Jordan; **(D)** *Aerva javanica* (Burm.f.) Juss. ex Schult. 22.2.2013, rocky slopes, Hormoz Island, Persian Gulf; **(E)** *Euphorbia serpens* Kunth, 3.7.2014, ruderal soil, Khuzestan, Iran; **(F)** *Blepharis ciliaris* (L.) B.L. Burtt, 22.2.2013, gravely-sandy soils, Hormoz Island, Persian Gulf; **(G)** *Trianthema portulacastrum* L., 23.2.2013, ruderal places, Hormoz Island, Persian Gulf; **(H)** *Tribulus macropterus* Boiss. 18.9.2001, Khuzestan, Iran; **(I)** *Climacoptera chorassanica* Pratov, 1.9.2003, hypersaline soils, 40 km SE Birjand, Khorassan, Iran; **(J)** *Bienertia kavirense* Akhani, 5.8.2016, Esfahan/Semnan, Iran; **(K)** *Caroxylon abarghuense* (Assadi) Akhani and Roalson, 5.10.2001, Touran protected Area, Iran (photos by H. Akhani).

C_4_ dominated plant communities in SW Asia occur in a wide range of habitats:

1. *Psammophytic C_4_ vegetation* occurs in inland sandy deserts and coastal dunes in the following types of vegetation: a) The large sand deserts of the Irano-Turanian and Saharo-Sindian vegetation in Iran, Turkmenistan, Afghanistan, Iraq, and the Arabian Peninsula. The Irano-Turanian sandy deserts are mainly dominated by pure or mixed communities of highly specialized psammophytic C_4_ Eudicots [(*Calligonum* spp. (**Figures 8J**, **9E**)], *Haloxylon* spp.*, Xylosalsola* spp.)] and Monocots (*Stipagrostis* spp.) (Nechayeva, 1992; Ghasemkhani et al., 2008; Fayvush and Aleksanyan, 2016). In the deserts of Turkmenistan and Central Iran *Haloxylon* communities may be rather densely populated, forming “Saxaul forests” (**Figure 8K**). From central Iran toward the Levant and the Rub Al-Khali the dominating C_4_ Eudicots change [(*Calligonum* spp.*, Hammada salicornica* (**Figure 8I**)*, Cornulaca* spp.*, Anabasis articulata*)] while the diversity of psammophytic C_4_ Monocot communities increases [*Cyperus* spp. (**Figure 9A**)*, Stipagrostis* spp. (**Figure 9E**)*, Aristida* spp.*, Centropodia* spp. (**Figure 9D**)*, Panicum* spp.] (Ghazanfar and Fisher, 1998), similarly the sand dune vegetation in Lut desert in SE Iran and the deserts of South-East Pakistan are dominated by communities of *Calligonum polygonoides, Calligonum mongolicum*, *Soda stocksii* (*Salsola stocksii*), *Tribulus* spp. (**Figure 10H**), *Aerva javanica, Lasiurus scindicus* and *Cymbopogon jwarancusa*, *Stipagrostis multinerva, Desmostachya bipinnata* (Dasti and Agnew, 1994). b) Hamadas, gravel, and coarse sand deserts show rather sparse vegetation, sometimes dominated by sparse communities of chenopods like *Hammada salicornica* (**Figure 8I**), *Cornulaca monacantha*, and *Anabasis* spp. in the tropical deserts of the South SW Asia (Ghazanfar and Fisher, 1998; Akhani, 2015a). However, the temperate deserts of Iran are covered by dense or sparse grasslands of *Stipagrostis plumosa*, often mixed with *Artemisia* subshrubs. c) Coastal dune vegetation varies within the region. E.g., the sandy dunes of the Indus delta and Pakistani Balochistan present mixed psammophytic communities with frequent presence of *Aerva javanica* (**Figure 10D**)*, Cyperus arenarius*, *Soda stocksii* (*Salsola stocksii*)*, Hammada salicornica*, and *Cenchrus biflorus* (Snead and Tasnif, 1966; Ananda Rao and Meher-Homji, 1985). The coastal dunes of the Arabian Peninsula are dominated by communities of halophytic sand grasses and sedges like *Zaqiqah mucronata, Urochondra setulosa, Sporobolus* spp.*, Dactyloctenium* spp.*, Leptothrium senegalense, Halopyrum mucronatum, Cenchrus divisus, Panicum turgidum, Lasiurus scindicus, Coelachyrum piercei*, and *Cyperus* spp. as well as Chenopods like *Cornulaca monacantha and Atriplex stocksii* (Ghazanfar and Fisher, 1998; Brown and Mies, 2012).
2. *Halophytic C_4_ vegetation* is highly diverse and affected by topography, water level, and local land use. Saline habitats range from marly and clayey hills with varying salt and gypsum composition to inland dry saline plains with clay, silt, and sandy soils in sabkhas, saline wetlands, and lakes, as well as coastal sabkhas and shorelines. They composed a wide range of communities from pure C_4_ communities to C_4_ patches occurring in microhabitats of C_3_ dominated communities:
  - Dry marly or clayey hills with varying salt and gypsum composition are often dominated by xerohalophytic and gypsophytic C_4_ Chenopods. In Iran such habitats are sparsely vegetated by xerohalophytic and gypsohalophytic shrubs like *Anabasis eugeniae* (**Figure 8F**)*, Anabasis calcarea, Anabasis firouzii* (**Figure 10B**)*, Halothamnus auriculus, Halothamnus lancifolius, Xylosalsola arbuscula, Noaea mucronata, Caroxylon verrucosum, Suaeda dendroides*, *Kaviria tomentosa, K. aucheri,* and *K. zedzadii* (Akhani, 2006; Pérez-García et al., 2018). In Israel similar saline chalk and marl slopes are dominated by communities of *Suaeda asphaltica, Hammada negevensis*, *and Caroxylon tetrandrum* (Danin and Orshan, 1999).
  - Saline plains, salt marshes, and depressions with varying water-table offer a habitat to pure or mixed C_4_ Chenopod communities, that are typical for the Irano-Turanian and Saharo-Sindian regions. Such communities are divided into two main subgroups namely “the C_4_ dominated shrubby communities (vegetation class Haloxylo-Kavirietea tomentosae)” and “the C_4_ rich Irano-Turanian nitrophilous annual halophytic communities (vegetation class Caroxylo-Climacopteretea).” The C_4_ shrubby dominated communities are composed of *Haloxylon ammodendron*, *Kaviria tomentosa*, *Hammada* spp., *Halothamnus subaphyllus*, *H. glaucus*, *Soda rosmarinus* (*Seidlitzia rosmarinus*), *Cornulaca monacantha*, *Anabasis aphylla, A. haussknechtii, A. iranica* spp., and *Suaeda fruticosa* (Akhani, 2004). The C_4_ rich Irano-Turanian nitrophilous annual halophytic communities composed of a rich variety of annual chenopods like *Climacoptera* spp. (**Figures 8G** and **10I**), annual *Caroxylon* spp.*, Halimocnemis* spp.*, Petrosimonia* spp.*, Cornulaca aucheri, *annual *Atriplex* spp.*, Halocharis* spp.*, Pyankovia brachiata, Bienertia* spp. (**Figures 8G** and **10J**), annual *Suaeda* spp. *(S. cochlearifolia, S. gracilis, S. microsperma, S. khalijefarsica, S. arcuata, S. aegyptiaca, S. altissima), Bassia* spp. (*B. hyssopifolia, B. erinatha, B. eriophora*) (**Figure 10A**), and *Soda florida (Seidlitzia florida*). (Ghazanfar and Fisher, 1998; Akhani, 2004; Akhani, 2006). This vegetation type develops on disturbed nitrified soils on salinized wastelands, ruderal habitats around the roads, and human settlements and as pioneer communities on exposed high saline soils of dried up salt lakes (Ghorbanalizadeh et al., 2020).
  - The vegetation of coastal saline flats resembles their inland counterparts. Such vegetation is present on the SE shores of the Caspian sea, along the coasts of the Persian Gulf, the Indian Ocean, and in lesser extent along the Red Sea. E.g., the coastal sabkha vegetation of the Persian Gulf is dominated by *Suaeda fruticosa, Caroxylon imbricatum, Atriplex leucoclada, Climacoptera* sp., *Soda rosmarinus* (*Salsola rosmarinus*), and *Bienertia sinuspersici*, while the Red Sea and Indian Ocean coastal flats harbor communities of *Suaeda monoica* (**Figure 10C**)*, S. moschata, S. vermiculata/S. fruticosa, Atriplex leucoclada*, *and A. coriacea* (Ghazanfar and Fisher, 1998; Akhani and Deil, 2012; Akhani, 2015a). Similar communities occur along the Makran coast and in the saline flats of the Indus delta, these communities also include *Suaeda baluchestanica*, *Halopyrum mucronatum*, and the remarkable tropical *Tecticornia indica* (**Figure 8H**) (Snead and Tasnif, 1966; Voznesenskaya et al., 2008).
  - A remarkable type of C_4_ halophytic vegetation are C_4_-grass communities present on saline clayey soils with high water-table, formed by halophytic grasses of the genus *Aeluropus* (**Figure 8B**). Such communities occur e.g., on the eastern shores of the Caspian Sea, where *Aeluropus littoralis* forms almost pure communities on flooded plains in Iran and Turkmenistan (Nechayeva, 1992; Rukhlenko, 2001). Similar pure stands and mixed communities of *Aeluropus lagopoides* with *Tamarix* spp., *Desmostachya bipinnata*, and *Salvadora oleoides* have been reported from inland Sabkhas and salt pans of Iran, Syria, the Arabian Peninsula, and Pakistan (Dasti and Agnew, 1994; Ghazanfar and Fisher, 1998; Akhani, 2006; Al-Oudat and Qadir, 2011). Finally, *Aeluropus* spp. pure and mixed communities (in association with *Suaeda monoica, Fimbristylis* spp., etc.) as well as communities of other halophytic grasses, like *Sporobolus* spp. and *Halopyrum mucronatum* are also frequent on the shores of the Indian Ocean and the Persian Gulf (Snead and Tasnif, 1966; Ananda Rao and Meher-Homji, 1985; Ghazanfar and Fisher, 1998; Akhani, 2015a).
3. *C_4_ grassland vegetation* is limited by high temperatures and the availability of water in the form of summer precipitation or high underground water table (Ghazanfar and Fisher, 1998). The following types of C_4_ grassland communities have been so far described:
  - Oreophytic grasslands on the rocky highlands of S Arabia and Socotra may be distinguished by pure C_4_ or mixed C_3_ and C_4_ grassland communities with the presence of local afromontane endemics. These are mainly secondary grasslands resulting from deforestation of local shrublands (Ghazanfar and Fisher, 1998). However, some examples of high-altitude primary grasslands exist in suitable habitats. Examples are high-altitude primary low growing mixed grasslands (up to 3,000 m) of the Arabian endemics *Festuca cryptantha* (C_3_), *Andropogon crossotos* (C_4_), *Tripogon oliganthos* (C_4_), and cold resistant *Stipa* spp. (C_3_); grasslands dominated by *Andropogon bentii* on Socotra (600 m) and associations of the C_4_ grass *Elionurus muticus* with the dwarf shrub *Macowania ericifolia* (Elionuro mutici-Macowanietum ericifoliae) on the mountainous basalt flats in Central Yemen (Deil and Müller-Hohenstein, 1985; Ghazanfar and Fisher, 1998; Brown and Mies, 2012). Examples of tall secondary grasslands in S Arabia are high altitude communities (2,000–3,000 m) dominated by *Themeda triandra, Heteropogon contortus, Andropogon distachyos, Bothriochloa insculpta, Cenchrus unisetus*, *Hyparrhenia hirta*, *Arthraxon lancifolius *and* A. hispidus* (Themedo-Hyparrhenietea); dry grasslands dominated by *Tetrapogon villosus* (C_4_), *Elionurus muticus* (C_4_), *Stipa tigrensis* (C_3_), and *Cenchrus setaceus* (C_4_) and tall monsoon facing grasslands (above 750 m) dominated by *Themeda quadrivalvis*, *Apluda mutica, Setaria pumila*, *Heteropogon contortus*, *Sporobolus spicatus*, and *Arthraxon junnarensis* (Ghazanfar and Fisher, 1998; Brown and Mies, 2012).
  - In appearance similar C_4_-grass dominated communities exist on exposed S-facing slopes of Caspian forests (Akhani and Ziegler, 2002). A combination of edaphic and climatic factors, e.g., presence of summer rainfall in one hand and edaphic constraint as a result of rocky substrate with usually poor soil layer in another hand are advantageous for the formation of a C_4_ grasslands in steep S-facing slopes, dominated by C_4_ grasses like *Bothriochloa ischaemum* (**Figure 9B**)*, B. bladhii, Cleistogenes serotina* (**Figure 9H**)*, Heteropogon contortus, and Cenchrus orientalis* (**Figure 8C**). The arboreal vegetation is made by N temperate elements such as *Carpinus orientalis, Zelkova carpinifolia, Quercus castaneifolia, Colutea buhsei, Crataegus* spp.*, Cotoneaster* spp., and *Rosa* spp. A comparable vegetation forms in olive orchards in Caspian lowlands as a result of artificial irrigation during summer time have been observed (Akhani, unpublished). The South Caspian C_4_ grasslands are paralogues to the Mediterranean C_4_ dominated grassland of the order Cymbopogono-Brachypodietalia ramosi (=Hyparrhenietalis hirtae) (Diez-Garretas and Asensi, 1999; Mucina et al., 2016). In the western Himalayas (E Afghanistan, N Pakistan), where monsoon provides sufficient rainfall, similar grasslands with *Cymbopogon, Chrysopogon, Heteropogon, Aristida*, etc. grow in association with *Acacia modesta* and *Olea cuspidata* woodlands. *Chrysopogon-Cymbopogon* grasslands are also typical for the uplands of Pakistani Balochistan (Suttie and Reynolds, 2003).
  - Xeromorphic grasslands occur mainly in lowlands of the Arabian Peninsula and in southern Iran. They occur rarely as separate communities but rather with open *Acacia* pseudo-savannas or mixed with shrubs like *Calligonum* sp., *Leptadenia pyrotechnica*, or *Lycium shawii* or with xeromorphic dwarf shrubs like *Hammada salicornica* and *Caroxylon cyclophyllum*. The dominating species is the C_4_ grass *Panicum turgidum*, which mostly forms pure grass communities or co-occurs with other C_4_ grasses, like *Lasiurus scindicus, Aristida mutabilis, Dichanthium* spp.*, Cenchrus* spp. (**Figure 9F**), *Stipagrostis* spp., and *Aristida* spp. (Kürschner, 1986; Ghazanfar and Fisher, 1998).
4. *Meso- and hygrophytic communities* are known on river shores, irrigation channels, seasonal rivers, and wadis of Turkmenistan, Afghanistan, Pakistan, and Iran, as well as on non or slightly salty sandy soils with high water-table, close to Caspian shores, with intensive human disturbance, with patches and communities of tall C_4_ grasses like *Imperata cylindrica*, *Saccharum spontaneum*, and *Tripidium ravennae*. *Saccharum spontaneum* (**Figure 8A**) communities often co-occur with *Tamarix* spp. shrubs or tall C_3_ grasses, like *Phragmites* spp. and *Arundo donax*, and in transition zones, with more constant water-table these patches form sometimes transitions with more hygrophytic C_4_ sedges, like *Cyperus* sp. or *Fimbristylis* sp. (Chaudhri et al., 1966; Nechayeva, 1992; Chandran, 2015). In Israel passes the northern distribution of another important C_4_ hygrophyte, *Cyperus papyrus*. Once forming communities together with C_3_ grasses like *Phragmites* and *Arundo*, as well as *Typha*, its communities unfortunately largely disappeared because of habitat destruction and pollution (Danin and Orshan, 1999). In drier regions of S Iran and Arabia more xerophytic grasses, like *Desmostachya bipinnata* (**Figure 8D**)*, Cymbopogon* sp., etc. often replace this kind of vegetation in wadi beds and seasonally flooded plains (Kürschner, 1986; Ghazanfar and Fisher, 1998; Akhani, 2015a).
5. *Ruderal C_4_ communities* can be classified as follows: C_4_ ruderal and weedy communities in irrigated fields or adjacent disturbed places (**Figure 8E**): In most parts of the Irano-Turanian region and Caspian lowlands the weedy communities develop during summer times dominated by species of *Sorghum*, *Euphorbia* subgen. *Chamaesyce* (**Figure 10E**), *Bothriochola*, *Setaria*, *Eleusine*, *Echinochloa*, *Digitaria* (**Figure 9I**), *Paspalum*, *Cynodon*, *Imperata cylindrica*, *Cyperus* spp., *Atriplex* spp. (mostly *Atriplex tatarica*), *Portulaca oleracea*, *Bassia scoparia*, *Salsola tragus, Suaeda altissima*, *S. arcuata*, *Caroxylon* spp.*, Tribulus terrestris*, and several *Amaranthus* species. The species combination varies based on the irrigation regime, soil, and management of weed control. In southern parts of SW Asia the diversity of more thermophilous ruderal grasses like *Cenchrus, Panicum, Eragrostis, Dactyloctenium, Chloris* (**Figure 9G**)*, Sporobolus, Urochloa, Hyparrhenia hirta, Desmostachya bipinnata* (**Figure 8D**) as well as Eudicots like *Boerhavia repens*, *Euphorbia* subgen. *Chamaesyce, Zaleya pentandra, Trianthema portulacastrum *(**Figure 10G**)*, Aerva javanica, Suaeda aegyptiaca* increases (Nasir and Ali, 1970-2003; Shmida and Aronson, 1986; El-Ghanim et al., 2010; Abdel Khalik et al., 2013; El-Sheikh, 2013).

### C_4_ Eudicots Are Related to Dominance of Continentality Index

Our findings show different tendencies of C_4_ Monocot and C_4_ Eudicot distributions in the study area. The usual pattern of increase of C_4_ species along a latitudinal gradient is more or less similar to global patterns (Pyankov et al., 2010, **Figure 6**) with deviations mostly due to its complex topography, edaphic factors, and the resulting presence of specific microclimates. The C_4_ grasses increase along the southern and eastern edges of the region (Yemen, Oman, Pakistan) with higher summer precipitation due to monsoon and tropical climates (Ghazanfar and Fisher, 1998), **Figure 6D**. In fact, C_4_-grasslands are known to depend on a dry and rain seasonality, where bushfires during dry seasons on one hand prevent forest growth while high temperatures on the other hand favor C_4_ grasses (Skinner et al., 2002; Bond et al., 2005; Keeley and Rundel, 2005; Hoetzel et al., 2013). The savanna-like C_4_ grasslands at the south-eastern corner of the Caspian forests are supported by a small peak of summer precipitation, while the spring flora is dominated by C_3_ grasses and forbs (Akhani and Ziegler, 2002). The C_4_ poor but rainfall rich Euro-Siberian portions of SW Asia also favor C_4_ Monocots over C_4_ Eudicots. For example, in the north-western edges of the study area the percentage of Monocot species in the local C_4_ flora may reach 84% (Zaqatala, Republic of Azerbaijan) (‘GBC’).

The high proportion of C_4_ Eudicots in Iran and Turkmenistan may be explained by large saline and sandy deserts and high continentality, which favor halophytic Chenopodiaceae species and psammophytic *Calligonum* spp. The increase of C_4_ Eudicots in W-E direction even in similar longitudinal belts may be explained by a combination of edaphic and climatic conditions (compare the opposite tendency in China, Wang and Ma, 2016). Indeed, the continentality index clearly increases along a W-E direction over the SW Asia to Central Asia (Djamali et al., 2011; Djamali et al., 2012). The harsh summer times with scarcity of fresh water resources in deserts of Iran and Turkmenistan reduce the competitive advantages of C_3_ species (Pyankov et al., 2010; Sage and Sultmanis, 2016). Additionally, absence of summer rainfall supresses C_4_ grassland formation. Although higher continentality adversely affects general C_4_ domination (**Figure 6C**), this is not the case for C_4_ Chenopodiaceae, which are adapted to temperate deserts with continental climate (**Figure 6A**). This is explained by the phenology of chenopods with an estival active growing season (Toderich et al., 2007). Many chenopods and species of *Calligonum* are highly specialized by their morpho-anatomical and physiological traits to live under harsh conditions (e.g., their long root systems have access to the underground and subsurface water-table) (Gintzburger et al., 2003; Soskov, 2011). The negative correlation of C_4_ Cyperaceae with continentality (**Figure 6B**) and positive correlation with average annual temperature relate to their sensitivity to low winter temperatures (Wang and Ma, 2016). C_4_ Cyperaceae however do not seem to be unaffected by precipitation. The later may be explained by a high variety of ecotypes within C_4_ *Cyperus* (the main bulk of C_4_ Cyperaceae), ranging from psammophytic xerophytes to hygrophytes limited to permanent wetlands and the consequent species shift in relation to various ecological conditions.

Finally, it is interesting to note, that while different metabolic subtypes often indicate the adaptation of C_4_ Monocots to specific ecological conditions, e.g., NADP-ME Monocots are more likely distributed in areas with high rainfall, while NAD-ME Monocots grow in conditions with lower rainfall (Schulze et al., 1996), this feature doesn’t seem indicative in C_4_ Eudicots E.g., xerophytic C_4_ chenopods show both NAD-ME or NADP-ME metabolisms (see **Supplementary Appendix Table 1**).

### Southwest Asia—Center of Biodiversity of Single-Cell C_4_ and C_3_–C_4_ Switching Plants

One of the fascinating aspects of C_4_ photosynthesis is the discovery of Single-Cell functioning C_4_ photosynthesis in two C_4_ lineages of the Chenopodiaceae (Voznesenskaya et al., 2001; Edwards et al., 2004; Akhani et al., 2005). This photosynthetic type was described for the first time in *Suaeda aralocaspica* (Bunge) Freitag and Schütze (= *Borszczowia aralocaspica* Bunge), a hygrohalophyte from the saline depressions of Central Asian semideserts (Freitag and Stichler, 2000; Voznesenskaya et al., 2001). *Bienertia* as a monophyletic lineage in which all species perform single-cell functioning C_4_ was discovered shortly after *S. aralocaspica* with some new species (Voznesenskaya et al., 2002; Akhani et al., 2003; Akhani et al., 2005; Kapralov et al., 2006; Akhani et al., 2012). The genus *Bienertia* is diversified mostly in Iran and some surrounding areas often on moist and highly saline soils in association with several annual C_4_ chenopods belonging to Caroxylo-Climacopteretea class in the interior Iran or on open habitats of saline shrublands on the lowlands around the Persian Gulf between *Tamarix* species or on tidal shores (Akhani et al., 2003; Akhani et al., 2009).

Instead of a conventional system of C_4_ terrestrial species, having a dual-cell compartment consisting of mesophyll and bundle sheath cells, in both single-cell C_4_ lineages, this achieved by localizing photosynthetic machinery in a single-cell type. In *Suaeda aralocapica* dimorphic chloroplasts are polarized in a single layer mesophyll cell, in which the proximal chloroplasts fix CO_2_ using PEPC into a C_4_ acid which moves to distal chloroplasts *via* a cytoskeleton network (Edwards et al., 2004; Chuong et al., 2006). Similarly, the *Bienertia* species single-cell system has a unique form in which lateral chloroplasts function as mesophyll cells and a bubble-like central chloroplast compartment (CCC) acts as Kranz-cells in usual C_4_ species. This discovery stimulated scientists to deeply investigate the biology and genomics of this simplified system, which might have advantages for those looking for genetic engineering of C_4_ photosynthesis in C_3_ crop plants (Schuler et al., 2016).

Another peculiarity within the C_4_ plants of SW and Central Asia, is the presence of two types of photosynthesis within the life cycles of particular lineages and species of Chenopodiaceae. In these species C_3_ cotyledon leaves are replaced by C_4_ shoots. This characteristic is widespread in the subfamily Salsoloideae and rarely in Suadedoideae (Pyankov et al., 1999; Pyankov et al., 2000b; Pyankov et al., 2001; Akhani and Ghasemkhani, 2007). In both tribes of Salsoleae and Caroxyleae several genera, such as *Haloxylon, Halothamnus, Hammada, Girgensohnia, Noaea* and *Soda inermis* (*Salsola soda*)*, Climacoptera, Halimocnemis, Petrosimonia, Kaviria*, and *Halocharis* are known to have this switching mechanism. In Suaedoideae, this type was known in *Suaeda microphylla* evidenced by carbon isotope values (Akhani and Ghasemkhani, 2007) or anatomy (Khoshravesh and Akhani, unpublished data). As this characteristic is of interest for gene engineering, the transcriptomes of *Haloxylon ammodendron* and *Soda inermis* (*Salsola soda)* have been studied (Li et al., 2015; Lauterbach et al., 2017).

Ecologically, the development of a switching mechanism from a C_3_ to a C_4_ photosynthetic metabolism hasn’t however received much attention. Switching chenopods are mainly halophytes and xerohalophytes of continental temperate saline ecosystems (*Climacoptera, Petrosimonia, Halimocnemis, Soda inermis*), gypsiferous (*Halothamnus*), and sandy (*Haloxylon*) habitats of the Irano-Turanian floristic region and are taxonomically among the main and most biodiverse taxa of SW Asian C_4_ Eudicots. Switching plants are also among the main biomass producers in the Irano-Turanian deserts, suggesting that the switching mechanism may imply an evolutionary advantage to those species. The continental climates of their habitats may probably favor a switching mechanism and the presence of C_3_ cotyledons at early developmental stages, when germination at low temperatures favors the presence of C_3_ cotyledons while increasing temperatures during the growth period favor C_4_ leaves (Akhani and Ghasemkhani, 2007). Further investigations however are needed to comprehend better the ecological advantages in comparison with tropical deserts.

### Palaeoclimatic Implications

The distribution pattern of C_4_ plants in SW Asia is at least partly related to the palaeoclimatic conditions which have prevailed in the region during the Neogene. Today, the region is dominated by the summertime subtropical anticyclones (Zarrin et al., 2009) which induce a long summer drought in most parts of SW Asia. The subtropical anticyclonic system is particularly intensified and maintained by the high elevations in SW Asia (Zarrin et al., 2011) which are mostly present since at least 7 million years ago (Djamali et al., 2012). During the late Neogene, a long summer drought has thus dominated over the region impeding the penetration of moisture-bearing westerlies into the Irano-Anatolian inlands and Central Asia. The continental inlands of SW Asia, although close to the Indian Ocean, receive no monsoon precipitation during the summertime because of the complex monsoon-desert mechanism described by Rodwell and Hoskins (1996). The palaeoclimatic archives suggest that excepting the Arabian Peninsula, most of the continental interior of SW Asia has not received summer monsoon rainfall during the intensification phase of the latter at the beginning of the Holocene (Djamali et al., 2010). Only SE Iran might have received some direct summer precipitation from the summer monsoons some 11,400 to 6,500 years ago (Vaezi et al., 2019). Some of the C_4_ plant communities found in currently dry areas of S Iran and Arabian Peninsula (see above) may be the relicts of formerly widespread C_4_ communities when the area received more summer rainfall. Relatively higher values of δ^13^C of organic matter in the Jazmurian playa sediments during the early Holocene (**Figure 8A** in Vaezi et al., 2019) may indeed reflect the important contribution of more abundant C_4_ grasses during the Indian Monsoon intensification phase in SE Iran. With the exception of increasing summer rains in SE Iran and Arabia, it seems thus that most of SW Asia has always been subjected to long summer droughts and high continentality since several million years (Djamali et al., 2012). Such long-lasting geo-climatic conditions are characterized by strong continentality, long summer droughts and presence of intracontinental endorheic basins which support the formation of a broad range of saline environments suitable for the diversification and specialization of C_4_ Eudicots in particular the halophytic chenopods.

### Human Utilization of C_4_ Plants in Southwest Asia

SW Asia including the Fertile Crescent had a long history of plant domestication and land use (Zohary and Hopf, 2000). Deserts and steppe populations utilize many C_4_ species in a variety of ways, as source for food, fire wood, for grazing, construction, greening of their surroundings, medicine, and in recent times for desert reclamation programs and afforestation. They have additional potentials such as usage as biofuel, genetic engineering practices and even invention of new crops. C_4_ crops exploited in SW Asia either natively originated or widely distributed or imported from other parts of the world together with their main applications are listed in the **Supplementary Table 4**. It has to be noted, that although the wild forms of many C_4_ crops, like *Eragrostis tef, Echinochloa frumentacea, Panicum miliaceum, Eleusine coracana, Setaria italica, Soda inermis*, etc. are distributed throughout SW Asia, they are mostly cultivated outside of this region.

The most important and most species rich group of C_4_ crops are millets and millet-like cereals cultivated traditionally for their grain in arid areas of S Asia and Africa. Among them several major millet crops like sorghum (*Sorghum bicolor*) (Sanjana Reddy, 2017a), proso millet (*Panicum miliaceum*) (Gomashe, 2017), foxtail millet (*Setaria italica*) (Hariprasanna, 2017), and pearl millet (*Cenchrus americanus*) (Sanjana Reddy, 2017b) made it to fame out of their region of domestication and have been introduced not only to SW Asia but are extensively cultivated worldwide for their grains. Sweet sorghum (*Sorghum bicolor*) is also an alternative source of syrup and sugar (Sanjana Reddy, 2017a), although the main pantropical sugar crop remains the extensively cultivated sugarcane (*Saccharum officinarum*) (James, 2014). Although the wild forms of small millets, are distributed in SW Asia, they are mainly cultivated as traditional cereals of cultural significance outside of this area (Seetharam and Riley, 1986). In fact, they form important grain crops in the traditional communities of S Asia and Subsaharan Africa. An interesting example is cultivation of teff (*Eragrostis tef*) concentrated in Ethiopia and Eritrea, where it is of the most important crop plants and is used mainly for the production of traditional Injera flat bread (Seetharam and Riley, 1986). Teff recently however is wining fame as a healthy alternative cereal outside of E Africa. Molecular studies have shown that this allotetraploid is closely related to *Eragrostis pilosa*, growing in SW Asia (Ingram and Doyle, 2003; Assefa et al., 2017).

Millets cultivated on smaller scales in SW Asia include finger millet (*Eleusine coracana*), Indian barnyard millet (*Echinochloa frumentacea*), Japanese millet (*E. esculenta*), and adlay millet (*Coix lacryma-jobi*). Their cultivation in SW Asia is mainly limited to regions where they bear cultural significance, such as the plains and hills of Afghanistan and Pakistan (Breckle and Rafiqpoor, 2010; Breckle et al., 2013). Corn (*Zea mays*) is the world’s most important C_4_ grain crop and SW Asia is not an exception, where it is extensively cultivated (Staller, 2010).

Many wild SW Asian C_4_ grasses are important fodder and pasture crops, for biomass production or used for landscape greening on large and small scales in arid areas of N America, Australia, S Europe, Central Asia, India, and Subsaharan Africa, namely *Bouteloua curtipendula*, *Cenchrus* spp., *Chloris gayana*, *Cynodon dactylon*, *Diplachne fusca*, *Lasiurus scindicus*, *Panicum antidotale*, *Setaria viridis*, *Sorghum halepense* and *Sporobolus* spp. They may present a source for further millet and forage grass breeding and cultivation for forage and erosion control in disturbed and desertifying areas of SW Asia.

The C_4_ Monocots are of high importance for summer grazing of wildlife such as Persian Ibex or livestock on steep rocky outcrops and disturbed or degraded South Caspian forests (Akhani and Ziegler, 2002). Grazing on salt marsh grasslands dominated by *Aeluropus* is common in most parts of the region (Whigham et al., 1993).

The Saharo-Sindian and Somali-Masai vegetations of SW Asia are rich in highly productive high biomass Monocots. Genera like *Panicum, Cenchrus, Desmostachys bipinnata, Saccharum, Tripidium, Mischanthus, Paspalum, Pogonatherum*, and *Cyperus* are high biomass producers that both have a value for grazing and industrial applications (Serag, 2003; Tubeileh et al., 2016; Mullet, 2017).

*Haloxylon persicum, H. ammodendron, Xylosalosla richteri*, and *Calligonum* spp. may yield up to 1.2 t, 3.0 t, 1.3, and 1.2 t green biomass per hectare respectively, depending on habitat type and population density. *Haloxylon ammodendron* is definitely the largest species by biomass and can reach a height of up to 9 m and an age of up to 100 years (Fet and Atamuradov, 1994; Gintzburger et al., 2003) (**Figure 8K**).

Additionally, psammophytic *Xylosalsola* sp. in Turkmenistan and Central Asia and *Soda stocksii* (*Salsola stocksii*) and *Hammada salicornica* in Pakistan and India are grown for the same purpose. *Haloxylon ammodendron* is mainly cultivated in Central Asia, SE Europe, NW China, and Iran for as erosion control and forage on salt and clay deserts, saline flats, and saline sands. From the Mediterranean toward Iran *Atriplex* spp. like *A. halimus* and *A. canescens* are cultivated for the same purpose on saline clayey soils (Hanelt, 2001; Danin, 2007; Walker et al., 2014). *Bassia prostrata* has been cultivated in Turkmenistan, Central Asia, Europe, and the USA as forage and erosion control on clayey, slightly saline, sandy, and rocky soils (Dzyubenko and Soskov, 2014).

Several C_4_ crops are gaining importance as healthy food plants. To mention are seeds of *Amaranthus caudatus*, *A. cruentus*, and *A. hypochondriacus*, used as gluten-free pseudocereals with increasing cultivation worldwide (Sauer, 1967). Furthermore, several C_4_ Eudicots are cultivated as leaf vegetables. E.g., *Portulaca oleracea* is cultivated around the Mediterranean, in Iran, Turkey, and Transcaucasia (Gonnella et al., 2010; Uddin et al., 2014). *Suaeda aegyptiaca* is cultivated in southern Iran and *Amaranthus tricolor* in Pakistan, India, and E Asia for food (Akhani, 2006). *Soda inermis*, growing on saline soils throughout Armenia, Iran, Turkey, and Turkmenistan, is cultivated and highly prized as a leaf vegetable (agretti) in the Mediterranean region (Centofanti and Bañuelos, 2015). Another chenopod, the common ruderal and ornamental *Bassia scoparia*, is cultivated for the production of a caviar substitute in E Asia (“Useful Temperate Plants Database”; Han et al., 2006; Nedelcheva et al., 2007). Recent efforts have also been done to introduce locally collected leaf vegetables (*Cleome gynandra*, *Boerhavia* sp. and *Sesuvium sesuvioides*) and medicinal plants [*Blepharis* sp. (**Figure 10F**) and *Tribulus terrestris*] into cultivation (Hanelt, 2001; Cheikhyoussef et al., 2011; Mahesh et al., 2012; Boteva et al., 2014; Kripa and Vijayalakshmi, 2016; Salamon et al., 2016; ‘PROTA4U web database’, 2018). Furthermore, tuber bearing sedges, like *Cyperus esculentus* are interesting candidates as food crops, already cultivated throughout the world (Pascual et al., 2000; Arafat et al., 2009). Several C_4_ Eudicots [e.g., *Gisekia pharnacoides*, *Hypertelis cerviana*, *Suaeda* spp., *Tecticornia indica*, *Atriplex* spp., and *Soda* and *Salosla* spp.], are used locally as famine foods or collected as local leaf vegetables and may be interesting for further investigations as crop plants (Hanelt, 2001; “PROTA4U web database”, 2018; “Useful Temperate Plants Database”). Some C_4_ plants are cultivated worldwide for their essential oils, such as *Cymbopogon* spp. (citronella oil, palmarosa oil, etc.), *Chrysopogon gryllus*, *Chrysopogon zizanioides* (vetiver oil), and *Cyperus articulatus* (Duke, 1993; Hanelt, 2001; Simpson and Inglis, 2001; Bertea and Maffei, 2009; Atala, 2012; Pareek and Kumar, 2013). Some local species of *Cymbopogon* are already used in local medicine for their aromatic properties and may be of interest for introduction into cultivation (El-Kamali et al., 2005; Bertea and Maffei, 2009; Prasad et al., 2014). A number of C_4_ species are also favored for their biomass and as a source of fiber, mat, basket weaving, broom, and construction materials, and could be interesting candidates for the diversification of local agriculture. E.g., *Bassia scoparia*, *Cyperus corymbosus*, *C. malaccensis*, *C. pangorei*, *Desmostachya bipinnata* and *Eulaliopsis binata*, and *Sorghum bicolor* are already cultivated for fibers and high quality mat weaving in Iran, India, and Africa (Wendrich, 1989; Ravichandran et al., 2005; Sahu et al., 2010; Jana and Puste, 2014; Khyade et al., 2018; Shioya et al., 2019).

Finally, some C_4_ plants are frequently grown ornamentals (e.g., *Cyperus alternifolius*, *C. papyrus*, *Bouteloua gracilis*, *B. curtipendula*, *Stenotaphrum secundatum*, *Coix lacryma-jobi*, *Imperata cylindrica*, *Miscanthus nepalensis*, *Miscanthus sinensis*, *Schizachyrium scoparium*, *Setaria palmifolia*, *Tripiduium ravennae*, *Amranthus caudatus*, *A. tricolor*, *Gomphrena globosa*, *G. haageana*, *B. scoparia*, *Portulaca pilosa* and *P. grandiflora*).

With climate change, overpopulation and resource mismanagement, SW Asia is highly in need of alternative, drought, and salt resistant crops to make agriculture more sustainable. A series of C_4_ crops, in addition to those already introduced and cultivated in SW Asia, are highly interesting for further introduction into cultivation, since their wild forms are already distributed and adapted to SW Asian climate conditions.

### Conservation

Being mainly part of the so-called MENA Region (Middle East and North Africa), SW Asia with its xeric climates is highly susceptible to climate change (Pal and Eltahir, 2016). Although future scenarios vary, concerning the degree of climatic changes, a general consensus on the increase of mean temperatures and heat extremes exist (Evans, 2009; Waha et al., 2017). The same is regarding the decrease of precipitation and increase of drought and aridity, with the exceptions of the southern shores of SW Asia, where the increase of monsoon precipitation due to a shift of the inter-tropical convergence zone is expected to occur according to some predictions (Waha et al., 2017; Byrne et al., 2018). The effects of climate change are enhanced by growing population and an aggressive mismanagement of water and land resources. For example, in Iran an ineffective irrigation agriculture, extensive dam construction and groundwater over-use has led to a significant decrease of groundwater levels and the drying and destruction of the main lake, river, and wetland ecosystems (Motagh et al., 2008; Madani, 2014; Akhani, 2015b; Motagh et al., 2017). On the other hand, the SW Asian C_4_ flora, although highly specialized, is very susceptible to minor changes in many extreme habitats (groundwater levels, period, and amount of precipitation, etc.).

Of the 923 C_4_ species of SW Asia, 141 (105 Eudicot and 36 Monocots—15.3%) are endemic to SW Asia, while 70 species (50 Eudicots and 20 Monocots—7.6%) are strict country endemics with very limited habitats. However, even some species distributed beyond SW Asia (e.g., several *Cyperus* species described from limited areas of the Somali-Masai floristic region) show very restricted distributions. The strict country endemics can be grouped mainly in two subgroups: a) C_4_ Eudicots (mainly chenopods) mainly endemic to habitats of the Irano-Turanian floristic region; b) C_4_ Monocots (mainly Poaceae) endemic to the Somali-Masai floristic region (of those the half of the species are endemic to the island of Socotra). Under high climatic and anthropogenic pressure on the narrow habitats of SW Asian C_4_ endemics, many such species are critically endangered.

**Figure 11:**
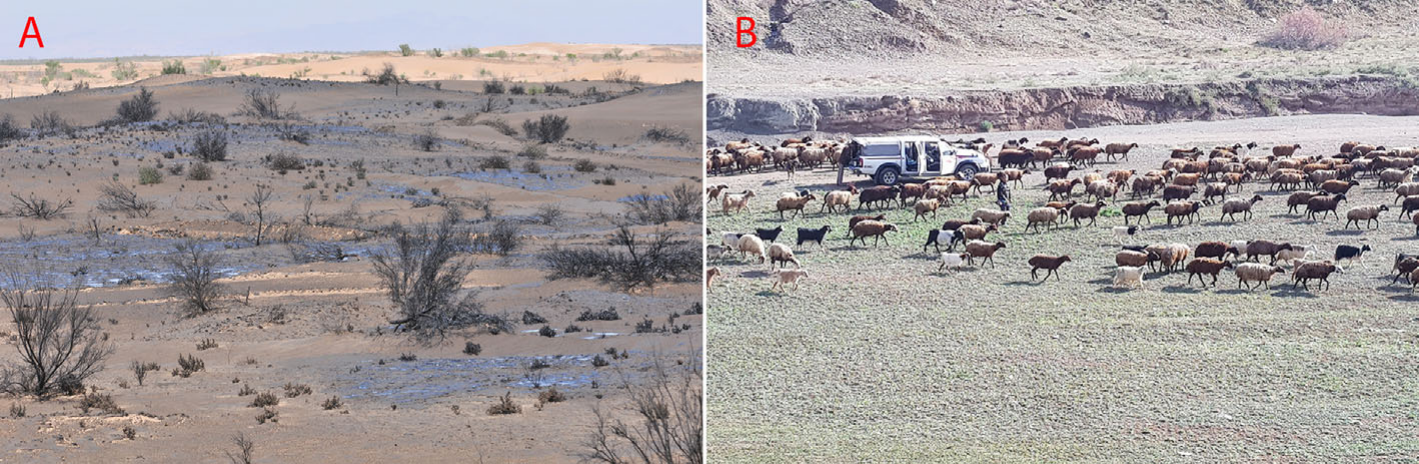
**(A, B)**: Mismanagement of C_4_ habitats in Iran. **(A)** Oil mulching in sand dunes with rich C_4_ plant species in Abuzeidabad, Kashan, Central deserts of Iran, 28.5.2020. **(B)** Overgrazing in gypsum and salty ground between Zanjan and Mianeh, Northeast (NE) Iran, 14.4.2018. (Photos by H. Akhani).

As an example, the recently discovered *Bienertia kavirense* Akhani (**Figure 10J**), restricted to a narrow region within Iran’s central saline desert, has been declared critically endangered from its discovery (Akhani et al., 2012). According to our own documentation in a saline flat located 60 km W of Tehran near Rude Shur (saline river), a very dense subpopulation of *Bienertia cycloptera* in 2003 completely disappeared in 2009 (Akhani et al., 2003; Akhani, 2016; Akhani and Rudov, 2018) apparently due to dropping of underground water levels. This tragic situation is observed in many similar habitats, where dropping of underground and subsurface water levels affects soil moisture and is consequently a threat for existence of many C_4_ annuals. Recent field trips of the authors discovered, that the narrow habitats of *Halimocnemis alaeflava* and *Halimocnemis azerbaijanensis* have been fragmented and partly destroyed by factory construction, complete removal of upper soil layers, as well as road and dam construction. In fact, if further localities of *H. alaeflava* are not discovered in future, there is a probability of its complete extinction as its type habitat is on the way toward complete destruction. Another critical example is a subpopulation of the local endemic *Caroxylon abarghuense* (**Figure 10K**) in Touran Biosphere Reserve, located in Central East of Iran. In a small valley dominated by *Tamarix* shrubs, only 16 living individuals of *C. abarghuense* have been found. Their seeds do not seem germinable probably because allee effect resulted from small size population. A subpopulation of *Piptoptera turkestana*, discovered in 1989 on sandy dunes of central Iran, ca. 30 km ESE of Kashan, could not be recollected in the same place after extensive searches and apparently disappeared from the locality probably due to habitat disturbance and oil mulching (**Figure 11A**). Several taxa (e.g., *Climacoptera zenobiae*) lack proper assessment and are known from very limited collection samples. Species like *Climacoptera czelekenica* Pratov, being island endemics, depend on the changing water level of the Caspian Sea. On the island of Socotra, the high number of endemic plants is threatened by both climate change and overgrazing (Attorre et al., 2007; Rejzek et al., 2016). This directly affects also the C_4_ endemics of Socotra, being all C_4_ grasses. Conservation of endangered C_4_ endemics is further complicated by the lack of proper population assessment in many regions of SW Asia because of lacks of interest and founding and specially because of inaccessibility due to long lasting military conflicts. The two of the four most endemic rich countries (Afghanistan and Yemen) are both long time battle grounds.

Additional threats to the local C_4_ flora are introduced and invasive C_4_ plants. The introduced C_4_ flora of SW Asia (68 species) is mainly composed of Poaceae (34 species), Amaranthaceae *sensu stricto* (20 species), and Euphorbiaceae (7 species) (Pahlevani et al., 2020). Several C_4_ lineages not typical for the region have been introduced to SW Asia (C_4_ *Flaveria* clade A, C_4_ *Alternanthera*, and *Gomphrena*). The genus *Amaranthus*, although a neophytic genus in most areas of SW Asia, includes not only recently introduced species but also apparently old neophytes (e.g., *A. blitum*) and local species (e.g., *A. graecizans*, *A. tenuifolius*, *A. sparganicephalus*). Of the 67 introduced C_4_ species at least 48 have been reported to be invasive in various regions of the world (“CABI—Invasive Species Compendium. Wallingford, UK: CAB International”; Elmore and Paul, 1983). These species may form a major threat to local floras and economic burdens for agriculture and livestock. An important aspect of invasiveness in the area is introducing C_3_ invasive species into C_4_ habitats. This happened in S Iran and Pakistan where introduction of *Prosopis juliflora* occupied many of the habitats of drought resident species including native C_4_ species.

The saline areas, sandy dunes, and marl or gypsum habitats, where the majority of C_4_ Eudicots grow, have no protection priority in most of the countries of SW Asia. Mostly, these habitats are considered as badlands with poor biodiversity. Grazing and shortage of water are big problems affecting the vegetation in such habitats. Therefore, most of these lands with poor vegetation cover are converted into dust emission centers with a huge environmental concern (Akhani, 2015b). An example of mismanagement of such habitats we refer to intensive oil mulching in many sand dunes in Iran with dense C_4_ plant vegetation. Such activities which aim to control dune movement result in destroying natural flora with many C_4_ annuals and shrubs (**Figure 11A**). Sadly, improper managements threatened many gypsophytic, halogypsophytic, and xerophytic hill habitats, which are the main habitats of endemic C_4_ chenopods (Akhani, 2006; Ghorbanalizadeh et al., 2020) (**Figure 11B**). We strongly recommend re-evaluation of protection policies to restore and protect C_4_-rich habitats that are of high advantage in desert areas because of their low water requirements and vegetation cover provision during harsh seasons.

## Conclusions

SW Asia is not only an area of origin and diversification of most interesting and highly adapted C_4_ Eudicot lineages, but also provide diverse and vast habitats for growing C_4_ Monocots. The evolution of various eco-morphological traits among C_4_ Eudicots lineages (notably single-cell functioning C_4_ plants and switching C_3_–C_4_ species) and the presence of many endemic species are indicative of long-lasting ecological pressure that supports speciation and specialization among different families in particular Chenopodiaceae and *Calligonum* (Polygonaceae).

The C_4_ Eudicots are known to dominate vast Irano-Turanian deserts. Our data suggest, that this is mainly related to the adaptation of C_4_ Eudicots to the continental climatic conditions of the Irano-Turanian deserts in contrast with the C_4_ Poaceae, that dominate areas with the presence of summer rainfall in the southern and southeastern parts of the area influenced by monsoon summer rains.

Unfortunately, the SW Asian C_4_ plant diversity is threatened by the impact of intensive land use synergized by global warming and rapid desertification. In spite of our knowledge on the taxonomy and phylogeny of many C_4_ lineages, many questions regarding SW Asian C_4_ plants are however still unresolved. For example, the taxonomy of the genera *Calligonum*, *Climacoptera, Hammada*, *Kaviria*, *Caroxylon*, and *Tribulus* needs still to be clarified. More studies are necessary to understand the phylogenetic relationships of C_3_ and C_4_ species of the genera *Fimbristylis*, *Polycarpaea* and clarify ambiguities in presence of some C_3_ and C_3_–C_4_ intermediate lineages within prevalently C_4_ Salsoloideae (Chenopodiaceae). The study of the vegetation of some neglected areas and the description of some specific C_4_ plant dominated communities as well as the compilation of some country floras in the region (e.g., Syria, Jordan) would be highly informative.

Due to political instability and low interest in the conservation of desert areas with lower biodiversity, several rare C_4_ endemics are under the threat of extinction. Being part of the ecologically and climatically vulnerable MENA region, SW Asia is also in need of a sustainable management of water resources and agriculture. The C_4_ dominated habitats requires protection priority and monitoring of highly adapted plants to harsh environments. We re-emphasize the importance of regional C_4_ crops and the selection of new C_4_ crop candidates with lesser ecological impact than genetic engineering as a much more sustainable approach to guarantee the food security facing the future global change.

## Data Availability Statement

The original contributions presented in the study are included in the article/supplementary material; further inquiries can be directed to the corresponding author.

## Author Contributions

AR provided the data, wrote the first draft, prepared all figures and tables. HA planned and supervised the research, contributed to the preparation of the data and text, provided all photos, edited the text and jointly worked together in writing of first draft. MM analyzed all the carbon isotopes in the paper, read, and edited the manuscript. MD contributed to the writing of the palaeoecology part of the paper.

## Funding

This research was funded by postdoctoral support for the first author by the International Office of the University of Tehran and the Erasmus Mundus Marhaba Program.

## Conflict of Interest

The authors declare that the research was conducted in the absence of any commercial or financial relationships that could be construed as a potential conflict of interest.

## Supplementary Material

Supplementary Appendix Table 1 Complete List of C4 plants in Southwest Asia showing their C_4_ lineage, life form, chorotype, endemism, ecotype, δ^13^C and C_4_ subtype and respective leaf or shoot anatomical type.

Supplementary Figure 1 Climatic diagrams of selected 29 stations in different parts of SW Asian countries obtained from of the Iranian Meterorological Organization (IRIMO), Scholte and De Geest (2010) and Raza et al. (2015).

Supplementary Table 2 List of *Polycarpaea* (Caryophyllaceeae) species known from SW Asia which we could not test their photosynthetic type because of absence of material.

Supplementary Table 3 The List of *Polycarpaea* (Caryophyllaeceae), *Cyperus* and *Fimbristylis* (Cyperceae) species with C_3_ type carbon isotope values.

Supplementary Table 4 List of C_4_ plants with economic importance in SW Asia including non-native cultivated or introduced species.

Supplementary Data Sheet 1 References of floristic database.

## Nomenclatural Appendix

### Chenopodiaceae

Disintegration of *Salsola* s.l. into several lineages following molecular evidences lead to ample nomenclatural changes (Akhani et al., 2007). A major change was distinguishing *Kali* as a separate genus encompassing species of *Salsola* sect. *Salsola* with the type of *Kali turgida* (Dumort.) Guterm., (Gutermann, 2011). This system was accepted by many botanists and most nomenclatural changes were fixed according to the rules of International Code of Nomenclature (Hakobyan, 2011; Brullo et al., 2015; see additional notes and references in Sukhorukov et al., 2019). Two additional independent studies using one nuclear (ITS) and four plastid markers (*atp*B-*rbcL* spacer, *ndh*F-*rpl*32 spacer, *trn*Q-*rps*16 spacer, *rpl*16 intron) strongly confirmed the monophyly of *Salsola* s. str. sensu Akhani et al. (2007) (Voznesenskaya et al., 2013; Schüssler et al., 2017). A proposal to retypify the genus *Salsola* with *Salsola kali* by Mosyakin et al. (2014) did not get a consensus in Nomenclatural Committee (Applequist, 2016). However, in the last edition of the ICN, *Salsola kali* L. is accepted as the type of the genus *Salsola* (Turland et al., 2018). This unexpected decision necessitates several nomenclatural changes which we apply for those affected species occurring in the area of this paper.

***Caroxylon omanense* (Boulos) Akhani and Rudov *comb. nov.*** Basionym: *Salsola omanensis* Boulos, Kew Bull. 46(2): 297 (1991).

***Soda austro-iranica* (Akhani) Akhani *comb. nov.*** Basionym: *Salsola austro-iranica* Akhani in Pl. Veg. N. W. Persian Gulf 286 (2015).

***Soda cyrenaica*** **(Maire and Weiller) Akhani *comb. nov.*** Basionym: *Darniella cyrenaica* Maire and Weiller, Bull. Soc. Hist. Nat. Afrique N. 1939, xxx. 301.

***Soda drummondii* (Ulbr.) Akhani *comb. nov.*** Basionym: Nat. Pflanzenfam., ed. 2 [Engler and Prantl] 16c: 565 (1934).

***Soda florida* (M. Bieb.) Akhani *comb. nov.*** Basionym: *Anabasis florida* M.Bieb., Fl. Taur.-Caucas. 1: 190 (1808).

***Soda foliosa* (L.) Akhani *comb. nov.*** Basionym: *Anabasis foliosa* L., Sp. Pl. 1: 223 (1753).

***Soda grandis*** **(Freitag, Vural and N. Adiguzel) Akhani** ***comb. nov.*** Basionym: *Salsola grandis* Freitag, Vural and Adıgüzel, Willdenowia 29(1–2): 131 (1999).

***Soda cinerea* (Moq.) Akhani *comb. nov.*** Basionym: *Halogeton*? *cinerea* Moq., Chenopod. Monogr. 159 (1840).

***Soda kerneri* (Wol.) Akhani *comb. nov.*** Basionym: *Hypocylix kerneri* Woł., Denkschr. Kaiserl. Akad. Wiss., Wien. Math.-Naturwiss. Kl. 51(2): 276 (1886).

***Soda longifolia*** **(Forssk.) Akhani *comb. nov.*** Basionym: *Salsola longifolia* Forssk., Fl. Aegypt.-Arab. 55. (1775).

***Soda makranica* (Freitag) Akhani *comb. nov.*** Basionym: *Salsola makranica* Freitag, Fl. Iranica [Rechinger] 172: 166 (1997).

***Soda oppositifolia* (Desf.) Akhani *comb. nov.*** Basionym: *Salsola oppositifolia* Desf., Fl. Atlant. 1: 219 (1798).

***Soda rosmarinus*** **(Ehrenb. ex Boiss.) Akhani *comb. nov.*** Basionym: *Seidlitzia rosmarinus* Ehrenb. ex Boiss., Fl. Or. 4: 951, 1879.

***Soda schweinfurthii* (Solm.) Akhani *comb. nov.*** Basionym: *Salsola schweinfurthii* Solms, Bot. Zeitung, 2. Abt. 59: 173, in obs. (1901).

***Soda stocksii* (Boiss.) Akhani comb. nov.** Basionym: *Salsola stocksii* Boiss., Diagn. Pl. Orient. ser. 2, 4: 75 (1859).

#### Poaceae

The phylogenetic relationships within Poaceae has been investigated in several recent revisions (Hodkinson et al., 2002; Kellogg, 2015; Soreng et al., 2015; 2017). These revisions regarded also changes within the PACMAD clade, which includes all C_4_ Poaceae lineages. According to these revisions *Pennisetum* und *Snowdenia* has been synonymized with *Cenchrus* and *Brachiaria* (s.s.) with *Urochloa*. Several species have been however neglected by the taxonomic changes. We propose here new combinations for the following four species, that have been affected by the last revision by Soreng et al. (2017):

***Cenchrus aethiopicus* (Fresen.) Rudov *comb. nov.*** Basionym: *Beckera polystachya* Fresen., Mus. Senckenberg. ii. 132 (1837).

***Cenchrus glaucifolius* (Hochst. ex A.Rich.) Rudov and Akhani *comb. nov.*** Basionym: *Pennisetum glaucifolium* Hochst. ex A. Rich. Tent. Fl. Abyss. 2: 382 (1841).

***Cenchrus nubicus* (Hochst.) Rudov and Akhani *comb. nov.*** Basionym: *Gymnotrix nubica* Hochst., Flora 27(1): 251 (1844).

***Cenchrus yemensis* (Deflers) Rudov and Akhani comb. nov.** Basionym: *Pennisetum yemense* Deflers, Voyage Yemen 217 (1889).

***Urochloa arida (*Mez) Rudov *comb. nov.*** Basionym: *Panicum aridum* Mez, Bot. Jahrb. Syst. 34(1): 139 (1905.

***Urochloa chusqueoides* (Hack.) Rudov** ***comb. nov.*** Basionym: *Panicum chusqueoides* Hack., Bull. Herb. Boissier iii. 377 (1895).

***Urochloa ovalis* (Stapf) Rudov *comb. nov.*** Basionym: *Brachiaria ovalis* Stapf, Fl. Trop. Afr. [Oliver et al.] 9(3): 546 (1919).
