# Supplementary material for: A Review of C4 Plants in Southwest Asia: An Ecological, Geographical and Taxonomical Analysis of a Region With High Diversity of C4 Eudicots
Source: Front Plant Sci. 2020 Nov 5;11:546518. doi: 10.3389/fpls.2020.546518 (PMC7694577; doi:10.3389/fpls.2020.546518)
Supplement: Supplementary Appendix Table 1 — Complete List of C4 plants in Southwest Asia showing their C4 lineage, life form, chorotype, endemism, ecotype, δ13C and C4 subtype and respective leaf or shoot anatomical type. [file Table_1.pdf]

Appendix Table 1. Complete List of C4 plants in Southwest Asia showing their C_4 lineage, life form, chorotype, endemism, ecotype, $\delta^{13}C$ and C_4 subtype and respective leaf or shoot anatomical type. New isotope data are marked with an asterisk, unpublished data by H.Ziegler are marked with a plus.

Abbreviation: **Life-forms: T** (Therophyte), **H** (Hemicryptophyte), **GH** (Graminoid hemicryptophytes including stoloniferous Cyperaceae), **Ch** (Chamaephyte), **P** (Phanerophyte), **HY** (Hydrophyte)

Chorotypes: PL (Pluriregional), **IT** (Irano-Turanian), **SS** (Saharo-Sindian), **SM** (Somali-Masai), **I** (Indian), **IC** (Indochinese), **M** (Mediterranean), **ES** (Euro-Siberian), **CA** (Central Asian), **SJ** (Sino-Japanese), **GC** (Guineo-Congolian), **ZI** (Zanzibar-Inhambane), **Z** (Zambezian) , **SU** (Sudanian), **MA** (Madrean), **BR** (Brazilian), **CR** (Caribbean), **NA** (North American), **AM** (Amazonian), **AN** (Andean), **CP** (Chile-Patagonian)

Endemism: N (Native) **Ne** (Native Endemic), **I** (Introduced), **Ii** (Invasive Introduced)

Ecotypes: GP (Gypsophytes), **HD** (Hydrophytes), **HG** (Hygrophytes, including plants growing on swamps, river banks and flooded soils), **HL** (Euhalophytes, incl. hygrohalophytes), **I** (Inland), **L** (Littoral), **MS** (Mesophytes, including species growing in moderate wet soils, meadows, forests and forest openings), **O** (Oreophytes incl. chasmophytes and plants growing in steep rocky outcrops), **PH** (Psammohalophytes), **R** (Ruderals including weedy species), **Tm** (Temperate), **Tr** (Tropical): The four subdivisions of Littoral, Inland, Temperate and Tropical are only given for those taxa which occur exclusively under specified category, **XE** (Xerophytes), **P** (Psammophytes), **XH** (Xerohalophytes, incl. gypsohalophytes and salt tolerant xerophytes).

$\Delta^{13}C$ ration: AR (Analysis required)

Leaf anatomy: AT (Atriplicoid), **SL** (Salsoloid), **PT** (Portulacelloid), **KC** (Kochioid), **KT** (Kranz-Tecticornoid), **SC** (Single-cell), **SD** (Suaedoid), **CS** (Conospermoid), **PI** (Pilosoid), **FS** (Fimbristyloid), **CC** (Chlorocyperoid), **NA** (Neurachneoid), **CL** (Classic), **AR** (Aristidoid), **AU** (Arundinelloid), **SA** (Stipagrostoid), **GC** (Glossocardoid), ‘ ’ (C_4 pathway deduced based on respective known C_4 clade)

Metabolic types: NAD-ME, NADP-ME, PEP-CK

Note leaf anatomy and metabolic pathways marked with ‘’ are deduced based on respective C_4 clade. If the C_4 clade shows variability of leaf anatomy or metabolic pathways, deductions are made only for phylogenetic subclades with known data. In all other cases no data are indicated. References in the column may refer to leaf anatomy and metabolic subtypes. References standing behind deduced data are referring to publications used for deduction

C_4 lineage **(bold)** and species	Life form	Choro-type	Ecotype	Distribution in Southwest Asia																			$\delta^{13}C$	Leaf anatomy, C_4 Photosynthetic subtype (references)
				Afghanistan	Armenia	Azerbaijan	Bahrain	Iran	Iraq	Israel/Palestine	Jordan	Kuwait	Lebanon	Oman	Pakistan	Qatar	Saudi Arabia	Sinai	Syria	Turkey	Turkmenistan	UAE	Yemen		
**MONOCOTS**																									
**ALISMATALES; Hydrocharitaceae**																									
**1)** ***Elodea***																									
Elodea densa (Planch.) Casp.	HY	AM, BR	HD																	Ii					Facultative SC/NADP-ME (50)
**2)** ***Hydrilla***																									

C_4 lineage **(bold)** and species	Life form	Choro-type	Ecotype	Distribution in Southwest Asia																				δ13C	Leaf anatomy, C_4 Photosynthetic subtype (references)
				Afghanistan	Armenia	Azerbaijan	Bahrain	Iran	Iraq	Israel/Palestine	Jordan	Kuwait	Lebanon	Oman	Pakistan	Qatar	Saudi Arabia	Sinai	Syria	Turkey	Turkmenistan	UAE	Yemen		
Hydrilla verticillata (L. f.) Royle	HY	PL	HD	N				N							N										Facultative SC/NADP-ME (50)
**POALES**																									
**Cyperaceae**																									
**3) *Bulbostylis***																									
Bulbostylis atrosanguinea (Boeckeler) C.B.Clarke	GH	PL	MS-I-Tr																				N	-16.0(26)	'FS/NADP-ME' (51, 52, 76)
Bulbostylis barbata (Rottb.) C.B.Clarke	T	PL	MS-Tr												N								N	-11.1(26)	FS/NADP-ME (51, 52, 76)
Bulbostylis densa (Wall. ex Roxb.) Hand.-Mazz.	T	PL	MS, R												N									-12.1(26)	'FS/NADP-ME' (51, 52, 76)
Bulbostylis hispidula (Vahl) R.W.Haines	T or H	PL	HG																	N			N	-12.1(26)	'FS/NADP-ME' (51, 52, 76)
Bulbostylis humilis (Kunth) C.B.Clarke	T	SM	MS-I-Tr																				N	-14.7(26)	'FS/NADP-ME' (51, 52, 76)
Bulbostylis tenerrima (Fisch. & C.A.Mey. ex Ledeb.) Palla	T	ES, IT	HG-Tm																	N				-15.90 (*)	'FS/NADP-ME' (51, 526)
**4) *Cyperus* (incl. *Kyllinga, Mariscus* and *Pycreus*)**																									
Cyperus alopecuroides Rottb.	T or GH	PL	HG-Tr						N	N	N		N		N		N	N	N				N	-12.3(26)	'CC/NADP-ME' (52)
Cyperus alulatus J.Kern	T	PL	HG-Tr	N										N	N									-14.2(26)	'CC/NADP-ME' (52)
Cyperus amabilis Vahl	T	PL	HG-Tr																				N	-13.0(28)	'CC/NADP-ME' (52)
Cyperus amauropus Steud.	GH	SM	O-I-Tr														N						N	-14.0(28)	'CC/NADP-ME' (52)
Cyperus arenarius Retz.	GH	SS, I, IC	PS-L-Tr				N	N	N					N	N							N		-13.19 (*)	'CC/NADP-ME' (52)
Cyperus articulatus L.	GH	PL	HG-Tr					N							N		N	N					N	-11.0(26)	'CC/NADP-ME' (52)
Cyperus atkinsonii C.B.Clarke	T or GH	SS, I	PS-Tr					N							N										'CC/NADP-ME' (52, 61)
Cyperus aucheri Jaub. & Spach	GH	SS	XE-I-Tr	N			N	N	N			N		N	N	N	N					N		-12.93 (*)	'CC/NADP-ME' (52)
Cyperus brevifolius (Rottb.) Hassk.	GH	PL	HG	N										N	N								N	-10.08 (24)	CC/NADP-ME (52, 74)
Cyperus bulbosus Vahl	GH	PL	PS-Tr					N							N		N						N	-14.6(26)	'CC/NADP-ME' (52)
Cyperus capitatus Vand.	GH	M	PS-L-Tm							N	N		N					N	N	N				-11.1(28)	'CC/NADP-ME' (52)
Cyperus celans Kukkonen	GH	SS	PS-Tr					N																	'CC/NADP-ME' (52, 61)
Cyperus chlorotropis (Steud.) Mattf. & Kuk.	GH	SM	MS-Tr														N						N		'CC/NADP-ME' (52, 62)
Cyperus compactus Retz.	GH	PL	HG-Tr												N									-11.80 (*)	CC/NADP-ME (52, 74)
Cyperus compressus L.	T	PL	R	N											N								N	-12.8 (29)	CC/NADP-ME (52, 74)
Cyperus congestus Vahl	T	PL	PS-L-Tr			I														I				-9.6 (24)	'CC/NADP-ME' (52)
Cyperus conglomeratus Rottb.	GH	SS, SM, I	PS-L-Tr	N				N		N	N		N	N	N		N	N	N			N	N	-11.6 (28)	'CC/NADP-ME' (52)
Cyperus corymbosus Rottb.	GH	PL	HG-Tr						N						N									-10.33(28)	'CC/NADP-ME' (52)
Cyperus cruentus Rottb.	GH	SS, SM, ZI	O-Tr											N			N						N	-10.97 (*)	'CC/NADP-ME' (52)
Cyperus cuspidatus Kunth	T	PL	HG-Tr												N									-16.0(26)	'CC/NADP-ME' (52)
Cyperus cyperinus (Retz.) Suringar	GH	SS	MS-Tr																				N		CC/NADP-ME (52, 63, 64, 74)
Cyperus cyperoides (L.) Kuntze	GH	PL	MS, R-Tr												N									-11.8(26)	'CC/NADP-ME' (52)
Cyperus digitatus Roxb.	GH	PL	HG-Tr												N			N						-13.4 (28)	'CC/NADP-ME' (52)
Cyperus dilatatus Schumach.	GH	PL	HG-Tr																				N		'CC/NADP-ME' (52, 65)

C_4 lineage **(bold)** and species	Life form	Chorotype	Ecotype	Distribution in Southwest Asia																				$\delta^{13}C$	Leaf anatomy, C_4 Photosynthetic subtype (references)
				Afghanistan	Armenia	Azerbaijan	Bahrain	Iran	Iraq	Israel/Palestine	Jordan	Kuwait	Lebanon	Oman	Pakistan	Qatar	Saudi Arabia	Sinai	Syria	Turkey	Turkmenistan	UAE	Yemen		
Cyperus dives Del.	GH	PL	HG-Tr												N			N						-9.9(26)	'CC/NADP-ME' (52)
Cyperus dubius Rottb.	GH	PL	HG-Tr											N	N								N	-12.7(28)	'CC/NADP-ME' (52)
Cyperus dwarkensis K.C.Sahni & H.B.Naithani	T	SS, SM	HG-Tr	N										N	N										'CC/NADP-ME' (52, 66, 70)
Cyperus elegantulus Steud.	GH	PL	MS-I-Tr														N						N	-12.2(26)	'CC/NADP-ME' (52)
Cyperus eremicus Kukkonen	GH	SS	PS-I-Tr	Ne				Ne									Ne							-12.93 (*)	'CC/NADP-ME' (52)
Cyperus esculentus L.	GH	PL	HG	N				N	N						N		N			N				-12.7(26)	'CC/NADP-ME' (52)
Cyperus exaltatus Retz.	GH	PL	HG-Tr												N									-11.7(26)	CC/NADP-ME (52, 74)
Cyperus fissus Steud.	GH	SM	MS-I-Tr																				N		'CC/NADP-ME' (52, 67)
Cyperus flavescens L.	T or GH	PL	HG	N	N	N		N	N	N	N		N		N				N	N			N	-10.4(26)	'CC/NADP-ME' (52)
Cyperus flavidus Retz.	T or GH	PL	HG	N	N	N		N	N	N	N		N		N				N	N				-12.4 (27)	CC/NADP-ME (52, 74)
Cyperus forskalianus Vare & Kukkonen	T	SM	PS-I-Tr																				Ne		'CC/NADP-ME' (52,61, 89)
Cyperus glaber L. (incl. *C. noeanus* Boiss.)	T or GH	PL	HL-Tm	N	N	N		N	N	N	N		N	N	N				N	N	N			-11.7(29)	'CC/NADP-ME' (52, 90)
Cyperus glomeratus L.	T or GH	IT, ES	HG-Tm			N		N							N					N				-12.8 (29)	CC/NADP-ME (52, 74)
Cyperus hamulosus M. Bieb.	T	PL	PS																		N			-12.36 (68)	'CC/NADP-ME' (52)
Cyperus imbricatus Retz.	GH	PL	HG-Tr	N											N									-13.1(28)	CC/NADP-ME (52, 74)
Cyperus iria L.	T or GH	PL	HG	N				N	N					N	N									-11.0(26)	CC/NADP-ME (52, 74)
Cyperus jeminicus Rottb.	GH	PL	XE-Tr							N	N			N			N					N	N	-12.7 (28)	'CC/NADP-ME' (52)
Cyperus laevigatus L.	GH	PL	HL	N			N	N	N	N	N		N	N	N		N	N	N	N		N	N	-12.0(26)	'CC/NADP-ME' (52)
Cyperus latifolius Poir.	GH	PL	HG-Tr							N														-13.0 (26)	'CC/NADP-ME' (52)
Cyperus longus L.	GH	PL	HG	N	N	N	N	N	N	N	N	N	N	N	N		N		N	N	N	N	N	-12.18(+)	'CC/NADP-ME' (52)
Cyperus macrorrhizus Nees	GH	SS, SM	PS-L-Tr					N																	'CC/NADP-ME' (52, 61)
Cyperus malaccensis Lam.	GH	PL	HG-Tr					N	N						N									-14.01 (*)	'CC/NADP-ME' (52)
Cyperus metzii (Hochst. ex Steud.) Mattf. & Kuk	T	PL	MS												N										'CC/NADP-ME' (52, 69)
Cyperus michelianus (L.) Delile (incl. *C. pygmaeus* Rottb.)	T	PL	HG	N	N	N			N	N	N		N	N	N		N		N	N	N		N	-12.3(28)	CC/NADP-ME (52, 74)
Cyperus microbolbos C.B.Clarke	GH	SM	PS-L-Tr																				N		'CC/NADP-ME' (52, 81)
Cyperus microstylus (C.B.Clarke) Mattf. & Kuk	GH	SM	MS-Tr																				N	-14.1 (26)	'CC/NADP-ME' (52)
Cyperus mindorensis (Steud.) Huygh	GH	PL	MS												N										'CC/NADP-ME' (52, 71)
Cyperus nitidus Lam.	GH	PL	MS, R-Tr							N									N					-12.5(26)	'CC/NADP-ME' (52)
Cyperus niveus Retz.	GH	PL	O-I-Tr	N				N							N		N						N	-11.26 (*)	'CC/NADP-ME' (52)
Cyperus nutans Vahl	GH	PL	HG-Tr	N						N	N			N	N								N	-11.32 (*)	CC/NADP-ME (52)
Cyperus odoratus L.	T or GH	PL	PS			N		N		N	N												N	-10.5(28)	'CC/NADP-ME' (52)
Cyperus pachyrhizus Nees ex Boeckeler	GH	I	PS-L-Tr												N										'CC/NADP-ME' (52, 81)
Cyperus pangorei Rottb.	GH	PL	HG-Tr												N									-11.16 (*)	'CC/NADP-ME' (52)
Cyperus pannonicus Jacq.	T or GH	PL	HL			N														N				-14.1 (29)	'CC/NADP-ME' (52)
Cyperus papyrus L.	GH	PL	HG-Tr							N	N													-13.3(26)	'CC/NADP-ME' (52)

				Distribution in Southwest Asia																					
C_4 lineage **(bold)** and species	Life form	Choro-type	Ecotype	Afghanistan	Armenia	Azerbaijan	Bahrain	Iran	Iraq	Israel/Palestine	Jordan	Kuwait	Lebanon	Oman	Pakistan	Qatar	Saudi Arabia	Sinai	Syria	Turkey	Turkmenistan	UAE	Yemen	$\delta^{13}C$	Leaf anatomy, C_4 Photosynthetic subtype (references)
Cyperus plateilema (Steud.) Kuk.	GH	SM, SU, ZI	MS-I-Tr																				N	-10.7 (26)	'CC/NADP-ME' (52)
Cyperus polystachyos Rottb.	T or GH	PL	HG												N			N		N				-12.5(26)	CC/NADP-ME (52, 74)
Cyperus pseuderemicus Kukkonen & Vare	GH	SS	PS-L-Tr				Ne		Ne			Ne		Ne		Ne	Ne					Ne	Ne	-12.01 (*)	'CC/NADP-ME' (52, 89)
Cyperus pumilus L.	T	PL	R						N					N	N								N	-16.7(26)	'CC/NADP-ME' (52)
Cyperus rigidifolius Steud.	GH	PL	HG, R-Tr														N						N	-12.9(28)	'CC/NADP-ME' (52)
Cyperus rotundus L.	GH	PL	MS, HG	N	N	N	N	N	N	N	N	N	N	N	N	N	N	N	N	N	N	N	N	-12.47 (+)	CC/NADP-ME (52, 74)
Cyperus rubicundus Vahl	T or GH	PL	MS, O-Tr											N			N						N	-11.8(28)	'CC/NADP-ME' (52)
Cyperus sanguinolentus Vahl	T or GH	PL	HG	N		N		N							N		N			N			N	-12.1(26)	CC/NADP-ME (52, 74)
Cyperus schimperianus Steud.	GH	PL	HG-I-Tr														N						N	-11.1(26)	'CC/NADP-ME' (52)
Cyperus serotinus Rottb.	GH	PL	HG	N		N		N							N					N				-13.1 (28)	CC/NADP-ME (52, 74)
Cyperus sesquiflorus (Torr.) Mattf. & Kuk.	GH	PL	MS, O-I-Tr																				N		CC/NADP-ME (52, 74)
Cyperus sharonensis Danin & Kukkonen	GH	M	XE							Ne															'CC/NADP-ME' (52, 72, 72)
Cyperus squarrosus L.	T	PL	MS	N										N	N		N						N	-13.1(28)	'CC/NADP-ME' (52)
Cyperus steadii Kuk.	T	SS	PS-L-Tr					Ne						Ne	Ne							Ne	Ne	-11.92 (*)	'CC/NADP-ME' (52)
Cyperus stolonifer Retz.	GH	PL	PS-L-Tr												N									-12.02 (*)	CC/NADP-ME (52, 74)
Cyperus tenuiculmis Boeckeler	GH	PL	MS-I-Tm																				N	-11.9 (28)	'CC/NADP-ME' (52)
Cyperus tenuifolius (Steud.) Dandy	GH	PL	HG-Tr												N									-12.8(26)	'CC/NADP-ME' (52)
Cyperus wissmannii O.Schwartz	GH	SM	XE, PS-L-Tr											N									N	-13.26 (*)	'CC/NADP-ME' (52)
**5) C_4 *Fimbristylis***																									
Fimbristylis bisumbellata (Forsk.) Bubani	T	PL	HG	N	N			N	N	N	N		N		N		N	N	N	N			N	-13.7(26)	'FS/NADP-ME' (52, 76)
Fimbristylis complanata (Retz.) Link	GH	PL	HG-Tr												N								N	-15.5(26)	FS/NADP-ME (52, 76)
Fimbristylis cymosa R. Br.	GH	PL	HG-L					N						N	N		N					N	N	-14.0(26)	FS/NADP-ME (52, 76)
Fimbristylis dichotoma (L.) Vahl (incl. *F. annua* (Allioni) Roem. & Schult.)	T or GH	PL	HG	N		N				N	N		N		N		N	N	N	N	N		N	-9.9(26), -11.04[1] (30)	FS/NADP-ME (52, 76)
Fimbristylis falcata (Vahl) Kunth	GH	PL	MS												N										'FS/NADP-ME' (52, 76)
Fimbristylis ferruginea (L.) Vahl	GH	PL	HG-Tr			N			N	N	N			N	N		N		N	N		N	N	-13.0(26)	'FS/NADP-ME' (52, 76)
Fimbristylis littoralis Gaud.	T or GH	PL	HG	N				N	N															-12.68 (*)	FS/NADP-ME (52, 76)
Fimbristylis ovata (Burm.f.) J.Kern	GH	PL	MS-Tr												N								N	-11.3(28)	'FS/NADP-ME' (52, 76)
Fimbristylis quinquangularis (Vahl) Kunth	T or GH	PL	HG-Tr	N										N	N									-12.4(26)	'FS/NADP-ME' (52, 76)
Fimbristylis rigidula Nees	GH	I, IC	MS-Tr												N										'FS/NADP-ME' (52, 76)
Fimbristylis schoenoides (Retz.) Vahl	T or GH	PL	HG-L-Tr												N									-10.77 (30)	'FS/NADP-ME' (52, 76)
Fimbristylis squarrosa Vahl	T	PL	HG			N		N																-9.8 (24)	'FS/NADP-ME' (52, 76)
Fimbristylis tenera Schult.	T or GH	I	PS-Tr												N										FS/NADP-ME(52, 73, 76)
Fimbristylis umbellaris (Lam.) Vahl	GH	I,IC	HG-Tr												N										FS/NADP-ME(52, 74, 76)
Fimbristylis woodrowii C.B.Clarke	T	I	PS-Tr												N										'FS/NADP-ME' (52, 76)
**Poaceae**																									
**6) C_4 *Alloteropsis***																									

C_4 lineage **(bold)** and species	Life form	Choro-type	Ecotype	Distribution in Southwest Asia																				δ^{13}C	Leaf anatomy, C_4 Photosynthetic subtype (references)
				Afghanistan	Armenia	Azerbaijan	Bahrain	Iran	Iraq	Israel/Palestine	Jordan	Kuwait	Lebanon	Oman	Pakistan	Qatar	Saudi Arabia	Sinai	Syria	Turkey	Turkmenistan	UAE	Yemen		
Alloteropsis cimicina (L.) Stapf	T	PL	MS-Tr												N									-13.20 (31)	NA/NAD-ME (or PEP-CK) (53, 57, 75)
7) Andropogoneae																									
Andropogon amethystinus Steud.	GH	PL	MS, O-I																				N		'CL/NADP-ME' (54, 56, 57)
Andropogon bentii Stapf	GH	SM	XE-I-Tr																				Ne		'CL/NADP-ME' (54, 56, 57)
Andropogon chinensis (Nees) Merr.	GH	PL	O-I-Tr																				N	-12.16(31)	'CL/NADP-ME' (54, 56, 57)
Andropogon crossotos Cope	GH	SM	O-I-Tr																				Ne		'CL/NADP-ME' (54, 56, 57)
Andropogon distachyos L.	GH	M, SS	MS							N	N		N	N			N	N	N	N			N	-12.0(32)	'CL/NADP-ME' (54, 56, 57)
Andropogon greenwayi Napper	GH	SM	O-I-Tr																				N		'CL/NADP-ME' (54, 56, 57)
Apluda mutica L.	GH	PL	MS	N					N					N	N								N	-11.18 (*)	'CL/NADP-ME' (54, 56, 57)
Arthraxon cuspidatus (Hochst. ex A. Rich.) Hochst. ex Hack.	T	SM	MS-I-Tr											N										-9.63 (*)	'CL/NADP-ME' (54, 56, 57)
Arthraxon hispidus (Thunb.) Makino (incl. *A. micans* (Nees) Hochst.)	T	PL	MS-I-Tr	N	N			N						N						N			N	-15.93 (+)²-10.45(*): -11.69 (*)	'CL/NADP-ME' (54, 56, 57)
Arthraxon junnarensis S.K.Jain & Hemadri	T	SS, IC	MS-I-Tr											N											'CL/NADP-ME' (54, 56, 57)
Arthraxon lancifolius (Trin.) Hochst.	T	PL	MS-I-Tr	N										N	N								N	-9.64 (*)	'CL/NADP-ME' (54, 56, 57)
Arthraxon nudus (Steud.) Hochst.	T	PL	MS-I-Tr											N	N									-8.58 (*)	'CL/NADP-ME' (54, 56, 57)
Arthraxon prionodes (Steud.) Dandy	GH	PL	MS-I	N										N	N		N						N	-12.01 (*)	'CL/NADP-ME' (54, 56, 57)
Bothriochloa bladhii (Retz.) S.T.Blake	GH	PL	MS-I-Tr	N	N	N		N						N	N					N				-12.43 (+)	'CL/NADP-ME' (54, 56, 57)
Bothriochloa insculpta (Hochst. ex A. Rich.) A. Camus	GH	PL	MS-Tr											N			N						N	-11.76 (31)	'CL/NADP-ME' (54, 56, 57)
Bothriochloa ischaemum (L.) Keng	GH	PL	XE, O-I-Tm	N	N	N		N	N				N		N				N	N	N			-12.80 (+)	'CL/NADP-ME' (54, 56, 57)
Bothriochloa pertusa (L .) A. Camus	GH	PL	MS-I-Tr	N											N										'CL/NADP-ME' (54, 56, 57)
Bothriochloa radicans (Lehm.) A. Camus	GH	PL	O-I-Tr											N			N						N	-13.85(31)	'CL/NADP-ME' (54, 56, 57)
Bothriochloa saccharoides (Sw.) Rydb.	GH	PL	R							Ii															'CL/NADP-ME' (54, 56, 57)
Capillipedium assimile (Steud.) A. Camus	GH	PL	MS-I-Tr												N										'CL/NADP-ME' (54, 56, 57)
Capillipedium parviflorum (R. Br.) Stapf	GH	PL	MS-I-Tr											N	N									-13.6(30)	'CL/NADP-ME' (54, 56, 57)
Chrysopogon aucheri (Boiss.) Stapf	GH	SS, SM	XE, O-I-Tr	N				N						N	N		N						N	-12.38 (+)	'CL/NADP-ME' (54, 56, 57)
Chrysopogon fulvus (Spreng.) Chiov.	GH	I, IC	XE-I=Tr												N									-10.56 (*)	'CL/NADP-ME' (54, 56, 57)
Chrysopogon gryllus (L.) Trin.	GH	PL	O	N	N	N		N	N	N	N		N		N	N			N	N				-11.35 (*)	'CL/NADP-ME' (54, 56, 57)
Chrysopogon macleishii Cope	GH	SM	MS-I-Tr											Ne											'CL/NADP-ME' (54, 56, 57)
Chrysopogon plumulosus Hochst.	GH	PL	XE, O-I-Tr											N		N	N						N	-11.69 (+)	'CL/NADP-ME' (54, 56, 57)
Chrysopogon serrulatus Trin.	GH	PL	XE-I-Tr												N								N	-12.06 (*)	'CL/NADP-ME' (54, 56, 57)
Chrysopogon zizanioides (L.) Roberty	GH	IC	MS-Tr												N										'CL/NADP-ME' (54, 56, 57)
Cleistachne sorghoides Benth.	T	PL	MS-I-Tr											N										-10.75 (*)	'CL/NADP-ME' (54, 56, 57)
Coix aquatica Roxb.	GH	I, IC	HG-Tr	N																				-9.85 (*)	'CL/NADP-ME' (54, 56, 57)

				Distribution in Southwest Asia																					
C_4 lineage **(bold)** and species	Life form	Choro-type	Ecotype	Afghanistan	Armenia	Azerbaijan	Bahrain	Iran	Iraq	Israel/Palestine	Jordan	Kuwait	Lebanon	Oman	Pakistan	Qatar	Saudi Arabia	Sinai	Syria	Turkey	Turkmenistan	UAE	Yemen	δ^{13}C	Leaf anatomy, C_4 Photosynthetic subtype (references)
Cymbopogon caesius (Nees) Stapf	GH	PL	MS-I-Tr												N								N	-12.9(30)	'CL/NADP-ME' (54, 56, 57)
Cymbopogon commutatus (Steud.) Stapf	GH	SS, SM	XE	N			N	N	N	N	N			N	N	N	N						N	-11.77 (+)	'CL/NADP-ME' (54, 56, 57)
Cymbopogon distans (Nees) Watson	GH	CA, SJ	XE, O-I-Tm												N										'CL/NADP-ME' (54, 56, 57)
Cymbopogon iwarancusa (Jones ex Roxb.) Schult.	GH	PL	XE-I	N			N	N	N				N	N	N				N			N	N		'CL/NADP-ME' (54, 56, 57)
Cymbopogon martini (Roxb.) Wats.	GH	I, IC	MS-Tr												N										'CL/NADP-ME' (54, 56, 57)
Cymbopogon nervatus (Hochst.) Chiov	T or GH	PL	XE-I-Tr														N								'CL/NADP-ME' (54, 56, 57)
Cymbopogon pospischilii (K. Schum.) C.E. Hubbard	GH	PL	O-I-Tm	N										N	N								N		'CL/NADP-ME' (54, 56, 57)
Cymbopogon schoenanthus (L.) Spreng.	GH	SS	XE-Tr				N			N	N			N			N		N			N	N	-12.0(32)	'CL/NADP-ME' (54, 56, 57)
Dichanthium annulatum (Forssk.) Stapf	GH	PL	MS, R	N			N	N	N	N	N	N		N	N	N	N	N		N		N	N	-13.67(31)	'CL/NADP-ME' (54, 56, 57)
Dichanthium aristatum (Poir.) C.E. Hubb.	GH	I, IC, SJ	MS-I-Tr											N									N		'CL/NADP-ME' (54, 56, 57)
Dichanthium caricosum (L.) A. Camus	GH	I, IC, SJ	MS, R-Tr														N								'CL/NADP-ME' (54, 56, 57)
Dichanthium foveolatum (Delile) Roberty	GH	PL	MS-Tr				N	N		N	N	N		N	N	N	N	N				N	N	-10.93 (+)	'CL/NADP-ME' (54, 56, 57)
Dichanthium micranthum Cope	GH	SM	MS											Ne											'CL/NADP-ME' (54, 56, 57)
Diectomis fastigiata (Sw.) P.Beauv.	T	PL	O-I-Tr																				N		'CL/NADP-ME' (54, 56, 57)
Dimeria ornithopoda Trin.	T	PL	MS-Tr											N										-10.45 (*)	'CL/NADP-ME' (54, 56, 57)
Elionurus muticus (Spreng.) Kuntze	GH	PL	O-I-Tr																				N	-11.59(31)	'CL/NADP-ME' (54, 56, 57)
Elionurus royleanus Nees ex A. Rich	T	PL	XE-Tr					N	N					N	N		N						N		'CL/NADP-ME' (54, 56, 57)
Euclasta clarkei (Hack.) Cope	T	SM, I, IC	MS-I-Tr											N											'CL/NADP-ME' (54, 56, 57)
Eulaliopsis binata (Retz.) C.E. Hubbard	GH	PL	MS-I	N											N									-9.16 (*)	'CL/NADP-ME' (54, 56, 57)
Hackelochloa granularis (L.) Kuntze	T	PL	MS-Tr											N									N	-10.92 (+), -10.37 (*)	'CL/NADP-ME' (54, 56, 57)
Hemarthria altissima (Poir.) Stapf & C.E. Hubb.	GH	PL	HG-Tr							N	N		N				N		N	N				-12.43(31)	'CL/NADP-ME' (54, 56, 57)
Hemarthria compressa (L.f.) R.Br.	GH	PL	HG-I-Tr	N					N						N										'CL/NADP-ME' (54, 56, 57)
Hemarthria sibirica (Gand.) Ohwi	GH	CA, SJ	HG-Tm												N										'CL/NADP-ME' (54, 56, 57)
Heteropogon contortus (L.) P. Beauv. ex Roem. & Schult.	GH	PL	MS-I-Tr	N				N	N				N	N	N		N						N	-12.93 (+)	'CL/NADP-ME' (54, 56, 57)
Heteropogon melanocarpus (Elliott) Benth.	T	PL	MS-I-Tr											N										-12.62(31)	'CL/NADP-ME' (54, 56, 57)
Hyparrhenia coleotricha (Steud.) Andersson ex Clayton	T	SM	O-I-Tr																				N		'CL/NADP-ME' (54, 56, 57)
Hyparrhenia dregeana (Nees) Stapf ex Stent	GH	PL	MS, O-I-Tr																				N		'CL/NADP-ME' (54, 56, 57)
Hyparrhenia formosa Stapf	GH	GC, ZI, SM	MS, O-I-Tr																				N		'CL/NADP-ME' (54, 56, 57)
Hyparrhenia hirta Stapf	GH	PL	XE-Tr	N			N	N	N	N	N		N	N	N		N	N	N	N			N	-13.03(31)	'CL/NADP-ME' (54, 56, 57)
Hyparrhenia papillipes (Hochsl. exA. Rich.) Andersson ex Stapf	GH	PL	XE, O-I-Tr																				N		'CL/NADP-ME' (54, 56, 57)
Hyparrhenia quarrei Robyns	GH	PL	MS, O-I-Tr																				N	-12.39 (*)	'CL/NADP-ME' (54, 56, 57)

C$_4$ lineage **(bold)** and species	Life form	Choro-type	Ecotype	Distribution in Southwest Asia																				$\delta^{13}C$	Leaf anatomy, C$_4$ Photosynthetic subtype (references)
				Afghanistan	Armenia	Azerbaijan	Bahrain	Iran	Iraq	Israel/Palestine	Jordan	Kuwait	Lebanon	Oman	Pakistan	Qatar	Saudi Arabia	Sinai	Syria	Turkey	Turkmenistan	UAE	Yemen		
Hyparrhenia variabilis Stapf	GH	PL	MS-I-Tr																				N		'CL/NADP-ME' (54, 56, 57)
Imperata cylindrica (L.) P.Beauv.	GH	PL	MS, R	N	N	N		N	N	N	N	N	N	N	N		N	N	N	N	N		N	-13.51 (+)	'CL/NADP-ME' (54, 56, 57)
Ischaemum impressum Hack.	T	SM, SS	MS-Tr																				N		'CL/NADP-ME' (54, 56, 57)
Ischaemum molle Hook. f.	GH	I, IC	HG-Tr												N		N							-10.96 (*)	'CL/NADP-ME' (54, 56, 57)
Ischaemum rugosum Salisb.	T	PL	MS, R-Tr												N									-9.82 (*)	'CL/NADP-ME' (54, 56, 57)
Iseilema prostratum (L.) Andersson	GH	I, IC	HG-Tr												N									-9.94 (*)	'CL/NADP-ME' (54, 56, 57)
Lasiurus scindicus Henr.	GH	SS, SU	XE-Tr	N				N	N	N	N	N		N	N	N	N					N	N	-11.72 (+)	'CL/NADP-ME' (54, 56, 57)
Microstegium fasciculatum (L.) Henrard	GH	PL	MS-Tr												N									-10.02 (*)	'CL/NADP-ME' (54, 56, 57)
Microstegium nudum (Trin.) A. Camus	T	PL	MS-Tr												N									-14.42 (*)	'CL/NADP-ME' (54, 56, 57)
Microstegium vimineum (Trin.) A. Camus	T	PL	MS			N		N																-9.08 (*)	'CL/NADP-ME' (54, 56, 57)
Miscanthus nepalensis (Trin.) Hack.	GH	PL	MS-Tr	N																				-10.01 (*)	'CL/NADP-ME' (54, 56, 57)
Mnesithea laevis (Retz.) Kunth	GH	PL	PS-L-Tr	N											N									-10.41 (*)	'CL/NADP-ME' (54, 56, 57)
Narenga porphyrocoma (Hance) Bor	GH	PL	HG-I-Tr												N									-11.34 (*)	'CL/NADP-ME' (54, 56, 57)
Ophiuros exaltatus (L.) Kuntze	T	PL	MS-Tr												N									-10.28 (*)	'CL/NADP-ME' (54, 56, 57)
Phacelurus digitatus (Sm.) Griseb.	GH	M	MS-L-Tm							N	N		N						N	N				-9.98 (*)	'CL/NADP-ME' (54, 56, 57)
Phacelurus speciosus (Steud.) C. E. Hubbard	GH	CA, SS	O-I-Tm	N											N									-11.48 (*)	'CL/NADP-ME' (54, 56, 57)
Pogonatherum crinitum (Thunb.) Kunth	GH	IC, I	MS-I-Tr												N									-12.2(30)	'CL/NADP-ME' (54, 56, 57)
Pogonatherum paniceum (Lam.) Hack.	GH	PL	O-I-Tr	N											N		N							-10.81 (*)	'CL/NADP-ME' (54, 56, 57)
Polytoca gigantea (J.Koenig) Mabb.	GH	I, IC	MS-I-Tr												N									-10.03 (*)	'CL/NADP-ME' (54, 56, 57)
Pseudodichanthium serrafalcoides (T. Cooke & Stapf) Bor	T	SM, I	MS-Tr											N											'CL/NADP-ME' (54, 56, 57)
Pseudopogonatherum trispicatum (Schult.) Ohwi	GH	PL	MS-Tr												N									-10.46 (*)	'CL/NADP-ME' (54, 56, 57)
Rottboellia cochinchinensis (Lour.) Clayton	T	PL	MS-I-Tr											N									N	-12.65(+)	'CL/NADP-ME' (54, 56, 57)
Saccharum filifolium Nees ex Steud.	GH	IT, SJ	O-I-Tm	N											N									-12.38 (*)	'CL/NADP-ME' (54, 56, 57)
Saccharum griffithii Munro ex Boiss.	GH	SS, I	HG-I-Tr	N				N						N	N		N					N	N		'CL/NADP-ME' (54, 56, 57)
Saccharum kajkaiense (Melderis) Melderis	GH	SS	MS-Tr	Ne				Ne						Ne	Ne										'CL/NADP-ME' (54, 56, 57)
Saccharum rufipilum Steud.	GH	I, SJ, IC	HG-I												N										'CL/NADP-ME' (54, 56, 57)
Saccharum spontaneum L.	GH	PL	HG-I	N				N		N	N		N		N		N	N	N		N		N	-11.7 (24), -14.12 (*)	'CL/NADP-ME' (54, 56, 57)
Schizachyrium brevifolium (Sw.) Nees ex Buse	T	PL	MS-Tr											N										-12.2(*)	'CL/NADP-ME' (54, 56, 57)
Schizachyrium impressum (Hack.) A. Calms	GH	IT, CA	MS-I-Tm												N									- 12.28(2)	'CL/NADP-ME' (54, 56, 57)
Schizachyrium scoparium (Michx.) Nash	GH	NA	MS-I-Tm												I									-13.12 (*)	'CL/NADP-ME' (54, 56, 57)
Sehima ischaemoides Forssk.	T	PL	MS-I-Tr											N	N								N	-12.45(31)	'CL/NADP-ME' (54, 56, 57)
Sehima nervosum (Rottl.) Stapf	GH	PL	MS-I-Tr												N		N						N	-9.80(+), -11.53 (*)	'CL/NADP-ME' (54, 56, 57)
Sorghum arundinaceum (Desv.) Stapf	T or H	PL	MS-I-Tr												N										'CL/NADP-ME' (54, 56, 57)

C_4 lineage **(bold)** and species	Life form	Chorotype	Ecotype	Distribution in Southwest Asia																				δ13C	Leaf anatomy, C_4 Photosynthetic subtype (references)
				Afghanistan	Armenia	Azerbaijan	Bahrain	Iran	Iraq	Israel/Palestine	Jordan	Kuwait	Lebanon	Oman	Pakistan	Qatar	Saudi Arabia	Sinai	Syria	Turkey	Turkmenistan	UAE	Yemen		
Sorghum halepense (L.) Pers. (incl. *S. miliaceum* (Roxb.) Snowden)	GH	PL	R, MS	N	N	N		N	N	N	N	N	N	N	N	N	N	N	N	N	N	N		-13.14 (+)	'CL/NADP-ME' (54, 56, 57)
Sorghum nitidum (Vahl) Pers.	GH	PL	MS-Tr												N									-12.35(*)	'CL/NADP-ME' (54, 56, 57)
Sorghum purpureosericeum (Hochst. ex A. Rich.) Asch. & Schweinf. (incl. *S. deccanense* Stapf ex Bor)	T	I, SM, SU	MS-I-Tr											N									N		'CL/NADP-ME' (54, 56, 57)
Sorghum virgatum (Hack.) Stapf	T or H	SS, SU	MS-I-Tr							N	N		N				N	N	N						'CL/NADP-ME' (54, 56, 57)
Spodiopogon cotulifer (Thunb.) Hack.	GH	SJ	MS-Tm												N										'CL/NADP-ME' (54, 56, 57)
Spodiopogon pogonanthus (Boiss. & Balansa) Boiss.	GH	M, It	MS-Tm						N	N	N		N						N	N				-10.22 (*)	'CL/NADP-ME' (54, 56, 57)
Thelepogon elegans Roth ex Roem. & Schult.	T	PL	HG, R-Tr												N									-10.22 (*)	'CL/NADP-ME' (54, 56, 57)
Themeda anathera (Nees ex Steud.) Hack.	GH	CA, SS, SJ	MS-I-Tm	N											N										'CL/NADP-ME' (54, 56, 57)
Themeda quadrivalvis (L.) Kuntze	T	I, IC	MS-I-Tr						N					N			N			N					'CL/NADP-ME' (54, 56, 57)
Themeda triandra Forssk.	GH	PL	MS-I-Tr										N				N		N	N			N	-11.59(31)	'CL/NADP-ME' (54, 56, 57)
Tripidium bengalense (Retz.) H.Scholz	GH	SS, I	HG, MS-I-Tr	N				N					N		N				N						'CL/NADP-ME' (54, 56, 57)
Tripidium ravennae (L.) H.Scholz	GH	PL	HG, MS	N	N	N		N	N	N	N		N	N	N		N		N	N	N	N	N	-14.79, -14.12, -14.31 (*)	'CL/NADP-ME' (54, 56, 57)
Tripidium strictum (Host) H.Scholz	GH	M, IT	MS-I-Tm						N	N	N		N						N	N					'CL/NADP-ME' (54, 56, 57)
**8) *Aristida***																									
Aristida abnormis Chiov.	T	SS, SM	XE, O-Tr				N							N	N	N	N					N	N	-13.99 (*)	'AR(55)/NADP-ME'(54, 56, 57)
Aristida adscensionis L. (incl. *A. heymannii* Regel and *A. coerulescens* Desf.)	T	PL	XE	N	N	N		N	N	N	N		N	N	N	N	N	N	N	N	N	N	N	-12.1 (*), -15.5[3](32)	'AR(55)/NADP-ME'(54, 56, 57)
Aristida anaclasta Cope	GH	SM	XE, O-Tr																				Ne		'AR(55)/NADP-ME'(54, 56, 57)
Aristida congesta Roem. & Schult.	GH	PL	O, R-Tr														N						N	-12.90(+)	'AR(55)/NADP-ME'(54, 56, 57)
Aristida cyanantha Nees ex Steud.	GH	SS, CA	XE, O	N				N							N										'AR(55)/NADP-ME'(54, 56, 57)
Aristida ferrilateris S.M.Phillips	GH	SM	XE, O-Tr														N						N		'AR(55)/NADP-ME'(54, 56, 57)
Aristida fredscholzii H.Scholz & Kurschner	T	SM	XE											Ne											'AR(55)/NADP-ME'(54, 56, 57)
Aristida funiculata Trin. & Rupr. (including *A. royleana* Trin. & Rupr.)	T	SS, SM	XE, PS-Tr	N										N	N		N					N	N	-12.7(2)	'AR(55)/NADP-ME'(54, 56, 57)
Aristida hystricula Edgew.	T	SS	PS-Tr												Ne										'AR(55)/NADP-ME'(54, 56, 57)
Aristida migiurtina Chiov.	GH	SM	O, GP-Tr											N			N						N		'AR(55)/NADP-ME'(54, 56, 57)
Aristida mutabilis Trin. & Rupr.	T	SS, SM	XE, R-Tr											N	N	N	N					N	N		'AR(55)/NADP-ME'(54, 56, 57)
Aristida pennei Chiov.	GH	SM	O, R-Tr														N						N		'AR(55)/NADP-ME'(54, 56, 57)
Aristida sieberiana Trin.	GH	SM	PS-Tr							N	N							N					N		'AR(55)/NADP-ME'(54, 56, 57)
Aristida tricornis H.Scholz & P.Koenig	GH	SM	XE, O-Tr														Ne						Ne		'AR(55)/NADP-ME'(54, 56, 57)
Aristida triticoides Henrard	GH	SS, SM	XE, PS-Tr											N	N								N		'AR(55)/NADP-ME'(54, 56, 57)

C_4 lineage **(bold)** and species	Life form	Choro-type	Ecotype	Distribution in Southwest Asia																				$\delta^{13}C$	Leaf anatomy, C_4 Photosynthetic subtype (references)
				Afghanistan	Armenia	Azerbaijan	Bahrain	Iran	Iraq	Israel/Palestine	Jordan	Kuwait	Lebanon	Oman	Pakistan	Qatar	Saudi Arabia	Sinai	Syria	Turkey	Turkmenistan	UAE	Yemen		
**9) *Centropodia***																									
Centropodia forskalii (Vahl) Cope	GH	IT, SS	PS-Tr	N			N	N	N	N	N	N		N		N	N	N				N	N	-13.92 (+)	'CL/NAD-ME' (57)
Centropodia fragilis (P.Guinet & Sauvage) Cope	GH	SS	PS-Tr														N					N	N		'CL/NAD-ME' (57)
**10) *Core Chloridoideae***																									
Acrachne racemosa (Heyne ex Roem. & Schult) Ohwi	GH	PL	XE-Tr	N										N	N		N						N	-13.25 (31)	'CL/NAD-ME(or PEP-CK)' (57)
Aeluropus badghyzi Tzevelev	GH	IT	XE																		Ne				'CL/NAD-ME(or PEP-CK)' (57)
Aeluropus laciniatus Khodashenas	GH	IT	HL					Ne																	'CL/NAD-ME(or PEP-CK)' (57)
Aeluropus lagopoides (L.) Trin. ex Thwaites (incl. *A. brevifolius* (Willd.) Steud.)	GH	PL	HL	N	N	N	N		N	N	N	N	N	N	N	N	N	N	N	N	N	N	N	-13.32(2)	'CL/NAD-ME(or PEP-CK)' (57)
Aeluropus littoralis (Gouan) Parl. (incluing *A. pungens* (M.Bieb.) Koch)	GH	PL	HL	N	N	N	N	N	N	N	N	N			N		N	N	N	N	N	N		-14.73 (+)	'CL/NAD-ME(or PEP-CK)' (57)
Aeluropus macrostachyus Hack. (incl. *A. peterganicus* Khodashenas)	GH	SS	XE-Tr	Ne				Ne							Ne										'CL/NAD-ME(or PEP-CK)' (57)
Chloris barbata Sw.	GH	PL	R				N			N	N	N		N	N	N	N					N	N	-13.60 (*)	'CL/NAD-ME(or PEP-CK)' (57)
Chloris flagellifera (Nees) P.M.Peterson	GH	SS,SM	PH-Tr	N			N	N						N	N	N	N					N	N	-13.30 (*), -15.09 (*)	'CL/NAD-ME(or PEP-CK)' (57)
Chloris gayana Kunth.	GH	PL	R-Tr				I		I	I	I			I	I		I					I		-12.2(33)	CL/NAD-ME or PEP-CK (58)
Chloris mensensis (Schweinf.) Cuf.	GH	SM	MS-I-Tr																				N		'CL/NAD-ME(or PEP-CK)' (57)
Chloris pycnothrix Trin.	T	PL	MS							N	N												N	-14.33(31)	'CL/NAD-ME(or PEP-CK)' (57)
Chloris quinquesetica Bhide	GH	SS	HL-L-Tr											N	N										'CL/NAD-ME(or PEP-CK)' (57)
Chloris virgata Sw.	T	PL	R, HL	Ii		Ii		Ii	Ii	Ii	Ii			Ii	Ii	Ii	Ii				Ii	Ii	Ii	-14.56(31)	'CL/NAD-ME(or PEP-CK)' (57)
Cleistogenes gatacrei (Stapf) Bor	GH	IT	XE, O-Tm	Ne											Ne										'CL/NAD-ME(or PEP-CK)' (57)
Cleistogenes serotina (L.) Keng	GH	PL	XE, O		N	N		N												N	N			-14.31 (+)	'CL/NAD-ME(or PEP-CK)' (57)
Cleistogenes songorica (Roshev.) Ohwi	GH	IT, CA	PS-I-Tm																		N				'CL/NAD-ME(or PEP-CK)' (57)
Coelachyrum piercei (Benth.) Bor	GH	SM, SS	PS-Tr					N						N	N		N					N	N		'CL/NAD-ME(or PEP-CK)' (57)
Coelachyrum brevifolium Hochst. & Nees	T	SS, SM	PS-Tr											N			N					N	N	-12.42 (+)	'CL/NAD-ME(or PEP-CK)' (57)
Coelachyrum poiflorum Chiov.	GH	SM	XE, R-Tr											N			N						N		'CL/NAD-ME(or PEP-CK)' (57)
Ctenium elegans Kunth.	T	PL	PS-Tr														N							-11.97 (*)	'CL/NAD-ME(or PEP-CK)' (57)
Cynodon dactylon (L.) Pers.	GH	PL	R	N	N	N	N	N	N	N	N	N	N	N	N	N	N	N	N	N	N	N	N	-13.87 (+)	CL/NAD-ME (58)
Cynodon nlemfuensis Vanderyst	GH	PL	R, MS-Tr														Ii								'CL/NAD-ME' (58)
Cynodon radiatus Roth	GH	PL	MS												N										'CL/NAD-ME' (58)
Cynodon transvaalensis Burtt Davy	GH	Z	MS, HG, R					I																	'CL/NAD-ME' (58)
Dactyloctenium aegyptium (L.) Willd.	T	PL	PS, R-Tr	N			N	N	N	N	N	N	N	N	N	N	N	N	N	N		N	N	-11.32(31)	'CL/NAD-ME' (58)
Dactyloctenium aristatum Link	T	SM	PS, R-Tr									N		N	N		N						N		'CL/NAD-ME' (58)
Dactyloctenium hackelii M. Wagner & Vierh.	GH	SM	PH-L-Tr																				Ne		'CL/NAD-ME' (58)

C_4 lineage **(bold)** and species	Life form	Choro-type	Ecotype	Distribution in Southwest Asia																				δ^{13}C	Leaf anatomy, C_4 Photosynthetic subtype (references)
				Afghanistan	Armenia	Azerbaijan	Bahrain	Iran	Iraq	Israel/Palestine	Jordan	Kuwait	Lebanon	Oman	Pakistan	Qatar	Saudi Arabia	Sinai	Syria	Turkey	Turkmenistan	UAE	Yemen		
Dactyloctenium robecchii (Chiov.) Chiov.	GH	SM	O-Tr											N									N		'CL/NAD-ME' (58)
Dactyloctenium scindicum Boiss.	GH	SM, SS	PS, R-Tr	N				N						N	N		N					N	N		'CL/NAD-ME' (58)
Desmostachya bipinnata (L.) Stapf	GH	PL	XE-Tr	N				N	N	N	N			N	N		N	N				N	N	-11.75(2)	'CL/NAD-ME(or PEP-CK)' (57)
Dignathia hirtella Stapf	T	SM, SS, IC	O-TR											N									N	-13.31(2)	'CL/NAD-ME(or PEP-CK)' (57)
Dinebra retroflexa (Vahl) Panz.	T	PL	MS, R-Tr	N				N	N	N	N	N		N	N		N						N	-12.92(31)	'CL/NAD-ME(or PEP-CK)' (57)
Dinebra somalensis (Stapf) P.M.Peterson & N.Snow	T	SM	HL-Tr														N								'CL/NAD-ME(or PEP-CK)' (57)
Diplachne fusca (L.) P.Beauv. ex Roem. & Schult. (incl. *Leptochloa uninervia* (J.Presl) Hitchc. & Chas)	GH	PL	HL					N		N	N	N			N	N	N					N	N		'CL/NAD-ME(or PEP-CK)' (57)
Disakisperma obtusiflorum (Hochst.) P.M.Peterson & N.Snow	GH	SM, Z	XE-Tr														N						N		'CL/NAD-ME(or PEP-CK)' (57)
Disakisperma yemenicum (Schweinf.) P.M.Peterson & N.Snow	GH	SM	O-Tr											N			N						N	-12.95(31)	'CL/NAD-ME(or PEP-CK)' (57)
Eleusine africana Kenn.-O'Byrne	T	PL	R-Tr											N			N						N		'CL/NAD-ME(or PEP-CK)' (57)
Eleusine coracana (L.) Gaertn.	T	SM, SU, GC	R-Tr	I	I		I							I	I		I						I	-12.2(33)	'CL/NAD-ME(or PEP-CK)' (57)
Eleusine floccifolia (Forssk.) Spreng	GH	SM	MS-I-Tr																				N	-10.33 (*)	'CL/NAD-ME(or PEP-CK)' (57)
Eleusine indica (L.) Gaertn.	T	PL	R	N	N	N		N		N	N			N	N		N	N		N	N			-16.27(31)	CL/NAD-ME (59)
Eleusine multiflora Hochst.	T	SM	R-I-Tr														N						N		'CL/NAD-ME(or PEP-CK)' (57)
Enneapogon cenchroides (Roem. & Schult.) C.E. Hubbard	T	PL	O-Tr											N	N		N						N	-14.95(31)	'CL/NAD-ME(or PEP-CK)' (57)
Enneapogon desvauxii P.Beauv	GH	PL	XE					N		N	N			N	N		N					N	N	-14.73(+)	'CL/NAD-ME(or PEP-CK)' (57)
Enneapogon foxii (Post) Valdйs & H. Scholz	GH	SS	XE-Tr							Ne	Ne														'CL/NAD-ME(or PEP-CK)' (57)
Enneapogon lophotrichus Chiov. ex H.Scholz & P Koenig	T	SM, SU	O-Tr											N			N						N		'CL/NAD-ME(or PEP-CK)' (57)
Enneapogon persicus Boiss. (incl. *E. schimperanus* (Hochst. ex A. Rich.) Renvoize)	GH	PL	O	N	N	N		N	N	N	N			N	N		N				N	N	N	-14.36 (*)	'CL/NAD-ME(or PEP-CK)' (57)
Enneapogon scaber Lehm.	GH	PL	O-Tr														N							-15.3(32)	'CL/NAD-ME(or PEP-CK)' (57)
Enneapogon scoparius Stapf	GH	PL	O-Tr																				N	-13.45(31)	'CL/NAD-ME(or PEP-CK)' (57)
Enteropogon dolichostachyus (Lag.) Keng ex Lazarides	GH	PL	XE-Tr											N	N								N		'CL/NAD-ME' (58)
Enteropogon macrostachyus (Hochst. Ex A.Rich) Munro ex Benth.	GH	PL	O-I-Tr														N						N	-13.60(31)	CL/NAD-ME (58)
Enteropogon prieurii (Kunth) Clayton	T	PL	XE-I-Tr														N							-11.24(31)	'CL/NAD-ME' (58)
Eragrostis aegyptiaca (Willd.) Delile	T	SS	XE-Tr														N								'CL/NAD-ME(or PEP-CK)' (57)
Eragrostis aethiopica Chiov.	T	PL	R-Tr														N						N		'CL/NAD-ME(or PEP-CK)' (57)
Eragrostis aspera (Jacq.) Nees	T	PL	O, R-Tr											N			N						N	-12.41(31)	CL/NAD-ME (57, 58)

				Distribution in Southwest Asia																					
C_4 lineage **(bold)** and species	Life form	Choro-type	Ecotype	Afghanistan	Armenia	Azerbaijan	Bahrain	Iran	Iraq	Israel/Palestine	Jordan	Kuwait	Lebanon	Oman	Pakistan	Qatar	Saudi Arabia	Sinai	Syria	Turkey	Turkmenistan	UAE	Yemen	δ^{13}C	Leaf anatomy, C_4 Photosynthetic subtype (references)
Eragrostis atrovirens (Desf.) Trin. ex Steud.	GH	PL	R-Tr												N										'CL/NAD-ME(or PEP-CK)' (57)
Eragrostis barrelieri Daveau	T	PL	R	N	N			N	N	N	N	N		N	N	N	N			N		N	N	-12.7 (+), -13.9 (24)	'CL/NAD-ME(or PEP-CK)' (57)
Eragrostis boriana Launert	T	SS	HG-Tr						N																'CL/NAD-ME(or PEP-CK)' (57)
Eragrostis braunii Schweinf.	GH	SM	O-I-Tr														N						N		'CL/NAD-ME(or PEP-CK)' (57)
Eragrostis cilianensis (All.) Janch.	T	PL	XE	N	N	N		N	N	N	N		N	N	N	N	N	N	N	N	N	N	N	-15.4 (34)	CL/NAD-ME (57.58)
Eragrostis ciliaris (L.) R.Br.	T	PL	XE-Tr												N									-15.38 (+)	'CL/NAD-ME(or PEP-CK)' (57)
Eragrostis collina Trin.	GH	IT	XH-I-Tm		N	N		N	N										N	N	N			-12.09 (*)	'CL/NAD-ME(or PEP-CK)' (57)
Eragrostis curvula (Schrad.) Nees	GH	PL	O-Tr	N				N							N		N			N			N	-11.6(33)	CL/NAD-ME (59)
Eragrostis cylindriflora Hochst.	GH	PL	R-I-Tr																				N	-13.29 (+)	'CL/NAD-ME(or PEP-CK)' (57)
Eragrostis echinochloidea Stapf	T	Z	R-Tr							Ii	Ii													-12.61(31)	CL/NAD-ME (57, 58)
Eragrostis japonica (Thunb.) Trin.	T	PL	R					N	N	N	N			N	N		N	N				N	N		'CL/NAD-ME(or PEP-CK)' (57)
Eragrostis lehmanniana Nees	GH	PL	R-Tr	Ii											Ii									-13.71(31)	'CL/NAD-ME(or PEP-CK)' (57)
Eragrostis lepida (A. Rich.) Hochst. ex Steud.	T	SM	R-Tr														N						N		'CL/NAD-ME(or PEP-CK)' (57)
Eragrostis longifolia (A.Rich.) Hochst. ex Steud.	GH	SM	XE-Tr																				N		'CL/NAD-ME(or PEP-CK)' (57)
Eragrostis macilenta (A. Rich.) Steud.	T	PL	R-I-Tr																				N		'CL/NAD-ME(or PEP-CK)' (57)
Eragrostis mahrana Schweinf.	GH	SM	PS-L-Tr											N									N		'CL/NAD-ME(or PEP-CK)' (57)
Eragrostis minor Host	T	PL	XE, R	N	N	N		N	N	N	N	N	N	N	N		N	N	N	N	N	N	N	-13.87 (+)	'CL/NAD-ME(or PEP-CK)' (57)
Eragrostis nigra Nees ex Steud.	GH	PL	MS, R												N										'CL/NAD-ME(or PEP-CK)' (57)
Eragrostis palmeri S. Watson	T	MA	R							Ii	Ii														'CL/NAD-ME(or PEP-CK)' (57)
Eragrostis papposa (Roem. & Schult.) Steud.	GH	PL	XE-I-Tr	N										N	N		N						N		'CL/NAD-ME(or PEP-CK)' (57)
Eragrostis pilosa (L.) P. Beauv.	T	PL	XE, R	N	N	N		N	N	N	N		N	N	N	N	N		N	N	N	N	N	-15.0 (34)	CL/NAD-ME (57, 58)
Eragrostis prolifera (Sw.) Steud.	T	PL	R-Tr							Ii	Ii														'CL/NAD-ME(or PEP-CK)' (57)
Eragrostis sarmentosa (Thunb.) Trin.	T	PL	R-Tr							Ii	Ii													-11.7(31)	'CL/NAD-ME(or PEP-CK)' (57)
Eragrostis scbweinfurthii Chiov.	T or GH	PL	MS-I-Tr																				N		'CL/NAD-ME(or PEP-CK)' (57)
Eragrostis superba Peyr.	GH	PL	R-Tr												I		I							-12.47(31)	CL/NAD-ME (57.58)
Eragrostis tef (Zuccagni) Trotter	T	SM	R-I-Tr							N							N						N	-16.31(31)	'CL/NAD-ME(or PEP-CK)' (57)
Eragrostis tenella (L.) P. Beauv. ex Roem. & Schult.	T	PL	XE, PS-Tr	N				N														N	N	-11.73[4] (30)	'CL/NAD-ME(or PEP-CK)' (57)
Eragrostis tenuifolia (A. Rich.) Hochst. ex Steud.	GH	PL	O-I-Tr											N			N						N		'CL/NAD-ME(or PEP-CK)' (57)
Eragrostis tremula Hochst. ex Steud.	T	PL	HG-Tr	N											N									-11.74(2)	'CL/NAD-ME(or PEP-CK)' (57)
Eragrostis turgida (Schumach.) De Wild	T	SS, SM, SU	R-Tr														N								'CL/NAD-ME(or PEP-CK)' (57)
Eragrostis unioloides (Retz.) Nees ex Steud.	T or H	PL	MS-I-Tr											N	N									-13.29(2)	'CL/NAD-ME(or PEP-CK)' (57)
Eragrostis virescens J. Presl	T	PL	R-Tr							I	I														'CL/NAD-ME(or PEP-CK)' (57)
Eragrostis viscosa (Retz.) Trin	T	PL	XE-Tr											N	N		N							-13.94(31)	CL/NAD-ME (57.58)

C_4 lineage **(bold)** and species	Life form	Choro-type	Ecotype	Distribution in Southwest Asia																				δ13C	Leaf anatomy, C_4 Photosynthetic subtype (references)
				Afghanistan	Armenia	Azerbaijan	Bahrain	Iran	Iraq	Israel/Palestine	Jordan	Kuwait	Lebanon	Oman	Pakistan	Qatar	Saudi Arabia	Sinai	Syria	Turkey	Turkmenistan	UAE	Yemen		
Eustachys paspaloides (Vahl) Lanza & Mattei	GH	PL	MS-I-Tr											N									N	-12.56(31)	'CL/NAD-ME(or PEP-CK)' (57)
Fingerhuthia africana Lehm.	GH	PL	O-I-Tr	N										N	N		N						N	-13.21(31)	'CL/NAD-ME(or PEP-CK)' (57)
Halopyrum mucronatum (L.) Stapf	GH	PL	PH				N	N				N		N	N		N					N	N	-13.42 (+)	'CL/NAD-ME(or PEP-CK)' (57)
Harpachne schimperi Hochst. ex A. Rich.	GH	PL	R-I-Tr														N						N	-13.27 (+), -11.23 (*)	'CL/NAD-ME(or PEP-CK)' (57)
Leptocarydion vulpiastrum (De Not.) Stapf	T	PL	HG, R-I-Tr																				N	-13.34(31)	'CL/NAD-ME(or PEP-CK)' (57)
Leptochloa mucronata (Michx.) Kunth	T	PL	R-Tr							Ii	Ii														'CL/NAD-ME(or PEP-CK)' (57)
Leptochloa panicea (Retz.) Ohwi	T	PL	R-Tr	Ii						Ii	Ii				Ii		Ii								'CL/NAD-ME(or PEP-CK)' (57)
Leptothrium senegalense (Kunth) W.D. Clayton	GH	SS, SM, SU	XE-Tr											N	N		N						N	-13.73 (+)	'CL/NAD-ME(or PEP-CK)' (57)
Lepturus calcareus Cope	GH	SM	XE-I-Tr																				Ne		'CL/NAD-ME(or PEP-CK)' (57)
Lepturus nesiotes Cope	GH	SM	XE-I-Tr																				Ne		'CL/NAD-ME(or PEP-CK)' (57)
Lepturus pulchellus (Balf.f.) Clayton	GH	SM	PS-L-Tr																				Ne		'CL/NAD-ME(or PEP-CK)' (57)
Lepturus tenuis Balf.f.	GH	SM	XE-I-Tr																				Ne		'CL/NAD-ME(or PEP-CK)' (57)
Melanocenchris abyssinica (R. Br. ex Fresen.) Hochst.	T	SS, SM	O, R-I-Tr					N						N	N		N						N	-11.85 (+)	'CL/NAD-ME(or PEP-CK)' (57)
Melanocenchris jacquemontii Jaub. & Spach	T	SS, I	XE-Tr												N								N		'CL/NAD-ME(or PEP-CK)' (57)
Microchloa kunthii Desv.	GH	PL	XE-I-Tr											N			N						N	-12.26(31)	'CL/NAD-ME(or PEP-CK)' (57)
Muhlenbergia duthieana Hack.	GH	IT, CA	O, MS-I-Tm	N											N										'CL/NAD-ME(or PEP-CK)' (57)
Muhlenbergia himalayensis Hack. ex Hook.	GH	IT, CA, SJ	O, MS-I-Tm	N											N										'CL/NAD-ME(or PEP-CK)' (57)
Muhlenbergia huegelii Trin.	GH	PL	MS-I	N											N									-13.40 (*)	'CL/NAD-ME(or PEP-CK)' (57)
Neyraudia arundinacea (L.) Henr.	GH	PL	MS-I-Tr	N											N										'CL/NAD-ME(or PEP-CK)' (57)
Orinus thoroldii (Stapf. ex Hems].) Bor	GH	IT, CA	PS-I-Tm												N										'CL/NAD-ME(or PEP-CK)' (57)
Oropetium capense Stapf	GH	PL	O-I-Tr											N			N						N	-13.03(31)	'CL/NAD-ME(or PEP-CK)' (57)
Oropetium minimum (Hochst.) Pilg.	GH	SM	O-I-Tr														N						N		'CL/NAD-ME(or PEP-CK)' (57)
Oropetium thomaeum (L.f.) Trin.	GH	PL	XE-Tr														N							- 12.28(2)	'CL/NAD-ME(or PEP-CK)' (57)
Schmidtia pappophoroides Steud.	T	PL	MS, R-Tr																				N	-12.57(31)	CL/NAD-ME or PEP-CK (58)
Schoenefeldia gracilis Kunth	T	SS, SM, I	XE-Tr														N						N	-12.99 (+)	'CL/NAD-ME(or PEP-CK)' (57)
Sporobolus aculeatus (L.) P.M.Peterson	T	PL	HG	N	N	N		N	N	N	N						N			N	N			-11.5 - -13.1 (24)	'CL/NAD-ME(or PEP-CK)' (57, 58)
Sporobolus africanus (Pair.) A. Robyns & Tournay	GH	PL	MS-I-Tr														N						N	-13.05(31)	CL/PNAD-ME or EP-CK(57.58)
Sporobolus airiformis Chiov.	GH	SM	O-I-Tr											N									N		'CL/NAD-ME(or PEP-CK)' (57, 58)
Sporobolus alopecuroides (Piller & Mitterp.) P.M.Peterson	T	IT, M	HG-I-Tm		N	N		N	N	N	N		N						N	N	N			-13.1 (24)	'CL/NAD-ME(or PEP-CK)' (57, 58)
Sporobolus angustifolius A. Rich.	GH	SM, GC, ZI	MS, O-I-Tr											N			N						N		'CL/NAD-ME(or PEP-CK)' (57, 58)
Sporobolus borszczowii Regel	T	PL	R-I-Tm						N	N	N		N						N	N					'CL/NAD-ME(or PEP-CK)' (57, 58)

C_4 lineage **(bold)** and species	Life form	Chorotype	Ecotype	Distribution in Southwest Asia																				δ^{13}C	Leaf anatomy, C_4 Photosynthetic subtype (references)
				Afghanistan	Armenia	Azerbaijan	Bahrain	Iran	Iraq	Israel/Palestine	Jordan	Kuwait	Lebanon	Oman	Pakistan	Qatar	Saudi Arabia	Sinai	Syria	Turkey	Turkmenistan	UAE	Yemen		
Sporobolus confinis (Steud.) Chiov.	GH	SM	MS-I-Tr																				N		'CL/NAD-ME(or PEP-CK)' (57, 58)
Sporobolus consimilis Fresen.	GH	PL	HL-Tr											N			N						N	-13.63(+)	'CL/NAD-ME(or PEP-CK)' (57, 58)
Sporobolus coromandelianus (Retz.) Kunth	T	PL	PH-Tr	N										N	N								N	-15.41(31)	'CL/NAD-ME(or PEP-CK)' (57, 58)
Sporobolus diandrus (Retz.) P.Beauv.	GH	PL	MS-I-Tr											N	N								N		'CL/NAD-ME(or PEP-CK)' (57, 58)
Sporobolus factorovskyi (Eig) P.M.Peterson	T	M	HL-I-Tm		N					N	N		N						N	N				-16.63 (*)	'CL/NAD-ME(or PEP-CK)' (57, 58)
Sporobolus festivus Hochst. ex A. Rich.	GH	PL	O-I-Tr														N						N	-12.49(31)	'CL/NAD-ME(or PEP-CK)' (57, 58)
Sporobolus gloeoclados Cope	GH	SM	MS-Tr											Ne									Ne		'CL/NAD-ME(or PEP-CK)' (57, 58)
Sporobolus helvolus (Trin.) T. Durand & Schinz	GH	PL	PS-Tr												N		N						N		'CL/NAD-ME(or PEP-CK)' (57, 58)
Sporobolus indicus (L.) R.Br.	GH	PL	R-Tr							Ii	Ii														'CL/NAD-ME(or PEP-CK)' (57, 58)
Sporobolus ioclados (Nees ex Trin.) Nees	GH	PL	PS-Tr	N			N	N				N		N	N	N	N					N	N	-13.23(31)	CL/NAD-ME(57.58)
Sporobolus minimus Cope	T	SM	XE-Tr											N											'CL/NAD-ME(or PEP-CK)' (57, 58)
Sporobolus minuartioides (Bornm.) P.M.Peterson	T	M	MS-Tm							Ne	Ne														'CL/NAD-ME(or PEP-CK)' (57, 58)
Sporobolus minutus Link	T	SM	PH-L-Tr														N						N		'CL/NAD-ME(or PEP-CK)' (57, 58)
Sporobolus natalensis (Steud.) T. Durand & Schinz	GH	PL	XE-I-Tr														N						N	-11.55(31)	'CL/NAD-ME(or PEP-CK)' (57, 58)
Sporobolus nervosus Hochst.	GH	PL	XE-I-Tr											N	N		N						N	-12.75(31)	'CL/NAD-ME(or PEP-CK)' (57, 58)
Sporobolus niliacus (Fig. & De Not.) P.M.Peterson	T	SS, SM	MS-Tr							N	N														'CL/NAD-ME(or PEP-CK)' (57, 58)
Sporobolus panicoides A. Rich.	T	PL	O-I-Tr																				N	-13.05(31)	'CL/NAD-ME(or PEP-CK)' (57, 58)
Sporobolus pellucidus Hochst.	GH	PL	PS-I-Tr											N			N						N	-12.33(31)	'CL/NAD-ME(or PEP-CK)' (57, 58)
Sporobolus pungens (Schreb.) Kunth	GH	M	XE-L-Tm							N	N		N						N						'CL/NAD-ME(or PEP-CK)' (57, 58)
Sporobolus pyramidalis P. Beauv	GH	PL	O-I-Tr											N			N						N	-11.78(31)	CL/NAD-ME or PEP-CK(57.58)
Sporobolus ruspolianus Chiov.	GH	SM	XE-Tr											N									N		'CL/NAD-ME(or PEP-CK)' (57, 58)
Sporobolus schoenoides (L.) P.M.Peterson	T	PL	HL	N	N	N		N	N	N	N		N		N		N	N	N	N			N	-12.88 (+)	'CL/NAD-ME(or PEP-CK)' (57, 58)
Sporobolus spicatus (Vahl) Kunth	GH	PL	PH-Tr				N							N		N	N	N				N	N	-13.15(31)	CL/NAD-ME(57.58)
Sporobolus stapfianus Gand.	GH	PL	O-I-Tr																				N	-12.69(31)	'CL/NAD-ME(or PEP-CK)' (57, 58)
Sporobolus tenuissimus (Schrank) Kuntze	T	BR, CR	R-Tr											Ii											'CL/NAD-ME(or PEP-CK)' (57, 58)
Sporobolus tourneuxii Coss.	GH	PL	O-I-Tr					N						N	N								N		'CL/NAD-ME(or PEP-CK)' (57, 58)
Sporobolus turkestanicus (Eig) P.M.Peterson	T	ES, IT	HL-Tm																		N				'CL/NAD-ME(or PEP-CK)' (57, 58)
Sporobolus virginicus (L.) Kunth	GH	PL	PH, HG-Tr											N	N					N			N	-14.08 (31)	'CL/NAD-ME(or PEP-CK)' (57, 58)
Tetrachaete elionuroides Chiov.	T	SM	XE-Tr											N									N		'CL/NAD-ME(or PEP-CK)' (57)
Tetrapogon cenchriformis (A.Rich) Clayton	T	SS, SM, SU	O-I-Tr														N						N		'CL/NAD-ME(or PEP-CK)' (57)
Tetrapogon roxburghiana (Schult.) P.M.Peterson	GH	PL	O-I-Tr																				N		'CL/NAD-ME(or PEP-CK)' (57)
Tetrapogon tenellus (Koen. ex Roxb.) Chiov.	T	PL	XE-Tr											N	N		N						N	-12.97(31)	'CL/NAD-ME(or PEP-CK)' (57)
Tetrapogon villosus Desf.	GH	PL	O-I-Tr	N			N	N		N	N		N	N	N		N	N	N		N	N	N	-12.36 (*)	'CL/NAD-ME(or PEP-CK)' (57)

C₄ lineage (bold) and species	Life form	Choro-type	Ecotype	Distribution in Southwest Asia																				δ13C	Leaf anatomy, C₄ Photosynthetic subtype (references)
				Afghanistan	Armenia	Azerbaijan	Bahrain	Iran	Iraq	Israel/Palestine	Jordan	Kuwait	Lebanon	Oman	Pakistan	Qatar	Saudi Arabia	Sinai	Syria	Turkey	Turkmenistan	UAE	Yemen		
Tragus berteronianus Schult.	T	PL	XH, R-Tr	N				N						N	N		N					N	N	-15.07(31)	CL/NAD-ME (57.58)
Tragus mongolorum Ohwi	T	PL	MS												N									-15.77 (*)	'CL/NAD-ME' (57, 58)
Tragus racemosus (L.) All.	T	PL	XE		N	N		N					N	N	N		N		N	N	N	N	N	-12.32(31), -14.57 (*)	CL/NAD-ME (57.58)
Trichoneura mollis (Kunth) Ekman	T	SS, SM, SU	XE-Tr											N			N						N		'CL/NAD-ME(or PEP-CK)' (57)
Trigonochloa rupestris (C.E.Hubb.) P.M.Peterson & N.Snow	GH	SM	MS-I-Tr																				N		'CL/NAD-ME(or PEP-CK)' (57)
Trigonochloa uniflora (Hochst. ex A.Rich.) P.M.Peterson & N.Snow	T	PL	O-I-Tr																				N	-14.69(31)	'CL/NAD-ME(or PEP-CK)' (57)
Tripogon afrieanus (Coss. & Durieu) H. Scholz	GH	SS, SM	O-I-Tr											N			N								'CL/NAD-ME(or PEP-CK)' (57)
Tripogon capillatus Jaub. & Spach	GH	SM, I, IC	MS											N										-10.50 (*)	'CL/NAD-ME(or PEP-CK)' (57)
Tripogon filiformis Nees ex Steud.	GH	I, IC	MS-Tr												N									-11.33 (*)	'CL/NAD-ME(or PEP-CK)' (57)
Tripogon leptophyllus (A. Rich.) Cufod.	GH	SM	MS-I-Tr											N									N		'CL/NAD-ME(or PEP-CK)' (57)
Tripogon montanus Chiov.	GH	SM	O-I-Tr														N						N		'CL/NAD-ME(or PEP-CK)' (57)
Tripogon multiflorus Mire & H. Gillet	GH	SM	O-I-Tr											N			N						N		'CL/NAD-ME(or PEP-CK)' (57)
Tripogon oliganthos Cope	GH	SM	MS-I-Tr																				Ne		'CL/NAD-ME(or PEP-CK)' (57)
Tripogon purpurascens Duthie	T	PL	O-I-Tm	N										N	N		N						N		'CL/NAD-ME(or PEP-CK)' (57)
Tripogon subtilissimus Chiov.	GH	SM	O-I-Tr											N									N		'CL/NAD-ME(or PEP-CK)' (57)
Triraphis pumilio R. Br.	T	SS, SM	MS, XE-Tr											N			N						N	-13.87 (+)	'CL/NAD-ME(or PEP-CK)' (57)
Urochondra setulosa (Trin.) C.E. Hubb.	GH	SM, SS	HL-Tr											N	N		N						N		'CL/NAD-ME(or PEP-CK)' (57)
Zaqiqah mucronata (Forssk.) P.M.Peterson & Romasch.	GH	SM	PH-L-Tr														N						N		'CL/NAD-ME(or PEP-CK)' (57)
**11) *Arundinellae***																									
Arundinella nepalensis Trin.	GH	PL	MS-I-Tr											N	N									-12.14 (*)	'AU(55)/NADP-ME' (56, 57)
Arundinella pumila (Hochst. ex A. Rich.) Steud.	T	PL	MS-I-Tr											N											'AU(55)/NADP-ME' (56, 57)
Garnotia tenella (Arn. ex Miq.) Janowski	GH	PL	MS-I-Tr											N											'AU(55)/NADP-ME' (56, 57)
**12) *Tristachyideae***																									
Danthoniopsis barbata (Nees) C.E. Hubb.	GH	SS, SM	O-Tr														N						N	-10.44 (*)	'AU(55)/NADP-ME' (56, 57)
Danthoniopsis stocksii (Boiss.) C. E. Hubb.	GH	SS	XE-I-Tr					Ne							Ne									-12.64 (*)	'AU(55)/NADP-ME' (56, 57)
Loudetia flavida (Stapf) C.E. Hubb.	GH	PL	MS-I-Tr											N										-11.44 (*)	'AU(55)/NADP-ME' (56, 57)
**13) *Digitaria***																									
Anthephora laevis Stapf & C. E. Hubb.	GH	SM, SS	MS, XE-Tr							N	N							N							'CL/NADP-ME' (56,57)
Anthephora nigritana Stapf & C.E. Hubb	GH	PL	O-I-Tr														N						N		'CL/NADP-ME' (56,57)
Anthephora pubescens Nees	GH	PL	MS, XE-Tr					N									N						N	-12.22(31)	'CL/NADP-ME' (56,57)
Digitaria abludens (Roem. & Schult.) Veldkamp	T	I,IC	MS, R-Tr												N										'CL/NADP-ME' (56,57)
Digitaria abyssiniea (Hochst. ex A. Rich.) Stapf	GH	PL	PS, R-I-Tr														N						N		'CL/NADP-ME' (56,57)

				Distribution in Southwest Asia																					
C_4 lineage **(bold)** and species	Life form	Choro-type	Ecotype	Afghanistan	Armenia	Azerbaijan	Bahrain	Iran	Iraq	Israel/Palestine	Jordan	Kuwait	Lebanon	Oman	Pakistan	Qatar	Saudi Arabia	Sinai	Syria	Turkey	Turkmenistan	UAE	Yemen	$\delta^{13}C$	Leaf anatomy, C_4 Photosynthetic subtype (references)
Digitaria bicornis (Lam.) Roem. & Schult.	T	PL	R-Tr												N										'CL/NADP-ME' (56,57)
Digitaria ciliaris (Retz.) Koeler	T	PL	R	N	N	N	N	N		N	N	N		N	N		N					N	N	-10.1 (24)	'CL/NADP-ME' (56,57)
Digitaria cruciata (Nees) A. Camus	T	PL	MS-I	N				N																-14.99 (*)	'CL/NADP-ME' (56,57)
Digitaria diagonalis (Nees) Stapf	GH	PL	MS-I-Tr																				N	-10.57(31)	'CL/NADP-ME' (56,57)
Digitaria horizontalis Willd.	T	PL	R-Tr			I																			'CL/NADP-ME' (56,57)
Digitaria ischaemum (Schreb.) Schreb.ex Muhl.	T	PL	MS					N							N					N	N			-12.5 (24)	'CL/NADP-ME' (56,57)
Digitaria longiflora (Retz.) Pers.	T or GH	PL	MS-Tr												N									-12.2(30)	'CL/NADP-ME' (56,57)
Digitaria neghellensis J.-P. Lebrun	GH	SM	XE-I-Tr											N											'CL/NADP-ME' (56,57)
Digitaria nodosa Parl.	GH	SS, SM	XE-Tr	N				N						N	N		N						N	-13.62 (*)	'CL/NADP-ME' (56,57)
Digitaria pennata (Hochst.) T. Cooke	GH	SM, I	MS, XE-Tr											N	N		N						N		'CL/NADP-ME' (56,57)
Digitaria radicosa (J.Presl) Miq.	T	PL	R-Tr												N									-11.84(31)	'CL/NADP-ME' (56,57)
Digitaria rivae (Chiov.) Stapf	GH	SM	XE-Tr																				N		'CL/NADP-ME' (56,57)
Digitaria sanguinalis (L.) Scop.	T	PL	R	N	N	N		N	N	N	N		N		N		N	N	N	N	N	N		-11.2 (33)	'CL/NADP-ME' (56,57)
Digitaria setigera Roth ex Roem. & Schult.	T	PL	MS-Tr												N										'CL/NADP-ME' (56,57)
Digitaria stewartiana Bor	T	CA	MS-Tm												N										'CL/NADP-ME' (56,57)
Digitaria stricta Roth ex Roemer & Schultes	T	PL	R, MS-I-Tr					N						N	N										'CL/NADP-ME' (56,57)
Digitaria ternata (A. Rich.) Stapf	T	PL	R-I-Tr																				N	-10.92(+)	'CL/NADP-ME' (56,57)
Digitaria velutina (Forssk.) P. Beauv.	T	PL	XE-Tr											N			N						N	-10.99(31)	'CL/NADP-ME' (56,57)
Digitaria violascens Link	T	PL	R	N		N									N									-11.53(30)	'CL/NADP-ME' (56,57)
**14)** ***Echinochloa***																									
Echinochloa colonum (L.) Link	T	PL	PS, R-I-Tr	N			N	N	N	N	N	N	N	N	N	N	N	N	N	N		N	N	-12.58(31)	'CL/NADP-ME' (56,57)
Echinochloa crus-galli (L.) P.Beauv.	T	PL	HG	N	N	N		N	N	N	N		N	N	N		N		N	N	N	N	N	-16.2 (34)	'CL/NADP-ME' (56,57)
Echinochloa frumentacea Link	T	I	R	I											N		I							-11.2(33)	'CL/NADP-ME' (56,57)
Echinochloa oryzoides (Ard.) Fritsch	T	PL	HG-Tm	N		N		N							N					N	N			-10.49 (*)	'CL/NADP-ME' (56,57)
Echinochloa pyramidalis (Lam.) Hitchc. & Chase	GH	PL	HG, R-I-Tr														N							-13.41(31)	'CL/NADP-ME' (56,57)
Echinochloa stagnina (Retz.) P. Beauv.	GH	PL	HG-Tr												N			N						-10.63(31)	'CL/NADP-ME' (56,57)
**15)** ***Melinidinae/Paninicae/Cenchrinae***																									
Cenchrus abyssinicus (Hack.) Morrone	GH	SM	HG-Tr																				N		'CL/NADP-ME' (57, 77)
Cenchrus aethiopicus (Fresen.) Rudov comb.nov. (Syn:. *Snowdenia polystachya* (Fresen.)Pilg.)	T	SM	MS-I-Tr														N						N		'CL/NADP-ME' (57, 77)
Cenchrus biflorus Roxb.	T	PL	XE-Tr					N							N		N						N	-11.08(31)	'CL/NADP-ME' (57, 77)
Cenchrus ciliaris L.	GH	Pl	R-Tr	N			N	N	N	N	N	N	N	N	N	N	N	N	N			N	N	-12.26(31)	'CL/NADP-ME' (57, 77)
Cenchrus clandestinus (Hochst. ex Chiov.) Morrone	GH	PL	R-Tr							Ii	Ii						Ii						Ii		'CL/NADP-ME' (57, 77)

C_4 lineage **(bold)** and species	Life form	Choro-type	Ecotype	Distribution in Southwest Asia																				$\delta^{13}C$	Leaf anatomy, C_4 Photosynthetic subtype (references)
				Afghanistan	Armenia	Azerbaijan	Bahrain	Iran	Iraq	Israel/Palestine	Jordan	Kuwait	Lebanon	Oman	Pakistan	Qatar	Saudi Arabia	Sinai	Syria	Turkey	Turkmenistan	UAE	Yemen		
Cenchrus divisus (J.F.Gmel.) Verloove (incl. *Cenchrus elatus* (Steud.) Verloove and *C. ramosissimus* Poir.)	GH	SS, SM	PS-TR	N			N	N	N	N	N	N	N	N	N	N	N	N	N			N	N	-13.56 (*)	'CL/NADP-ME' (57, 77)
Cenchrus echinatus L.	T	PL	R-Tr				Ii			Ii	Ii				Ii		Ii	Ii				Ii			'CL/NADP-ME' (57, 77)
*Cenchrus flaccidus (*Griseb.) Morrone	GH	IT, CA	O-I-Tm	N				N							N									-11.24 (*)	'CL/NADP-ME' (57, 77)
Cenchrus geniculatus Thunb.	GH	PL	MS-I-Tr																				N	-11.25(31)	'CL/NADP-ME' (57, 77)
Cenchrus glaucifolius (Hochst. ex A.Rich.) Rudov & Akhani comb.nov. (Basionym: Pennisetum glaucifolium Hochst. ex A.Rich.)	GH	SM	XE-Tr																				N	-10.76 (*)	'CL/NADP-ME' (57, 77)
Cenchrus hohenackeri (Hochst. ex Steud.) Morrone	GH	PL	HG-I-Tr												N									-10.71 (*)	'CL/NADP-ME' (57, 77)
Cenchrus lanatus (Klotzsch) Morrone	GH	IT	O-I-Tm	N											N									-11.58 (*)	'CL/NADP-ME' (57, 77)
Cenchrus longisetus M.C.Johnst.	GH	SM	MS-I-Tr														N						N	-10.48 (*)	'CL/NADP-ME' (57, 77)
Cenchrus longispinus (Hack.) Fernald	T	MA, NA	R-Tm					Ii		Ii	Ii														'CL/NADP-ME' (57, 77)
Cenchrus macrourus (Trin.) Morrone	GH	PL	MS-I-Tr														N						N	-9.73 (*)	'CL/NADP-ME' (57, 77)
Cenchrus nubicus (Hochst.) Rudov & Akhani comb.nov. (Basionym: *Gymnotrix nubica* Hochst.)	T	SM	R-I-Tr														N							-11.57 (*)	'CL/NADP-ME' (57, 77)
*Cenchrus orientalis (*Rich.) Morrone	GH	PL	XE	N	N	N		N	N	N	N		N	N	N		N	N	N	N	N			-11.59 (+)	'CL/NADP-ME' (57, 77)
Cenchrus pennisetiformis Hochst. & Steud.	GH	PL	MS-Tr					N						N	N		N	N				N	N	-13.40 (*)	'CL/NADP-ME' (57, 77)
Cenchrus polystachios (L.) Morrone	GH	PL	MS-I-Tr														N							-12.06 (*)	'CL/NADP-ME' (57, 77)
Cenchrus prieurii (Kunth) Maire	T	PL	XE, R-Tr					N							N								N		'CL/NADP-ME' (57, 77)
Cenchrus purpureus (Schumach.) Morrone	GH	PL	R-Tr							Ii	Ii			Ii			Ii							-10.07(31)	'CL/NADP-ME' (57, 77)
Cenchrus setaceus (Forssk.) Morrone	GH	SS, SM	MS, -Tr				N			N	N	N	N	N		N	N	N	N				N	-12.6 (24)	'CL/NADP-ME' (57, 77)
Cenchrus setiger Vahl	GH	PL	R-Tr					N				N		N	N	N	N					N	N	-10.55(2)	'CL/NADP-ME' (57, 77)
*Cenchrus sieberianus (S*chltdl.) Verloove	T	PL	R-Tr				I							I			I						I		'CL/NADP-ME' (57, 77)
Cenchrus spinifex Cav.	T or H	PL	R-Tr							Ii	Ii														'CL/NADP-ME' (57, 77)
Cenchrus stramineus (Peter) Morrone	GH	SM	XE-I-Tr														N						N	-11.95 (*)	'CL/NADP-ME' (57, 77)
Cenchrus unisetus (Nees) Morrone	GH	PL	MS-I-Tr														N						N	-10.39(*)	'CL/NADP-ME' (57, 77)
Cenchrus yemensis (Deflers) Rudov & Akhani comb nov. (Basionym: *Pennisetum yemense* Deflers)	GH	SM	XE-I-Tr														N						N		'CL/NADP-ME' (57, 77)
Eriochloa barbatus (Trin.) S.Yadav & M.R.Almeida	T	PL	PS-Tr											N	N		N						N	-12.3(31)	'CL/NAD-ME or PEP-CK'(57, 77)
Eriochloa meyeriana (Nees) Pilg.	GH	PL	HG-I-Tr																				N	-12.7(33)	'CL/NAD-ME or PEP-CK'(57, 77)
Eriochloa procera (Retz.) C. E. Hubbard	T	PL	MS-Tr												N									-12.02 (*)	'CL/NAD-ME or PEP-CK'(57, 77)
Eriochloa succincta (Trin.) Kunth	T	IT	R-Tm		N	N		N	N												N				'CL/NAD-ME or PEP-CK'(57, 77)

				Distribution in Southwest Asia																					
C₄ lineage **(bold)** and species	Life form	Choro-type	Ecotype	Afghanistan	Armenia	Azerbaijan	Bahrain	Iran	Iraq	Israel/Palestine	Jordan	Kuwait	Lebanon	Oman	Pakistan	Qatar	Saudi Arabia	Sinai	Syria	Turkey	Turkmenistan	UAE	Yemen	$\delta^{13}C$	Leaf anatomy, C₄ Photosynthetic subtype (references)
Melinis longiseta (Hochst. ex A. Rich.) Zizka	GH	PL	XE-Tr																				N	-12.55(31)	'CL/NAD-ME or PEP-CK'(57, 77)
Melinis repens (Willd.) Zizka	T	PL	XE-Tr									N		N	N		N						N	-13.0(31)	'CL/NAD-ME or PEP-CK'(57, 77)
Moorochloa eruciformis (Sm.) Veldkamp	T	PL	R	N	N	N			N	N	N			N	N		N			N	N	N	N	-12.1(33)	'CL/NAD-ME or PEP-CK'(57, 77)
Panicum antidotale Retz.	GH	PL	PS, R	N		N		N		N	N	N		N	N		N				N	N	N	-14.1 (35)	'CL/NAD-ME' (57, 77)
Panicum atrosanguineum Hochst. ex A.Rich.	T	PL	MS, R											N	N		N						N		'CL/NAD-ME' (57, 77)
Panicum capillare L.	T	MA, NA	R-Tm		I			I		I	I				I					I				-14.3 (35)	'CL/NAD-ME' (57, 77)
Panicum coloratum L.	GH	PL	PS, R-Tr							N	N			N	N		N						N	-12.6(31)	'CL/NAD-ME' (57, 77)
Panicum deustum Thunb.	GH	PL	MS-Tr	N																				-11.9 (35)	'CL/NAD-ME' (57, 77)
Panicum dichotomiflorum Michx.	T	PL	R																	I				-14.1(35)	'CL/NAD-ME' (57, 77)
Panicum flexuosum Retz.	T	PL	MS, HG-Tr												N									-10.5 (36)	'CL/NAD-ME' (57, 77)
Panicum humile Steud.	T	PL	R-Tr												N									-9.7 (35)	'CL/NAD-ME' (57, 77)
Panicum miliaceum L.	T	I, X	R-Tr	N	I	I		I	I	I	I			I	N		I			I	I	I	I	-16.0 (35)	'CL/NAD-ME' (57, 77)
Panicum paludosum Roxb.	T	PL	MS, HG-Tr					N							N									-12.8 (35)	'CL/NAD-ME' (57, 77)
Panicum repens L.	GH	PL	R					N	N	N	N		N		N		N	N	N	N			N	-12.3(35)	'CL/NAD-ME' (57, 77)
Panicum rigidum Balf.	GH	SM	PS-Tr																				Ne	-13.3 (36)	'CL/NAD-ME' (57, 77)
Panicum socotranum Cope	GH	SM	MS-Tr																				Ne		'CL/NAD-ME' (57, 77)
Panicum turgidum Forssk.	GH	PL	PH-Tr				N	N	N	N	N	N		N	N	N	N	N				N	N	-12.7(35)	'CL/NAD-ME' (57, 77)
Setaria barbata (Lam.) Kunth	T	PL	MS-I-Tr																				N		'CL/NADP-ME' (57, 77)
Setaria desertorum (A.Rich.) Morrone	GH	SM	R-Tr											N			N						N		'CL/NADP-ME' (57, 77)
Setaria flavida (Retz.) Veldkamp	GH	PL	MS-I-Tr												N								N		'CL/NADP-ME' (57, 77)
Setaria geminata (Forssk.) Veldkamp	GH	PL	PS-I-Tr							N	N		N	N	N		N		N				N	-12.68(31)	'CL/NADP-ME' (57, 77)
Setaria helvola (L.f.) Roem. & Schult.	T	PL	R-I	N				N	N	N	N	N	N	N	N		N		N	N			N	-11.9 (24)	'CL/NADP-ME' (57, 77)
Setaria intermedia Roem. & Schult.	T	PL	MS-I-Tr											N	N								N		'CL/NADP-ME' (57, 77)
Setaria megaphylla (Steud.) T. Durand & Schinz	GH	PL	MS-I-Tr														N						N		'CL/NADP-ME' (57, 77)
Setaria parviflora (Poir.) Kerguelen	GH	PL	MS							Ii	Ii														'CL/NADP-ME' (57, 77)
Setaria punctata (Burm.f.) Veldkamp	GH	PL	HG-Tr												N										'CL/NADP-ME' (57, 77)
Setaria sagittifolia (Hochst. ex A. Rich.) Walp.	T	PL	MS-I-Tr																				N	-12.07(31)	'CL/NADP-ME' (57, 77)
Setaria sphacelata (Schumach.) Stapf & C.E. Hubb. ex M.B. Moss	GH	PL	R-I-Tr														N						N	-11.92(31)	'CL/NADP-ME' (57, 77)
Setaria verticillata (L.) P. Beauv.	T	PL	R	N	N	N	N	N	N	N	N	N	N	N	N	N	N	N	N	N	N	N	N	-13.6(32)	'CL/NADP-ME' (57, 77)
Setaria viridis (L.) P. Beauv.	T	PL	R-I	N	N	N	N	N	N	N	N	N	N	N	N	N	N	N	N	N	N	N		-12.93 (+)	'CL/NADP-ME' (57, 77)
Tricholaena teneriffae (L. f.) Link	GH	PL	R	N			N	N		N	N		N	N	N		N		N			N	N	-13.14(2)	'CL/NAD-ME or PEP-CK'(57, 77)
Tricholaena vestita (Balfour) Stapf & C.E. Hubb	GH	SM	XE-I-Tr																				Ne		'CL/NAD-ME or PEP-CK'(57, 77)
Urochloa arida (Mez) Rudov comb.nov. (syn.: *Brachiaria arida* (Mez.) Stapf)	GH	SM	MS-I-Tr																				N		'CL/NAD-ME or PEP-CK'(57, 77)
Urochloa brizantha (A.Rich.) R.D.Webster	GH	PL	MS-I-Tr																				N	-11.65(31)	'CL/NAD-ME or PEP-CK'(57, 77)

C_4 lineage **(bold)** and species	Life form	Choro-type	Ecotype	Distribution in Southwest Asia																				δ^{13}C	Leaf anatomy, C_4 Photosynthetic subtype (references)
				Afghanistan	Armenia	Azerbaijan	Bahrain	Iran	Iraq	Israel/Palestine	Jordan	Kuwait	Lebanon	Oman	Pakistan	Qatar	Saudi Arabia	Sinai	Syria	Turkey	Turkmenistan	UAE	Yemen		
Urochloa chusqueoides (Hack.) Rudov comb. nov. (Syn.: *Brachiaria chusqueoides* (Hack.) Clayton)	GH	PL	MS-I-Tr																				N		'CL/NAD-ME or PEP-CK'(57, 77)
Urochloa comata (Hochst. ex A.Rich.) Sosef	T	PL	R, MS-I-Tr														N						N		'CL/NAD-ME or PEP-CK'(57, 77)
Urochloa deflexa (Schumach.) H.Scholz	T	PL	MS-Tr											N	N		N						N	-13.98(31)	'CL/NAD-ME or PEP-CK'(57, 77)
Urochloa distachya (L.) T.Q.Nguyen	GH	PL	R-Tr					N							N										'CL/NAD-ME or PEP-CK'(57, 77)
Urochloa lata (Schumach.) C.E.Hubb.	T	SS, SM, I	MS-I-Tr											N			N						N		'CL/NAD-ME or PEP-CK'(57, 77)
Urochloa leersioides (Hochst.) A.M.Torres & C.M.Morton	T	PL	MS-I-Tr											N			N						N		'CL/NAD-ME or PEP-CK'(57, 77)
Urochloa maxima (Jacq.) R.D.Webster	GH	PL	R-Tr	N			N			N	N			N	N		N					N	N	-13.3 (35)	'CL/NAD-ME or PEP-CK'(57, 77)
Urochloa mutica (Forssk.) T.Q.Nguyen	GH	PL	HG-Tr							N	N		N						N				N		'CL/NAD-ME or PEP-CK'(57, 77)
Urochloa ovalis (Stapf) Rudov comb. nov. (Syn.: *Brachiaria ovalis* Stapf)	T	SM,SS	O-Tr											N	N		N						N		'CL/NAD-ME or PEP-CK'(57, 77)
Urochloa panicoides P. Beauv.	T	PL	MS, R-Tr					N		N	N			N	N								N	-12.2 (33)	'CL/NAD-ME or PEP-CK'(57, 77)
Urochloa ramosa (L.) T.Q.Nguyen	T	PL	MS-Tr	N										N	N		N					N	N		'CL/NAD-ME or PEP-CK'(57, 77)
Urochloa remota (Retz.) Ashalatha & V.J. Nair	GH	I, SM	MS-I-Tr																				N		'CL/NAD-ME or PEP-CK'(57, 77)
Urochloa reptans (L.) Stapf	T	PL	R-Tr	N											N		N					N	N		'CL/NAD-ME or PEP-CK'(57, 77)
Urochloa texana (Buckley) R.D.Webster	T	MA, NA	R-Tm							I	I														'CL/NAD-ME or PEP-CK'(57, 77)
Urochloa trichopus (Hochst.) Stapf	T	PL	MS-I-Tr														N						N	-12.75(+)	'CL/NAD-ME or PEP-CK'(57, 77)
Urochloa xantholeuca (Hack.) H.Scholz (incl. *Brachiaria leucacrantha* (K. Schum.) Stapf)	T	PL	MS-I-Tr														N							-11.80(+)	'CL/NAD-ME or PEP-CK'(57, 77)
**16) *Paspalum***																									
Paspalum dilatatum Poir.	GH	AM, AN, BR	R-Tr		Ii	Ii		Ii	Ii	Ii	Ii				Ii		Ii	Ii		Ii		Ii		-11.20 (*)	'CL/NADP-ME' (56, 57)
Paspalum distichum L.	GH	PL	HG, MS	Ii	Ii	Ii		Ii	Ii	Ii	Ii		Ii	Ii	Ii		Ii	Ii	Ii	Ii	Ii		Ii	-13.75 (+)	'CL/NADP-ME' (56, 57)
Paspalum scrobiculatum L.	GH	PL	R-I-Tr												N								N	-12.31(31)	'CL/NADP-ME' (56, 57)
Paspalum thunbergii Kunth ex Steudel	GH	SJ	R-Tm																	Ii					'CL/NADP-ME' (56, 57)
Paspalum vaginatum Sw.	GH	PL	HG-Tr				Ii							Ii			Ii					Ii	Ii	-12.82(31)	'CL/NADP-ME' (56, 57)
**17) *Stipagrostis***																									
Stipagrostis acutiflora (Trin. & Rupr.) De Winter	GH	SS	PS, XE-Tr							N	N			N				N				N	N	-13.6 (*)	'SA(55)/NADP-ME' (56, 60)
Stipagrostis arachnoidea (Litv.) De Winter	GH	IT	PS, XE-Tm																		N				'SA(55)/NADP-ME' (56, 60)
Stipagrostis barbata H.Scholz	GH	IT	PS, XE-I-Tm					N																	'SA(55)/NADP-ME' (56, 60)
Stipagrostis ciliata (Desf.) De Winter	GH	SS	PS, XE-I-Tr	N				N	N	N	N	N		N	N	N	N	N					N	-13.54(31)	'SA(55)/NADP-ME' (56, 60)
Stipagrostis dhofariensis Cope	GH	SM	PS-L-Tr											Ne											'SA(55)/NADP-ME' (56, 60)
Stipagrostis drarii (Taeckh.) De Winter	GH	SS	PS, XE-Tr							Ne	Ne	Ne					Ne	Ne				Ne	Ne		'SA(55)/NADP-ME' (56, 60)
Stipagrostis foexiana (Maire &Wilczek) De	GH	SM	PS, XE-I-Tr														N					N	N		'SA(55)/NADP-ME' (56, 60)

C_4 lineage **(bold)** and species	Life form	Chorotype	Ecotype	Distribution in Southwest Asia: Afghanistan	Armenia	Azerbaijan	Bahrain	Iran	Iraq	Israel/Palestine	Jordan	Kuwait	Lebanon	Oman	Pakistan	Qatar	Saudi Arabia	Sinai	Syria	Turkey	Turkmenistan	UAE	Yemen	δ^{13}C	Leaf anatomy, C_4 Photosynthetic subtype (references)
Winter																									
Stipagrostis griffithii (Henrard) De Winter	GH	IT	XE-I-Tm	Ne																					'SA(55)/NADP-ME' (56, 60)
Stipagrostis hirtigluma (Steud.) De Winter	T	SS, SM	XE-TR					N		N	N			N	N		N	N				N	N	-12.43(31)	'SA(55)/NADP-ME' (56, 60)
Stipagrostis karelinii (Trin. & Rupr.) H.Scholz	GH	IT	PS, XE-I-Tm	N				N													N			-13.27 (*)	SA(55)/NADP-ME (56, 60)
Stipagrostis lanata (Forssk.) De Winter	GH	SS	PS, XE-Tr					N	N	N	N		N				N	N	N			N			'SA(55)/NADP-ME' (56, 60)
Stipagrostis masirahensis H. Scholz	GH	SM	XE-Tr											Ne											'SA(55)/NADP-ME' (56, 60)
Stipagrostis multinerva H. Scholz	T	SS	PS, XE-I-Tr					N						N			N	N					N	-13.98 (*)	'SA(55)/NADP-ME' (56, 60)
Stipagrostis obtusa (Delile) Nees	GH	PL	PS-Tr					N	N	N	N	N			N	N	N	N					N	-12.63 (*)	'SA(55)/NADP-ME' (56, 60)
Stipagrostis pennata (Trin.) De Winter	GH	IT, CA	PS, XE-I-Tm	N				N													N			-12.61 (*)	SA(55)/NADP-ME (56, 60)
Stipagrostis plumosa (L.) Munro ex T.Anders. (incl. *S. brachypoda* (Tausch) De Winter and *S. fallax* H.Scholz)	GH	PL	PS, XE	N	N	N	N	N	N	N	N	N	N	N	N	N	N	N	N	N	N	N	N	-12.87(2)	SA(55)/NADP-ME (56, 60)
Stipagrostis pogonoptila (Jaub. & Spach.) De Winter	GH	PL	PS, XE-I-Tr	N				N							N										'SA(55)/NADP-ME' (56, 60)
Stipagrostis pungens (Desf.) De Winter	GH	SS	XE-I-Tr	N				N										N					N	-12.8 (*)	'SA(55)/NADP-ME' (56, 60)
Stipagrostis raddiana (Savi) De Winter (incl. *S. paradisea* (Edgew.) De Winter)	GH	SS, SM	PS, XE-Tr	N				N	N	N	N			N	N		N	N				N	N	-14.06 (*)	'SA(55)/NADP-ME' (56, 60)
Stipagrostis scoparia (Trin. & Rupr.) De Winter	GH	SS	PS, XE-Tr							N	N						N	N						-12.83(2)	'SA(55)/NADP-ME' (56, 60)
Stipagrostis sokotrana (Vierh.) De Winter	GH	SM	PS, XE-Tr				Ne							Ne		Ne	Ne					Ne	Ne		'SA(55)/NADP-ME' (56, 60)
Stipagrostis uniplumis (Licht.) De Winter	GH	PL	PS, XE-Tr	N				N						N	N		N						N	-13.33(31)	'SA(55)/NADP-ME' (56, 60)
Stipagrostis xylosa Cope	GH	SM	PS-Tr											N									N		'SA(55)/NADP-ME' (56, 60)
**EUDICOTS**																									
**ZYGOPHYLLALES; Zygophyllaceae**																									
**18) *Tribulus/Kallstroemia***																									
Tribulus macropterus Boiss. s.l. (incl. *T. ochroleucus* (Maire) Ozenda & Quezel, *T. mollis* Ehrenb. ex Schweinf.), *T. arabicus* Hosni, *T.omanense* Hosni))	T	PL	PS-I	N				N	N	N	N			N	N	N	N				N	N	N	-13.56 (*), -14.77[5] (*), -12.52 [6](*)	'AT/NADP-ME' (41)
Tribulus pentandrus Forssk. (incl. *T. longipetalus* Viv., *T. bimucronatus* Viv. And *T. pterophorus* C.Presl)	T	PL	PS	N			N	N	N	N	N			N	N	N	N	N						-12.86 [7](*), -12.64[8] (*)	'AT/NADP-ME' (41)
Tribulus terestris L.	T	PL	PS	N	N	N		N	N	N	N	N	N	N	N	N	N	N	N	N	N	N	N	-13.9 (24)	'AT/NADP-ME' (41)
**19) *Tetraena***																									
Tetraena simplex (L.) Beier & Thulin	T	SS	XH-Tr				N	N		N	N			N	N		N					N	N	-13.49 (25)	Similar to PI or KC/NAD-ME (41, 25)
**MALPIGHIALES; Euphorbiaceae**																									
**20) *Euphorbia subgen. Chamaesyce***																									

C_4 lineage **(bold)** and species	Life form	Choro-type	Ecotype	Distribution in Southwest Asia																				δ13C	Leaf anatomy, C_4 Photosynthetic subtype (references)
				Afghanistan	Armenia	Azerbaijan	Bahrain	Iran	Iraq	Israel/Palestine	Jordan	Kuwait	Lebanon	Oman	Pakistan	Qatar	Saudi Arabia	Sinai	Syria	Turkey	Turkmenistan	UAE	Yemen		
Euphorbia abdulghafooriana Abedin	T	SS	XE-Tr														Ne						Ne		'AT/NADP-ME' (41)
Euphorbia anisopetala (Prokh.) Prokh.	T	IT	XH, R-Tr																		N			-11.41 (*)	'AT/NADP-ME' (41)
Euphorbia arabica Hochst. & Steud. Ex Anderson	T	SS	XE-Tr											N			N					N	N	-12.19 (*)	'AT/NADP-ME' (41)
Euphorbia chamaesyce L.	T	IT, M	XE, O-I	N	N	N		N	N	N	N		N		N		N		N	N				-13.12 (22)	'AT/NADP-ME' (41)
Euphorbia clarkeana Hook.	T	SS	PS, R-Tr	N											N										'AT/NADP-ME' (41)
Euphorbia forsskalii J. Gay	Ch	SS, SM	PS, R-Tr		N	N				N	N		N	N			N	N	N	N			N	-11.12 (*)	'AT/NADP-ME' (41)
Euphorbia orymbose Forssk. (incl. *E. turcomanica* Boiss.)	T	PL	PH, R	N	N	N	N	N	N	N	N	N	N	N	N		N	N	N		N		N		'AT/NADP-ME' (41)
Euphorbia hirta L.	T	PL	R						Ii	Ii	Ii	Ii	Ii	Ii	Ii		Ii	Ii	Ii			Ii	Ii	-12.88 - -13.28(23)	AT/NADP-ME (41)
Euphorbia hispida Boiss.	T	SS	XE, R-I	N											N										'AT/NADP-ME' (41)
Euphorbia humifusa Willd.	T	PL	HL, R		N	N		N							N					N				-13.87(23)	'AT/NADP-ME' (41)
Euphorbia hypericifolia L.	T	PL	R						Ii					Ii	Ii					Ii				-11.75 (*)	'AT/NADP-ME' (41)
Euphorbia inaequilatera Sond.	T	SM	PS, R-Tr	N											N								N	-14.16(22)	'AT/NADP-ME' (41)
Euphorbia indica Lam.	T	PL	XH, R	N		N		N	N			N		N	N	N	N					N	N	-13.6 - -13.56(22)	'AT/NADP-ME' (41)
Euphorbia lasiocarpa Klotzsch	T	PL	R-Tr							Ii	Ii													-11.16 (*)	'AT/NADP-ME' (41)
Euphorbia orymbos L.	T	NA, MA	R-Tm		Ii	Ii		Ii		Ii	Ii									Ii				-14.36(23), -15.40 (*)	'AT/NADP-ME' (41)
Euphorbia nutans Lag.	T	PL	R			I		I					I				I		I	I				-13.84 (*)	'AT/NADP-ME' (41)
Euphorbia peplis L.	T	PL	PH-L					N	N	N	N		N							N				-11.77 – 14.07(22)	'AT/NADP-ME' (41)
Euphorbia polycnemoides Hochst. Ex Boiss.	T	SM	XE-Tr														N						N		'AT/NADP-ME' (41)
Euphorbia prostrata Aiton	T	CR, BR, AN	R					I	I	I	I				I									-12.1(22)	'AT/NADP-ME' (41)
Euphorbia riebeckii Pax	Ch	SM	PS-Tr														Ne						Ne		'AT/NADP-ME' (41)
Euphorbia scordiifolia Jacq.	Ch	SS, SM	XE-Tr														N						N	-13.00 (*)	'AT/NADP-ME' (41)
Euphorbia serpens Kunth	T	PL	R					I		I	I				I					I				-11.54 (*)	'AT/NADP-ME' (41)
Euphorbia thymifolia L.	T	I	R-I												N									-12.22 (*)	'AT/NADP-ME' (41)
**BRASSICALES, Cleomaceae**																									
21) Cleome angustifolia																									
Cleome angustifolia Forssk.	T	SS, SM	R-I-Tr																				N	-13.2 (4)	GC/NAD-ME(39)
**22) *Cleome gynandra***																									
Cleome gynandra L.	T	PL	R-Tr							N	N			N	N		N					N	N	-13.5 - -14.5 (4)	AT/NAD-ME (39)
**CARYOPHYLLALES**																									
**Polygonaceae**																									

C_4 lineage **(bold)** and species	Life form	Choro-type	Ecotype	Distribution in Southwest Asia																				$\delta^{13}C$	Leaf anatomy, C_4 Photosynthetic subtype (references)
				Afghanistan	Armenia	Azerbaijan	Bahrain	Iran	Iraq	Israel/Palestine	Jordan	Kuwait	Lebanon	Oman	Pakistan	Qatar	Saudi Arabia	Sinai	Syria	Turkey	Turkmenistan	UAE	Yemen		
**23) *Calligonum***																									
Calligonum acanthopterum Borszcz.	P	IT	PS-I-Tm																		N			-12.35 (*)	'SL/NAD-ME' (41)
Calligonum alatosetosum Maassoumi & Kazempour	P	SS	PS-I-Tr					Ne																-13.01 (*)	'SL/NAD-ME' (41)
Calligonum aphyllum (Pall.) Guerke.	P	IT	PS-I-Tm			N																		-10.09 (*)	'SL/NAD-ME' (41)
Calligonum arborescens Litw.	P	IT	PS-I-Tm					N													N			-12.1(9)	'SL/NAD-ME' (41)
Calligonum bakuense Litw. (incl. *C. petunnikowii* Litw.)	P	IT	PS-Tm			Ne																		-11.50 (*)	'SL/NAD-ME' (41, 18)
Calligonum caput-medusae Schrenk	P	IT	PS-I-Tm					N	N												N			-13.96 (#)	SL/NAD-ME (41)
Calligonum crinitum Boiss.	P	SS	PS-I-Tr	Ne				Ne						Ne	Ne		Ne					Ne	Ne	-15.13 (#)	'SL/NAD-ME' (41)
Calligonum eriopodum Bunge	P	IT	PS-I-Tm					N													N			-15.17 (*)	'SL/NAD-ME' (41)
Calligonum junceum (Fisch. & C. A. Mey.) Litw.	Ch	IT	GP-I-Tm					N													N			-12.7(9)	'SL/NAD-ME' (41)
Calligonum laristanicum Rech. F. & Schiman-Czieka	P	SS	PS-Tr					Ne																-12.89 (*)	'SL/NAD-ME' (41)
Calligonum leucocladum (Schrenk) Bunge (incl. *C. persicum* (Boiss. & Buhes) Boiss.)	P	IT	PS-I-Tm					N													N			-14.62 for C.persicum(#), -12.3(9)	'SL/NAD-ME' (41)
Calligonum litwinowii Drob.	P	IT	PS-I-Tm																		N			-11.94 (*)	'SL/NAD-ME' (41)
Calligonum microcarpum Borszcz.	P	IT	PS-I-Tm					N													N			-11.3(9)	'SL/NAD-ME' (41)
Calligonum mongolicum Turcz. (incl. *C. griseum* Eug Kor. Ex Pavl., *C. rubescens* Mattei, *C. pumilum* Losinskaja, *C. turkestanicum* (Korov.) Pavl., *C. amoenum* Rech. F. & Schiman-Czieka)	P	IT	PS-I-Tm	N				N													N			-12.28 (*)[9], -11.04 [10](*) , -12.1 [11] and -12.6 [12] (9)	'SL/NAD-ME' (41, 79)
Calligonum murex Bunge	P	IT	PS-I-Tm																		N			-10.68 (*)	'SL/NAD-ME' (41)
Calligonum polygonoides L. (incl. *C. intertextum* Rech. F. & Schiman-Czieka , *C. comosum* L Her.)	P	IT, SS	PS	N	N	N	N	N	N	N	N	N	N	N	N	N	N	N	N	N		N	N	-12.59[13] (*), -14.07 (#), -11.87 (2)	'SL/NAD-ME' (41)
Calligonum schizopterum Rech. F. & Schiman.Czeika	P	SS, IT	PS-I					Ne																	'SL/NAD-ME' (41)
Calligonum setosum Litv. (incl. C. ory Litw.)	P	IT	PH-I	N				N													N			-11.6(9)	'SL/NAD-ME' (41)
Calligonum spinosetosum Maassoumi & Batooli	P	IT	PS-I-Tm					Ne																-14.14 (*)	'SL/NAD-ME' (41)
Calligonum tetrapterum Jaub.& Spach. (incl. *C. denticulatum* Bunge ex Boiss., *C. stenopterum* Bunge & Boiss. And *C. orymb* Boiss.)	P	IT, SS	PS-I-Tm					N	N	N	N			N			N			N				-13.72[14] (*), -15.57[15] (*), -14.88[16] (#). -12.24(9)	'SL/NAD-ME' (41)

C_4 lineage **(bold)** and species	Life form	Choro-type	Ecotype	Distribution in Southwest Asia																				δ13C	Leaf anatomy, C_4 Photosynthetic subtype (references)
				Afghanistan	Armenia	Azerbaijan	Bahrain	Iran	Iraq	Israel/Palestine	Jordan	Kuwait	Lebanon	Oman	Pakistan	Qatar	Saudi Arabia	Sinai	Syria	Turkey	Turkmenistan	UAE	Yemen		
Calligonum triste Litv.	P	IT	XH-I-Tm																		N				'SL/NAD-ME' (41)
Calligonum x densum Borszcz. (incl. *C. x platyacanthum* Borszcz.)	P	IT	PS-I-Tm					N													N			-13.24 (*), -11.1 [17](9)	'SL/NAD-ME' (41)
Calligonum x dubianskyi Litv. (incl. *C. x bubyrii* B.Fetsch. ex Pavl. And *C. muravljanskyi* Pavl.)	P	IT	PS-I-Tm																		N			-12.25 [18](*), -11.31 [19](*), -11.6 (9)	'SL/NAD-ME' (41)
Calligonum x paletzkianum Litw. (incl. *C. x cordatum* Korov. Ex Pavl.)	P	IT	PS-I-Tm					N													N			-11.91[20](*), -14.65 (#)	'SL/NAD-ME' (41)
Caryophyllaceae																									
**24) *C₄ Polycarpea***																									
Polycarpaea orymbose (L.) Lam.	T	PL	PS, R-Tr												N		N						N		AT/NADP-ME (41, 43)
**Amaranthaceae *s. str.***																									
**25) C₄ *Aerva***																									
Aerva javanica (Burm.f.) Juss. ex Schult.	T	PL	XE, R	N			N	N	N	N	N	N		N	N	N	N	N				N	N	-13.2(7)	AT/NADP-ME (41)
**26) C₄ *Alternanthera***																									
Alternanthera paronychioides St. Hil.	H	CR, BR, AM	PS-Tr												Ii									-11.5(7)	'AT/NADP-ME' (42, 7)
Alternanthera pungens Kunth.	H	CR, AN, BR	R-Tr							Ii	Ii				Ii		Ii						I	-12.3 (7)	AT/NADP-ME (42, 24)
**27) *Amaranthus***																									
Amaranthus albus L.	T	MA, CR	R	Ii	Ii	Ii		Ii	Ii	Ii	Ii		Ii				Ii	Ii	Ii	Ii	Ii			-14.74 (+)	'AT/NAD-ME' (41, 42)
Amaranthus arenicola I. M. Johnst.	T	MA, NA, CR	R							Ii	Ii													-13.4 (7)	'AT/NAD-ME' (41, 42)
Amaranthus blitoides S. Watson	T	NA	R			Ii		Ii	Ii	Ii	Ii		Ii					Ii	Ii	Ii	Ii			-12.5 (7)	'AT/NAD-ME' (41, 42)
Amaranthus blitum L.	T	PL	R			N		N		N	N		N		N		N	N	N	N	N		N	-13.6 (7)	'AT/NAD-ME' (41, 42)
Amaranthus caudatus L.	T	AN	R			Ii		Ii	Ii	Ii	Ii		Ii		Ii		Ii	Ii	Ii	Ii	Ii			-13.25 (+)	'AT/NAD-ME' (41, 42)
Amaranthus cruentus L.	T	CR	R	Ii				Ii	Ii	Ii	Ii		Ii		Ii		Ii	Ii	Ii	Ii	Ii		Ii	-12.4 (7)	'AT/NAD-ME' (41, 42)
Amaranthus deflexus L.	H	PL	R			Ii		Ii		Ii	Ii		Ii		Ii			Ii	Ii	Ii	Ii			-14.1 (7)	'AT/NAD-ME' (41, 42)
Amaranthus dubius Mart.	T	AN, CR	R						Ii					Ii	Ii									-12.8 (7)	'AT/NAD-ME' (41, 42)
Amaranthus graecizans L. (incl. *A. thellungianus* Nevski and *A. sylvestris* Vill.)	T	PL	R	N	N	N	N	N	N	N	N	N	N	N	N	N	N	N	N	N	N	N	N	-12.76 (+), -12.4(7)	'AT/NAD-ME' (41, 42)
Amaranthus hybridus L.	T	PL	R	Ii	Ii	Ii		Ii	Ii	Ii	Ii		Ii	Ii	Ii	Ii	Ii	Ii	Ii	Ii		Ii	Ii	-13.9 (7)	'AT/NAD-ME' (41, 42)
Amaranthus hypochondriacus L.	T	MA	R					I		I	I		I					I	I	I				-12.2 (7)	'AT/NAD-ME' (41, 42)
Amaranthus muricatus (Moq.) Hieron.	T	BR, AN	R							Ii	Ii													- 12.7 (7)	'AT/NAD-ME' (41, 42)
Amaranthus palmeri S. Watson	T	MA, CR	R							Ii	Ii							Ii		Ii				- 12.6 (7)	'AT/NAD-ME' (41, 42)
Amaranthus retroflexus L.	T	MA, CR	R	Ii	Ii	Ii		Ii	Ii	Ii	Ii		Ii		Ii			Ii	Ii	Ii	Ii			- 13.3 (7)	AT/NAD-ME (41, 42)
Amaranthus sparganicephalus Thell.	T	SM	R-Tr											Ne									Ne		'AT/NAD-ME' (41, 42)
Amaranthus spinosus L.	T	PL	R	Ii				Ii		Ii					Ii		Ii			Ii		Ii	Ii	- 12.6 (7)	'AT/NAD-ME' (41, 42)
Amaranthus standleyanus Parodi ex Covas	T	BR, CP	R																	Ii				-13.2 (7)	'AT/NAD-ME' (41, 42)
Amaranthus tenuifolius Willd.	T	I	R												N									-12.9(7)	'AT/NAD-ME' (41, 42)

C_4 lineage **(bold)** and species	Life form	Choro-type	Ecotype	Distribution in Southwest Asia																				δ13C	Leaf anatomy, C_4 Photosynthetic subtype (references)
				Afghanistan	Armenia	Azerbaijan	Bahrain	Iran	Iraq	Israel/Palestine	Jordan	Kuwait	Lebanon	Oman	Pakistan	Qatar	Saudi Arabia	Sinai	Syria	Turkey	Turkmenistan	UAE	Yemen		
Amaranthus tricolor L.	T	I, IC	R						I						I		I			I			I	-13.6 (7)	'AT/NAD-ME' (41, 42)
Amaranthus tuberculatus (Moq.) J. D. Sauer	T	NA	R							Ii	Ii													- 12.6, - 13.7 [21](7)	'AT/NAD-ME' (41, 42)
Amaranthus viridis L.	T	PL	R	Ii			Ii		Ii	Ii	Ii	Ii	Ii	Ii	Ii	Ii	Ii	Ii	Ii	Ii		Ii	Ii	-13.9 (7)	'AT/NAD-ME' (41, 42)
**28) *Gomphreneae***																									
Gomphrena celosoides Mart.	H	AM, Br	R												I								I	-14.3 (7)	'AT/NADP-ME' (41, 42)
Gomphrena globosa L.	T	AM, BR, CR	R						I						I		I						I	-14.9(7)	AT/NADP-ME (41, 42)
**Chenopodiaceae**																									
**29) C₄ *Atriplex***																									
Atriplex canescens (Pursh) Nutt.	Ch	MA, NA	XH					I							I									-15.6 (9)	AT/NAD-ME (41)
Atriplex coriacea Forssk.	Ch	M, SS	PH-L-Tr														N	N					N	-15.6 (8)	'AT/NAD-ME' (41)
Atriplex dimorphostegia Kar. & Kir.	T	SS, IT, CA	HL	N				N		N	N	N			N		N	N	N		N			-11.66 (8)	AT/NAD-ME (42)
Atriplex flabellum Bunge ex Boiss.	T	IT	HL-I-Tm	N				N													N			-14.06(8)	'AT/NAD-ME' (41)
Atriplex fominii Iljin	T	ES, IT	PH-L-Tm			N																		-12.46 (*)	'AT/NAD-ME' (41)
Atriplex glauca L.	Ch	M, SS	PS							N							N	N						-12.9 (8)	'AT/NAD-ME' (41)
Atriplex griffithii Moq.	Ch	IT	XH-I-Tm	Ne				Ne							Ne									-14.24 (8)	'AT/NAD-ME' (41)
Atriplex halimus L.	Ch	M, SS	XH					N		N	N	N	N		N		N	N	N	N			N	-14.3 (8)	AT/NAD-ME (42)
Atriplex laciniata L.	Ch	ES	XE, R-Tm		N																			-11.44 (8)	'AT/NAD-ME' (41)
Atriplex lasiantha Boiss.	T	IT, M	R-Tm	N				N		N	N		N		N				N	N				-12.34 (8)	'AT/NAD-ME' (41)
Atriplex leucoclada Boiss. (incl. *A. turcomanica* Fisch. & C.A.Mey. ex Kar.)	H or Ch	IT	HL-I-Tm	N		N	N	N	N	N	N	N		N	N	N	N	N	N	N		N	N	-13.54 (8)	AT/NAD-ME (41, 82)
Atriplex moneta Bunge ex Boiss.	T	IT	XH-I-Tm	N				N													N			- 13.71 (8)	'AT/NAD-ME' (41)
Atriplex ornata Iljin	T	CA	HL-I-Tm					Ne													Ne			-12.79 (8)	'AT/NAD-ME' (41)
Atriplex pamirica Iljin	T	IT	XE-I-Tm	N											N									-12.09 (8)	'AT/NAD-ME' (41)
Atriplex pungens Trautv.	T	IT	XH																	N					'AT/NAD-ME' (41)
Atriplex repens Roth	T or Ch	ES, IT, M	R	N				N	N												N			-12.65 (8)	'AT/NAD-ME' (41)
Atriplex rosea L.	T	CA	XE-I-Tm		N															N				-13.40(8)	AT/NAD-ME (41)
Atriplex schugnanica Iljin	T	SS, SM	XH-L-Tr	N											N									-12.58 (8)	'AT/NAD-ME' (41)
Atriplex stocksii Boiss.	Ch	PL	XH,R											Ne	Ne								Ne	-14.23 (*)	'AT/NAD-ME' (41)
Atriplex tatarica L.	T	M	PH-L	N	N	N		N		N	N		N		N		N	N		N	N	N		-13.67(8)	AT/NAD-ME (41, 78)
Atriplex tornabenei Tineo	T	IT, SS	HL																	N					'AT/NAD-ME' (41)
Atriplex zahlensis Mouterde	T	M	XH,R-Tm										Ne						Ne						'AT/NAD-ME' (41)
**30) *Bassia***																									
Bassia arabica (Boiss.) Maire & Weiller in Maire	H	SS	XE-I-Tr							N	N						N							-12.5 (*)	KC/'NADP-ME'(42, 80, 10)
Bassia eriantha (Fisch. & C. A. Mey.) Kuntze	T	IT	HL-Tm	N				N		N	N				N		N	N			N			-12.80(8)	AT /NADP-ME (44, 78, 10)
Bassia eriophora (Schrad.) Aschers.	T	SS	HL, XH-Tr	N				N	N	N	N	N			N		N		N					-13.42(8)	KC/NADP-ME (44, 10)

C_4 lineage **(bold)** and species	Life form	Choro-type	Ecotype	Distribution in Southwest Asia																				δ¹³C	Leaf anatomy, C_4 Photosynthetic subtype (references)
				Afghanistan	Armenia	Azerbaijan	Bahrain	Iran	Iraq	Israel/Palestine	Jordan	Kuwait	Lebanon	Oman	Pakistan	Qatar	Saudi Arabia	Sinai	Syria	Turkey	Turkmenistan	UAE	Yemen		
Bassia hyssopifolia (Pall.) O.Kuntze	T	IT, SS	HL-Tm	N	N	N		N							N		N				N			-12.9(8)	KC/NADP-ME (42, 80)
Bassia indica (Wight) A.J. Scott	T	SS, I	R-Tr	N						N					N		N	N						-13.0 (8)	KC/'NADP-ME' (42, 80)
Bassia laniflora (S.G. Gmel.) A.J. Scott	T	ES, IT, CA	PS-Tm			N																		-11.37(8)	KC/NADP-ME(42, 24, 10)
Bassia lasiantha Freitag & G. Kadereit	T	IT, CA	XE-I-Tm	N																	N			-12.2(10)	AT/'NADP-ME'(42, 80)
Bassia muricata (L.) Aschers.	T	SS	PH-I-Tr				N	N		N	N	N		N		N	N	N	N			N	N	-12.21(8)	KC/'NADP-ME'(42, 80)
Bassia pilosa (Fisch. & C.A. Mey.) Freitag & G. Kadereit (incl. *B. monticola* (Boiss.) Kuntze)	T	IT	XH-I-Tm	N	N	N		N		N			N		N				N	N				-12.88(8)	KC/'NADP-ME'(42, 80)
Bassia prostrata (L.) A.J. Scott	H	PL	XE-I-Tm	N	N	N		N							N					N	N			-13.44(8)	KC/NADP-ME(42, 41, 80)
Bassia scoparia (L.) A.J. Scott	T	PL	R-I-Tm	N	N	N		N				N			N		N			N	N			-13.4 (8)	AT/NADP-ME(42, 80)
Bassia stellaris (Moq.) Bornm. (incl. *B. odontoptera* (Schrenk) Freitag & G. Kadereit)	T	IT, CA	R-I-Tm	N	N			N							N						N			-13.43 (+)	KC/'NADP-ME'(42, 80)
**31) *Camphorosma***																									
Camphorosma monspeliaca L.	H	PL	XH-I-Tm	N	N	N		N							N					N	N			-13.51(8)	SL/NADP-ME(42, 78)
**32) *C4 Tecticornia***																									
*Tecticornia indica (*Willd.) K.A.Sheph. & Paul G.Wilson	Ch, H	PL	HL-L-Tr												N									-14.22 (8)	KT/NAD-ME (45)
**33) *Caroxyleae***																									
Caroxylon abarghuense (Assadi) Akhani & Roalson	Ch, P	IT	HL-I-Tm					Ne																-11.35(8)	'SL/NAD-ME' (41,24)
Caroxylon arabicum (Botsch.) Akhani & Roalson	Ch	SS	XE-I-Tr														Ne							-10.93 (*)	'SL/NAD-ME' (41,24)
Caroxylon canescens (Moq.) Akhani & Roalson	H	IT	XE-I-Tm	N				N	N						N				N	N				-13.24(8)	'SL/NAD-ME' (41,24)
Caroxylon chorassanicum (Botsch.) Akhani & Roalson	T	SS, IT	XH-I-Tm	Ne				Ne							Ne									-12.87(8)	'SL/NAD-ME' (41,24)
Caroxylon cyclophyllum (Baker) Akhani & Roalson	H, Ch	SS	XE-I-Tr				N	N	N			N		N	N	N	N					N	N	-14.2 (8)	'SL/NAD-ME' (41,24)
Caroxylon dendroides (Pall.) Tzvelev	H	IT	XH-I-Tm	N	N	N		N	N											N	N			-13.92(8)	SL/NAD-ME (41,24, 84)
Caroxylon ericoides (M. Bieb.) Akhani & Roalson	Ch	IT	XH-I-Tm		N	N		N												N				-12.9(9)	'SL/NAD-ME' (41,24)
Caroxylon forcipitatum (Iljin) Akhani & Roalson	T	IT	XE-I-Tm	N																	N			-13.6(9)	'SL/NAD-ME' (41,24)
Caroxylon gemmascens (Pall.) Tzvelev	H	IT	PH-I-Tm	N	N			N													N			-12.44(8)	'SL/NAD-ME' (41,24)
Caroxylon iljinii (Botsch.) Akhani	H	IT	XE-I-Tm					Ne													Ne			-13.75 (*)	'SL/NAD-ME' (41,24)
Caroxylon imbricatum (Forssk.) Akhani & Roalson	H, Ch	SS	XH, R-L-Tr	N			N	N		N	N	N		N	N	N	N					N	N	-14.2 (8)	'SL/NAD-ME' (41,24)
Caroxylon incanescens (C. A. Mey.) Akhani & Roalson	T	PL	XH-I-Tm	N		N		N	N						N		N			N	N		N	-11.8(8)	'SL/NAD-ME' (41,24)

C_4 lineage **(bold)** and species	Life form	Choro-type	Ecotype	Distribution in Southwest Asia																				δ^{13}C	Leaf anatomy, C_4 Photosynthetic subtype (references)
				Afghanistan	Armenia	Azerbaijan	Bahrain	Iran	Iraq	Israel/Palestine	Jordan	Kuwait	Lebanon	Oman	Pakistan	Qatar	Saudi Arabia	Sinai	Syria	Turkey	Turkmenistan	UAE	Yemen		
Caroxylon inerme (Forssk.) Akhani & Roalson	T	SS, IT	HL-Tr					N	N	N	N						N	N	N	N				-13.62(8)	'SL/NAD-ME' (41,24)
Caroxylon jordanicola (Eig) Akhani & Roalson	T	SS	HL-Tr				Ne	Ne	Ne	Ne	Ne	Ne				Ne	Ne	Ne	Ne			Ne		-11.9(8)	'SL/NAD-ME' (41,24)
Caroxylon laricinum (Pall.) Tzvelev	Ch	IT	XH-I-Tm																	N				-14.6(9)	SL/NAD-ME(41,24, 85)
Caroxylon nitrarium (Pall.) Akhani & Roalson	T	IT, SS	XH, R	N	N	N		N	N						N		N			N	N			-13.3(8)	'SL/NAD-ME' (41,24)
Caroxylon nodulosum Moq. (incl. *Salsola verrucosa* M.Bieb.)	H	IT	XH-I-Tm		N	N		N												N				-12.8(8)	'SL/NAD-ME' (41,24)
Caroxylon omanense (Boulos) Akhani & Rudov (Syn. *Salsola omanensis* Boulos)	Ch	SS	PH-L-Tm											Ne										-11.55 (*)	'SL/NAD-ME' (41,24)
Caroxylon orientale (S. G. Gmel.) Tzvelev	H	IT	XH-Tm	N		N		N							N						N			-13.61(8)	'SL/NAD-ME' (41,24)
Caroxylon persicum (Bunge ex Boiss.) Akhani & Roalson	H	IT	XH-I-Tm					Ne																	'SL/NAD-ME' (41,24)
Caroxylon scleranthum (C. A. Mey.) Akhani & Roalson (incl. *C. implicatum* (Botsch.) Akhani & Roalson)	T	IT	XH-I-Tm	N				N							N						N			-11.7(8)	'SL/NAD-ME' (41,24)
Caroxylon spinescens (Moq.) Akhani & Roalson	Ch	SS	XE-Tr											N			N						N	-12.11 (*)	'SL/NAD-ME' (41,24)
Caroxylon stenopterum (Wagenitz) Akhani & Roalson (incl. *Salsola anatolica* Aellen)	T	IT	XH-I-Tm																	Ne				-13.0(9)	'SL/NAD-ME' (41,24)
Caroxylon tetrandrum (Forssk.) Akhani & Roalson	Ch	SS	XH-I-Tr							N	N						N							-13.2(8)	'SL/NAD-ME' (41,24)
Caroxylon turkestanicum (Litw.) Akhani & Roalson (incl. *Salsola leptoclada* Gand.)	T	IT	XH-I-Tm	N				N							N						N			-13.45 (*)	'SL/NAD-ME' (41,24)
Caroxylon vermiculatum (L.) Akhani & Roalson (incl. *C. camphorosmoides* (Iljin) Sukhor.)	H	IT, SS	XE-I-Tm		N	N		N	N	N	N						N			N				-13.29(8)	'SL/NAD-ME' (41,24)
Caroxylon villosum (Schult.) Akhani & Roalson	Ch	SS	XE-I-Tr														N							-11.6(9)	'SL/NAD-ME' (41,24)
Caroxylon volkensii (Aschers & Schweinf.) Akhani & Roalson	T	SS	XE, R-I-Tr						N	N	N						N	N						-14.0(9)	'SL/NAD-ME' (41,24)
Caroxylon yazdianum (Assadi) Akhani & Roalson	H	IT	XH-I-Tm					Ne																-12.5(8)	'SL/NAD-ME' (41,24)
Cimacoptera iraqensis Botsch.	T	SS	HL-Tr						Ne																'SL/NAD-ME' (41,24)
Climacoptera afghanica Botsch.	T	IT	HL-I-Tm	Ne											Ne									-13.24 (*)	'SL/NAD-ME' (41,24)
Climacoptera botschantzevii Pratov	T	IT	HL-I-Tm					Ne																-11.67 (*)	'SL/NAD-ME' (41,24)
Climacoptera bucharica (Ilin) Botsch.	T	IT	HL-I-Tm																		N			-12.54 (*)	'SL/NAD-ME' (41,24)
Climacoptera chorassanica Pratov	T	IT	HL-I-Tm					Ne																	'SL/NAD-ME' (41,24)
Climacoptera crassa (M. Bieb.) Botsch.	T	IT	HL		N	N		N		N										N				-11.0(8)	SL/NAD-ME(41,24, 80)
Climacoptera czelekenica Pratov	T	IT	HL-L-Tm																		Ne			-11.45 (*)	'SL/NAD-ME' (41,24)
Climacoptera ferganica (Drob.) Botsch.	T	IT	HL-I-Tm	N				N													N			-12.06 (*)	'SL/NAD-ME' (41,24)
Climacoptera glaberrima Botsch.	T	IT	HL-I-Tm					N													N			-12.99 (*)	'SL/NAD-ME' (41,24)

C_4 lineage **(bold)** and species	Life form	Choro-type	Ecotype	Distribution in Southwest Asia																				δ13C	Leaf anatomy, C_4 Photosynthetic subtype (references)
				Afghanistan	Armenia	Azerbaijan	Bahrain	Iran	Iraq	Israel/Palestine	Jordan	Kuwait	Lebanon	Oman	Pakistan	Qatar	Saudi Arabia	Sinai	Syria	Turkey	Turkmenistan	UAE	Yemen		
Climacoptera iranica Pratov	T	IT	HL-I-Tm					Ne												Ne				-13.65 (*)	'SL/NAD-ME' (41,24)
Climacoptera khalisica Botsch.	T	IT	HL-L						Ne																'SL/NAD-ME' (41,24)
Climacoptera lanata (Pall.) Botsch.	T	IT	HL	N				N													N			-14.64(8)	SL/NAD-ME'(41,24, 80)
Climacoptera longipistillata Botsch.	T	IT	HL	Ne				Ne																-14.53 (*)	'SL/NAD-ME' (41,24)
Climacoptera longistylosa (Iljin) Botsch.	T	IT	HL	N																	N			-12.94 (*)	'SL/NAD-ME' (41,24)
Climacoptera maimanica (Freitag) Akhani	T	IT	HL-I-Tm	Ne																				-11.69 (*)	'SL/NAD-ME' (41,24)
Climacoptera sukaczevii Botsch.	T	IT	HL-I																		N			-12.04 (*)	'SL/NAD-ME' (41,24)
Climacoptera transoxana (Iljin) Botsch.	T	IT	HL-I	N																	N			-14.38 (*)	'SL/NAD-ME' (41,24)
Climacoptera turcomanica (Litw.) Botsch.	T	IT	HL	N				N	N						N				N		N			-13.34(8)	SL/NAD-ME (41,24, 82)
Climacoptera zenobiae (Mouterde) Botsch.	T	IT	HL-I-Tm																Ne					-13.20 (*)	'SL/NAD-ME' (41,24)
Halarchon vesciculosus (Moq.) Bunge	T	IT	XH-I-Tm	Ne																				-9.27(8)	'SL/NAD-ME' (41,24)
Halimocnemis alaeflava (Assadi) Akhani	T	IT	HL-I-Tm					Ne																-12.65 (*)	'SL/NAD-ME' (41,24)
Halimocnemis azarbaijanensis Assadi	T	IT	HL-I-Tm					Ne																-14.03 (*)	'SL/NAD-ME' (41,24)
Halimocnemis beresinii Iljin	T	IT	HL-I-Tm	N																	N			-12.40 (*)	'SL/NAD-ME' (41,24)
Halimocnemis commixta (Bunge) Akhani	T	IT	HL-I-Tm	Ne																	Ne			-11.71 (*)	'SL/NAD-ME' (41,24)
Halimocnemis gamocarpa Moq.	T	IT	HL-I-Tm	N				N							N						N			-12.76(8)	'SL/NAD-ME' (41,24)
Halimocnemis glaberrima Iljin	T	IT	HL-I-Tm																		N			-10.56 (*)	'SL/NAD-ME' (41,24)
Halimocnemis karelinii Moq.	T	IT	HL-I-Tm																		N			-13.43 (*)	'SL/NAD-ME' (41,24)
Halimocnemis kulpiana C. Koch	T	IT	GP-I-Tm			Ne														Ne				-13.0(8)	'SL/NAD-ME' (41,24)
Halimocnemis longifolia Bunge	T	IT	HL-I-Tm					N													N			-13.96 (*)	'SL/NAD-ME' (41,24)
Halimocnemis macrantha Bunge	T	IT	HL-Tm																		N			-9.33 (*)	'SL/NAD-ME' (41,24)
Halimocnemis mamamense (Bunge) Assadi	T	IT	HL-I-Tm					Ne																-13.41 (*)	'SL/NAD-ME' (41,24)
Halimocnemis mollissima Bunge	T	IT	HL-I-Tm	N				N													N			-12.46(8)	SL/NAD-ME (41,24, 82)
Halimocnemis occulta (Bunge) Hedge	T	IT	HL-I-Tm					Ne							Ne									-12.26(8)	'SL/NAD-ME' (41,24)
Halimocnemis pedunculata (Assadi) Akhani	T	SS	HL-I-Tr					Ne																-14.07 (*)	'SL/NAD-ME' (41,24)
Halimocnemis pilifera Moq.	T	IT	HL-I-Tm		N	N		N	N	N	N										N			-12.52(8)	SL/NAD-ME (41,24, 82)
Halimocnemis pilosa (Pall.) Akhani	T	IT	HL-I-Tm		Ne	Ne		Ne																-12.9(8)	'SL/NAD-ME' (41,24)
Halimocnemis purpurea Moq	T	IT	XH-I-Tm	Ne				Ne	Ne															-12.32(8)	'SL/NAD-ME' (41,24)
Halimocnemis rarifolia (C. Koch) Akhani	T	IT	HL-I-Tm		N	N		N												N				-13.65(8)	SL/NAD-ME (41,24, 82)
Halimocnemis rosea (Trautv.) Akhani	T	IT	HL-I-Tm																	Ne				-11.72 (*)	'SL/NAD-ME' (41,24)
Halimocnemis sclerosperma (Pall.) C. A. Mey.	T	IT	XH-I-Tm		N	N															N			-9.57 (*)	'SL/NAD-ME' (41,24)
Halimocnemis smirnowii Bunge	T	IT	HL-I-Tm																		N			-10.17 (*)	'SL/NAD-ME' (41,24)
Halimocnemis villosa Kar. et Kir.	T	IT	HL-I-Tm																		N			-12.3 (9)	'SL/NAD-ME' (41,24)
Halocharis brachyura Eig	T	IT	HL-I-Tm						Ne															-12.5(9)	'SL/NAD-ME' (41,24)
Halocharis clavata Bunge	T	IT	HL-I-Tm	Ne											Ne										'SL/NAD-ME' (41,24)
Halocharis hispida (Schrenk) Bunge	T	IT	XH-I-Tm	N				N							N						N			-13.8(8)	'SL/NAD-ME' (41,24)
Halocharis lachnantha E.Korov	T	IT	HL-I-Tm	N																	N				'SL/NAD-ME' (41,24)

C_4 lineage **(bold)** and species	Life form	Choro-type	Ecotype	Distribution in Southwest Asia																				δ^{13}C	Leaf anatomy, C_4 Photosynthetic subtype (references)
				Afghanistan	Armenia	Azerbaijan	Bahrain	Iran	Iraq	Israel/Palestine	Jordan	Kuwait	Lebanon	Oman	Pakistan	Qatar	Saudi Arabia	Sinai	Syria	Turkey	Turkmenistan	UAE	Yemen		
Halocharis sulphurea (Moq.) Moq.	T	IT	HL	Ne				Ne	Ne						Ne		Ne		Ne					-12.88(8)	'SL/NAD-ME' (41,24)
Halocharis turcomanica Iljin	T	IT	HL-I-Tm	Ne																	Ne			-13.41 (*)	'SL/NAD-ME' (41,24)
Halocharis violacea Bunge	T	IT	HL-I	Ne				Ne							Ne									-11.07 (*)	'SL/NAD-ME' (41,24)
Kaviria aucheri (Moq.) Akhani	H, Ch	IT	XE-I-Tm	Ne				Ne													Ne			-13.71(8)	'SL/NAD-ME' (41,24)
Kaviria azaurena (Mouterde) Sukhor.	H, Ch	SS	GP-I-Tr						Ne															-13.93 (*)	'SL/NAD-ME' (41,24)
Kaviria cana (C. Koch) Akhani	H	IT	XE-I-Tm		N	N		N																-13.5(9)	'SL/NAD-ME' (41,24)
Kaviria futilis (Iljin) Akhani	H	IT	XE-I-Tm			Ne		Ne																-9.54 (*)	'SL/NAD-ME' (41,24)
Kaviria gossypina (Bunge) Akhani	T	IT	XE-I-Tm	N				N																-14.59 (8)	'SL/NAD-ME' (41,24)
Kaviria lachnantha (Botsch.) Akhani	H	SS	XH-I-Tr					Ne	Ne															-13.96(8)	'SL/NAD-ME' (41,24)
Kaviria rubescens (Franch.) Akhani	H	SS	XE-I-Tr											N	N							N	N	-14.15(8)	'SL/NAD-ME' (41,24)
Kaviria tomentosa (Moq.) Akhani	H	IT	XH-I-Tm	N	N	N		N	N						N						N			-13.36(8)	'SL/NAD-ME' (41,24)
Kaviria vvedenskyi (Iljin & M. Pop.) Akhani	T	IT	XH-I-Tm	N																	N			-12.5(8)	'SL/NAD-ME' (41,24)
Kaviria zehzadii (Akhani) Akhani	H	IT	GP-I					Ne																-10.72(8)	'SL/NAD-ME' (41,24)
Petrosimonia brachiata (Pall.) Bunge	T	IT	HL-Tm		N	N		N												N				-13.43(8)	'SL/NAD-ME' (41,24)
Petrosimonia glauca (Pall.) Bunge	T	IT	HL-I		N			N													N			-13.69(8)	SL/NAD-ME (41,24, 82)
Petrosimonia nigdeensis Aellen	T	IT	HL-I-Tm																	Ne				-12.2(9)	'SL/NAD-ME' (41,24)
Petrosimonia oppositifolia (Pall.) Litv.	T	IT	HL-I-Tm			N																		-14.39 (*)	'SL/NAD-ME' (41,24)
Petrosimonia sibirica (Pall.) Bunge	T	IT	HL-I-Tm	N																	N			-13.17 (*)	'SL/NAD-ME' (41,24)
Petrosimonia squarrosa (Schrenk) Bunge	T	IT	HL-I-Tm																	N					'SL/NAD-ME' (41,24)
Petrosimonia triandra (Pall.) Simonkai	T	IT	HL-Tm			N		N																-14.44 (*)	'SL/NAD-ME' (41,24)
Piptoptera turkestana Bunge	T	IT	PS-I-Tm	N				N													N			-12.33 (8)	'SL/NAD-ME' (41,24)
Pyankovia brachiata (Pall.) Akhani & Roalson	T	IT	HL-I-Tm	N				N													N			-12.24 (8)	'SL/NAD-ME' (41,24)
**34) *Nanophyton***																									
Nanophyton erinaceum (Pall.) Bunge	Ch	IT	XE-I-Tm																		N			-13.00 (*)	'SL/NAD-ME' (41,24)
**35) *C4 Salsoleae***																									
Anabasis annua Bunge	T	IT	XH-I-Tm	N				N	N												N			-13.1 (9)	SL/NADP-ME(41, 24)
Anabasis aphylla L.	Ch	IT	XH-I-Tm		N	N		N												N	N			-12.39 (11)	'SL/NADP-ME'(41, 24)
Anabasis articulata (Forssk.) Moq.	Ch	SS	XH-I				N		N	N	N						N							-12.4 (11)	'SL/NADP-ME'(41, 24)
Anabasis brachiata Fisch. & C.A.Mey. ex Kar. & Kir.	Ch	IT	XE-I-Tm			N															N			-12.89 (8)	'SL/NADP-ME'(41, 24)
Anabasis calcarea (Sharif & Aellen) Bokhari & Wendelbo	H	IT	XH-I-Tm					Ne																-11.48(11)	'SL/NADP-ME'(41, 24)
Anabasis ebracteolata Korov. ex Botsch.	H	IT	XE-I-Tm																		N			-12.29 (8)	'SL/NADP-ME'(41, 24)
Anabasis ehrenbergii Schweinf. ex Boiss.	Ch	SS	HL-L-Tr														N						N	-12.8(9)	'SL/NADP-ME'(41, 24)
Anabasis eriopoda (Schrenk) Benth. ex Volkens	H	IT	XE-I-Tm	N				N													N			-12.96 (11)	SL/NADP-ME(41, 24)
Anabasis eugeniae Iljin	H	IT	GP-I-Tm		Ne	Ne		Ne																-9.8 (11)	'SL/NADP-ME'(41, 24)

C_4 lineage **(bold)** and species	Life form	Choro-type	Ecotype	Distribution in Southwest Asia																				δ13C	Leaf anatomy, C_4 Photosynthetic subtype (references)
				Afghanistan	Armenia	Azerbaijan	Bahrain	Iran	Iraq	Israel/Palestine	Jordan	Kuwait	Lebanon	Oman	Pakistan	Qatar	Saudi Arabia	Sinai	Syria	Turkey	Turkmenistan	UAE	Yemen		
Anabasis firouzii Akhani	H	IT	XE-I-Tm					Ne																-10.08 (11)	'SL/NADP-ME'(41, 24)
Anabasis haussknechtii Bunge ex Boiss.	H	IT	XH-I-Tm					Ne							Ne									-12.44 (11)	'SL/NADP-ME'(41, 24)
Anabasis iranica Iljin	H	IT	XH-I-Tm					Ne																-10.62 (*)	'SL/NADP-ME'(41, 24)
Anabasis jaxartica (Bunge) Benth ex Volkens	H	IT	XE-I-Tm					N													N			-13.49(11)	'SL/NADP-ME'(41, 24)
Anabasis lachnantha Aellen & Rech. f.	Ch	SS	XE-I					Ne	Ne	Ne	Ne	Ne			Ne		Ne							-11.3 (11)	'SL/NADP-ME'(41, 24)
Anabasis macroptera Moq.	H	IT	XE-I-Tm	Ne																				-11.09 (11)	'SL/NADP-ME'(41, 24)
Anabasis oropediorum Maire	Ch	IT	XE-I-Tm							N								N						-15.2 (11)	'SL/NADP-ME'(41, 24)
Anabasis salsa (C. A. Mey.) Benth. ex Volkens	Ch	IT	XH-I-Tm			N		N													N			-11.56 (11)	SL/NADP-ME(41, 24)
Anabasis setifera Moq.	H or T	IT, SS	XH-I-Tm	N			N	N	N	N	N	N		N	N	N	N	N				N		-12.53 (11)	'SL/NADP-ME'(41, 24)
Anabasis syriaca Iljin	H	SS	GP-I-Tm							N	N		N						N					-13.0 (11)	'SL/NADP-ME'(41, 24)
Anabasis truncata (Schrenk) Bunge	H	IT	XH-I-Tm																		N			-11.39 (*)	'SL/NADP-ME'(41, 24)
Anabasis turkestanica Iljin & Korov.	H	IT	XH-I-Tm	N																	N			-12.01 (11)	'SL/NADP-ME'(41, 24)
Arthrophytum gracile Aellen	H	IT	O-I-Tm	Ne																				-13.21(8)	'SL/NADP-ME'(41)
Arthrophytum lehmannianum Bunge	CH	IT	XH-I-Tm																		N			– 12.78 (13)	'SL/NADP-ME'(41)
Cornulaca aucheri Moq.	T or H	SS, IT	PS	N			N	N	N			N		N	N	N	N		N	N				-12.62(8)	'SL/NADP-ME'(41)
Cornulaca ehrenbergii Asch.	Ch	SM, SS	PH-L-Tr														N						N	-12.16 (*)	'SL/NADP-ME'(41)
Cornulaca korshinskyi Litv.	T	IT	PH-I-Tm																		N			-11.4 (8), -10.58 (*)	'SL/NADP-ME'(41)
Cornulaca monacantha Delile	Ch	SS	XH	N				N	N	N	N	N		N	N	N	N	N				N	N	-13.23(8)	'SL/NADP-ME'(41)
Cornulaca setifera (DC.) Moq.	Ch	SS	XE						Ne				Ne				Ne		Ne					-12.4(8)	'SL/NADP-ME'(41)
Girgensohnia imbricata Bunge	T	IT	XE-I-Tm					Ne																-13.08 (*)	'SL/NADP-ME'(41)
Girgensohnia minima E. Korov	T	IT	XE-I-Tm	Ne				Ne													Ne				'SL/NADP-ME'(41)
Girgensohnia bungeana Sukhor.	T	IT	XE-I-Tm	N				N																-11.35 (*)	'SL/NADP-ME'(41)
Girgensohnia diptera Bunge	T	IT	XE-I-Tm	N																				-11.79 (*)	'SL/NADP-ME'(41)
Girgensohnia fruticulosa Bunge (*Cyathobasis fruticulosa* (Bunge) Aellen)	H	IT	XH-I-Tm																	Ne				-13.4(8)	'SL/NADP-ME'(41)
Girgensohnia oppositiflora (Pall.) Fenzl.	T	IT, SS	XE-I-Tm	N	N	N		N	N	N					N						N			-13.2(8)	SL/NADP-ME(41,80)
Halogeton alopecuroides (Delile) Moq.	H	SS	XH-I-Tr						N	N	N	N	N		N		N	N	N					-14.3(9)	'SL/NADP-ME'(41)
Halogeton glomeratus (M. Bieb.) C. A. Mey.	T	IT, CA	XE-I-Tm	N											N						N			-11.33(8)	SL/NADP-ME(41, 83)
Halogeton tibeticus Bunge	T	IT,CA	XE-I-Tm	N											N										'SL/NADP-ME'(41)
Halothamnus afghanicus Kothe-H.	Ch	SS	XE-I-Tm	Ne																					'SL/NADP-ME'(41)
Halothamnus auriculus (Moq.) Botsch.	H	IT	XH, GP-I-Tm	N				N							N						N			-13.06(8)	SL/NADP-ME(41, 42)
Halothamnus bamianicus (Gilli) Botsch.	Ch	IT	XE-I-Tm	Ne																				-12.32(8)	'SL/NADP-ME'(41)
Halothamnus bottae Jaub. & Spach	Ch	SS	O-I-Tm											Ne			Ne					Ne	Ne	-13.4(8)	'SL/NADP-ME'(41)
Halothamnus cinerascens Moq.	Ch	IT	XH-I-Tm					Ne																	'SL/NADP-ME'(41)
Halothamnus glaucus (M. Bieb.) Botsch.	H, Ch	IT	XE-I-Tm		N	N		N												N	N			-12.9(8)	'SL/NADP-ME'(41)
Halothamnus hierochunticus (Bornm.) Botsch.	T	IT	XH, R-I-Tm					Ne	Ne	Ne	Ne		Ne						Ne					-12.56(8)	'SL/NADP-ME'(41)

				Distribution in Southwest Asia																					
C_4 lineage **(bold)** and species	Life form	Choro-type	Ecotype	Afghanistan	Armenia	Azerbaijan	Bahrain	Iran	Iraq	Israel/Palestine	Jordan	Kuwait	Lebanon	Oman	Pakistan	Qatar	Saudi Arabia	Sinai	Syria	Turkey	Turkmenistan	UAE	Yemen	δ13C	Leaf anatomy, C_4 Photosynthetic subtype (references)
Halothamnus iliensis (Lipsky) Botsch.	T	IT	XH-I-Tm	N																	N			-12.46 (8), -12.8 (9)	'SL/NADP-ME'(41)
Halothamnus iranicus Botsch.	H, Ch	SS	XH-I-Tm					Ne							Ne									-12.79 (*)	'SL/NADP-ME'(41)
Halothamnus iraqensis Botsch.	Ch	SS	XH-I-Tm					Ne	Ne			Ne					Ne		Ne						'SL/NADP-ME'(41)
Halothamnus kermanensis Kothe-Heinr.	H	IT	HL-I-Tm					Ne																	'SL/NADP-ME'(41)
Halothamnus lancifolius (Boiss.) Kothe-H.	Ch	IT, SS	GP-I-Tm						Ne	Ne	Ne						Ne		Ne					-11.8(8)	'SL/NADP-ME'(41)
Halothamnus oxianus Botsch.	Ch	IT	XE-I-Tm	N																				-11.26 (*)	'SL/NADP-ME'(41)
Halothamnus schurobi Botsch.	Ch	IT	GP-I-Tm	Ne																				-12.43(8)	'SL/NADP-ME'(41)
Halothamnus sistanicus (De Marco & Dinelli) Kothe-Heinr.	Ch	IT	XE-I-Tm					Ne																	'SL/NADP-ME'(41)
Halothamnus subaphyllus (C. A. Mey.) Botsch.	Ch	IT	XH-I-Tm	N				N							N						N			-12.19(8)	'SL/NADP-ME'(41)
Halothamnus turcomanicus Botsch.	Ch	IT	XE-I-Tm																		Ne			- 11.2 (12)	'SL/NADP-ME'(41)
Haloxylon ammodendron (C. A. Mey.) Bunge ex Fenzl	P	IT	PH, XH-I	N				N													N			-13.2(8)	'SL/NADP-ME'(41)
Haloxylon persicum Bunge ex Boiss. & Buhse	P	IT, SS	PS-I	N				N	N	N	N			N	N		N	N			N	N		-12.6(8)	'SL/NADP-ME'(41)
Hammada articulata (Moq.) O. Bolтs & Vigo (incl. *H. scoparia* (Pomel) Iljin)	Ch	SS	PH-Tr						N	N	N		N					N	N						'SL/NADP-ME'(41)
Hammada eigii Iljin (incl. *H. ramosissima* (Boiss. ex Eig) Iljin)	Ch	IT, M	XE						Ne	Ne	Ne		Ne						Ne					-11.27 [22](*), -14.6(9)	'SL/NADP-ME'(41)
Hammada eriantha Botsch.	Ch	IT	XE-I-Tm																		N				'SL/NADP-ME'(41)
Hammada griffithii (Moq.) Iljin (incl. *H. leptoclada* (M.Pop. Ex Iljin) Iljin)	H	IT	PS, XE-I-Tm	Ne											Ne						Ne			-13.35(8), -13.22 (*)	'SL/NADP-ME'(41)
Hammada multiflora (Moq.) Iljin	H	IT	O-I-Tm	Ne																				-12.34(8)	'SL/NADP-ME'(41)
Hammada negevensis Iljin & Zohary	Ch	SS	GP, XE-I-Tr							Ne	Ne							Ne						-12.9(9)	'SL/NADP-ME'(41)
Hammada salicornica (Moq.) Iljin	Ch	SS	PS, XH-Tr	N			N	N	N	N	N	N		N	N	N	N	N				N		-13.68(8), -11.71 (*)	'SL/NADP-ME'(41)
Hammada schmittiana (Pomel) Botsch.	Ch	SS	XH-I-Tr							N	N							N						-13.6(8)	'SL/NADP-ME'(41)
Hammada thomsoni (Bunge) Iljin	H	CA	XE-I-Tm												N									-10.60 (*)	'SL/NADP-ME'(41)
Hammada wakhanica (Paulsen) Iljin	Ch	CA, IT	XH-I-Tm	N											N									-14.3(11)	'SL/NADP-ME'(41)
Horaninovia anomala (C. A. Mey.) Moq.	T	IT	PS-I-Tm					N													N			-14.0(8)	'SL/NADP-ME'(41)
Horaninovia minor Schrenk	T	IT	HL-I-Tm																		N			-11.00 (*)	'SL/NADP-ME'(41)
Horaninovia platypetra Charif & Aellen	T	IT	PS-I-Tm					Ne																-13.22(8)	'SL/NADP-ME'(41)
Horaninovia pungens (Gill.) Botsch.	T	IT	PS-I-Tm	N				N																-11.86(8)	'SL/NADP-ME'(41)
Horaninovia ulicina Fisch. & C.A.Mey.	T	IT	PH-I-Tm	N				N													N			-12.5(8)	'SL/NADP-ME'(41)
Iljinia regelii (Bunge) Korov.	H	IT	XE-I-Tm																		N			– 13.46 (13)	'SL/NADP-ME'(41)
Lagenantha cycloptera (Stapf) M.G. Gilbert & Friis	Ch	SM	PH-L-Tr																				N		SL/NADP-ME(41, 84)

C_4 lineage **(bold)** and species	Life form	Choro-type	Ecotype	Distribution in Southwest Asia																				δ^{13}C	Leaf anatomy, C_4 Photosynthetic subtype (references)
				Afghanistan	Armenia	Azerbaijan	Bahrain	Iran	Iraq	Israel/Palestine	Jordan	Kuwait	Lebanon	Oman	Pakistan	Qatar	Saudi Arabia	Sinai	Syria	Turkey	Turkmenistan	UAE	Yemen		
Noaea major Bunge	T	SS, IT	XE-I-Tm	Ne											Ne									-12.82(8)	'SL/NADP-ME'(41)
Noaea minuta Boiss. & Balansa	T	IT	O-I-Tm		Ne															Ne				-12.2(8)	'SL/NADP-ME'(41)
Noaea mucronata (Forssk.) Asch. & Schweinf. (incl. *N. leptoclada* (Woronow) Iljin and *N. cadmea* Yild.)	Ch, H	PL	XE-I-Tm	N	N	N		N	N	N	N						N			N	N			-14.14(8)	'SL/NADP-ME'(41)
Soda austro-iranica (Akhani) Akhani (Syn. *Salsola austro*-iranica Akhani)	T	SS	HL-L-Tr					Ne																-14.47 (*)	'SL/NADP-ME'(41)
Soda cyrenaica (Maire & Weiller) Akhani (Syn. *Salsola cyrenaica* (Maire & Weiller) Brullo)	H, Ch	M	HL-L-Tm																	N				-12.93 (*)	'SL/NADP-ME'(41)
Soda drummondii (Ulbr.) Akhani (Syn. *Salsola drummondii* Ulbrich)	Ch	SS	HL-Tr					N						N	N	N	N					N		-12.14(8)	'SL/NADP-ME'(41)
Soda florida (M. Bieb.) Akhani (Syn. *Salsola florida* (M. Bieb.) Poir)	T or H	IT	HL-I-Tm		Ne	Ne		Ne												Ne				-10.72(8)	'SL/NADP-ME'(41)
Soda foliosa (L.) Akhani (Syn. *Salsola foliosa* (L.) Schrad. ex Schult.)	T	IT	HL-I-Tm			N															N			-11.98 (8)	'SL/NADP-ME'(41)
Soda grandis (Freitag, Vural & N. Adiguzel) Akhani (Syn. *Salsola grandis* Freitag, Vural & N. Adiguzel)	T	IT	XE-I-Tm																	Ne				-11.31 (*)	'SL/NADP-ME'(41)
Soda cinerea (Moq.) Akhani (Syn. *Salsola kavirensis* Akhani)	T	IT, SS	HL-I-Tm					Ne	Ne		Ne				Ne				Ne					-12.34 (*)	'SL/NADP-ME'(41)
Soda kerneri (Wol.) Akhani (syn. *Salsola kerneri* (Wol.) Botsch.)	Ch	IT	XE-I-Tm					Ne																-12.9(8)	'SL/NADP-ME'(41)
Soda longifolia (Forssk.) Akhani (Syn. Salsola longifolia Forssk.)	Ch	SS	HL-Tr							N	N							N						-14.7(8)	'SL/NADP-ME'(41)
Soda makranica (Freitag) Akhani (Syn. *Salsola makranica* Freitag)	Ch	IT, SS	HL-Tr												Ne				Ne						'SL/NADP-ME'(41)
Soda oppositifolia (Desf.) Akhani (Syn. *Salsola oppositifolia* Desf.)	Ch	SS	HL-Tr							N								N						-11.14(8)	'SL/NADP-ME'(41)
Soda rosmarinus (Ehrenb. ex Boiss.) Akhani (Syn. *Salsola rosmarinus* (Ehrenb. ex Boiss.) Akhani)	Ch	IT, SS	XH-I	N			N	N	N	N	N			N	N		N	N	N		N		N	-12.53(8)	'SL/NADP-ME'(41)
Soda schweinfurthii (Solm.) Akhani (syn. *Salsola schweinfurthii* Solms-Laub.)	Ch	SS	HL-Tr							N	N			N			N							-14.1(8)	'SL/NADP-ME'(41)
Soda inermis Fourr. (Syn. *Salsola soda* L.)	T	PL	HL-Tm		N	N		N		N										N	N			-10.1(8)	'SL/NADP-ME'(41)
Soda stocksii (Boiss.) Akhani (Syn. *Salsola stocksii* Boiss.)	Ch	SS	HL-Tr	Ne				Ne							Ne									-11.46(8)	'SL/NADP-ME'(41)
Sevada schimperi Moq.	Ch	SM	HL-L-Tr											N			N						N	-13.28 (*)	'SL/NADP-ME'(41)

C_4 lineage **(bold)** and species	Life form	Choro-type	Ecotype	Distribution in Southwest Asia																				δ¹³C	Leaf anatomy, C_4 Photosynthetic subtype (references)
				Afghanistan	Armenia	Azerbaijan	Bahrain	Iran	Iraq	Israel/Palestine	Jordan	Kuwait	Lebanon	Oman	Pakistan	Qatar	Saudi Arabia	Sinai	Syria	Turkey	Turkmenistan	UAE	Yemen		
Traganum nudatum Delile	Ch	SS	XE-Tr						N	N	N	N	N			N	N	N	N					-10.89(8)	'SL/NADP-ME'(41)
Turania androssowii (Litv.) Akhani	T	IT	XH-T-Tm																		N			-12,00 (9)	'SL/NADP-ME'(41)
Turania aperta (Paulsen) Akhani	T	IT	PS-I-Tm	N				N													N			-12.04(8)	'SL/NADP-ME'(41)
Turania deserticola (Iljin) Akhani	T	IT	XE-I-Tm																		N			-11.52 (8)	'SL/NADP-ME'(41)
Turania sogdiana (Bunge) Akhani	T	IT	PH-I-Tm																		N			-10.45 (*)	'SL/NADP-ME'(41)
Turania x angusta (*T.aperta x T.sogdiana*)	T	IT	XE-L-Tm																		N			-9.36 (*)	'SL/NADP-ME'(41)
Xylosalsola arbuscula Pall.	Ch	IT	XH-I-Tm	N				N							N						N			-12.39(8)	SL/NADP-ME(41, 84)
Xylosalsola chiwensis (Popov) Akhani & Roalson	Ch	IT	XE, O-I-Tm																		N			-11.15 (*)	'SL/NADP-ME'(41)
Xylosalsola paletzkiana (Litv.) Akhani & Roalson	P	IT	PS-I-Tm	N																	N			-12.9(8)	'SL/NADP-ME'(41)
Xylosalsola richteri (Moq.) Kar. ex Litw.	P	IT	PS-I-Tm	N				N							N						N			-12.92(8)	'SL/NADP-ME'(41)
**36) *Salsola***																									
Salsola collina Pall. (Syn. *Kali collinum* (Pallas) Akhani & Roalson)	T	PL	XH, R-I-Tm	N	N																N			-11.62(8)	SL/NADP-ME(41, 86)
Salsola griffithii (Bunge) Freitag & Khani (Syn. *Kali griffithii* (Bunge) Akhani & Roalson)	Ch	IT	PS-I-Tm	N				N							N									-12.15(8)	'SL/NADP-ME'(41)
Salsola jacquemontii Moq. (Syn. *Kali jaquemontii* (Moq.) Akhani & Roalson))	T	CA	XE-I-Tm	N											N										'SL/NADP-ME'(41)
Salsola paulsenii Litv. (Syn. *Kali paulsenii* (Litv.) Akhani & Roalson)	T	IT	PS-Tm	N		N		N													N			-12.32(8)	'SL/NADP-ME'(41)
Salsola pellucida Litv. (Syn. *Kali pellucidum* (Litvinov) Brullo, Giusso & Hrusa)	T	IT	PH-Tm			N																		-11.3 (9)	'SL/NADP-ME'(41)
Salsola pontica (Pall.) Iliin (Syn. Kali ponticum (Pallas) Sukhorukov)	T	M, IT	PH-L-Tm					N												N				-10.36 (*)	'SL/NADP-ME'(41)
Salsola praecox (Litv.) Litv. (Syn. *Kali praecox* (Litv.) Sukhorukov)	T	IT	PS-Tm	N		N		N							N						N			-13.7(8)	'SL/NADP-ME'(41)
Salsola tamamschjanae Iljin (Syn. *Kali tamamschjanae* (Iljin) Akhani & Roalson)	T	IT	XE-I-Tm		Ne	Ne		Ne												Ne				-10.47 (*)	'SL/NADP-ME'(41)
Salsola tragus L. (Syn. *Kali tragus* (L.) Scop.)	T	PL	R-Tm	N	N	N		N	N	N					N		N			N	N		N	-11.14(8)	'SL/NADP-ME'(41)
**37) *Bienertia***																									
Bienertia cycloptera Bunge ex Boiss.	T	IT	HL-Tm		N	N		N												N	N			-12.6 - -15.6(14)	SC/NAD-ME(46)
Bienertia kavirense Akhani	T	IT	HL-I-Tm					Ne																-11.77 - -13.03 (15)	SC/NAD-ME(46)
Bienertia sinuspersici Akhani	T	SS	HL-Tr	Ne				Ne	Ne			Ne			Ne	Ne	Ne					Ne		-13.4 - -13.9 (14)	SC/NAD-ME(46)

C_4 lineage **(bold)** and species	Life form	Choro-type	Ecotype	Distribution in Southwest Asia																				δ13C	Leaf anatomy, C_4 Photosynthetic subtype (references)
				Afghanistan	Armenia	Azerbaijan	Bahrain	Iran	Iraq	Israel/Palestine	Jordan	Kuwait	Lebanon	Oman	Pakistan	Qatar	Saudi Arabia	Sinai	Syria	Turkey	Turkmenistan	UAE	Yemen		
**38)** ***Suaeda sect. Salsina***																									
Suaeda aegyptiaca (Hasselq.) Zohary	T	SS	HL-Tr	N			N	N	N	N	N	N	N	N	N	N	N	N	N			N	N	-14.41(8)	'SD/NAD-ME'(41, 47)
Suaeda altissima (L.) Pall. ex J.F. Gmel.	T	IT	HL-I-Tm	N	N	N		N	N	N	N		N					N	N	N	N			-13.26(8)	SD/NAD-ME(41,87)
Suaeda arcuata Bunge	T	IT	HL-I-Tm	N				N							N						N			-12.75(8)	'SD/NAD-ME'(41, 47)
Suaeda asphaltica (Boiss.) Boiss.	Ch	SS	HL-Tr							Ne	Ne		Ne						Ne					-9.7(8)	'SD/NAD-ME'(41, 47)
Suaeda baluchestanica Akhani & Podlech	Ch	SS	HL-L-Tr					Ne							Ne									-12.92(8)	'SD/NAD-ME'(41, 47)
Suaeda dendroides (C.A. Mey) Moq.	Ch	IT	HL-I-Tm		N	N		N													N			-12.29(8)	'SD/NAD-ME'(41, 47)
Suaeda fruticosa Forssk. ex J.F. Gmel.	Ch	SS	HL	N				N	N	N	N				N		N						N	-13.74(8)	'SD/NAD-ME'(41, 47)
Suaeda microphylla Pall.	Ch	IT	XH-Tm	N	N	N		N											N	N	N			-11.09(8)	'SD/NAD-ME'(41, 47)
Suaeda monoica Forssk. ex J. F. Gmel.	Ch, P	SS	HL-Tr					???	N	N	N			N	N		N	N					N	-13.27(8)	'SD/NAD-ME'(41, 47)
Suaeda moschata A.J. Scott	Ch	SS	HL-Tr											Ne										-11.94 (*)	'SD/NAD-ME'(41, 47)
Suaeda vermiculata Forssk. ex J.F. Gmel.	Ch	SS	HL-Tr	N			N	N	N	N	N	N		N		N	N	N				N	N	-13.0(9)	'SD/NAD-ME'(41, 47)
**39)** ***Suaeda sect. Schoberia***																									
Suaeda acuminata C.A. Mey.	T	IT	HL-Tm	N	N			N							N					N	N			-12.8(9)	'CS/NAD-ME'(47)
Suaeda carnosissima Post	T	IT	HL-Tm						N		N		N						N	N				-12.6(9)	'CS/NAD-ME'(47)
Suaeda cochlearifolia Woł. ex Stapf	T	IT	HL-I-Tm					Ne																-13.68 (*)	'CS/NAD-ME'(47)
Suaeda confusa Iljin	T	IT	HL-Tm			N														N				-10.45(8)	CS/NAD-ME(47, 41)
Suaeda cucullata Aellen	T	IT	HL-Tm																	N				-12.62(8)	'SD/NAD-ME'(41, 47)
Suaeda eltonica Iljin	T	IT	HL-I-Tm																	N				-12.05 (*)	CS/NAD-ME(47,41)
Suaeda gracilis Moq.	T	IT	HL-I-Tm					Ne																-14.52 (*)	'SD/NAD-ME'(41, 47)
Suaeda khalijefarsica Akhani	T	SS	HL-Tr					Ne																-13.50 (*)	'SD/NAD-ME'(41, 47)
Suaeda microsperma (C.A. Mey.) Fenzl	T	IT	HL-Tm					N							N									-13.56(8)	'SD/NAD-ME'(41, 47)
Suaeda splendens (Pourr.) Gren. & Godr.	T	IT	HL-L-Tm							N	N							N		N				-13.41(8)	'SD/NAD-ME'(41, 47)
Suaeda turkestanica Litv.	T	IT	HL-Tm																		N			-11.83 (8)	'SD/NAD-ME'(41, 47)
Gisekiaceae																									
**40) C_4 *Gisekia***																									
Gisekia pharnaceoides L.	T	SS	PS, R-Tr	N				N		N	N			N	N		N					N	N	-10.6 - -14.3 (16)	AT/NAD-ME (16)
**Aizoaceae**																									
**41)** ***Sesuvium/ Trianthema/Zaleya***																									
Sesuvium sesuvioides Verdc.	T or H	PL	HL, R-Tr												N		N						N	−12.4(5)	PT(5)/NADP-ME(88)
Trianthema crystallinum (Forssk.) Vahl	T or H	SM, SS, IC	R-Tr														N						N	−14.0(5)	'AT(5)NADP-ME and PEP-CK'(40, 5)
Trianthema portulacastrum L.	T	PL	R-Tr					N		N	N				N		N						N	-12.3 (5)	AT/NADP-ME and PEP-CK (40, 5)
Trianthema sheilae A.G. Mill & J.Nyberg	T or H	SS, SM	XE-Tr														N						N		AT/NAD-ME (5)
Trianthema triquetrum Rottler & Willd.	T	PL	XE, PS-Tr											N	N		N						N	-13.25- -14.31 (6)	'AT/NADP-ME and PEP-CK' (40, 5)

C_4 lineage **(bold)** and species	Life form	Choro-type	Ecotype	Distribution in Southwest Asia																				δ^{13}C	Leaf anatomy, C_4 Photosynthetic subtype (references)
				Afghanistan	Armenia	Azerbaijan	Bahrain	Iran	Iraq	Israel/Palestine	Jordan	Kuwait	Lebanon	Oman	Pakistan	Qatar	Saudi Arabia	Sinai	Syria	Turkey	Turkmenistan	UAE	Yemen		
Zaleya pentandra (L.) C. Jeffrey	T or H	PL	XE, PS-Tr	N				N		N	N			N	N		N					N	N	−12.9 (5)	AT/NAD-ME and PEP-CK (40, 5)
**Nyctaginaceae**																									
**42) *Boerhavia***																									
Boerhavia diandra L.	H	SS, SG	XE-Tr												N										'AT/NADP-ME' (41)
Boerhavia elegans Choisy	Ch	PL	XE-Tr					N						N	N		N					N	N	-11.99 (2)	'AT/NADP-ME' (41)
Boerhavia procumbens Banks ex Roxb.	H	I	R-Tr												N									-11.97 (*)	'AT/NADP-ME' (41)
Boerhavia repens L.	T or H	PL	XE-Tr							N	N				N		N	N					N	-12.27 (*)	AT/NADP-ME (41)
**Molluginaceae**																									
**43) *Hypertelis cerviana/H. fragilis***																									
Hypertelis cerviana (L.) Thulin	T	PL	PS, R					N							N		N			N			N	−13.3 (17)	AT/NAD-ME(41)
Portulacaceae																									
**44) Portulacaceae**																									
Portulaca foliosa Ker Gawl	T or H	SS,SM	R-Tr														N						N	−15.0 (19)	PI (19)
Portulaca grandiflora W.J. Hook	T or H	PL	R-Tr	I		I			I						I									-11.7 - -12.2 (19)	PI/NADP-ME (19)
Portulaca kermesina N.E.Br.	T or H	PL	XE-Tr														N							-12.65 (*)	'PI/NADP-ME' (19)
Portulaca oleracea L. aggregate (incl. *P. zaffranii* Danin , *P. cypria* Danin , *P. edulis* Danin & Bagella , *P. nitida* (Danin & H. G. Baker) Ricceri & Arrigoni , *P. granulatostellulata* (Poelln.) Ricceri & Arrigoni, *P. papillatostellulata* (Danin & H. G. Baker) Danin, *P. rausii* Danin , *P. sativa* Haw. , *P. trituberculata* Danin & al. , *P. socotrana* Domina & Raimondo)	T	PL	R	N	N	N	N	N	N	N	N	N	N	N	N	N	N	N	N	N	N	N	N	-12.4 - -12.6 [23](19)	AT/NAD-ME (19)
Portulaca pilosa L.	T	PL	R-Tr												I		I							−13.0 (19)	PI/NADP-ME (19)
Portulaca quadrifida L.	T	PL	R-Tr				N							N	N		N						N	−12.5 (19)	PT (19)
Potulaca tuberosa Roxb.	T	SS	R-Tr												N									−11.3 (19)	PI (19)
**BORAGINALES, Boraginaceae**																									
**45) *Euploca***																									
Euploca personata (Thulin) Thulin	H	SM	XE-Tr																				N		'AT/NADP-ME' (37)
Euploca rariflora (Stocks) Diane & Hilger	H	SS, SM, SU	R-Tr					N							N		N						N	-14.61 - - 15.09 (1)	'AT/NADP-ME' (37)
Euploca sessilistigma (Hutch. & E.A.Bruce) E.L.A.N.Simons & Wieringa	H	SM	XE-Tr																				N		'AT/NADP-ME' (37)
Euploca strigosa (Willd.) Diane & Hilger (incl. *Heliotropium brevifolium* Wall. and *H.*	H	PL	R-Tr	N										N	N		N					N	N	-13.01(2)	'AT/NADP-ME' (37, 49)

C_4 lineage **(bold)** and species	Life form	Choro-type	Ecotype	Distribution in Southwest Asia																				δ^{13}C	Leaf anatomy, C_4 Photosynthetic subtype (references)
				Afghanistan	Armenia	Azerbaijan	Bahrain	Iran	Iraq	Israel/Palestine	Jordan	Kuwait	Lebanon	Oman	Pakistan	Qatar	Saudi Arabia	Sinai	Syria	Turkey	Turkmenistan	UAE	Yemen		
marifolium J.Koenig ex Retz.)																									
**LAMIALES**																									
**Scrophulariaceae**																									
46) Anticharis																									
Anticharis glandulosa Asch. *(incl. A. arabica Endl.)*	T	SS	XE, O-Tr					N		N	N				N		N	N				N	N	-13.7 - - 14.2(21)	AT/NAD-ME (21)
Anticharis senegalensis (Walp.) Bhandari	T	SS	XE, O-Tr												N								N	-12.7 - -15.0 (21)	AT/NAD-ME (21)
**Acanthaceae**																									
**47) *Blepharis***																									
Blepharis attenuata Napper	Ch	SS	XE, PS, O-Tr							N	N													-12.5 (20)	AT/NADP-ME (41)
Blepharis ciliaris (L.) B.L. Burtt	Ch	SS,SM, EA	XE, PS, O-Tr					Ne		Ne	Ne			Ne	Ne			Ne					Ne	-14.2 (20)	AT/NAD-ME (48)
Blepharis edulis (Forssk.) Pers.	Ch	SS, SM	XE, PS, O-Tr					N							N		N						N	-14.6 (20)	'AT'(48)
Blepharis kuriensis Vierh.	Ch	SM	XE, PS, O-Tr																				Ne	-13.5 (20)	'AT'(48)
Blepharis saudensis Y. Masrahi	T or H	SM	PS-L-Tr														Ne								AT (91)
Blepharis sindica Stocks ex T. Anders.	Ch	I	XE, PS, O-Tr											Ne	Ne									-13.4 (20)	'AT'(48)
**ASTERALES, Asteraceae**																									
**48) *Flaveria* clade A**																									
Flaveria bidentis (L.) Kuntze	T	PL	R-Tr						Ii									Ii						-16.5 (3)	AT/NADP-ME (38)
Flaveria trinervia (Spreng) C. Mohr	T	PL	R-Tr						I					I			I					I	I	-14.0 (3)	AT/NADP-ME (38)

[1] under *F. annua;* [2] under *A. micans;* [3] under *A. coerulescens* Desf.; [4] under *E. amabilis;* [5] under *T. ochroleucus;* [6] under *T. mollis;* [7] under *T. longipetalus;* [8] under *T. pterophorus;* [9] under *C. mongolicum;* [10] under *C. amoenum;* [11] under *C. turkestanicum;* [12] under *C. rubescens;* [13] under *C. intertextum;* [14] under *C. denticulatum;* [15] under *C. stenopterum;* [16] under *C. bungei;* [17] under *C. platyacanthum;* [18] under *C. bubyrii;* [19] under *C. muravlyansyi;* [20] under *C. cordatum;* [21] under *A. rudis;* [22] under *H. ramosissima;* [23] under *P. nitida*

References for isotope data, leaf anatomy and C4 photosynthetic subtypes. List of references are provided in Supplementary Data 1.

(*)=New data; (+)= Unpublished data Ziegler; 1. Akhani, 2007; 2. Ziegler et al., 1981; 3. Smith and Turner, 1975; 4. Feodorova et al., 2010; 5. Bohley et al., 2014; 6. Fedha, 2017; 7. Sage et al., 2007; 8. Akhani et al., 1997; 9. Winter, 1981; 10. Freitag and Kadereit, 2014; 11. Akhani et al., 2016; 12. Pyankov et al., 2001b; 13. Freitag and Stichler, 2000; 14. Akhani et al., 2005; 15. Akhani et al., 2012; 16. Bissinger et al., 2014; 17. Christin et al., 2011; 18. Soskov, 2011; 19. Ocampo et al., 2013; 20. Fisher et al., 2015; 21. Khoshravesh et al., 2012; 22. Batanouny et al., 1991; 23. Horn et al., 2014; 24. Pyankov et al., 2010; 25. Lauterbach et al., 2016; 26. Hesla et al., 1982; 27. Takeda et al., 1985; 28. Bräutigam et al., 2014; 29. Kalapos et al., 1997; 30. Ehleringer et al., 1987; 31. Schulze et al., 1996; 32. Winter et al., 1976; 33. Hattersley, 1982; 34. Pyankov et al., 2000a; 35. Brown, 1977; 36. Osborne et al., 2014; 37. Muhaidat et al., 2011; 38. Kopriva et al., 1996; 39. Koteyeva et al., 2011; 40. Muhaidat and McKown, 2013; 41. Muhaidat et al., 2007; 42. Kadereit et al., 2003; 43. Carolin et al., 1978; 44. Akhani and Khoshravesh, 2013; 45. Voznesenskaya et al., 2008; 46. Akhani et al., 2003; 47. Voznesenskaya et al., 2007; 48. Akhani et al., 2008; 49. Förther, 1998; 50. Bowes, 2011; 51. Bruhl et al., 1987; 52. Soros and Bruhl, 2000; 53. Ibrahim et al., 2009; 54. Grass Phylogeny Working Group, 2001; 55. Sage, 2011; 56. Christin et al., 2009; 57. Edwards and Voznesenskaya, 2011; 58. Bell et al., 2000; 59. Gutierrez et al. 1974; 60. Voznesenskaya et al., 2005; 61)(Väre and Kukkonen, 2004); .62. Muasya et al., 2002; 63. Amini Rad and Sonboli, 2008; 64. Hofstra et al., 1972; 65. Ayeni et al., 2015; 66. Sahni and Naithani, 1976; 67. Druyts - Voets, 1970; 68. Li et al., 1993; 69. Govindarajalu, 2008; 70. Hooper, 1985; 71. Larridon et al., 2014; 72. Danin and Kukkonen, 1995; 73. Sabnis, 1919; 74. Ueno and Takeda, 1992; 75. Hattersley and Watson, 2009; 76. Ueno et al., 1986; 77. Gómez-Martínez and Culham, 2000; 78. Toderich et al., 2007; 79. Shi et al., 2016; 80. Pyankov et al., 1986; 81. POWO, 2019; 82. Akhani et al., 2009; 83. Pyankov VI and Molotkovskii YI., 1992; 84.

Pyankov et al., 2001a; 85. Pyankov et al., 2000b; 86. Pyankov et al., 1999; 87. Gamaley et al., 1972; 88. Bohley et al., 2019; 89. Väre and Kukkonen, 2005; 90. Mesterhazy and Verloove, 2018; 91. Basahi and Masrahi, 2019; (#)=Doostmohammadi et al. 2020.

Refeences

Akhani H. 2007. Diversity, biogeography, and photosynthetic pathways of Argusia and Heliotropium (Boraginaceae) in South-West Asia with an analysis of phytogeographical units. Botanical Journal of the Linnean Society **155**, 401–425.

Akhani H, Barroca J, Koteyeva N, Voznesenskaya E, Franceschi V, Edwards G, Ghaffari S, Ziegler H. 2005. Bienertia sinuspersici (Chenopodiaceae): A New Species from Southwest Asia and Discovery of a Third Terrestrial C4 Plant Without Kranz Anatomy. Systematic Botany **30**, 290–301.

Akhani H, Chatrenoor T, Dehghani M, Khoshravesh R, Mahdavi P, Matinzadeh Z. 2012. A new species of Bienertia (Chenopodiaceae) from Iranian salt deserts: A third species of the genus and discovery of a fourth terrestrial C4 plant without Kranz anatomy. Plant Biosystems **146**, 1–10.

Akhani M, Ghasemkhani M, Chuong S, Edwards G. 2008. Occurrence and forms of Kranz anatomy in photosynthetic organs and characterization of NAD-ME subtype C 4 photosynthesis in Blepharis ciliaris (L.) B. L. Burtt (Acanthaceae). Journal of Experimental Botany **59**, 1755–1765.

Akhani H, Ghobadnejhad M, Hashemi S. 2003. Ecology, Biogeography and Pollen Morphology of Bienertia cycloptera Bunge ex Boiss. (Chenopodiaceae), an Enigmatic C4 Plant without Kranz Anatomy. Plant Biology **5**, 167–178.

Akhani H, Khoshravesh R. 2013. The relationship and different C4 Kranz anatomy of Bassia eriantha and Bassia eriophora , two often confused Irano-Turanian and Saharo-Sindian species. Phytotaxa **93**, 1–24.

Akhani H, Khoshravesh R, Malekmohammadi M. 2016. Taxonomic novelties from Irano-Turanian region and NE Iran: Oreosalsola, a new segregate from Salsola s.l., two new species in Anabasis and Salvia , and two new combinations in Caroxylon and Seseli. Phytotaxa **249**, 159–180.

Akhani H, Lara M, Ghasemkhani M, Ziegler H, Edwards G. 2009. Does Bienertia cycloptera with the single-cell system of C(4) photosynthesis exhibit a seasonal pattern of delta (13)C values in nature similar to co-existing C (4) Chenopodiaceae having the dual-cell (Kranz) system? Photosynthesis Research **99**, 23–36.

Akhani H, Trimborn P, Ziegler H. 1997. Photosynthetic pathways in Chenopodiaceae from Africa, Asia and Europe with their ecological, phytogeographical and taxonomical importance. Plant Systematics and Evolution **206**, 187–221.

Amini Rad M, Sonboli A. 2008. Leaf and stem anatomy of the cyperus subgenus cyperus in iran. Rostaniha **9**, 6–21.

Ayeni O, Jimoh M, Saheed S. 2015. Inflorescence and floral characters indicating C_3 and C_4 photosynthesis in some species of the genus Cyperus L. (Cyperaceae). International Journal of Biological and Chemical Sciences **9**, 1844–1850.

Basahi M, Masrahi Y. 2019. *Blepharis saudensis* (Acanthaceae), a new species from Saudi Arabia. Saudi Journal of Biological Sciences.

Batanouny K, Stichler W, Ziegler H. 1991. Photosynthetic pathways and ecological distribution ofEuphorbia species in Egypt. Oecologia **87**, 565–569.

Bell T, Stock W, Linder H. 2000. Ecophysiological investigations of the distribution of Poaceae and Restionaceae in the Cape Floristic region, South Africa. Grasses - Systematics and Evolution.267–278.

Bissinger K, Khoshravesh R, Kotrade J, Oakley J, Sage T, Sage R, Hartmann H, Kadereit G. 2014. Gisekia (Gisekiaceae): Phylogenetic relationships, biogeography, and ecophysiology of a poorly known C4 lineage in the Caryophyllales. American Journal of Botany **101**, 499–509.

Bohley K, Joos O, Hartmann H, Sage R, Liede-Schumann S, Kadereit G. 2014. Phylogeny of Sesuvioideae (Aizoaceae) – Biogeography, leaf anatomy and the evolution of C4 photosynthesis. Perspectives in Plant Ecology, Evolution and Systematics **17**, 116–130.

Bohley K, Schröder T, Kesselmeier J, Ludwig M, Kadereit G. 2019. C4-like photosynthesis and the effects of leaf senescence on C4-like physiology in Sesuvium sesuvioides (Aizoaceae). Journal of Experimental Botany **70**, 1553–1565.

Bowes G. 2011. Chapter 5 Single-Cell C_4 Photosynthesis in Aquatic Plants. In: Raghavendra AS,, In: Sage RF, eds. Advances in Photosynthesis and Respiration. C4 Photosynthesis and Related CO2 Concentrating Mechanisms. Dordrecht: Springer Netherlands, 63–80.

Bräutigam A, Schliesky S, Külahoglu C, Osborne C, Weber A. 2014. Towards an integrative model of C_4 photosynthetic subtypes: insights from comparative transcriptome analysis of NAD-ME, NADP-ME, and PEP-CK C4 species. Journal of Experimental Botany **65**, 3579–3593.

Brown W. 1977. The Kranz syndrome and its subtypes in grass systematics. Memoirs of the Torrey Botanical Club **23**, 1–97.

Bruhl J, Stone N, Hattersley P. 1987. C_4 Acid Decarboxylation Enzymes and Anatomy in Sedges (Cyperaceae): First Record of NAD-Malic Enzyme Species | Request PDF. Functional Plant Biology **14**, 719–728.

Carolin R, Jacobs S, Vesk M. 1978. Kranz cells and mesophyll in the Chenopodiales | Request PDF. Australian Journal of Botany **26**, 683–698.

Christin P, Sage T, Edwards E, Ogburn R, Khoshravesh R, Sage R. 2011. Complex evolutionary transitions and the significance of C_3-C_4 intermediate forms of photosynthesis in Molluginaceae. Evolution; International Journal of Organic Evolution **65**, 643–660.

Christin P, Salamin N, Kellogg E, Vicentini A, Besnard G. 2009. Integrating Phylogeny into Studies of C_4 Variation in the Grasses. Plant Physiology **149**, 82–87.

Danin A, Kukkonen I. 1995. Contributions to the flora of Israel. VIII. A new Cyperus from Israel, Cyperus sharonensis Danin et Kukkonen sp. n. Israel Journal of Plant Sciences **43**, 77–82.

Doostmohammadi M, Malekmohammadi M, Djamali M, Akhani H. 2020. Is Pteropyrum a pathway to C4 evolution in Polygonaceae? An integrative approach to the taxonomy and anatomy of Pteropyrum (C_3), an immediate relative of Calligonum (C_4). Botanical Journal of the Linnean Society **192**, 369–400.

Druyts - Voets E. 1970. Types van stengel: en bladstrukturen in het genus Cyperus L. **52**, 28–49.

Edwards G, Voznesenskaya E. 2011. Chapter 4 C4 Photosynthesis: Kranz Forms and Single-Cell C4 in Terrestrial Plants. In: Raghavendra A,, In: Sage R, eds. Advances in Photosynthesis and Respiration. C4 Photosynthesis and Related CO2 Concentrating Mechanisms. Dordrecht: Springer Netherlands, 29–61.

Ehleringer J, Lin Z, Field C, Sun G, Kuo C. 1987. Leaf carbon isotope ratios of plants from a subtropical monsoon forest | SpringerLink. Oecologia **72**, 109–114.

Fedha S. 2017. Ecological Implications Of The δ13c Values of Plant Species Growing in Natural Environment, Greenhouse and Plant Respired Carbon Dioxide (Captured as Carbonate). Journal of Pharmacy and Biological Sciences **12**, 51–58.

Feodorova T, Voznesenskaya E, Edwards G, Roalson E. 2010. Biogeographic Patterns of Diversification and the Origins of C4 in Cleome (Cleomaceae). Systematic Botany **35**, 811–826.

Fisher A, McDade L, Kiel C, Khoshravesh R, Johnson M, Stata M, Sage T, Sage R. 2015. Evolutionary History of Blepharis (Acanthaceae) and the Origin of C4 Photosynthesis in Section Acanthodium. International Journal of Plant Sciences **176**, 770–790.

Förther H. 1998. Die infragenerische Gliederung der Gattung Heliotropium L. und ihre Stellung innerhalb der subfam. Heliotropioideae (Schrad.) Arn. (Boraginaceae). Sendtnera : Mitteilungen der Botanischen Staatssammlung und des Instituts für Systematische Botanik der Universitat München. **5**, 35–241.

Freitag H, Kadereit G. 2014. C3 and C4 leaf anatomy types in Camphorosmeae (Camphorosmoideae, Chenopodiaceae). Plant Systematics and Evolution **300**, 665–687.

Freitag H, Stichler W. 2000. A Remarkable New Leaf Type With Unusual Photosynthetic Tissue in a Central Asiatic Genus of Chenopodiaceae. Plant Biology **2**, 154–160.

Gamaley Y, Glagoleva T, Kolanovskaja M. 1972. Ecology and evolution of C 4 -syndrome types in relation with phylogeny of families Chenopodiaceae and Poaceae. Botanicheskii Zhurnal **77**, 2–12.

Gómez-Martínez R, Culham A. 2000. Phylogeny of the subfamily Panicoideae with emphasis on the tribe Paniceae: evidence from the trnL-F cpDNA region. Grasses: systematics and evolution. CSIRO Publishing, 136–140.

Govindarajalu E. 2008. The systematic anatomy of south Indian Cyperaceae: Cyperus L. subgen. Kyllinga (Rottb.) Suringar. Botanical Journal of the Linnean Society **62**, 41–58.

Grass Phylogeny Working Group (last), Barker N, Clark L, *et al.* 2001. Phylogeny and Subfamilial Classification of the Grasses (Poaceae). Annals of the Missouri Botanical Garden **88**, 373–457.

Gutierrez M, Gracen V, Edwards G. 1974. Biochemical and cytological relationships in C4 plants. Planta **119**, 279–300.

Hattersley P. 1982. δ13 Values of C4 Types in Grasses. Functional Plant Biology **9**, 139–154.

Hattersley P, Watson L. 2009. Diversification of photosynthesis. Grass evolution and domestication. Cambridge University Press, 38–116.

Hesla B, Tieszen L, Imbamba S. 1982. A Systematic Survey of C3 and C4 Photosynthesis in the Cyperaceae of Kenya, East Africa. Photosynthetica **16**, 196–205.

Hofstra J, Aksornkoae S, Atmowidjojo S, Banaag J, Santosa, Sastrohoetomo R, Thu L. 1972. A study on the occurrence of plants with a low CO2 compensation point in different habitats in the tropics. Annales Bogorienses **5**, 143–157.

Hooper S. 1985. An Interesting Species of Pycreus (Cyperaceae) of Saharo-Sindian Distribution. Kew Bulletin **40**, 467–469.

Horn J, Xi Z, Riina R, Peirson J, Yang Y, Dorsey B, Berry P, Davis C, Wurdack K. 2014. Evolutionary bursts in Euphorbia (Euphorbiaceae) are linked with photosynthetic pathway. Evolution **68**, 3485–3504.

Ibrahim D, Burke T, Ripley B, Osborne C. 2009. A molecular phylogeny of the genus Alloteropsis (Panicoideae, Poaceae) suggests an evolutionary reversion from C4 to C3 photosynthesis. Annals of Botany **103**, 127–136.

Kadereit G, Borsch T, Weising K, Freitag H. 2003. Phylogeny of Amaranthaceae and Chenopodiaceae and the Evolution of C4 Photosynthesis | International Journal of Plant Sciences: Vol 164, No 6. International Journal of Plant Sciences **164**, 959–986.

Kalapos T, Baloghné-Nyakas A, Csontos P. 1997. Occurrence and ecological characteristics of C4 dicot and Cyperaceae species in the Hungarian flora. Photosynthetica **33**, 227–240.

Khoshravesh R, Akhani H, Sage T, Nordenstam B, Sage R. 2012. Phylogeny and photosynthetic pathway distribution in Anticharis Endl. (Scrophulariaceae). Journal of Experimental Botany **63**, 5645–5658.

Kopriva S, Chu C, Bauwe H. 1996. Molecular phylogeny of Flaveria as deduced from the analysis of nucleotide sequences encoding the H-protein of the glycine cleavage system. Plant, Cell & Environment **19**, 1028–1036.

Koteyeva N, Voznesenskaya E, Roalson E, Edwards G. 2011. Diversity in forms of C4 in the genus Cleome (Cleomaceae). Annals of Botany **107**, 269–283.

Larridon I, Bauters K, Reynders M, Huygh W. 2014. Taxonomic changes in C4 Cyperus (Cypereae, Cyperoideae, Cyperaceae): Combining the sedge genera Ascolepis, Kyllinga and Pycreus into Cyperus s.l. Phytotaxa **166**, 33–48.

Lauterbach M, Van der Merwe P, Keßler L, Pirie M, Bellstedt D, Kadereit G. 2016. Evolution of leaf anatomy in arid environments **-** A case study in southern African Tetraena and Roepera (Zygophyllaceae). Molecular phylogenetics and evolution **97**, 129–144.

Li M, Wedin D, Tieszen L. 1993. Distribution of C 3 and C 4 species of Cyperus in Europe. Photosynthetica **28**, 119–126.

Mesterhazy A, Verloove F. 2018. On the identity of the Turkish endemic Cyperus noeanus (Cyperaceae). Turkish Journal of Botany **42**, 233–238.

Muasya A, Simpson D, Chase M. 2002. Phylogenetic relationships in Cyperus L. s.l. (Cyperaceae) inferred from plastid DNA sequence data. Botanical Journal of the Linnean Society **138**, 145–153.

Muhaidat R, McKown A. 2013. Significant involvement of PEP-CK in carbon assimilation of C4 eudicots. Annals of Botany **111**, 577–589.

Muhaidat R, Sage R, Dengler N. 2007. Diversity of Kranz anatomy and biochemistry in C4 eudicots. American Journal of Botany **94**, 362–381.

Muhaidat R, Sage T, Frohlich MW, Dengler NG, Sage R. 2011. Characterization of C3 –C4 intermediate species in the genus Heliotropium L. (Boraginaceae): anatomy, ultrastructure and enzyme activity. Plant, Cell and Environment **34**, 1723–1736.

Ocampo G, Koteyeva N, Voznesenskaya E, Edwards G, Sage T, Sage R, Columbus J. 2013. Evolution of leaf anatomy and photosynthetic pathways in Portulacaceae. American Journal of Botany **100**, 2388–2402.

Osborne C, Salomaa A, Kluyver T, Visser V, Kellogg E, Morrone O, Vorontsova M, Clayton W, Simpson D. 2014. A global database of C4 photosynthesis in grasses. New Phytologist **204**, 441–446.

POWO (2019). "Plants of the World Online. Facilitated by the Royal Botanic Gardens, Kew. Published on the Internet; http://www.plantsoftheworldonline.org/ [Accessed 20.07.2019]

Pyankov V, Artyusheva E, Edwards G. 1999. Formation of C-4 syndrome in leaves and cotyledons of Kochia scoparia and Salsola collina (Chenopodiaceae). **46**, 452–466.

Pyankov V, Artyusheva E, Edwards G, Soltis P. 2001*a*. Phylogenetic analysis of tribe Salsoleae (Chenopodiaceae) based on ribosomal ITS sequences: implications for the evolution of photosynthesis types. American Journal of Botany **88**, 1189–1198.

Pyankov V, Gunin P, Tsoog S, Black C. 2000*a*. C4 plants in the vegetation of Mongolia: their natural occurrence and geographical distribution in relation to climate. Oecologia **123**, 15–31.

Pyankov V, Vakhrusheva D, Burundukova O. 1986. The photosynthetic pathway types in the hot desert of Central Karakum and its ecological importance. Problems of Desert Development **2**.

Pyankov VI, Molotkovskii YI. 1992. Species composition and ecological role of C 4 grasses in the arid zone of Central Asia. Soviet Journal of Ecology **23**, 144–151.

Pyankov V, Voznesenskaya E, Kuz'min A, Ku M, Ganko E, Franceschi V, Black C, Edwards G. 2000*b*. Occurrence of C(3) and C(4) photosynthesis in cotyledons and leaves of Salsola species (Chenopodiaceae). Photosynthesis Research **63**, 69–84.

Pyankov V, Ziegler H, Akhani H, Deigele C, Luettge U. 2010. European plants with C4 photosynthesis: geographical and taxonomic distribution and relations to climate parameters. Botanical Journal of the Linnean Society **163**, 283–304.

Pyankov V, Ziegler H, Kuz'min A, Edwards G. 2001*b*. Origin and evolution of C4 photosynthesis in the tribe Salsoleae (Chenopodiaceae) based on anatomical and biochemical types in leaves and cotyledons. Plant Systematics and Evolution **230**, 43–74.

Sabnis T. 1919. The physiological anatomy of the plants in the Indian desert. Journal of Indian Botany **1**, 93–115.

Sage R. 2011. The C(4) plant lineages of planet Earth. Journal of Experimental Botany **62**, 3155–3169.

Sage R, Sage T, Pearcy R, Borsch T. 2007. The taxonomic distribution of C4 photosynthesis in Amaranthaceae sensu stricto. American Journal of Botany **94**, 1992–2003.

Sahni K, Naithani H. 1976. A New Species of Cyperus from India. Indian Forester **102**, 357–359.

Schulze E, Ellis R, Schulze W, Trimborn P, Ziegler H. 1996. Diversity, metabolic types and δ13C carbon isotope ratios in the grass flora of Namibia in relation to growth form, precipitation and habitat conditions. Oecologia **106**, 352–369.

Shi W, Wen J, Pan B. 2016. A comparison of ITS sequence data and morphology for Calligonum pumilum and C. mongolicum (Polygonaceae) and its taxonomic implications. Phytotaxa **261**, 157–167.

Smith B, Turner B. 1975. Distribution of Kranz Syndrome Among Asteraceae. American Journal of Botany **62**, 541–545.

Soros C, Bruhl J. 2000. Multiple evolutionary origins of C 4 photosynthesis in the Cyperaceae. Monocots – systematics and evolution. CSIRO Publishing, 629–636.

Soskov Y. 2011. *Rod Calligonum L. — zhuzgun (sistematika, geografiya, evolyutsiya, introduktsiya) [Genus Calligonum L. — zhuzgun (taxonomy, geography, evolution, introduction)*. Novosibirsk: Siberian Sci. Agric. Library.

Takeda T, Ueno O, Samejima M, Ohtani T. 1985. An investigation for the occurrence of C4 photosynthesis in the Cyperaceae from Australia. The botanical magazine = Shokubutsu-gaku-zasshi **98**, 393–411.

Toderich K, Black C, Juylova E, Kozan O, Mukimov T, Matuso N. 2007. C3/C4 plants in the vegetation of Central Asia, geographical distribution and environmental adaptation in relation to climate. Climate Change and Terrestrial Carbon Sequestration in Central Asia. CRC Press, 33–63.

Ueno O, Takeda T. 1992. Photosynthesis pathways, ecological characteristics, and the geographical distribution of the Cyperaceae in Japan. Oecologia **89**, 195–203.

Ueno O, Takeda T, Murata T. 1986. C4 acid decarboxylating enzyme activities of C4 species possessing different anatomical types in the Cyperaceae. Photosynthetica **20**, 111–116.

Väre H, Kukkonen I. 2004. Typification of names in Cyperus section Arenarii (Cyperaceae). Nordic Journal of Botany **24**, 279–294.

Väre H, Kukkonen I. 2005. Seven new species of Cyperus (Cyperaceae) section Arenarii and one new combination and typification. Annales Botanici Fennici **42**, 473–483.

Voznesenskaya E, Akhani H, Koteyeva N, Chuong S, Roalson E, Kiirats O, Franceschi V, Edwards G. 2008. Structural, biochemical, and physiological characterization of photosynthesis in two C4 subspecies of Tecticornia indica and the C3 species Tecticornia pergranulata (Chenopodiaceae). Journal of Experimental Botany **59**, 1715–1734.

Voznesenskaya E, Chuong S, Kiirats O, Franceschi V, Edwards G. 2005. Evidence that C4 species in genus Stipagrostis, family Poaceae, are NADP-malic enzyme subtype with nonclassical type of Kranz anatomy (Stipagrostoid) **-** ScienceDirect. Plant Science **168**, 731–739.

Voznesenskaya E, Chuong S, Koteyeva N, Franceschi V, Freitag H, Edwards G. 2007. Structural, Biochemical, and Physiological Characterization of C4 Photosynthesis in Species Having Two Vastly Different Types of Kranz Anatomy in Genus Suaeda (Chenopodiaceae). Plant Biology **9**, 745–757.

Winter K. 1981. C4 plants of high biomass in arid regions of asia-occurrence of C4 photosynthesis in Chenopodiaceae and Polygonaceae from the Middle East and USSR. Oecologia **48**, 100–106.

Winter K, Troughton J, Card K. 1976. δ13C values of grass species collected in the northern Sahara desert. Oecologia **25**, 115–123.

Ziegler H, Batanouny K, Sankhla N, Vyas O, Stichler W. 1981. The photosynthetic pathway types of some desert plants from India, Saudi Arabia, Egypt, and Iraq. Oecologia **48**, 93–99.
