# Supplementary figures and images for: A Review of C4 Plants in Southwest Asia: An Ecological, Geographical and Taxonomical Analysis of a Region With High Diversity of C4 Eudicots
Source: Front Plant Sci. 2020 Nov 5;11:546518. doi: 10.3389/fpls.2020.546518 (PMC7694577; doi:10.3389/fpls.2020.546518)

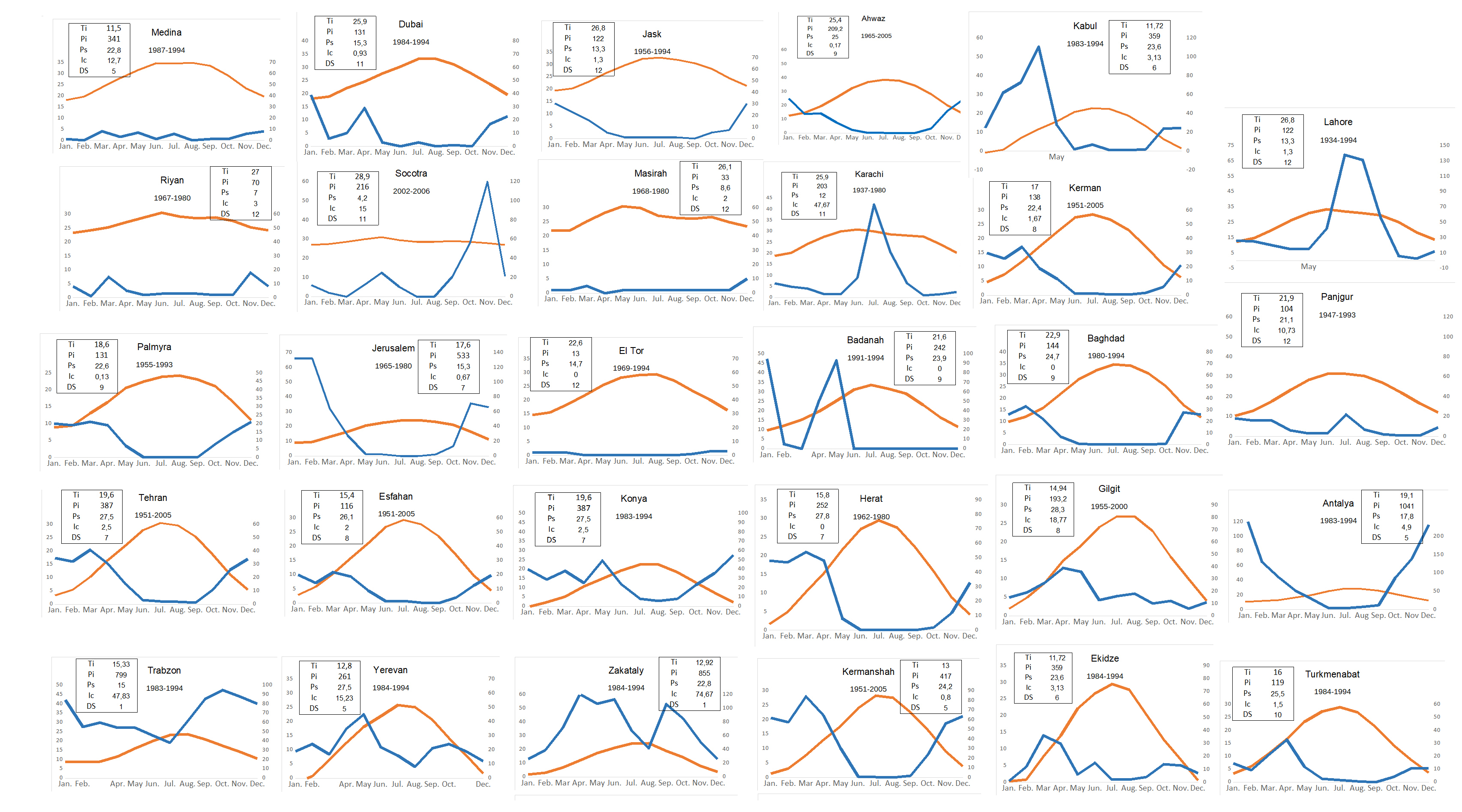

Supplement: Supplementary Figure 1 — Climatic diagrams of selected 29 stations in different parts of SW Asian countries obtained from of the Iranian Meterorological Organization (IRIMO), Scholte and De Geest (2010) and Raza et al. (2015). [file Image_1.jpeg]
