# Supplementary material for: A Review of C4 Plants in Southwest Asia: An Ecological, Geographical and Taxonomical Analysis of a Region With High Diversity of C4 Eudicots
Source: Front Plant Sci. 2020 Nov 5;11:546518. doi: 10.3389/fpls.2020.546518 (PMC7694577; doi:10.3389/fpls.2020.546518)
Supplement: Supplementary Table 2 — List of Polycarpaea (Caryophyllaceeae) species known from SW Asia which we could not test their photosynthetic type because of absence of material. [file Table_2.pdf]

Rudov A, Mashkour M, Djamali M and Akhani H (2020) A Review of C4 Plants in Southwest Asia: An Ecological, Geographical and Taxonomical Analysis of a Region With High Diversity of C4 Eudicots. Front. Plant Sci. 11:546518. doi: 10.3389/fpls.2020.546518

Supplementary table 2: List of *Polycarpaea* (Caryophyllaceeae) species known from SW Asia which we could not test their photosynthetic type because of absence of material.

**Species**	**Life form**	**Chorotype**	**Ecotype**	**Distribution**
Caryophyllaceae				
Polycarpaea kuriensis Wagner	H	SM	XE, O-I-Tr	Endemic to Soqotra
Polycarpaea balfourii Briq.	T	SM	O, XE-Tr	Endemic to Soqotra
Polycarpaea hayoides D.F.Chamb.	T	SM	XE, O-Tr	Endemic to Soqotra.
Polycarpaea jazirensis P.A.Clement	H	SM	PS-Tr	Oman, Yemen
Polycarpaea pulvinata M.G. Gilbert	Ch	SM	O-I-Tr	Yemen
Polycarpaea paulayana Wagner	T	SM	PS-L-Tr	Endemic to Soqotra.
Polycarpaea robbairea (Kuntze) Greuter & Burdet	T or H	SM, SS	PS, O, R-Tr	Saudi Arabia, Yemen, Oman, UAE, Qatar, Kuwait, Jordan, Palestine/Israel
