# Supplementary material for: A Review of C4 Plants in Southwest Asia: An Ecological, Geographical and Taxonomical Analysis of a Region With High Diversity of C4 Eudicots
Source: Front Plant Sci. 2020 Nov 5;11:546518. doi: 10.3389/fpls.2020.546518 (PMC7694577; doi:10.3389/fpls.2020.546518)
Supplement: Supplementary Table 3 — The List of Polycarpaea (Caryophyllaeceae), Cyperus and Fimbristylis (Cyperceae) species with C3 type carbon isotope values. [file Table_3.pdf]

Supplementary table 3: List of *Polycarpaea* (Caryophyllaeceae), *Cyperus* and *Fimbristylis* (Cyperceae) species with C_3 type carbon isotope values.

Species	Distribution in SW Asia	Isotope value (‰)	Source
Polycarpaea caespitosa Balf.	Endemic of Socotra	-25.32	Komarov Institute Herbarium -Socotra, Bayley Balfour , Aug. 1880, No.683) - endemic from Socotra Island (Yemen)
Polycarpaea repens (Forssk.) Aschers. & Schweinf.	Arabian Peninsula, Pakistan	-23.83	Komarov Institute Herbarium - Egypt, Sinai, dunes between Al-Kantara and Al-Arish, Bochantsev, 09.05.1962
Polycarpaea spicata Wight ex Arn.	S Iran, Pakistan, Arabian Peninsula	-23.42	Komarov Institute Herbarium - Socotra, Bayley Balfour, Feb.-Mar. 1880, No.671
Cyperus pulcherrimus Willd. ex Kunth	Doubtful rescord from Pakistan	-27.43	Komarov Institute Herbarium - Indonesia, Java, H. Zollinger, No. 638
Fimbristylis turkestanica (Regel) B. Fedtsch.	Whole SW Asia	-21.7	Herbarium Edinburgh - Iran, Gilan, Lagrosd.Lham Khaleh, 20.07.2000, Naqinezhad, 3 Eoo283472
