# Supplementary material for: A Review of C4 Plants in Southwest Asia: An Ecological, Geographical and Taxonomical Analysis of a Region With High Diversity of C4 Eudicots
Source: Front Plant Sci. 2020 Nov 5;11:546518. doi: 10.3389/fpls.2020.546518 (PMC7694577; doi:10.3389/fpls.2020.546518)
Supplement: Supplementary Table 4 — List of C4 plants with economic importance in SW Asia including non-native cultivated or introduced species. [file Table_4.pdf]

Supplementary Table 4. List of C_4 plants with economic importance in SW Asia including non-native cultivated or introduced species

Species	Utilization	Area of cultivation	Reference
Monocots Cyperaceae			
Cyperus alopecuroides Rottb.	Mat-making	Egypt	(Hanelt, 2001)
Cyperus alternifolius R.Br.	Ornamental, Mat-making	Worldwide	('Missouri Botanical Garden - Plant Finder - *Cyperus alternifolius* "Variegatus"'; Hanelt, 2001)
Cyperus articulatus L.	Essential oil, tubers, construction, traditional art, medicine, mat making	Africa	(Simpson and Inglis, 2001; Atala, 2012)
Cyperus corymbosus L.	Mat making, fibre	SE and S Asia	(Ravichandran et al., 2005; Toderich et al., 2007)
Cyperus esculentus L. and other *stoloniferous Cyperus sp.* (see note 1)	Edible tubers, comestible oil, bio-diesel	Worldwide	(Pascual et al., 2000; Hanelt, 2001; Simpson and Inglis, 2001; Arafat et al., 2009)
Cyperus exaltatus Retz.	Mat-making, Fibre, Tubers	Korea, Japan	(Hanelt, 2001; Simpson and Inglis, 2001)
Cyperus malaccensis Lam.	Fibre, construction, mat weaving	Tropical and subtropical Asia	(Ravichandran et al., 2005; Shioya et al., 2019)
Cyperus pangorei Rottb.	Fibre, construction, mat weaving	Tropical and subtropical Asia	(Ravichandran et al., 2005; Jana and Puste, 2014)
Cyperus papyrus L.	Papyrus/paper, bioenergy, construction, ornamental, mat-making, young stems and rhizomes as vegetable	Worldwide	(Duke, 1983; Hanelt, 2001; Simpson and Inglis, 2001)
Fimbristylis umbellaris (Lam.) Vahl	Weaving	SE Asia	(Hanelt, 2001)
Poaceae			
Anthephora pubescens (Steud.) Nees	Forage/Fodder	Australia	(Hanelt, 2001)
Arthraxon prionodes (Steud.) Dandy	Forage/Fodder	India	(Hanelt, 2001)
Bothriochloa bladhii (Retz.) S.T. Blake	Forage/Fodder, Erosion control	Throughout Subtropics, India, USA	(Hanelt, 2001)
Bothriochloa ischaemum (L.) Keng	Forage/Fodder, Erosion control	Tropical Africa, USA	(Hanelt, 2001)
Bothriochloa insculpta (Hochst.) A. Camus	Erosion control	Africa	(Hanelt, 2001)
Bothriochloa pertusa (L.) A. Camus	Forage/Fodder, Erosion control	Throughout tropics, India, Australian	(Hanelt, 2001)
Bothriochloa saccharoides (Sw.) Rydb.	Forage/Fodder	USA	(Hanelt, 2001)
Bouteloua curtipendula (Michx) Torr.	Ornamental, Forage/Fodder	Saudi Arabia, USA, Argentina, India	(Miller and Cope, 1996; 'The Plant Database-*USDA-NRCS*', 2019)
Bouteloua gracilis (Kunth) Lag. ex Griffiths	Ornamental	Saudi Arabia	(Miller and Cope, 1996; Hanelt, 2001; 'The Plant Database-*USDA-NRCS*', 2019)
Cenchrus americanus (L.) Morrone	Cereal	Warm and tropical regions	(Sanjana Reddy, 2017*a*)
Cenchrus sp.	Forage/Fodder, famine food	See footnote 2	(Hanelt, 2001; Belgacem and Louhaichi, 2014; Kumawat et al., 2015)
Chloris gayana Kunth	Forage/Fodder, erosion control	Tropics and Subtropics	(Hanelt, 2001)
Chloris virgata Sw.	Forage/Fodder, erosion control	India, Africa	(Hanelt, 2001)
Chrysopogon aucheri (Boiss.) Stapf	Forage/Fodder, Remediation of landscapes	India, USA	(Hanelt, 2001)
Chrysopogon fulvus (Spr.) Chiov.	Forage/Fodder, Remediation of landscapes	India, USA	(Hanelt, 2001)
Chrysopogon gryllus (L.) Trin.	Essential oil	Italy	(Hanelt, 2001)
Chrysopogon zizanioides (L.) Roberty 3	Essential oil, food additive, medicine, fibre, construction material	Tropics	(Pareek and Kumar, 2013)
Coix lacryma-jobi L. 4	Cereal, ornamental beads	Wet areas of Asia	(Jain and Banerjee, 1974; Seetharam and Riley, 1986)
Cymbopogon sp. 5	Essential oil (C. nardus (L.) Rendle, C. iwarancusa (Jones) Schult. ex Roem. & Schult.and C. winterianus	Tropics and subtropics	(Duke, 1993; Bertea and Maffei, 2009)

Species	Utilization	Area of cultivation	Reference
	Jowitt., C. martinii (Roxb.) Wats.), food (C. citratus), medicine		
Cynodon dactylon (L.) Pers.	Forage/Fodder	Worldwide	(Hanelt, 2001)
Cynodon nlemfuensis Vanderyst	Forage/Fodder	Africa, Sri Lanka	(Hanelt, 2001)
Cynodon transvaalensis Burtt Davy	Loangrass	South Africa	(Hanelt, 2001)
Dactyloctenium aegyptium (L.) Willd.	Forage/Fodder, rosion control, famine food	South Africa	(Hanelt, 2001)
Desmostachya bipinnata (L.) Stapf	Mat making, handicrafts, medicine, ritual	Extensively collected in Asia	(Wendrich, 1989; Khyade et al., 2018)
Dichanthium annulatum (Forssk.) Stapf ex Prain	Forage/Fodder	Africa, Australia, USA, India	(Hanelt, 2001)
Dichanthium aristatum (Poir.) C.E. Hubb.	Forage/Fodder, Erosion control	Tropics	(Hanelt, 2001)
Digitaria sp. 6	Cereal, Forage/Fodder, Lawn	See footnote 6	(Seetharam and Riley, 1986; Hanelt, 2001; Nesbitt, 2005)
Diplachne fusca (L.) P.Beauv. ex Roem. & Schult.	Biomass production	Pakistan	(Hanelt, 2001)
Echinochloa colonum (L.) Link	Forage/Fodder, famine food	North America	(Hanelt, 2001)
Echinochloa crusgalli (L.) P. Beauv.	Forage/Fodder, erosion control, famine food	India, Egypt, Mediterranian	(Hanelt, 2001)
Echinochloa esculenta (A.Braun) H.Scholz 7	Cereal	SE Asia, Afghanistan	(Nesbitt, 2005; Breckle et al., 2013; Gomashe, 2017*a*)
Echinochloa frumentacea Link	Cereal	S Asia, Afghanistan, Pakistan and Saudi Arabia	(Nesbitt, 2005; Gomashe, 2017*a*)
Echinochloa pyramidalis (Lam.) Hitchc. & Chase	Forage/Fodder, erosion control	Tropical Africa	(Hanelt, 2001)
Echinochloa stagnina (Retz.) P. Beauv.	Forage/Fodder, food	Niger, Egypt, Chad	(Hanelt, 2001)
Eleusine coracana (L.) Gaertn. 8	Cereal	Africa, S Asia and Pakistan	(Ganapathy, 2017*a*)
Eleusine indica (L.) Gaertn.	Frage, Famine food, fibre	Africa, India, Australia, North America	(Hanelt, 2001)
Enteropogon macrostachyus (Hochst. ex A. Rich.) Munro ex Benth.	Land remediation	Kenya	(Hanelt, 2001)
Eragrostis cilianensis (All.) Vignolo Lutati ex Janchen	Land remediation	Kenya	(Hanelt, 2001)
Eragrostis curvula (Schrad.) Nees	Forage/Fodder, erosion control	Tropics	(Hanelt, 2001)
Eragrostis lehmanniana Nees	Forage/Fodder, land remediation, erosion control	India, USA, South Africa	(Hanelt, 2001)
Eragrostis superba Peyr.	Forage/Fodder, land remediation	Africa, USA	(Hanelt, 2001)
*Eragrostis tenella (*L.) P. Beauv. ex Roem. & Schult.	Forage/Fodder, famine food	India	(Hanelt, 2001)
Eragrostis tef (Zuccagni) Trotter 9	Cereal	Ethiopia and Eritrea, recently gaining popularity worldwide	(Assefa et al., 2017)
Eragrostis unioloides (Retz.) Nees ex Steud.	Forage/Fodder	S and SE Asia	(Hanelt, 2001)
Eulaliopsis binata (Retz.) C.E. Hubbard	Paper, fibre, ropes, etc.	East India	(Sahu et al., 2010)
Hemarthria altissima (Poir.) Stapf & Hubb.	Forage/Fodder	Africa, Australia, America	(Hanelt, 2001)
Hemarthria compressa (L.f.) R. Br.	Forage/Fodder	Africa, India, Australia	(Hanelt, 2001)
Heteropogon contortus (L.) P. Beauv. ex Roem. & Schult.	Forage/Fodder, Erosion control	India, Australia, Africa, USA	(Hanelt, 2001)
Hyparrhenia hirta (L.) Stapf ex Prain	Mat and basket weaving, Forage/Fodder, erosion control	Tropics, Southern USA	(Hanelt, 2001)
Imperata cylindrica (L.) Raeuschel.	Ornamental, mat weaving, roof construction, fodder	S and SE Asia, N Africa	(Hanelt, 2001; Khyade et al., 2018)
Ischaemum rugosum Salisb.	Forage/Fodder	SE Asia	(Hanelt, 2001)
Iseilema prostratum (L.) Anderss.	Forage/Fodder	S Asia	(Hanelt, 2001)
Lasiurus scindicus Henrard	Dune fixation, cereal	India, Iraq	(Hanelt, 2001)
Leptothrium senegalense (Kunth) W.D. Clayton	Land remediation	Kenya	(Hanelt, 2001)

Species	Utilization	Area of cultivation	Reference
Miscanthus nepalensis (Trin.) Hack.	Ornamental in temperate regions	Temperate regions	('Miscanthus nepalensis | New Zealand Plant Conservation Network'; Chung and Kim, 2012)
Miscanthus sinensis Andersson	Ornamental, high yielding bioenergy crop	Worldwide	(Chung and Kim, 2012)
Panicum antidotale Retz.	Forage/Fodder, Erosion control	(Hanelt, 2001)	
Panicum coloratum L.	Forage/Fodder, Erosion control	Africa, Australia, USA	(Hanelt, 2001)
Panicum flexuosum Retz.	Cereal	Central India	(Ganapathy, 2017*b*)
Panicum miliaceum L.	Cereal	Worldwide	(Gomashe, 2017*b*)
Panicum repens L.	Forage/Fodder, Erosion control	Africa, India	(Hanelt, 2001)
Panicum turgidum Forssk.	Dune fixation, cereal	Arabia, Africa	(Hanelt, 2001)
Paspalum dilatatum Poir.	Forage/Fodder	Humid subtropics	(Hanelt, 2001)
Paspalum distichum L.	Forage/Fodder, Erosion control	USA, Argentina	(Hanelt, 2001)
Paspalum scrobiculatum L.	Cereal	India, Africa	(Hariprasanna, 2017*a*)
Paspalum vaginatum Sw.	Lawn grass	Worldwide	(Riefner Jr and Columbus, 2008; 'The Plant Database-*USDA-NRCS*', 2019)
Rottboellia cochinchinensis (Lour.) W.D. Clayton	Forage/Fodder	Humid tropics	(Hanelt, 2001)
Saccharum officinarum L. 10	Sugar, syrup, medicine, bioethanol (together with S. barberi Jeswiet and S. sinense Roxb.)	Tropics and subtropics	(Berding et al., 2004; James, 2014)
Saccharum spontaneum L.	Erosion control, Forage/Fodder, leaf vegetable	India, Phillipines, tropical America and Africa	(Hanelt, 2001)
Schizachyrium scoparium (Michx.) Nash	Ornamental	Worldwide	('The Plant Database-*USDA-NRCS*', 2019)
Sehima nervosum (Rottl.) Stapf	Forage/Fodder	India	(Hanelt, 2001)
Setaria italica (L.) P. Beauv.	Cereal	Worldwide	(Hariprasanna, 2017*b*)
Setaria palmifolia (J.Koenig) Stapf	Ornamental, cereal	Subtropics and tropics	(De Wet et al., 1979)
Setaria sphacelata (Schumach.) Stapf & Hubb. ex Prain	Forage/Fodder, Famine food	Tropics	(Hanelt, 2001)
Setaria verticillata (L.) P. Beauv.	Grain, Brewing	Africa	(Hanelt, 2001)
Setaria viridis (L.) P. Beauv.	Forage/Fodder	Europe, Central Asia, Indonesia	(Hanelt, 2001)
Snowdenia polystachya (Fresen.) Pilger	Forage/Fodder	Kenya	(Hanelt, 2001)
Sorghum arundinaceum (Desv.) Stapf ex Prain	Forage/Fodder, emergency food	Africa, Brazil	(Hanelt, 2001)
Sorghum bicolor (L.) Moench	Cereal, syrup, bioethanol and brooms	Worldwide	(Sanjana Reddy, 2017*b*)
Sorghum halepense (L.) Pers.	Forage/Fodder	Tropics and Subtropics	(Hanelt, 2001)
Sorghum purpureo-sericeum (Hochst.) Asch. & Schweinf. ex Schweinf.	Forage/Fodder	India	(Hanelt, 2001)
Sorghum virgatum (Hackel) Stapf ex Prain	Forage/Fodder	Africa, USA	(Hanelt, 2001)
Sorghum x almum Parodi	Forage/Fodder	Turkmenistan	(Nikitin and Geldikhanov, 1988; Avutkhonov et al., 2016)
Sorghum × drummondi (Nees ex. Steud.) Millsp. & Chase	Cereal, Forage/Fodder	Warm and tropical regions	('The Plant Database-*USDA-NRCS*', 2019)
Spodiopogon cotulifer (Thunb.) Hack. ex A. DC. & C. DC.	Erosion control	Japan	(Hanelt, 2001)
Sporobolus airoides (Torr.) Torr.	Forage/Fodder	Saudi Arabia	(Miller and Cope, 1996)
Stenotaphrum secundatum (Walter) Kuntze	Lawn grass	Saudi Arabia, UAE	(Genovesi et al., 2009)
Stipagrostis karelinii (Trin. & Rupr.) Tzvelev	Dune stabilization	Central Asia	(Hanelt, 2001)
Stipagrostis pennata (Trin.) de Winter	Dune stabilization	Russia	(Hanelt, 2001)
Stipagrostis pungens (Desf.) De Winter	Dune fixation, grain, fodder/forage	Collected for its grain in subsaharan Africa, recently in cultivation	(Hanelt, 2001; Gamoun and Louhaichi, 2018)
Thelepogon elegans Roth ex Roem. & Schult.	Forage/Fodder	India	(Hanelt, 2001)

Species	Utilization	Area of cultivation	Reference
Themeda quadrivalvis (L.) Kuntze	Forage/Fodder	India, Australia	(Hanelt, 2001)
Themeda triandra Forssk.	Forage/Fodder, famine food, erosion control	India, Australia, South Africa	(Hanelt, 2001)
Tripidium bengalense (Retz.) H.Scholz	Erosion control, fibre, basket and mat weaving	India	(Hanelt, 2001)
Tripidium ravennae (L.) H.Scholz	Ornamental, erosion control	Worldwide	(James, 2014; Vincent and Gardner, 2016)
Urochloa brizantha (A.Rich.) R.D.Webster	Forage/Fodder, Erosion control	Humid tropics	(Hanelt, 2001)
Urochloa deflexa (Schumach.) H.Scholz	Cereal	Africa	(Seetharam and Riley, 1986)
Urochloa distachya (L.) T.Q.Nguyen	Forage/Fodder, Erosion control	Australia, SE Asia	(Hanelt, 2001)
Urochloa mutica (Forssk.) T.Q.Nguyen	Forage/Fodder, Erosion control	Tropics and Subtropics	(Hanelt, 2001)
Urochloa panicoides P. Beauv.	Cereal, Forage/Fodder	India, Australia	(Hanelt, 2001)
Urochloa ramosa (L.) T.Q.Nguyen Nair 11	Cereal, Forage/Fodder	S India, USA	(Seetharam and Riley, 1986)
*Urochloa trichopus (*Hochst.) Stapf ex Prain	Cereal, Forage/Fodder	India	(Hanelt, 2001)
Zea mays L.	Cereal	Worldwide	(Staller, 2010)
Dicots Acanthaceae			
Blepharis sp. 12	Seed as food additive and medicinal	Collected from the wild and traded in SW Asia	(Kripa and Vijayalakshmi, 2016)
Aizoaceae			
Sesuvium sesuvioides (Fenzl) Verdc. 13	Leaf vegetable, ornamental	Collected from the wild in littoral regions	(Cheikhyoussef et al., 2011)
Trianthema portulacastrum L.	Fodder, Medicinal, Leaf vegetable	SE Asia, Gabon	(Hanelt, 2001)
Amaranthaceae			
Aerva javanica (Burm.f.) Juss. ex Schult.	Fodder/Forage	Australia	(Hanelt, 2001)
Amaranthus blitum L.	Leaf vegetable, ornamental	India and Africa	(Costea et al., 2003; Das, 2016; 'PROTA4U web database', 2018)
Amaranthus caudatus L.	Pseudoceral, ornamental	Worldwide	(Sauer, 1967; Das, 2016)
Amaranthus cruentus L.	Pseudoceral, ornamental, leaf vegetable	Worldwide	(Sauer, 1967; Costea et al., 2003; Das, 2016)
Amaranthus dubius Mart. ex Thell.	Leaf vegetable	India, Africa	(Costea et al., 2003; Das, 2016; 'PROTA4U web database', 2018)
Amaranthus graecizans L.	Leaf vegetable	India	(Costea et al., 2003; Das, 2016; 'PROTA4U web database', 2018)
Amaranthus hypochondriacus L.	Pseudoceral, ornamental	Worldwide	(Sauer, 1967; Das, 2016)
Amaranthus retroflexus L.	Fodder/Forage	North Korea	(Hanelt, 2001)
Amaranthus tricolor L.	Leaf vegetable, ornamental	E and S Asia, Africa	(Costea et al., 2003; Das, 2016; 'PROTA4U web database', 2018)
Amaranthus viridis L.	Leaf vegetable	(Hanelt, 2001)	
Gomphrena globosa L.	Ornamental	Worldwide	(Hanelt, 2001)
Gomphrena haageana Klotzsch	Ornamental	Worldwide	('Missouri Botanical Garden - Plant Finder')
Asteraceae			
Flaveria bidentis (L.) Kuntze	Medicinal	Brazil	(Hanelt, 2001)
Flaveria trinervia (Spreng.) C. Mohr	Medicinal	South America	(Hanelt, 2001)
Chenopodiaceae			
Anabasis aphylla L.	Alkaloid extraction, medicine	(Hanelt, 2001)	
Atriplex canescens (Pursh) Nutt.	Fodder/Forage, erosion control	Iran, Israel/palestine	(Hanelt, 2001)
Atriplex halimus L.	Leaf vegetables, seeds, Land remediation, Erosion control	SW Asia, N Africa, Mediterranian, India	(Danin; Hanelt, 2001)
Atriplex leucoclada Boiss.	Fodder/Forage, Land remediation, erosion control	Investigated for cultivation	(Louhaichi et al., 2017)
Atriplex repens Roth	Fodder/Forage	Central Asia, SE Europe	(Hanelt, 2001)
Atriplex rosea L.	Fodder/Forage	North America	(Hanelt, 2001)

Species	Utilization	Area of cultivation	Reference
Atriplex tatarica L.	Fodder/Forage	Central Asia	(Hanelt, 2001)
Bassia indica (Wight) A.J. Scott	Forage/Fodder	Egypt	(Hanelt, 2001)
Bassia prostrata (L.) A.J. Scott	Forage/Fodder, Soil-fixation	Central Asia, Europe, USA	(Hanelt, 2001)
Bassia scoparia (L.) A.J. Scott	Ornamental, leaf vegetable, edible seeds (caviar substitute)	Worldwide (ornamental), Japan (caviar)	('Useful Temperate Plants Database'; Han et al., 2006; Nedelcheva et al., 2007)
Caroxylon orientale (S.G.Gmel.) Tzvelev	Fodder/Forage	Uzbekistan, Kyrgyzstan	(Hanelt, 2001)
Caroxylon vermiculatum (L.) Akhani & Roalson	Fodder/Forage	Central Asia	(Hanelt, 2001)
Climacoptera turcomanica (Litv.) Botsch.	Erosion control	Central Asia	(Hanelt, 2001)
Halothamnus subaphyllus (C. A. Mey.) Botsch.	Fodder/Forage	Central Asia	(Hanelt, 2001)
Haloxylon ammodendron (C. A. Mey.) Bunge ex Fenzl	Sand and saline soil fixation, Fodder/Forage, Biomass	From China to Iran and SE Europe	(Hanelt, 2001)
Haloxylon persicum Bunge ex Boiss. & Buhse	Dune fixation, Fodder/Forage, Biomass	From China to North Africa	(Hanelt, 2001)
Hammada salicornica (Moq.) Iljin	Dune fixation, Fodder/Forage, Biomass	Experimental culture in Asian deserts	(Hanelt, 2001; Singh et al., 2015)
Horaninovia ulicina Fisch. & C. A. Mey.	Dune fixation, Fodder/Forage, Biomass	Central Asia	(Hanelt, 2001)
Salsola paulsenii Litv. (Syn. *Kali paulsenii* (Litv.) Akhani & Roalson)	Saline soil remediation, Fodder/Forage	Central Asia	(Hanelt, 2001)
Salsola praecox (Litv.) Litv. (Syn. *Kali praecox* (Litv.) Sukhorukov)	Dune fixation	Central Asia	(Hanelt, 2001)
Salsola tragus L. (Syn. *Kali tragus* (L.) Scop.)	Saline soil remediation, Fodder/Forage	India, Central Asia	(Hanelt, 2001)
Soda inermis Fourr. (Syn. *Salsola soda* L.)	Leaf vegetable	Mediterranian	(Centofanti and Bañuelos, 2015)
Soda stocksii (Boiss.) Akhani (Syn. *Salsola stocksii* Boiss.)	Dune fixation, Fodder/Forage, Biomass	India, Pakistan	(Hanelt, 2001; Rathore et al., 2012)
Suaeda aegyptiaca (Hasselq.) Zohary 14	Leaf vegetable	Collected and cultivated in S Iran	(Akhani, 2006)
Suaeda fruticosa Forssk.	Saline soil remediation, biomass	India	(Hanelt, 2001)
Suaeda monoica Forssk.	Saline soil remediation, biomass	India	(Hanelt, 2001)
Xylosalsola paletzkiana (Litv.) Akhani & Roalson	Dune fixation, Fodder/Forage, Biomass	Central Asia, Turkmenistan	(Hanelt, 2001)
Xylosalsola richteri (Moq.) Kar. ex Litw.	Dune fixation, Fodder/Forage, Biomass, Medicine	Central Asia, SE Europe, Turkmenistan	(Hanelt, 2001)
Cleomaceae			
Cleome gynandra L.	Leaf vegetable, seed oil, ornamental	Africa, SE Asia, Carribean	(Hanelt, 2001; CHeikhyoussef et al., 2011; 'PROTA4U web database', 2018)
Nyctaginaceae			
Boerhavia sp. 15	Leaf vegetable, seeds as bread additive, medicine	Traditionally collected in the wild in India and South Arabia	(Mahesh et al., 2012; Ammar et al., 2016)
Polygonaceae			
Calligonum sp.	Dune stabilization, biomass, food, fodder/forage	C, S, SW Asia, N Africa, SE Europe	(Hanelt, 2001)
Portulacaceae			
Portulaca oleracea L.	Leaf vegetable, seed and oil	Widespread weed, regionally cultivated in Asia, N Africa and Europe	(Gonnella et al., 2010; Uddin et al., 2014)
Portulaca grandiflora W.J. Hook	Ornamental	Worldwide	(Jia et al., 2017)
Portulaca pilosa L.	Ornamental	America, SE Asia	(Hanelt, 2001)
Portulaca quadrifida L.	Leaf vegetable, Medicinal	Africa, SE Asia, India	(Hanelt, 2001)
Zygophyllaceae			
Tribulus terrestris L. 16	Whole plant used as food and medical supplement	Cultivated in S and E Europe, India	(Hanelt, 2001; Boteva et al., 2014; Salamon et al., 2016)

Notes:1. Other stoloniferus *Cyperus* sp., like *C. amauropus* Steud. *C. bulbosus* Vahl, *C. compressus* L., *C. cyperoides* (L.) Kuntze, *Cyperus longus* L. and *C. rotundus* L. also bear edible tubers, although not cultivated but collected from the wild. 2. *C. biflorus* Roxb., *C. ciliaris* L., *C. pennisetiformis* Hochst. ex Steud., *C. prieurii* (Kurth) Maire, *C. clandestinus* (Hochst. ex Chiov.) Morrone, *C. divisus* (J.F.Gmel.) Verloove, *C. flaccidus* (Griseb.)

Morrone, *C. macrourus* (Trin.) Morrone, *C. orientalis* (Rich.) Morrone, *C. polystachios* (L.) Morrone, *C. purpureus* (Schumach.) Morrone, *C. setaceus* (Forssk.) Morrone, *C. sieberianus* (Schltdl.) Verloove, *C. stramineus* (Peter) Morrone and *C. setiger* Vahl are cultivated as forage/fodder throughout the tropics and warm regions of the world. 3. Six *Chrysopogon* species distributed in SW Asia. 4. A second species *C. aquatica* Roxb. Grows wild in Afghanistan. 5. *C. martinii* (Roxb.) Wats.*C. iwarancusa* (Jones) Schult. ex Roem. & Schult. and 6 other *Cympogogon* sp. are distributed within SW Asia and are used locally. 6. A number of 21 *Digitaria* species are distributed in SW Asia: *D. exilis* (Kippst.) Stapf and *D. iburua* Stapf (Africa), *D. sanguinalis* (L.) Scop. (Europe and N America) and *D. compacta* (Roth ex Roem. & Schult.) Veldkamp and *D. cruciata* (Ness) A. Camus (E India) grown as cereals: *D. abyssinica* (Hochst.) Stapf (S.Africa), *D. ciliaris* (Retz.) Koeler (USA), *D. sanguinalis* (L.) Scop. (N. America), *D. ternata* (A.Rich.) Stapf ex Thiselton-Dyer (Nigeria) are grown as Forage/Fodder: *D. longiflora* (Retz.) Pers. (Sri Lanka) is grown as lawn. 7. Its wild relative *E. crus-galliis* (L.) P. Beauv. Sometimes. collected and has been cultivated in the past for its grain.8. Four more *Eleusine* species grow in SW Asia. 9. 36 species of *Eragrostis*, including direct relatives of *E. tef* L. (e.g. eglandulate forms of *E. pilosa* (L.) P. Beauv.)) are widespread throughout SW Asia. 10. Six further species of *Saccharum* are distributed throughout SW Asia and used locally for medicinal purposes. 11. 15-18 wild *Urochloa* sp. (including the two cultivated species) are distributed in SW Asia. 12. Recent domestication and cultivation efforts. 13. Recent domestication and cultivation efforts. 14. Further 21 *Suaeda* sp. distributed in SW Asia and some collected as leaf vegetables. 15. Recent efforts of domestication. 16. Other two species also distributed in SW Asia.

References:

Akhani, H. (2006). Biodiversity of halophytic and sabkha ecosystems in Iran. Tasks for Vegetation Science. Sabkha Ecosystems Volume II: West and Central Asia. Springer, 71–88. doi: 10.1007/978-1-4020-5072-5_6

Ammar A, Zhang H, Chamba M, et al. 2016. Physicochemical and Cooking Properties of a Novel Food: Alhydwan (*Boerhavia elegans* Choisy) Seed Flour. Journal of Academia and Industrial Research **4**.

Arafat S, Gaafar A, Basuny A, Nassef S. 2009. Chufa Tubers (*Cyperus esculentus* L.): As a New Source of Food. World Applied Sciences Journal **7**, 151–156.

Assefa K, Chanyalew S, Tadele Z. 2017. Tef, *Eragrostis tef* (Zucc.) Trotter. Millets and Sorghum: Biology and Genetic Improvement. Editor(s): J. V. Patil. John Wiley & Sons Ltd, 226–256.

Atala A. 2012. A new ingredient: The introduction of priprioca in gastronomy. International Journal of Gastronomy and Food Science **1**, 61–63.

Avutkhonov B, Safarov A, Safarov K. 2016. Physiological peculiarities of Columbus grass (*Sorghum almum* Parodi) in Samarkand region conditions of Uzbekistan. European science review, 7–8.

Belgacem A, Louhaichi M. 2014. Managing rangelands: promoting sustainable grass/forage species: Buffel Grass: A resilient, drought-tolerant forage species to alleviate feed shortages and feeding costs. The International Center for Agriculture Research in the Dry Areas (ICARDA).

Berding N, Hogarth M, Cox M. 2004. Plant Improvement of Sugarcane. Sugarcane Editor(s): Glyn James. Blackwell Publishing Ltd, 20–53.

Bertea M, Maffei M. 2009. Botany, including anatomy, physiology, biochemistry and molecular biology. Essential Oil-Bearing Grasses: The genus *Cymbopogon*. Taylor & Francis, London, Editors: Akhila A., 1–24.

Boteva H, Dintcheva T, Masheva S, Yankova V, Markova D. 2014. Opportunities for Growing *Tribulus terrestris* L. as Semi-Culture. Biotechnology & Biotechnological Equipment **25**, 2388–2390.

Centofanti T, Bañuelos G. 2015. Evaluation of the halophyte Salsola soda as an alternative crop for saline soils high in selenium and boron. Journal of Environmental Management **157**, 96–102.

Cheikhyoussef A, Mapaure I, Shapi M. 2011. The use of some Indigenous Plants for Medicinal and other Purposes by Local Communities in Namibia with Emphasis on Oshikoto Region: A Review. Research Journal of Medicinal Plant **5**, 406–419.

Chung J, Kim D. 2012. Miscanthus as a potential bioenergy crop in East Asia. Journal of Crop Science and Biotechnology **15**, 67–77.

Costea M, Tardif F, Brenner D. 2003. The Identity of a Cultivated Amaranthus from Asia and a New Nomenclatural Combination. Economic Botany **57**, 646–649.

Danin A. Edible Leaves and Fruits. Plant Stories. PublishedOnline, .

Das S. 2016. Amaranths: The Crop of Great Prospect. In: Das S, ed. *Amaranthus*: A Promising Crop of Future. Springer Singapore, 13–48.

De Wet J, Oestry-Stidd L, Cubero J. 1979. Origins and evolution of foxtail millets (*Setaria italica*) - Persée. Journal d'agriculture traditionnelle et de botanique appliquée: JATBA **26**, 53–64.

Duke J. 1983. Handbook of Energy Crops Index. Published online - NewCROPS.

Duke J. 1993. CRC handbook of alternative cash crops. CRC Press.

Gamoun M, Louhaichi M. 2018. Managing rangelands: promoting and establishing sand dune fixing species: *Stipagrostis pungens* (Desf.) De Winter: a xerophytic quicksand- and dune-fixing species adapted to sandy deserts. International Center for Agricultural Research in the Dry Areas (ICARDA).

Ganapathy K. 2017a. Improvement in Finger Millet: Status and Future Prospects. Millets and Sorghum: Biology and Genetic Improvement. Editor(s): J. V. Patil. John Wiley & Sons Ltd, 87–111.

Ganapathy K. 2017b. Genetic Improvement in Little Millet. Millets and Sorghum: Biology and Genetic Improvement Editor(s): J. V. Patil. John Wiley & Sons Ltd, 170–183.

Genovesi A, W. Jessup R, C. Engelke M, L. Burson B. 2009. Interploid St. Augustinegrass [*Stenotaphrum secundatum* (Walt.) Kuntze] hybrids recovered by embryo rescue. In Vitro Cellular & Developmental Biology - Plant **45**.

Gomashe S. 2017a. Barnyard Millet: Present Status and Future Thrust Areas. Millets and Sorghum: Biology and Genetic Improvement Editor(s): J. V. Patil. John Wiley & Sons Ltd, 184–198.

Gomashe S. 2017b. Proso Millet, *Panicum miliaceum* (L.): Genetic Improvement and Research Needs. Millets and Sorghum: Biology and Genetic Improvement. Editor(s): J. V. Patil. John Wiley & Sons Ltd, 150–169.

Gonnella M, Charfeddine M, Conversa G, Santamaria P. 2010. Purslane: A Review of its Potential for Health and Agricultural Aspects. The European Journal of Plant Science and Biotechnology **4**, 131–136.

Han L, Nose R, Li W, Gong X, Zheng Y, Yoshikawa M, Koike K, Nikaido T, Okuda H, Kimura Y. 2006. Reduction of fat storage in mice fed a high-fat diet long term by treatment with saponins prepared from Kochia scoparia fruit. Phytotherapy Research **20**, 877–882.

Hanelt P. 2001. Mansfeld's Encyclopedia of Agricultural and Horticultural Crops. Springer Verlag Berlin Heidelberg.

Hariprasanna K. 2017a. Kodo Millet, *Paspalum scrobiculatum* L. Millets and Sorghum: Biology and Genetic Improvement. Editor(s): J. V. Patil. John Wiley & Sons Ltd, 199–225.

Hariprasanna K. 2017b. Foxtail Millet, Setaria italica (L.) P. Beauv. - Millets and Sorghum - Wiley Online Library. Millets and Sorghum: Biology and Genetic Improvement. Editor(s): J. V. Patil. John Wiley & Sons Ltd, 112–149.

Jain S, Banerjee D. 1974. Preliminary observations on the ethnobotany of the genus *Coix*. Economic Botany **28**, 38–42.

James G. 2014. An Introduction to Sugarcane. Sugarcane. Blackwell Publishing Ltd, 1–19.

Jana K, Puste A. 2014. Madur Kathi – An Important Economic Non-food Crop of West Bengal. Asian Agri-History **18**, 145–151.

Jia S, Yan Z, Wang Y, Wei Y, Xie Z, Zhang F. 2017. Genetic diversity and relatedness among ornamental purslane (*Portulaca* L.) accessions unraveled by SRAP markers. 3 Biotech **7**, 241.

Khyade V, Pawar S, Sarwade J. 2018. Novel Sacrificial Medicinal Repositories: Halfa grass, *Desmostachya bipinnata* (L.) and Cogon grass, *Imperata cylindrica* (L.). World Scientific News **100**, 35–50.

Kripa K, Vijayalakshmi S. 2016. Therapeutic uses of plants of genus *Blepharis* - a systematic review. International Journal of Pharma and Bio Sciences **7**, 236–243.

Kumawat R, Louhaichi M, Misra A. 2015. Managing rangelands: promoting sustainable grass forage species: Birdwood grass (*Cenchrus setigerus*): A resilient, drought-tolerant perennial grass for pastures in hot, dry areas. The International Center for Agriculture Research in the Dry Areas (ICARDA).

Louhaichi M, Belgacem A, Hassan S. 2017. Managing rangelands: promoting well-adapted shrub species: Atriplex leucoclada: mitigating highly saline soils. International Center for Agricultural Research in the Dry Areas (ICARDA).

Mahesh A, Kumar H, Ranganath M, Devkar R. 2012. Detail Study on *Boerhaavia diffusa* Plant for its Medicinal Importance-A Review. Research Journal of Pharmaceutical Sciences **1**, 28–36.

Miller A. and Cope T. (1996). Flora of the Arabian Peninsula and Socotra. Edinburgh University Press.

Miscanthus nepalensis | New Zealand Plant Conservation Network.

Missouri Botanical Garden - Plant Finder.

Nedelcheva A, Dogan Y, Maria Guarrera P. 2007. Plants traditionally used to make brooms in several European countries. Journal of ethnobiology and ethnomedicine **3**, 20.

Nesbitt M. 2005. Grains. The Cultural History of Plants. Routledge, 45–60.

Nikitin V, Geldikhanov A. 1988. Opredelitel rastenij Turkmenistana. Academy of sciences of the Turkmen SSR.

Pareek A, Kumar A. 2013. Ethnobotanical and pharmaceutical uses of *Vetiveria zizanioides* (Linn) Nash: a medicinal plant of Rajasthan. International Journal of Life Scence and Pharma Research **3**.

Pascual B, Maroto J, Lopez-Galarza S, Sanbautista A. 2000. Chufa (*Cyperus esculentus* L. var. sativus Boeck.): Unconventional crop. Studies related to applications and cultivation | Request PDF. Economic Botany **54**, 439–448.

PROTA4U web database. 2018. PROTA4U.

Rathore V, Singh J, Roy M. 2012. *Haloxylon stocksii* (Boiss.) Benth. et Hook. f., a promising halophyte: distribution, cultivation and utilization. Genetic Resources and Crop Evolution **59**, 1213–1221.

Ravichandran P, Thumilan M, Benazir J, Manimekalai V. 2005. Anatomy and Vascular Bundle Diversity in Mat sedges. Phytomorphology **55**, 75–83.

Riefner Jr R, Columbus J. 2008. *Paspalum vaginatum* (Poaceae), a new threat to wetland diversity in southern California. Journal of the Botanical Research Institute of Texas **2**, 743–759.

Sahu S, Rout N, Dhal N. 2010. Ethnobotany of *Eulaliopsis binata* (Retz.) Hubbard- Poaceae, in Odisha, Eastern India: Cultivation Practice, Economics and Prospects. Journal of Advances in Developmental Research **1**, 155–160.

Salamon I, Grulova D, De Feo V. 2016. Comparison of two methods for field grow of puncture vine (*Tribulus terrestris* L.) in Slovakia. Acta Agriculturae Scandinavica, Section B - Soil & Plant Science **66**, 267–271.

Sanjana Reddy P. 2017a. Pearl Millet, *Pennisetum glaucum* (L.) R. Br. Millets and Sorghum: Biology and Genetic Improvement Editor(s): J. V. Patil. John Wiley & Sons Ltd, 49–86.

Sanjana Reddy P. 2017b. Sorghum, *Sorghum bicolor* (L.) Moench - Millets and Sorghum - Wiley Online Library. Millets and Sorghum: Biology and Genetic Improvement. Editor(s): J. V. Patil. John Wiley & Sons Ltd, 1–48.

Sauer J. 1967. The Grain Amaranths and Their Relatives: A Revised Taxonomic and Geographic Survey. Annals of the Missouri Botanical Garden **54**, 103–137.

Seetharam A, Riley K. 1986. Small Millets in Global Agriculture: Proceedings of the First International Small Millets Workshop, Bangalore, India, October 29-November 2, 1986. Oxford & IBH Publishing Company.

Shioya M, Myoga A, Kitagawa A, Tokunaga Y, Hayashi H, Kogo Y, Shimada H, Satake S. 2019. Analysis of deflection and dynamic plant characteristics of *Cyperus malaccensis* Lam. Plant Production Science **22**, 1–8.

Simpson D, Inglis C. 2001. Cyperaceae of Economic, Ethnobotanical and Horticultural Importance: A Checklist. Kew Bulletin **56**, 257–360.

Singh J, Rathore V, Roy M. 2015. Notes about *Haloxylon salicornicum* (Moq.) Bunge ex Boiss., a promising shrub for arid regions. Genetic Resources and Crop Evolution **62**, 451–463.

Staller J. 2010. Ethnohistory: Impressions and Perceptions of Maize. In: Staller J, ed. Maize Cobs and Cultures: History of *Zea mays* L. Berlin, Heidelberg: Springer Berlin Heidelberg, 7–83.

Toderich K, Black C, Juylova E, Kozan O, Mukimov T, Matuso N. 2007. C_3/C_4 plants in the vegetation of Central Asia, geographical distribution and environmental adaptation in relation to climate. Climate Change and Terrestrial Carbon Sequestration in Central Asia. CRC Press, 33–63.

The Plant Database-*USDA-NRCS*. 2019. The Plant Database National Plant Data Team, Greensboro, NC 27401-4901 USA.

Uddin M, Juraimi A, Hossain M, Nahar M, Ali M, Rahman M. 2014. Purslane Weed (Portulaca oleracea): A Prospective Plant Source of Nutrition, Omega-3 Fatty Acid, and Antioxidant Attributes. The Scientific World Journal **2014**.

Useful Temperate Plants Database.

Vincent M, Gardner R. 2016. Spread of the invasive Ravenna grass (*Tripidium ravennae*, Poaceae) in Ohio. Phytoneuron **78**, 1–9.

Wendrich W. 1989. Preliminary report on the Amarna basketry and cordage. Amarna Reports V. Egypt Exploration Society, 169–201.
