# Supplementary material for: A Review of C4 Plants in Southwest Asia: An Ecological, Geographical and Taxonomical Analysis of a Region With High Diversity of C4 Eudicots
Source: Front Plant Sci. 2020 Nov 5;11:546518. doi: 10.3389/fpls.2020.546518 (PMC7694577; doi:10.3389/fpls.2020.546518)
Supplement: Supplementary Data Sheet 1 — References of floristic database. [file DataSheet_1.pdf]

__**Supplementary data 1:**__

A) Sources for database:

a) Country floras (Flora of Pakistan; Davis, 1966-2001; K. H. Rechinger, ed.; Post, 1933; Karyagin, 1950-1961; Takhtajian, 1954-2009; Guest and Ghazanfar, 1966-2013; Mouterde, 1966; Zohary, 1966; Takhtajian and Fedorov, 1972; Tackholm, 1974; Nikitin and Geldikhanov, 1988; Miller and Cope, 1996; Al-Eisawi, 1998; Boulos, 1999; Breckle and Rafiqpoor, 2010; Breckle *et al.*, 2013)

b) Monographs, revisions, reports and other scientific publications (Brullo 1982; Lamarck and Poiret, 1810; Edgecombe, 1970; Scholz and Gabriel, 1973; Termeh, 1975, 1987, 2000; Al-Eisawi, 1982, 1983; Assadi and Runemark, 1983; Mozaffarian, 1985, 1994; Pratov, 1986; Sümbül, 1991; Boulos, 1992; Baierle, 1993; Kothe-Heinrich, 1993; Akhani, 1996, 2015; Al-Hemaid and Thomas, 1996; Akhani and Scholz, 1998; Assadi, 1998; Fragman, 1999; Freitag *et al.*, 1999; Rilke, 1999; Freitag and Duman, 2000; Peterson *et al.*, 2001; Mutlu, 2002; Csiky *et al.*, 2004; Danin, 2004; Ghobadnejhad *et al.*, 2004; Hadjikyriakou *et al.*, 2004; Miller *et al.*, 2004; Akhani *et al.*, 2005, 2007, 2008, 2012, 2016; Aminirad, 2005; McKwon *et al.*, 2005; Väre and Kukkonen, 2005; El-Bana, 2006; Varghese *et al.*, 2006; Parolly and Eren, 2007; Sukhorukov, 2007; Ghasemkhani *et al.*, 2008; Seçmen and Leblebici, 2008; Hamzehei and Naghinezhad, 2009; Amini Rad and Scholz, 2011; Hakobyan, 2011; Maassoumi, 2011; Pahlevani and Akhani, 2011; Soskov, 2011; Sukhorukov *et al.*, 2011; Danin and Bagella, 2012; Khodashenas and Joharchi, 2012; Mosti *et al.*, 2012; Verloove and Sanchez Gullon, 2012; Yaprak and Baskoese, 2012; Akhani and Khoshravesh, 2013; Uotila *et al.*, 2014; Brullo *et al.*, 2015; El Naggar *et al.*, 2015; Fisher *et al.*, 2015; Pahlevani, 2017; Basahi and Masrahi, 2019)

c) Data from Herbarium collections (Akhani Herbarium, Edinburgh Herbarium, Komarov Institute Herbarium).

d) Electronic databases (Watson *et al.*, 1992; Euro+Med, 2006-2018; Royal Botanic Garden Edinburgh - Herbarium catalogue, 2018; Seregin, 2019; Flora of Israel Online; POWO, 2019; Musselman, 2007; Lebanon flora)

e) Personal communication for Cyperaceae of the Arabian Peninsula, kindly provided by Dr. David A. Simpson (Royal Botanical Gardens Kew).

References:

Akhani, H. (1996). A new species and a synonym in Chenopodiaceae from Iran. *Sendtnera : Mitteilungen der Botanischen Staatssammlung und des Instituts für Systematische Botanik der Universitat München.* 3, 5–10.

Akhani, H. (2015). Plants and Vegetation of North-West Persian Gulf: The coasts and Islands of Khore Musa, Mahshahr and Adjacent Areas. *University of Tehran Press.*

Akhani, H. and Khoshravesh, R. (2013). The relationship and different C4 Kranz anatomy of *Bassia eriantha* and *Bassia eriophora,* two often confused Irano-Turanian and Saharo-Sindian species. *Phytotaxa* 93, 1–24. doi: 10.11646/phytotaxa.93.1.1

Akhani, H. and Scholz, H. (1998). Studies on the flora and vegetation of the Golestan National Park, NE Iran. II. A new *Poa* and some new and noteworthy grass records for Iran. *Edinburgh Journal of Botany* 55, 443–453. doi: 10.1017/S0960428600003280

Akhani, H., Barroca, J., Koteyeva, N., Voznesenskaya, E., Franceschi, V., Edwards, G., Ghaffari, S. and Ziegler, H. (2005). *Bienertia sinuspersici* (Chenopodiaceae): A New Species from Southwest Asia and Discovery of a Third Terrestrial C4 Plant Without Kranz Anatomy. *Systematic Botany* 30, 290–301. doi: 10.1600/0363644054223684

Akhani, H., Chatrenoor, T., Dehghani, M., Khoshravesh, R., Mahdavi, P. and Matinzadeh, Z. (2012). A new species of *Bienertia* (Chenopodiaceae) from Iranian salt deserts: A third species of the genus and discovery of a fourth terrestrial C_4 plant without Kranz anatomy. *Plant Biosystems* 146, 1–10. doi: 10.1080/11263504.2012.662921

Akhani, H., Edwards, G. and Roalson, E. (2007). Diversification of the Old World Salsoleae s.l. (Chenopodiaceae): Molecular Phylogenetic Analysis of Nuclear and Chloroplast Data Sets and a Revised Classification. *International Journal of Plant Sciences* 168, 931–956. doi: 10.1086/518263

Akhani, M., Ghasemkhani, M., Chuong, S. and Edwards, G. (2008). Occurrence and forms of Kranz anatomy in photosynthetic organs and characterization of NAD-ME subtype C_4 photosynthesis in *Blepharis ciliaris* (L.) B. L. Burtt (Acanthaceae). *Journal of Experimental Botany* 59, 1755–1765. doi: 10.1093/jxb/ern020

Akhani, H., Khoshravesh, R. and Malekmohammadi, M. (2016). Taxonomic novelties from Irano-Turanian region and NE Iran: *Oreosalsola*, a new segregate from *Salsola* s.l., two new species in *Anabasis* and *Salvia* , and two new combinations in *Caroxylon* and *Seseli*. *Phytotaxa* 249, 159–180. doi: 10.11646/phytotaxa.249.1.7

Al-Hemaid, F. and Thomas, J. (1996). Review of the genus *Tribulus* L. in Saudi Arabia. *Arab Gulf Journal of Scientific Research* 14, 415–443.

Al-Eisawi, D. (1982). List of Jordan vascular pants. *Mitteilungen der Botanischen Staatssammlung München* 18, 79–182.

Al-Eisawi, D. (1983). Studies on the flora of Jordan. 10. Nine new species to the flora of Jordan. *Candollea* 38, 359–364.

Al-Eisawi, D. (1998). Field guide to wild flowers of Jordan and neighbouring countries. *Jordan Press Foundation.*

Amini Rad, M. and Scholz, H. (2011). *Urochloa panicoides* and a subspecies of *Eragrostis cilianensis* (Poaceae) new to the flora of Iran. *Rostaniha (Botanical Journal of Iran)* 12, 195–198. doi: 10.22092/botany.2012.101420

Amini Rad, M. (2005). New records and interseting species of Cyperaceae family from Iran. *The Iranian Journal of Botany* 11, 49–53.

Assadi, M. (1998). Noteworthy plant records from Iran. *Iranian Journal of Botany* 7, 217–220.

Assadi, M. and Runemark, H. (1983). Notes on the flora and vegetation of S. Baluchistan, Iran. *Iranian Journal of Botany* 2, 69–78.

Baierle, H.U. (1993). Vegetation und Flora im südwestlichen Jordanien. *Dissertationes Botanicae 200, 1-254.*

Basahi, M., Masrahi, Y. (2019). *Blepharis saudensis* (Acanthaceae), a new species from Saudi Arabia. *Saudi Journal of Biological Sciences,* 26, 1509-1512. doi: 10.1016/j.sjbs.2019.01.002

Boulos, L. (1992). Notes on *Agathophora* (Fenzl) Bunge and *Cornulaca* Del. *Studies in the Chenopodiaceae of Arabia 5. Kew Bulletin 47, 283–287. doi: 10.2307/4110669*

Boulos, L. (1999). Flora of Egypt. *Al Hadara Publishing.*

Breckle, S., Hedge, I. And Rafiqpoor, M. (2013). Vascular Plants of Afghanistan: An Augmented Checklist. *Scientia Bonnensis, Bonn, Manama, New York, Florianapólis*.

Breckle, S. and Rafiqpoor, M. (2010). Field Guide Afghanistan - Flora and Vegetation. Deutsche Nationalbibliothek.*Scientia Bonnensis, Bonn, Manama, New York, Florianapólis*.

Brullo, S. (1982) Notes on the Genus *Salsola* (Chenopodiaceae). 1. The *Salsola oppositifolia* and *S. longifolia* Groups. *Willdenowia* 12, 241–247.

Brullo, C., Brullo, S., Gaskin, J., Galdo, G., Hrusa, G. and Salmeri, C. (2015). A new species of *Kali* (Salsoloideae, Chenopodiaceae) from Sicily, supported by molecular analysis. *Phytotaxa* 201, 256–277. doi: 10.11646/phytotaxa.201.4.2

Csiky, J., Király, G., Oláh, E., Pfeiffer, N. and Virók, V. (2004). *Panicum dichotomiflorum* Michaux., a new element in the Hungarian flora. *Acta Botanica Hungarica* 46, 137–141. doi: 10.1556/ABot.46.2004.1-2.9

Danin, A. (2004). Distribution atlas of plants in the Flora Palaestina area. *Jerusalem: Israel academy of sciences and humanities.*

Danin, A. and Bagella, S. (2012). A new cultivar microspecies of the *Portulaca oleracea* aggregate from the E Mediterranean. Willdenowia 42, 63–65. doi: 10.3372/wi42.42106

Danin, A. and Fragman-Sapir, O. (2016+) Flora of Israel Online. http://flora.org.il/en/plants/

Davis, P. (1966-2001) Flora of Turkey and the East Aegean Islands. *Edinburgh University Press.*

Edgecombe, W.S. (1970). Weeds of Lebanon. *American University of Beirut, Beirut, Lebanon.*

El-Bana, M. (2006). Floristic composition of a threatened Mediterranean sabkhat of Sinai. *Tasks for Vegetation Science. Sabkha Ecosystems: Volume II: West and Central Asia. Dordrecht: Springer Netherlands,* 155–162. doi: 10.1007/978-1-4020-5072-5_12

El Naggar, S., El-Hadidy, A. and Olwey, A. (2015). Taxonomic revision of the genus *Heliotropium* (Boraginaceae s.l.) in south Yemen. *Nordic Journal of Botany* 33, 401–413. doi: 10.1111/njb.00746

Euro+Med (2006-): Euro+Med PlantBase - the information resource for Euro-Mediterranean plant diversity. *Published on the Internet* http://ww2.bgbm.org/EuroPlusMed/ [Accessed 15.10,18]

Fisher, A., McDade, L., Kiel, C., Khoshravesh, R., Johnson, M., Stata, M., Sage, T. and Sage, R. (2015). Evolutionary History of *Blepharis* (Acanthaceae) and the Origin of C_4 Photosynthesis in Section *Acanthodium*. *International Journal of Plant Sciences* 176, 770–790. doi: 10.1086/683011

Flora of Pakistan - Pakistan Plant Database. *Published Online:* https://www.tropicos.org/Project/Pakistan [Accessed 27.09.2018]

Fragman, O. (1999). Checklist and ecological database of the flora of Israel and its surroundings including Isreael, Jordan, The Palestinian Autonomy, Golan Heights, Mt Hermon and Sinai. *Jerusalem : Israel Nature & National Parks Protection Authority.*

Freitag, H. and Duman, H. (2000). An unexpected new taxon of *Salsola* (Chenopodiaceae) from Turkey. *Edinburgh Journal of Botany 57, 339–348. doi: 10.1017/S0960428600000354*

Freitag, H., Vural, M. and Adiguzel, N. (1999). A remarkable new *Salsola* and Some new records of Chenopodiaceae from Central Anatolia, Turkey. *Willdenowia 29, 123–139. doi: 10.3372/wi.29.2911*

Ghasemkhani, M., Akhani, H., Sahebi, J. and Scholz, H. (2008). The genera *Aristida* and *Stipagrostis* (Poaceae) in Iran. Willdenowia 38, 135–148. doi: 10.3372/wi.38.38108

Ghobadnejhad, M., Joharchi, M., Akhani, H. (2004). Notes on the flora of Iran 5: *Halimocnemis longifolia* (Chenopodiaceae) a new record from Iran. *Linzer biologische Beitraege 36, 1309–1316.*

Guest, E. and Ghazanfar, S. (1966-2013). Flora of Iraq. *Royal Botanic Gardens, Kew, Bentham-Moxon Trust*.

Hadjikyriakou, F., Makris, C., Christofides, Y. and Alziar, G. (2004). Additions to the flora of Cyprus. *Journal de Botanique* 27, 31–46.

Hakobyan, J. (2011). The genus *Salsola* sensu lato (Chenopodiaceae) in southern Transcaucasia. Takhtajania 1, 124–132.

Hamzehei, B. and Naghinezhad, A. (2009). *Arthraxon* P. Beauv. (Gramineae) and *Carex caryophyllea* (Cyperaceae), new genus and species records from Iran. *The Iranian Journal of Botany*, 15, 68–71.

Karyagin, I. (1950-1961). Flora Azerbayjana. *Academy of sciences of Azerbayjan SSR*.

Khodashenas, M. and Joharchi, M. 2012. *Aeluropus laciniatus* (Poaceae), A New Species From Iran. *Iranian Journal of Botany*, 18, 64–66.

Kothe-Heinrich, G. (1993). Revision der Gattung *Halothamnus* (Chenopodiaceae). *Bibliotheca Botanica*, Heft 143.

Lamarck, J. and Poiret, J. (1810). Encyclopédie méthodique. Botanique. Supplement. *Biodiversity Heritage Library. Paris,Agasse*.

Lebanon flora. Available from http://www.lebanon-flora.org. [Accessed 20.09.2018]

McKwon, A., Moncalvo, J. and Dengler, N. (2005). Phylogeny of *Flaveria* (Asteraceae) and inference of C_4 photosynthesis evolution. *American Journal of Botany* 92, 1911–192. doi: 10.3732/ajb.92.11.1911

Miller, A. and Cope, T. (1996). Flora of the Arabian Peninsula and Socotra. *Edinburgh University Press*.

Miller, A., Morris, M., Atkinson, R. and Alexander, D. (2004). Ethnoflora of the Soqotra Archipelago. *Edinburgh: Royal Botanic Garden Edinburgh*.

Mosti, S., Raffaelli, M., Tardelli, M. (2012). Contribution to the Flora of Central-Southern Dhofar (Sultanate of Oman). *Webbia* 67, 65–91. doi: 10.1080/00837792.2012.10670909

Mouterde, P. (1966). Nouvelle Flore du Liban et de la Syrie. *Editions de l'Impr. Catholique*.

Mozaffarian, V. (1985). New species and new plant records from Iran. *Iranian Journal of Botany* 3, 81–86.

Mozaffarian, V. (1994). Studies on the flora of Iran, new species and new records. *Iranian Journal of Botany* 6, 235–243.

Musselman, L.J. (2007). Checklist of Plants of the Hashemite Kingdom of Jordan – Old Dominion University. http://ww2.odu.edu/~lmusselm/plant/jordan/. [Accessed 30.6.2019]

Mutlu, B. (2002). New Floristic records from various squares in flora of Turkey. *Hacettepe Journal of Biology and Chemistry* 31, 17–22.

Nikitin, V., Geldikhanov, A. (1988). *Opredelitel rastenij Turkmenistana*. Academy of sciences of the Turkmen SSR.

Pahlevani A. 2017. Diversity of the genus Euphorbia (Euphorbiaceae) in SW Asia, *Universität Bayreuth*.

Pahlevani, A,. and Akhani, H. (2011). Seed morphology of Iranian annual species of *Euphorbia* (Euphorbiaceae). *Botanical Journal of the Linnean Society* 167, 212–234. doi: 10.1111/j.1095-8339.2011.01165.x

Parolly, G. and Eren, Ö. (2007). Contributions to the flora of Turkey, 2. *Willdenowia* 37, 243–272. doi: 10.3372/wi.37.37114

Peterson, P., Soreng, R., Davidse, G., Filgueiras, T., Zuloaga, F. and Judziewicz, E. (2001). Catalogue of New World Grasses (Poaceae): II. Subfamily Chloridoideae. *Contributions from the United States National Herbarium* 41, 1–255.

Post G. 1933. Flora of Syria, Palestine, and Sinai ed.2. *American University of Beirut: Publications of the Faculty of Arts and Sciences*.

POWO (2019). "Plants of the World Online. Facilitated by the Royal Botanic Gardens, Kew. Published on the Internet; http://www.plantsoftheworldonline.org/ [Accessed 20.07.2019]

Pratov, U. (1986). The genus *Climacoptera* Botsch. (systematics, geography, phylogeny, and conservation issues). *Fan, Tashkent.*

Rechinger, K.H. 1963-2015. Flora Iranica: Flora des iranischen Hochlandes und der umrahmenden Gebirge, , Fas. 1-181. *Graz, Austria: Akademische Druck - u. Verlagsanstalt.*

Rilke, S. (1999). Revision der Sektion Salsola s.l. der Gattung - *Salsola (Chenopodiaceae). E. Schweizerbart'sche Verlagsbuchhandlung (Nägele u. Obermiller).*

Royal Botanic Garden Edinburgh - Herbarium catalogue. https://data.rbge.org.uk/search/herbarium/ [Accessed 03.05.2018]

Scholz, H. and Gabriel, B. (1973). Neue Florenliste aus der libyschen Sahara (A New List of Plants to the Flora of the Libyan Sahara). *Willdenowia 7, 169–181.*

Seçmen, Ö. and Leblebici, E. (2008). Türkiye Sulak Alan Bitkileri ve Bitki Örtüsü. *Ege Üniversitesi Basimevi.*

Seregin, A. P. (2019). Moscow Digital Herbarium: Electronic resource. – *Moscow State University, Moscow.* https://plant.depo.msu.ru/ [Accessed 15.01.2019]

Soskov, Y. (2011). Rod *Calligonum* L. — zhuzgun (sistematika, geografiya, evolyutsiya, introduktsiya) [Genus *Calligonum* L. — zhuzgun (taxonomy, geography, evolution, introduction).] *Novosibirsk: Siberian Sci. Agric. Library.*

Sukhorukov, A. (2007). Notes on the taxonomy of *Girgensohnia* (Chenopodiaceae/Amaranthaceae). *Edinburgh Journal of Botany* 64, 317–330. doi: 10.1017/S0960428607004751

Sukhorukov, A., Hakobyan, J. and Zernov, A. (2011). On some new and critical taxa of the Family Chenopodiaceae in the flora of the Caucasus. *Novosti Sistematiki Vysshikh Rastenii* 42, 106–110.

Sümbül, H. (1991). Ten new species from Anatolia and two new records for the Flora of Turkey. *Edinburgh Journal of Botany 48, 27–40. doi: 10.1017/S0960428600003577*

Tackholm, V. (1974). Student's Flora of Egypt. *University Press, Cairo.*

Takhtajian, A. (1954-2009). Flora Armenii. *Armenian Academy of Sciences.*

Takhtajian, A. and Fedorov, A. (1972). Flora Yerevana: Opredelitel dikorastushix rastenij Araratskoj kotloviny. *USSR Academy of Sciences.*

Termeh, F. (1975). Contribution à l'etude de quelques. Graminées nouvelles pour la Flore de l'Iran. *Ministere de l'Agriculture et des Ressources Naturelles*.

Termeh, F. (1987). Contribution à l'etude de quelques Graminées nouvelles pour la Flore de l'Iran. Fasc. 2. *Ministry of Agriculture, Plant Pests & Diseases Research Institute, Tehran*.

Termeh, F. (2000). New records of the family Gramineae from Iran (3). *Rostaniha 1, 43–62.*

Uotila, P., Sennikov, A., Danin, A. (2014.) The nomenclature of *Portulaca oleracea* and *P. sativa* (Portulacaceae). *Willdenowia* 42, 25–28. doi: 10.3372/wi.42.42102

Väre, H. and Kukkonen, I. (2005). Seven new species of *Cyperus* (Cyperaceae) section Arenarii and one new combination and typification. *Annales Botanici Fennici 42, 473–483.*

Varghese, M., Yadav, S. and Thomas, J. 2006. Taxonomic Status of Some of the *Tribulus* Species in the Indian Subcontinent. *Saudi Journal of Biological Sciences 3, 7–12.*

Verloove, F. and Sanchez Gullon, E. 2012. A taxonomic revision of non-native *Cenchrus s*.str. (Paniceae, Poaceae) in the Mediterranean area. *Willdenowia* 42, 67–75. doi: 10.3372/wi.42.42107

Watson, L., Macfarlane, T.D., and Dallwitz, M.J. (1992+). The grass genera of the world: descriptions, illustrations, identification, and information retrieval; including synonyms, morphology, anatomy, physiology, phytochemistry, cytology, classification, pathogens, world and local distribution, and references. delta-intkey.com [Accessed25.11.2019]

Yaprak, A. and Baskoese, I. (2012). *Atriplex lehmanniana* Bunge (Chenopodiaceae): A New record for The Flora of Turkey. Biological Diversity and Conservation 5, 66–69.

Zohary, M. (1966). Flora Palaestina. I*srael Academy of sciences and humanities.*
